# Supplementary material for: Cold exposure promotes the progression of osteoarthritis through downregulating APOE in cartilage
Source: EMBO Mol Med. 2025 Jul 15;17(8):2137–62. doi: 10.1038/s44321-025-00268-6 (PMC12340072; doi:10.1038/s44321-025-00268-6)
Supplement: Supplementary file 1 — Table EV1 [file 44321_2025_268_MOESM1_ESM.docx]

**Table EV1.** Demographic data of total knee replacement patients.

| Sex | Age | City | South/North | BMI  (Kg/cm^2) | TG  (mmol/L) | TC  (mmol/L) | LDL  (mmol/L) |
| --- | --- | --- | --- | --- | --- | --- | --- |
| M | 67 | Shanghai | South | 26.53 | 1.58 | 6.89 | 4.41 |
| F | 73 | Shanghai | South | 24.03 | 1.72 | 6.23 | 4.04 |
| M | 77 | Shanghai | South | 25.76 | 1.63 | 5.79 | 3.59 |
| F | 69 | Guangzhou | South | 25.1 | 1.32 | 5.21 | 3.07 |
| F | 70 | Guangzhou | South | 28.13 | 1.35 | 5.39 | 3.39 |
| F | 81 | Beijing | North | 22.03 | 1.46 | 5.46 | 2.37 |
| M | 65 | Shen  yang | North | 27.13 | 1.59 | 6.02 | 4.13 |
| F | 68 | Jinan | North | 27.06 | 1.74 | 6.52 | 4.09 |
| F | 72 | Jinan | North | 23.83 | 1.53 | 5.79 | 3.22 |
| M | 75 | Beijing | North | 25.35 | 1.75 | 6.39 | 4.14 |
| M | 67 | Shanghai | South | 26.53 | 1.60 | 5.12 | 3.98 |
| F | 73 | Shanghai | South | 24.03 | 1.32 | 5.02 | 3.14 |
| M | 77 | Shanghai | South | 25.76 | 1.62 | 5.89 | 3.62 |
| F | 69 | Guangzhou | South | 25.1 | 1.58 | 5.72 | 3.32 |
| F | 70 | Guangzhou | South | 28.13 | 1.60 | 5.23 | 2.87 |
| F | 81 | Beijing | North | 22.03 | 1.22 | 5.11 | 2.58 |
| M | 65 | Shen  yang | North | 27.13 | 1.73 | 6.48 | 4.56 |
| F | 68 | Jinan | North | 27.06 | 1.64 | 5.32 | 3.39 |
| F | 72 | Jinan | North | 23.83 | 1.47 | 5.47 | 3.26 |
| M | 75 | Beijing | North | 25.35 | 1.59 | 5.89 | 3.96 |
