# Supplementary material for: Cold exposure promotes the progression of osteoarthritis through downregulating APOE in cartilage
Source: EMBO Mol Med. 2025 Jul 15;17(8):2137–62. doi: 10.1038/s44321-025-00268-6 (PMC12340072; doi:10.1038/s44321-025-00268-6)
Supplement: Supplementary file 2 — Table EV2 [file 44321_2025_268_MOESM2_ESM.docx]

**Table EV2.** Primer information for genotyping of transgene mice and qPCR.

| Apoe flox | 5'arm | Forward | TTAGAGAAAGCTTGGCCAGGCATG |
| --- | --- | --- | --- |
|  |  | Reverse | ACATTTCCTGTTTCAGCCTGGGAA |
|  | 3'arm | Forward | TCTGCTCCTCTGTGGACTCTATTCTA |
|  |  | Reverse | TTGGAGGTCAGAGTTCTAGAGGGAA |
| Acan Cre | 5'arm | Forward | TGTTAAAGCCTTCAGGTGGTTGG |
|  |  | Reverse | CATGTCCATCAGGTTCTTGCGAAC |
|  | WT | Forward | TGGGAGAACCAATGTACCACCA |
|  |  | Reverse | ACTTGTACCCTGTATTCGGAACCC |
| Gapdh | | Forward | GGCAAATTCAACGGCACAGTCAAG |
|  |  | Reverse | TCGCTCCTGGAAGATGGTGATGG |
| Col2a1 | | Forward | GGTCCTCCTGGTCCTGGCATC |
|  |  | Reverse | CGTGCTGTCTCAAGGTACTGTCTG |
| Acan | | Forward | GGAGACCCAGACAGCAGAAACAAC |
|  |  | Reverse | GCAGGTGGCTCCATTCAGACAAG |
| Mmp13 | | Forward | ACAGTTGACAGGCTCCGAGAAATG |
|  |  | Reverse | CCACATCAGGCACTCCACATCTTG |
| Adamts5 | | Forward | CCGAGGAACTCCCAGGACAGAC |
|  |  | Reverse | CGCACCACAGCACACCACAG |
