# Supplementary material for: Cold exposure promotes the progression of osteoarthritis through downregulating APOE in cartilage
Source: EMBO Mol Med. 2025 Jul 15;17(8):2137–62. doi: 10.1038/s44321-025-00268-6 (PMC12340072; doi:10.1038/s44321-025-00268-6)

Appendix

Table of Content

**Appendix Figure S1**	**P1**
**Appendix Figure S2**	**P2**
**Appendix Figure S3**	**P3**
**Appendix Figure S4**	**P4**
**Appendix Figure S5**	**P5**

Appendix Figure S1. Photographs of the measurement of mice knee articular temperatures using a thermo probe.

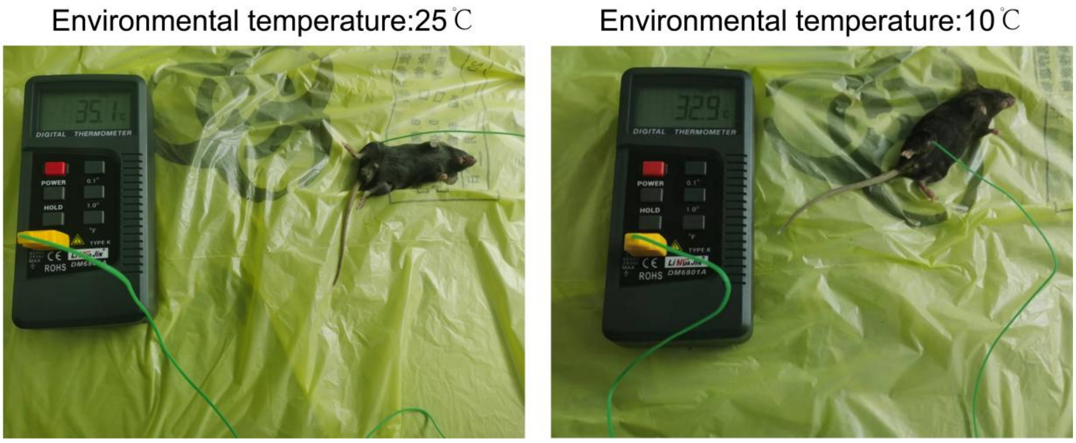

Appendix Figure S2. Body weights of wildtype, *Apoe* $^{f/f}$ and *Apoe* $^{-/-}$ mice at room and low temperature (n=5 mice per group). Statistical analysis was performed using One-way ANOVA test. Data are shown as mean ± SD (error bar).

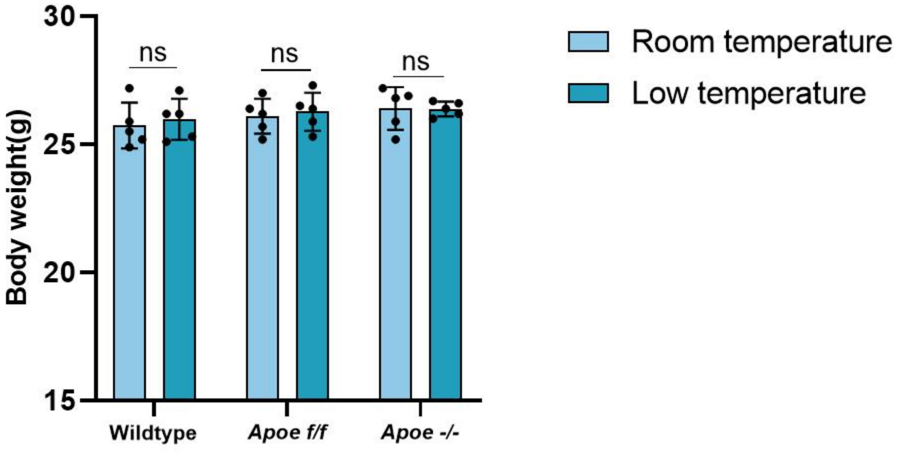

Appendix Figure S3. The result of qPCR for Col2a1, Acan, Mmp13 and Admts5 in cartilage of DMM mice under room and low temperature. mRNA fold change of (A) Col2a1 (n=5 mice per group). (B) Acan (n=5 mice per group). (C) Mmp13 (n=5 mice per group). (D) Adamts5 (n=5 mice per group). Statistical analysis was performed using One-way ANOVA test (A-D). Data are shown as mean ± SD(*p<0.05,**p<0.01,***p<0.001).

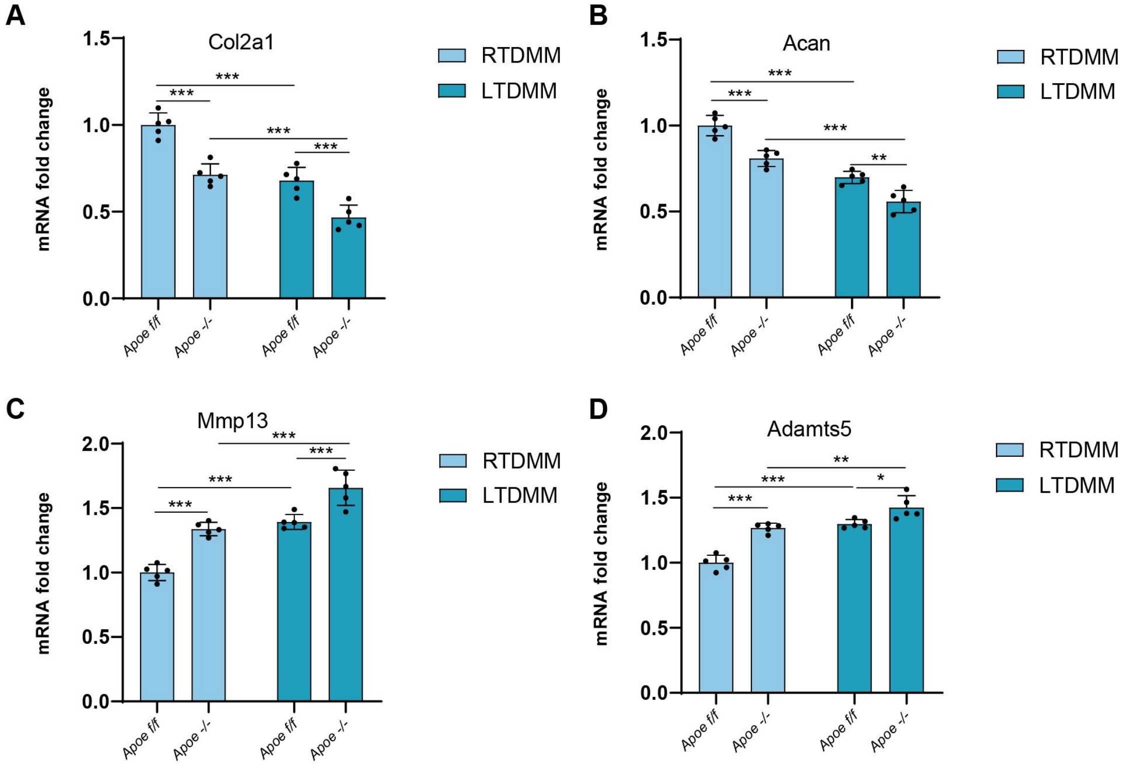

Appendix Figure S4. The result of qPCR for Acan, Col2a1, Mmp13, Adamts5 and Apoe in cartilage of different DMM mice under RT and LT with RGX-104 treatment. mRNA fold change of (A) Acan (n=5 mice per group) (B) Col2a1 (n=5 mice per group) (C) Mmp13 (n=5 mice per group). (D)Adamts5 (n=5 mice per group). (E) Apoe (n=5 mice per group). Statistical analysis was performed using One-way ANOVA test (A-D). Data are shown as mean ± SD (error bar) (*p<0.05, **p<0.01,***p<0.001).

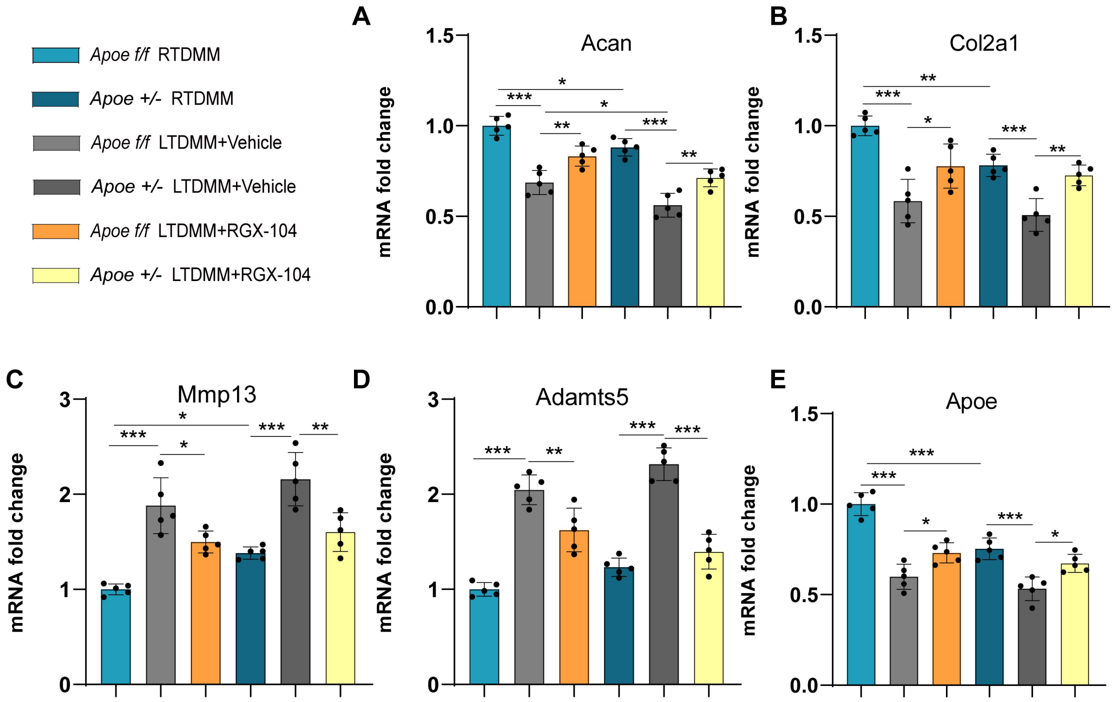

Appendix Figure S5. Representative hematoxylin and eosin (H&E)-stained sections of heart, liver, spleen, lung, and kidney from mice treated with vehicle control (n=10) or RGX-104 (n=10) articular injection.

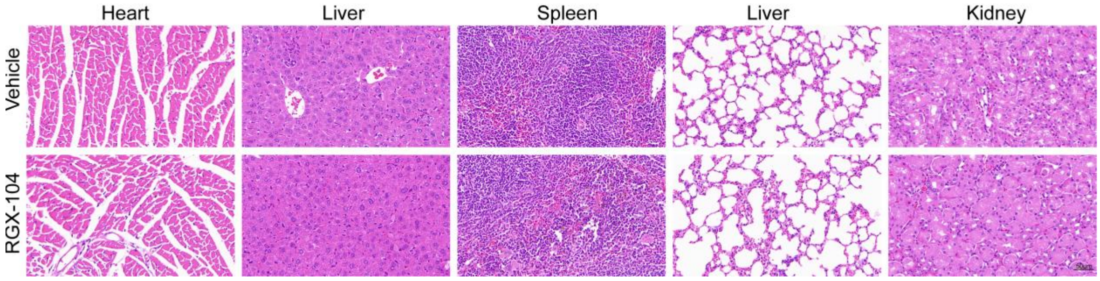

Supplement: Supplementary file 3 — Appendix [file 44321_2025_268_MOESM3_ESM.pdf]
