# Supplementary figures and images for: Cold exposure promotes the progression of osteoarthritis through downregulating APOE in cartilage (part 1 of 3)
Source: EMBO Mol Med. 2025 Jul 15;17(8):2137–62. doi: 10.1038/s44321-025-00268-6 (PMC12340072; doi:10.1038/s44321-025-00268-6)

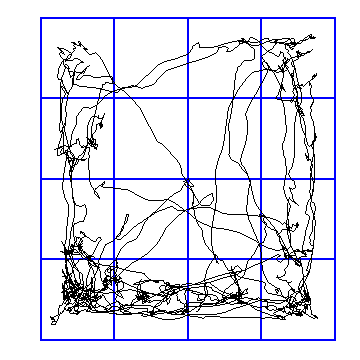

Supplement: Supplementary file 5 — Source data Fig. 1 [file 44321_2025_268_MOESM5_ESM.zip › Figure 1/1C/LT.tif]

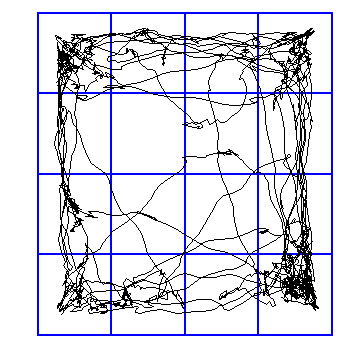

Supplement: Supplementary file 5 — Source data Fig. 1 [file 44321_2025_268_MOESM5_ESM.zip › Figure 1/1C/RT.tif]

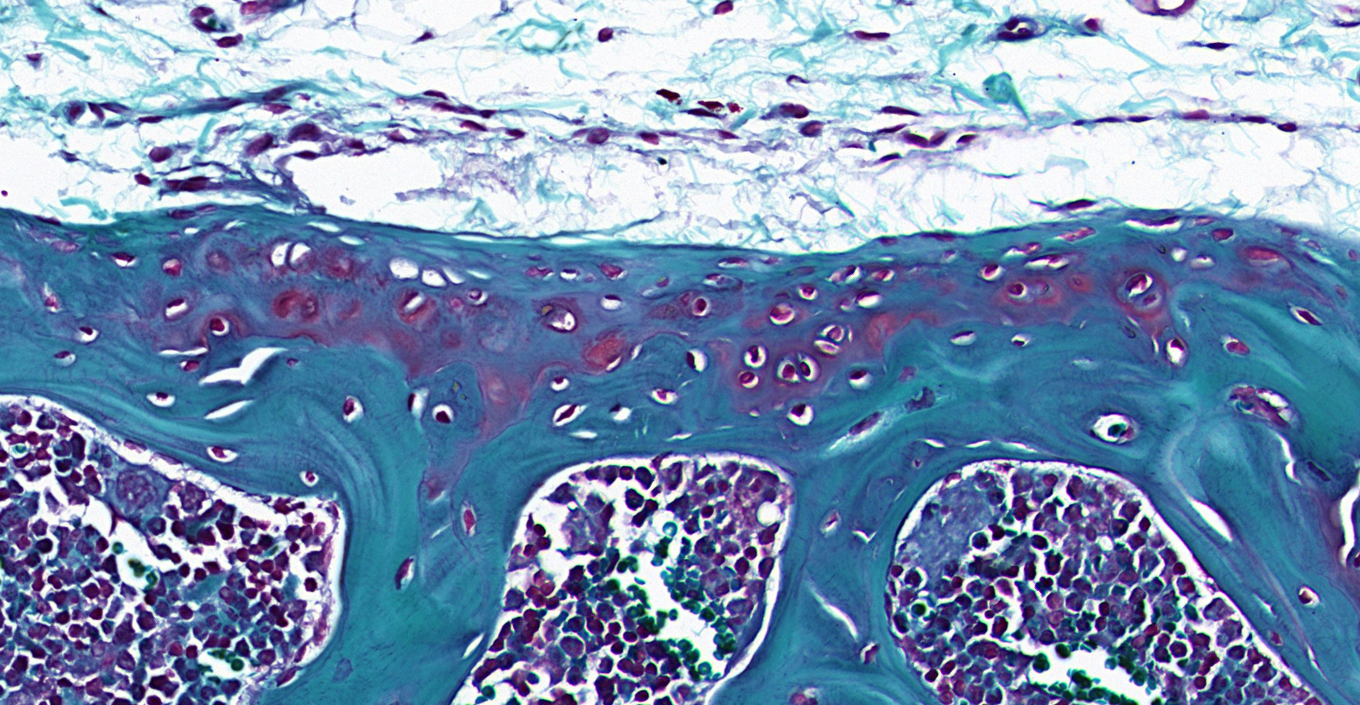

Supplement: Supplementary file 5 — Source data Fig. 1 [file 44321_2025_268_MOESM5_ESM.zip › Figure 1/1J/DMM LT.tif]

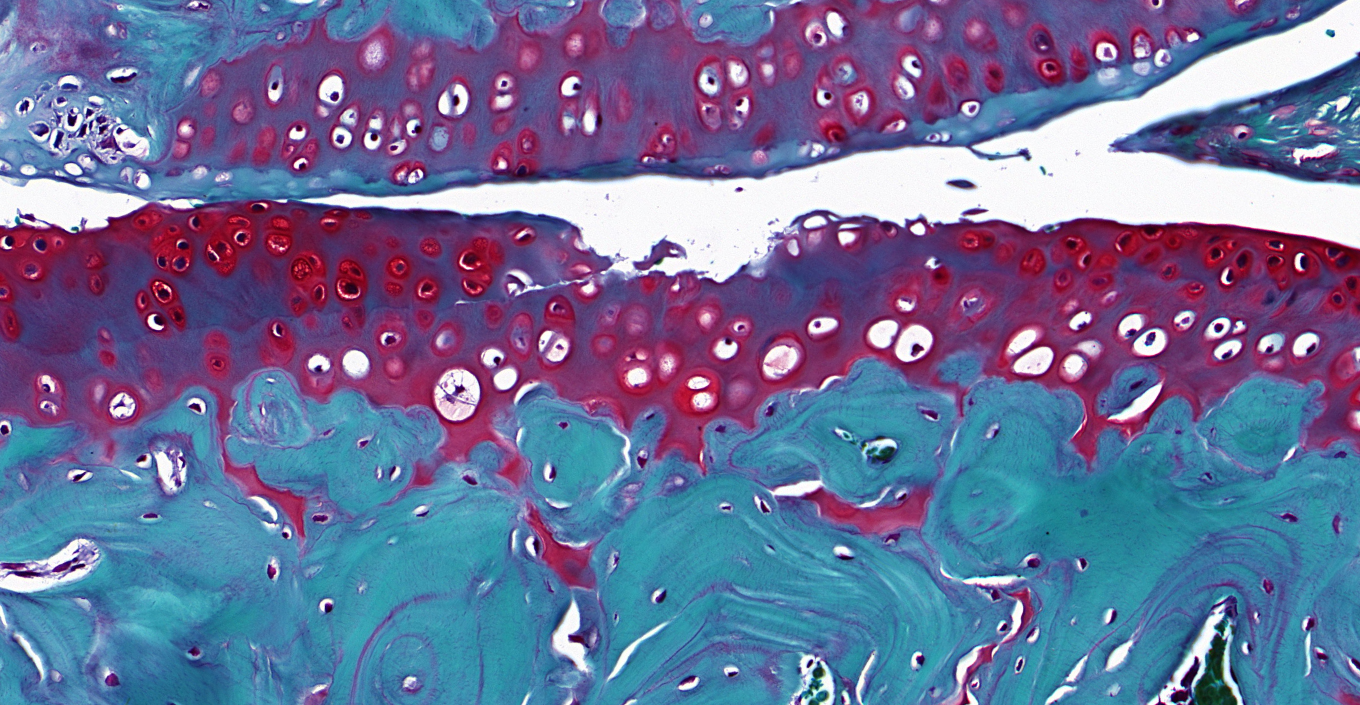

Supplement: Supplementary file 5 — Source data Fig. 1 [file 44321_2025_268_MOESM5_ESM.zip › Figure 1/1J/DMM RT.tif]

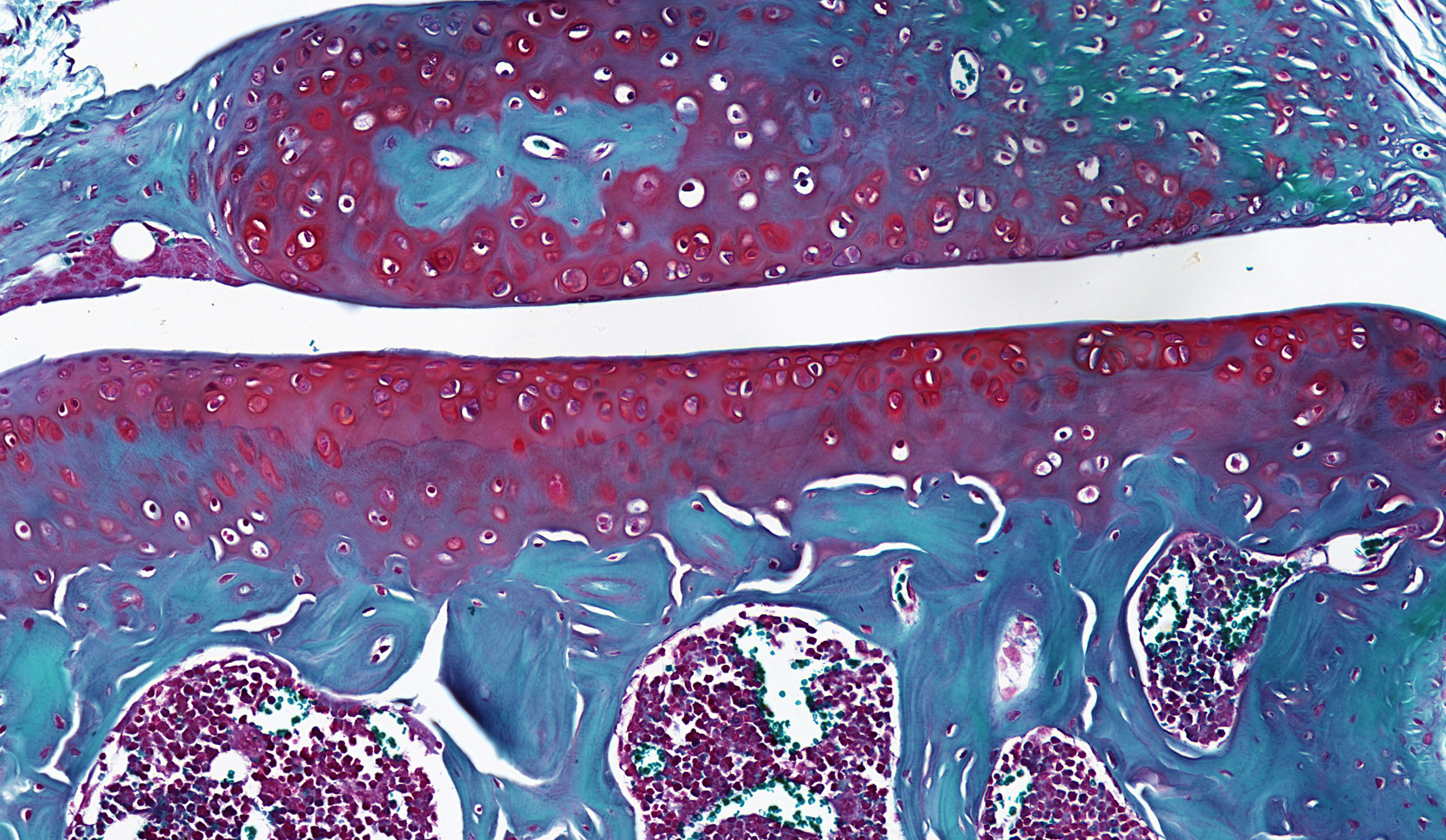

Supplement: Supplementary file 5 — Source data Fig. 1 [file 44321_2025_268_MOESM5_ESM.zip › Figure 1/1J/SHAM LT.tif]

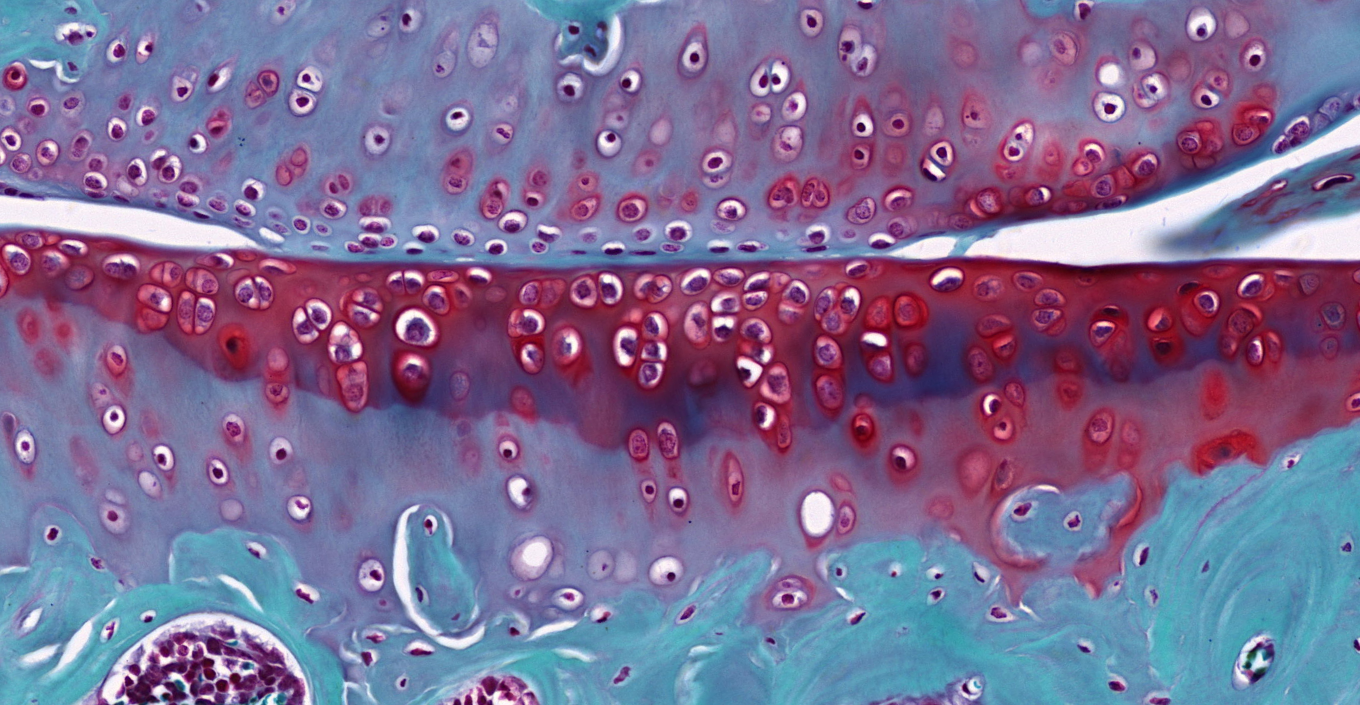

Supplement: Supplementary file 5 — Source data Fig. 1 [file 44321_2025_268_MOESM5_ESM.zip › Figure 1/1J/SHAM RT.tif]

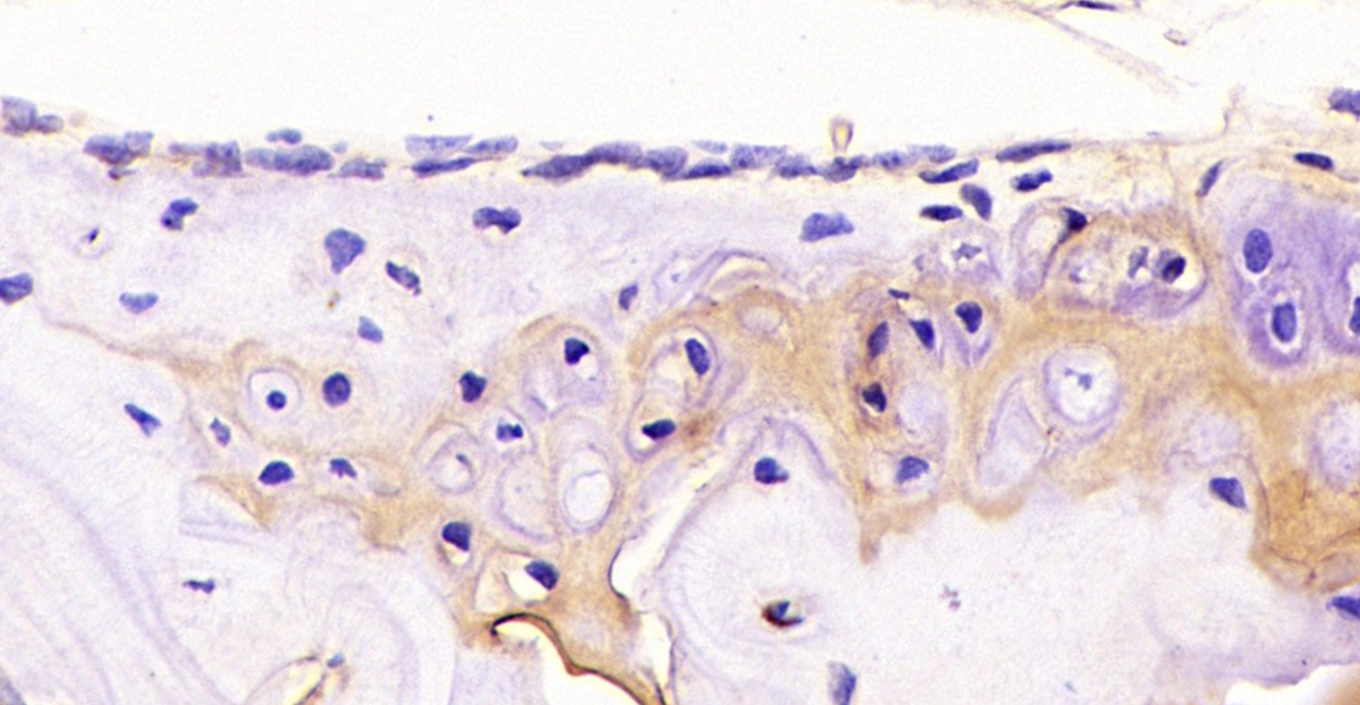

Supplement: Supplementary file 5 — Source data Fig. 1 [file 44321_2025_268_MOESM5_ESM.zip › Figure 1/1K/LT DMM.tif]

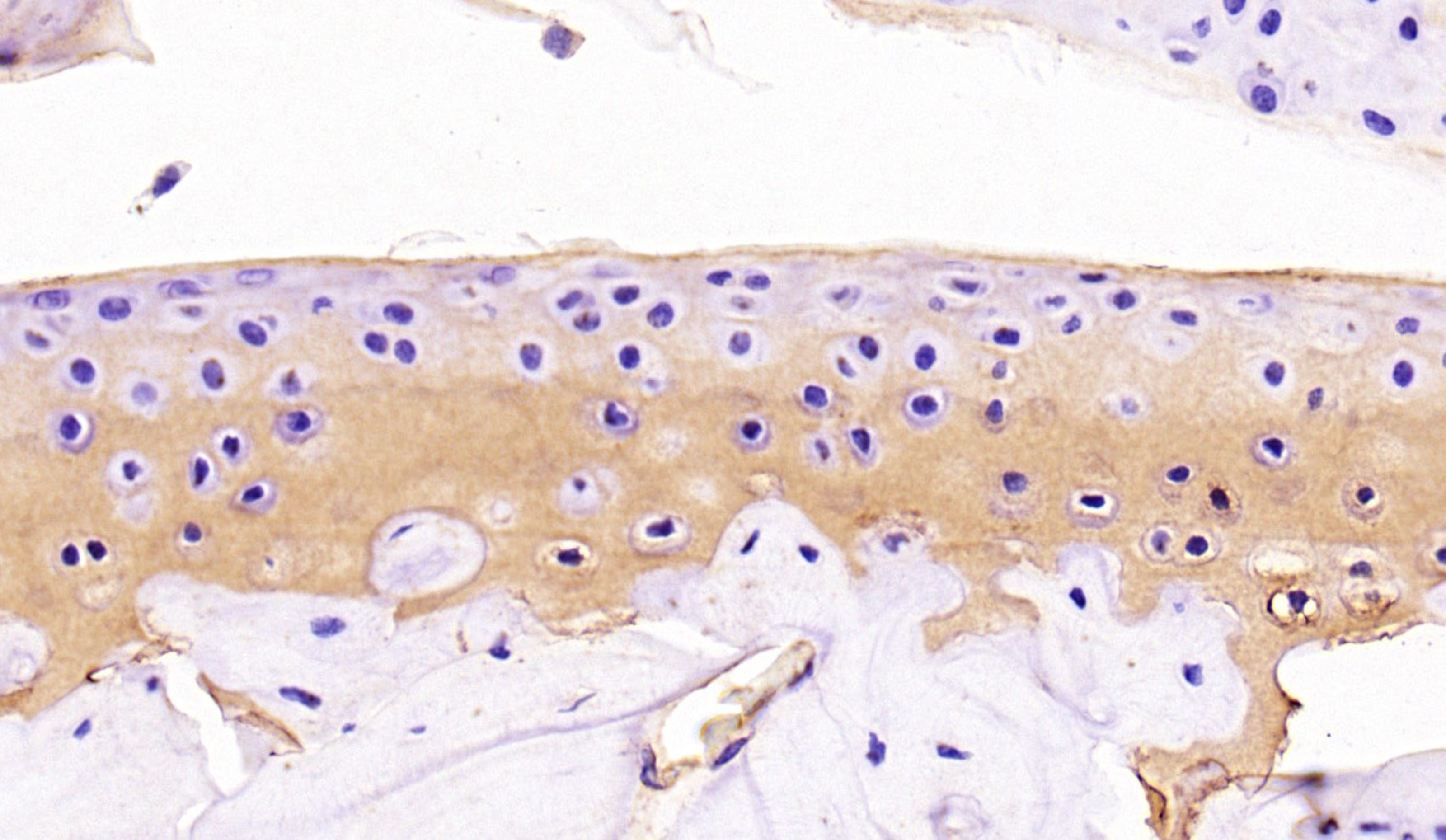

Supplement: Supplementary file 5 — Source data Fig. 1 [file 44321_2025_268_MOESM5_ESM.zip › Figure 1/1K/LTSHAM.tif]

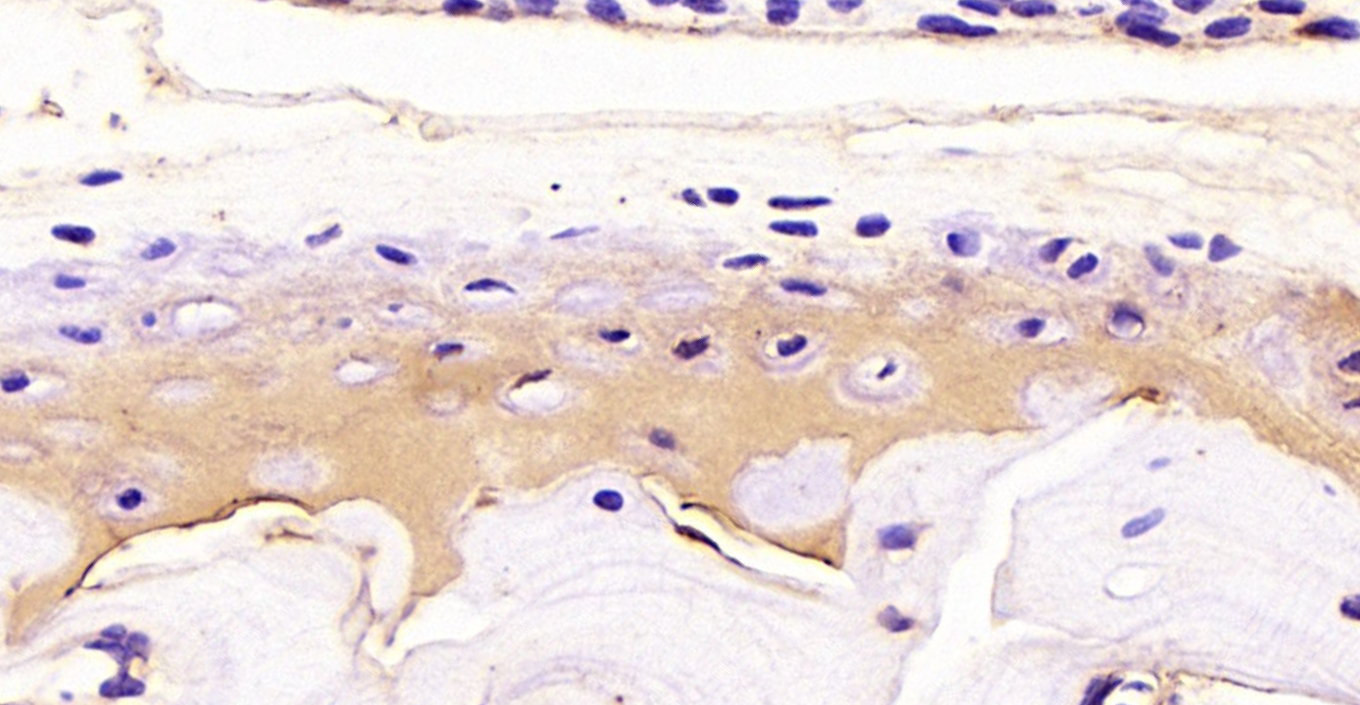

Supplement: Supplementary file 5 — Source data Fig. 1 [file 44321_2025_268_MOESM5_ESM.zip › Figure 1/1K/RT DMM.tif]

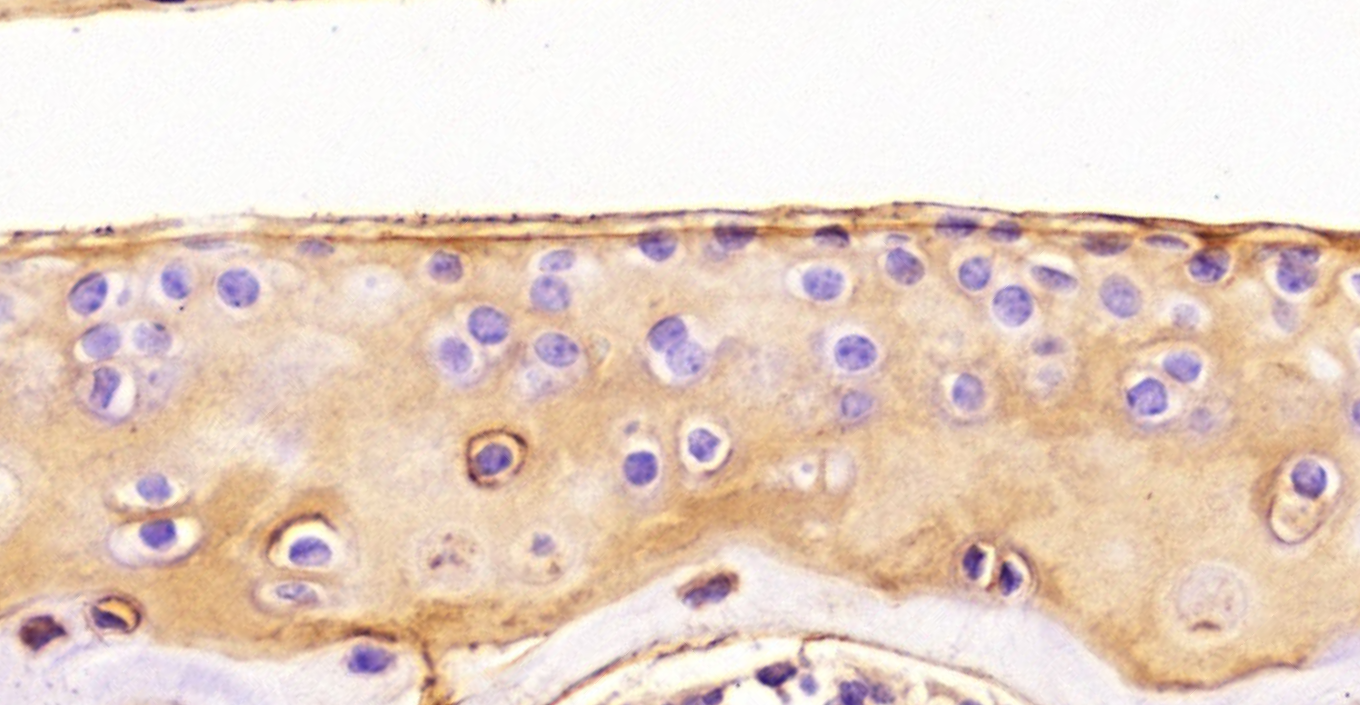

Supplement: Supplementary file 5 — Source data Fig. 1 [file 44321_2025_268_MOESM5_ESM.zip › Figure 1/1K/RT SHAM.tif]

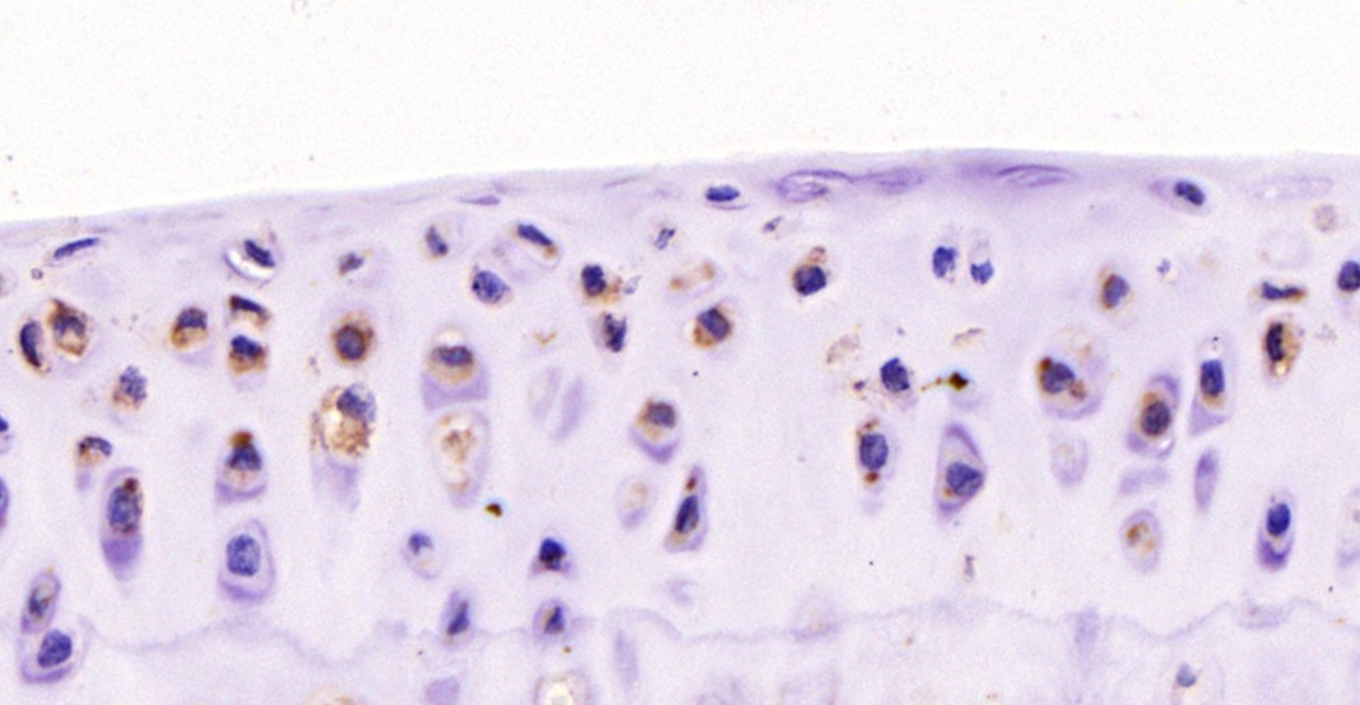

Supplement: Supplementary file 5 — Source data Fig. 1 [file 44321_2025_268_MOESM5_ESM.zip › Figure 1/1L/LT DMM.tif]

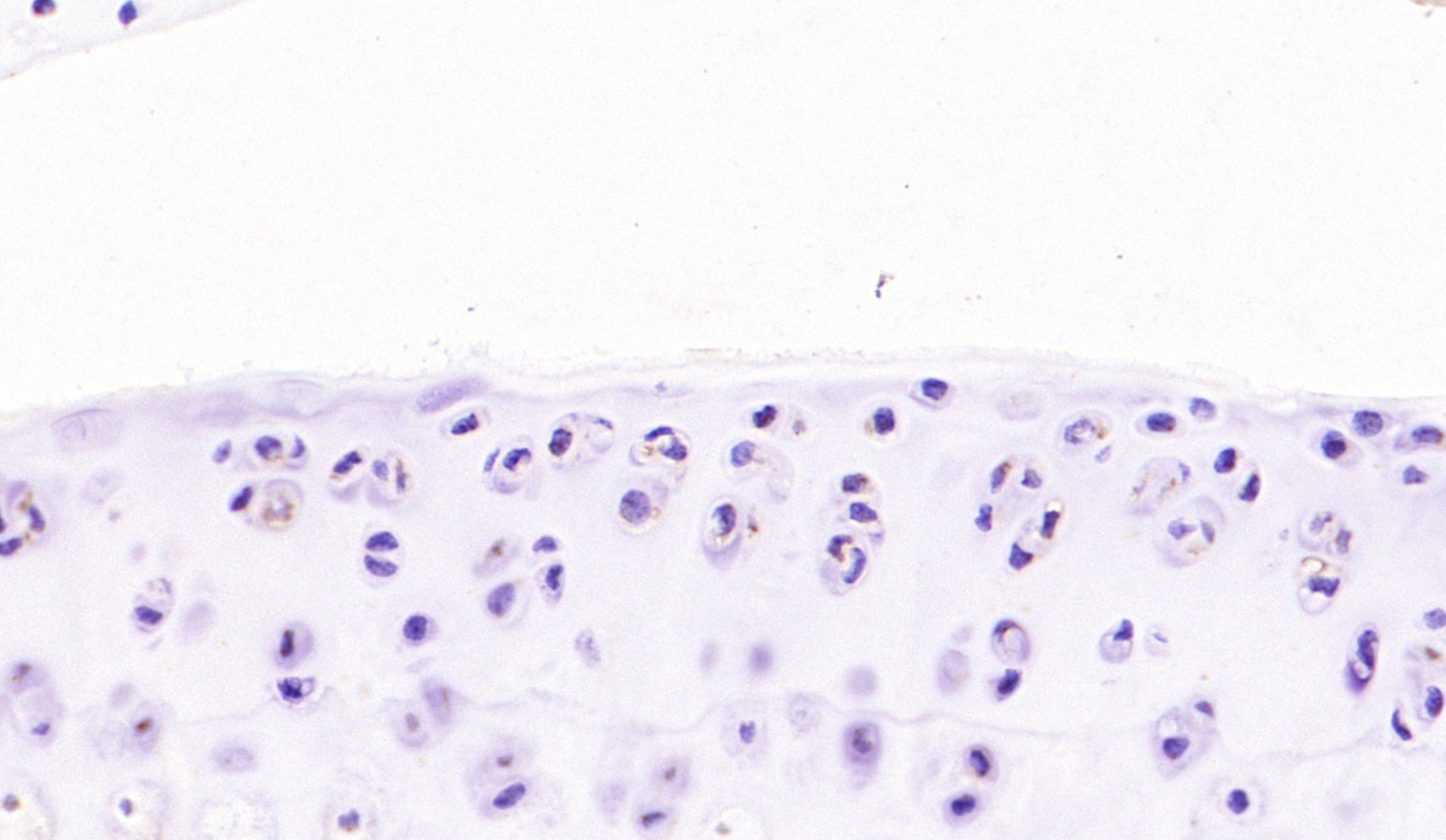

Supplement: Supplementary file 5 — Source data Fig. 1 [file 44321_2025_268_MOESM5_ESM.zip › Figure 1/1L/LTSHAM.tif]

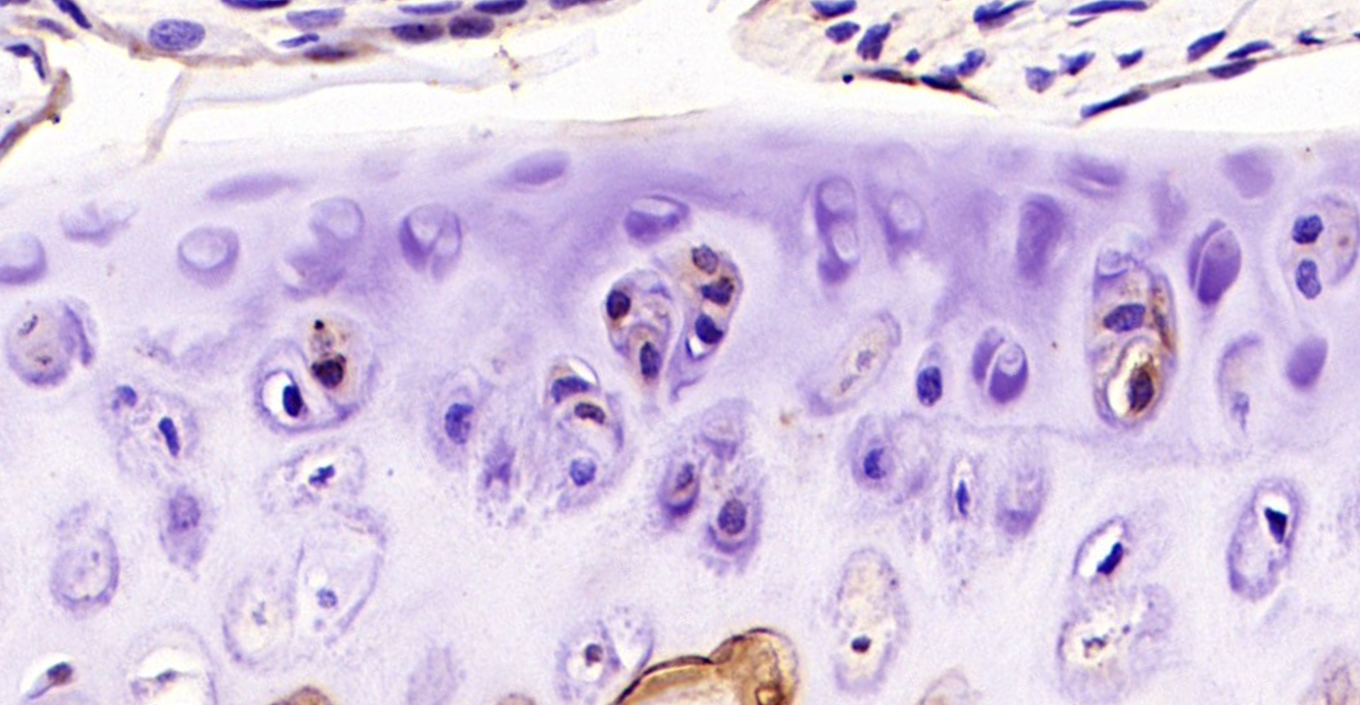

Supplement: Supplementary file 5 — Source data Fig. 1 [file 44321_2025_268_MOESM5_ESM.zip › Figure 1/1L/RT DMM.tif]

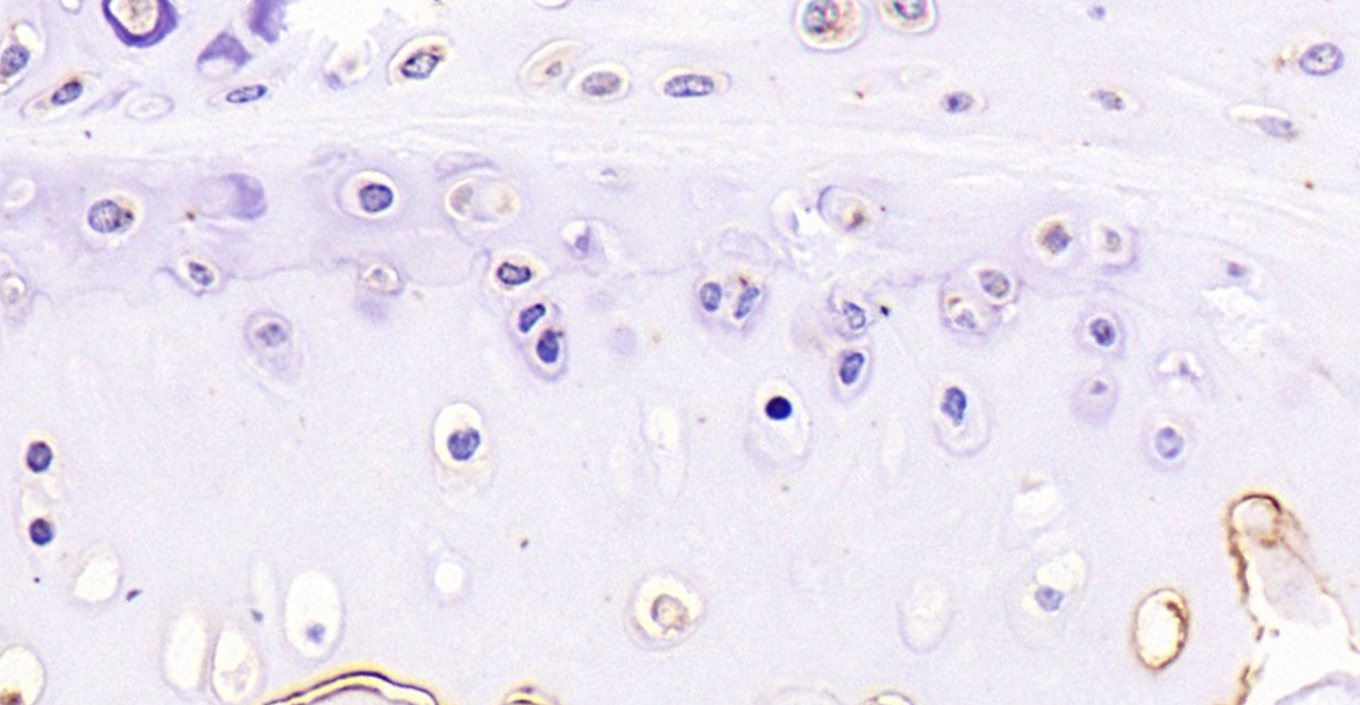

Supplement: Supplementary file 5 — Source data Fig. 1 [file 44321_2025_268_MOESM5_ESM.zip › Figure 1/1L/RT SHAM.tif]

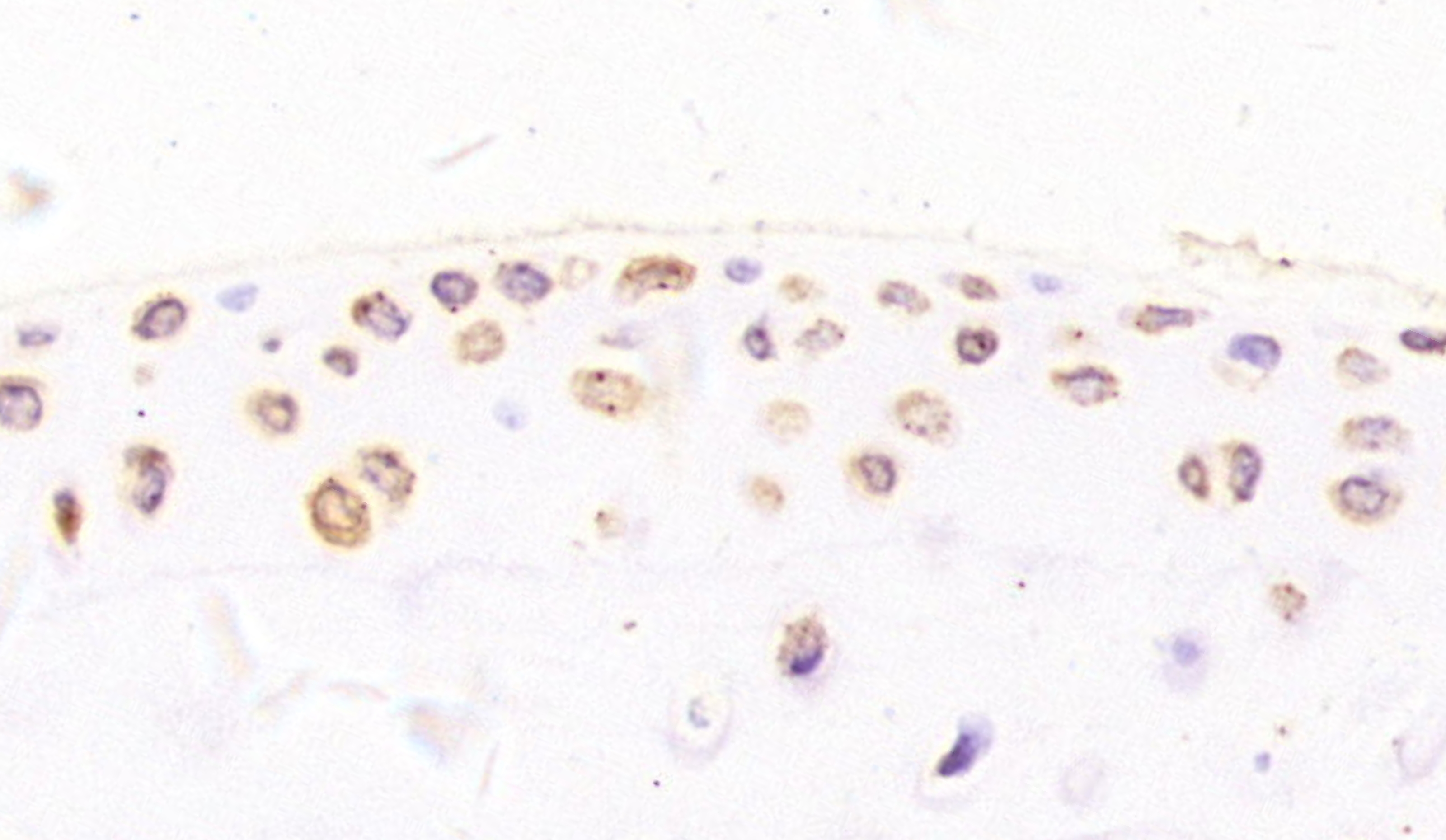

Supplement: Supplementary file 6 — Source data Fig. 2 [file 44321_2025_268_MOESM6_ESM.zip › Figure 2/2E/LT SHAM.tif]

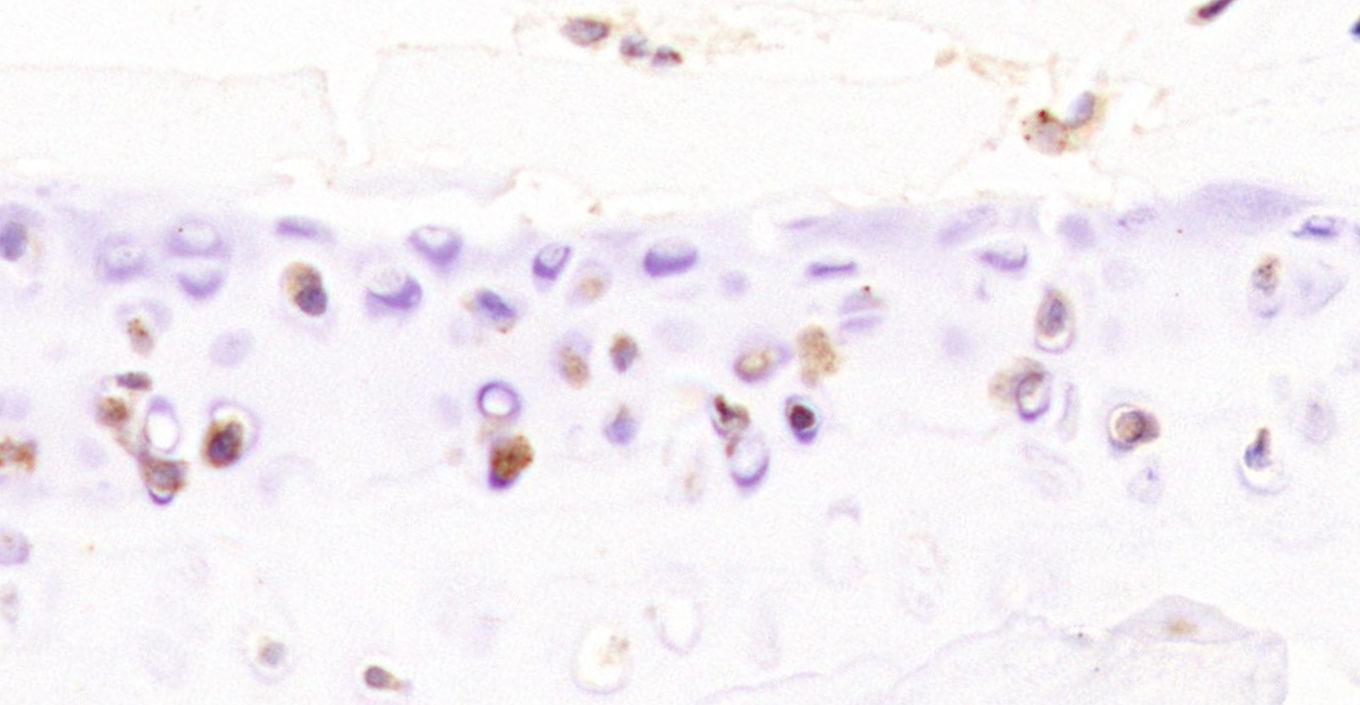

Supplement: Supplementary file 6 — Source data Fig. 2 [file 44321_2025_268_MOESM6_ESM.zip › Figure 2/2E/LTDMM.tif]

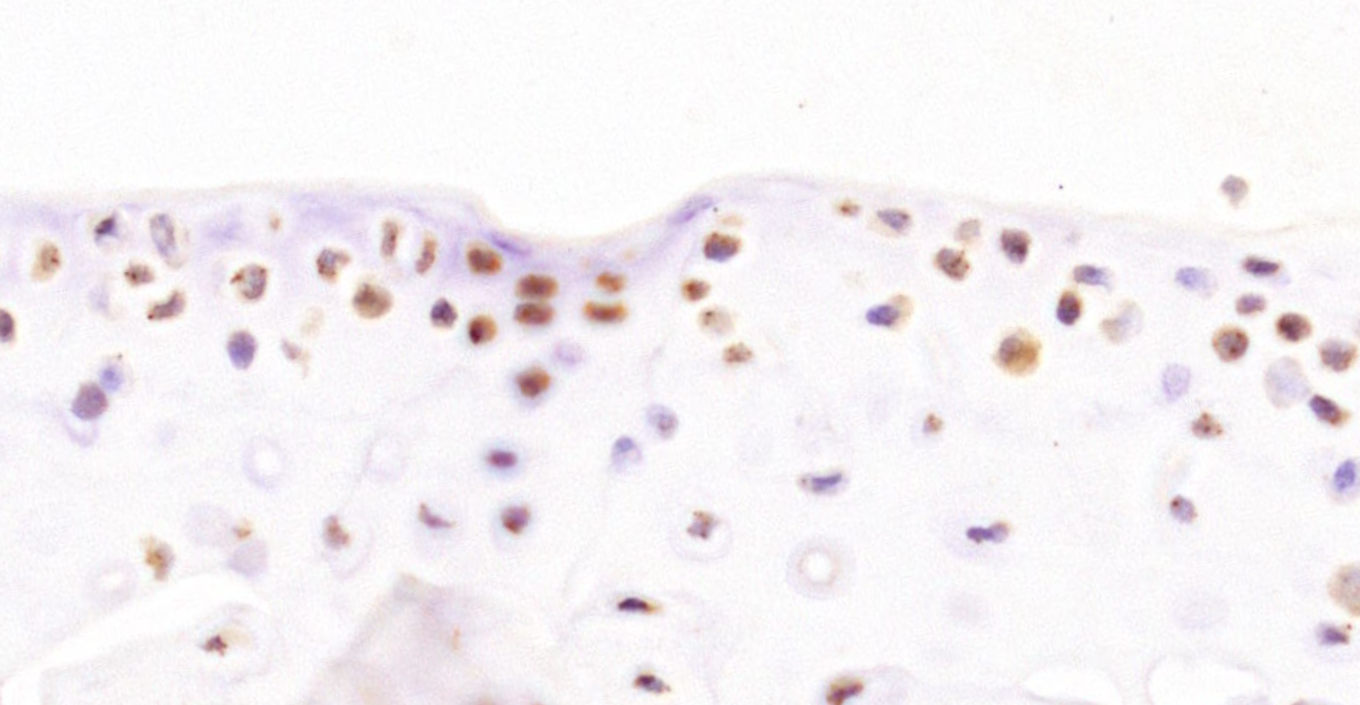

Supplement: Supplementary file 6 — Source data Fig. 2 [file 44321_2025_268_MOESM6_ESM.zip › Figure 2/2E/RT DMM.tif]

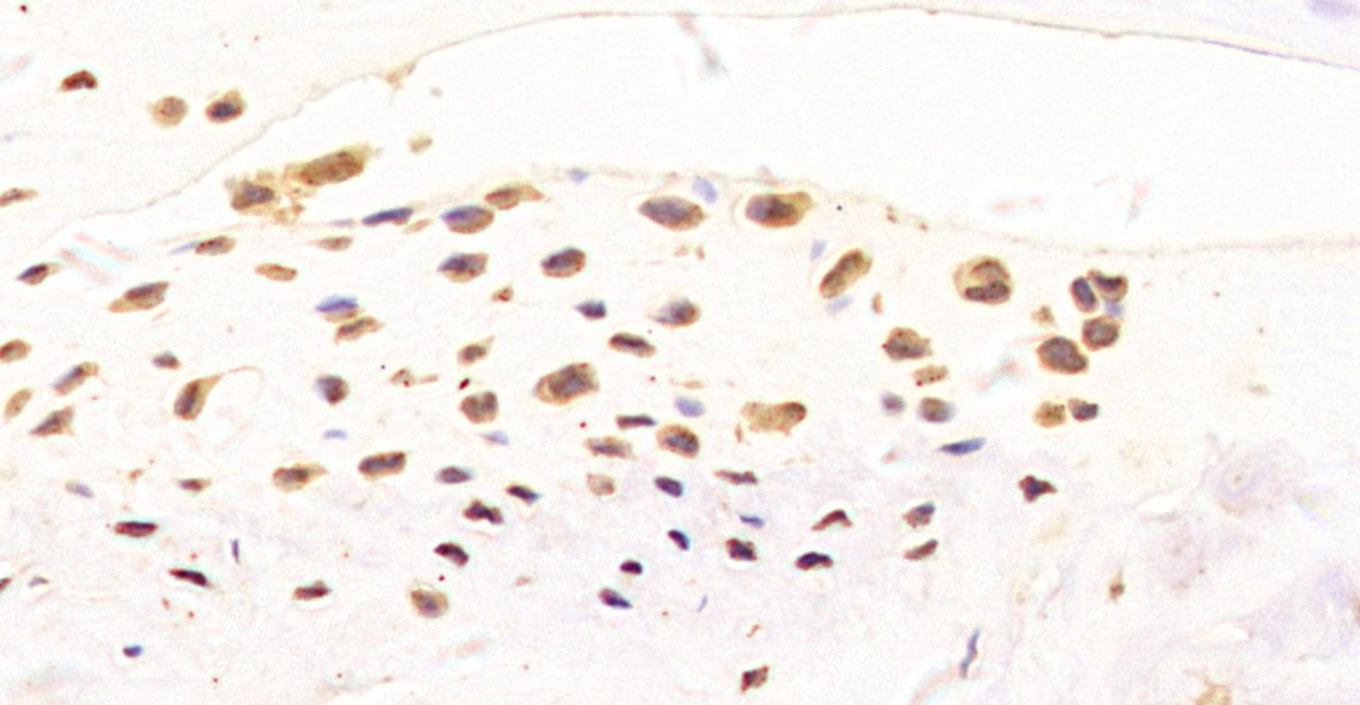

Supplement: Supplementary file 6 — Source data Fig. 2 [file 44321_2025_268_MOESM6_ESM.zip › Figure 2/2E/RT SHAM.tif]

Figure 2F

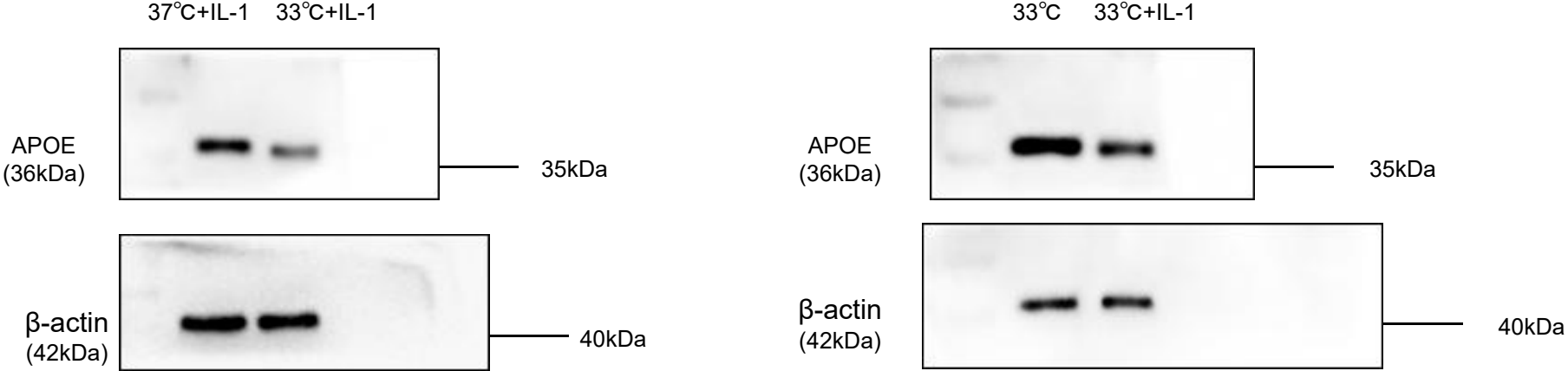

Supplement: Supplementary file 6 — Source data Fig. 2 [file 44321_2025_268_MOESM6_ESM.zip › Figure 2/2F/2F.pdf]

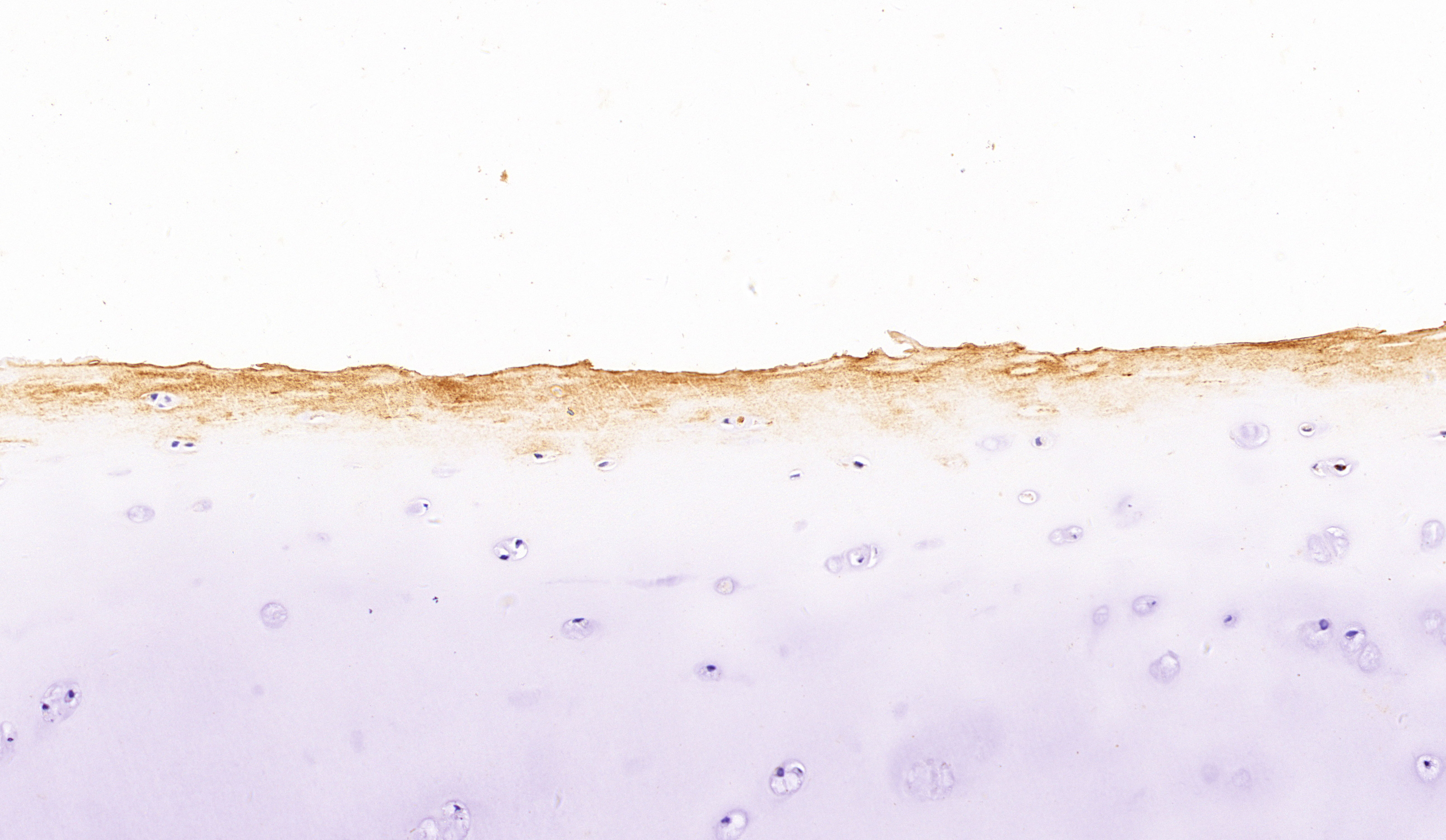

Supplement: Supplementary file 6 — Source data Fig. 2 [file 44321_2025_268_MOESM6_ESM.zip › Figure 2/2J/APOE North LC.tif]

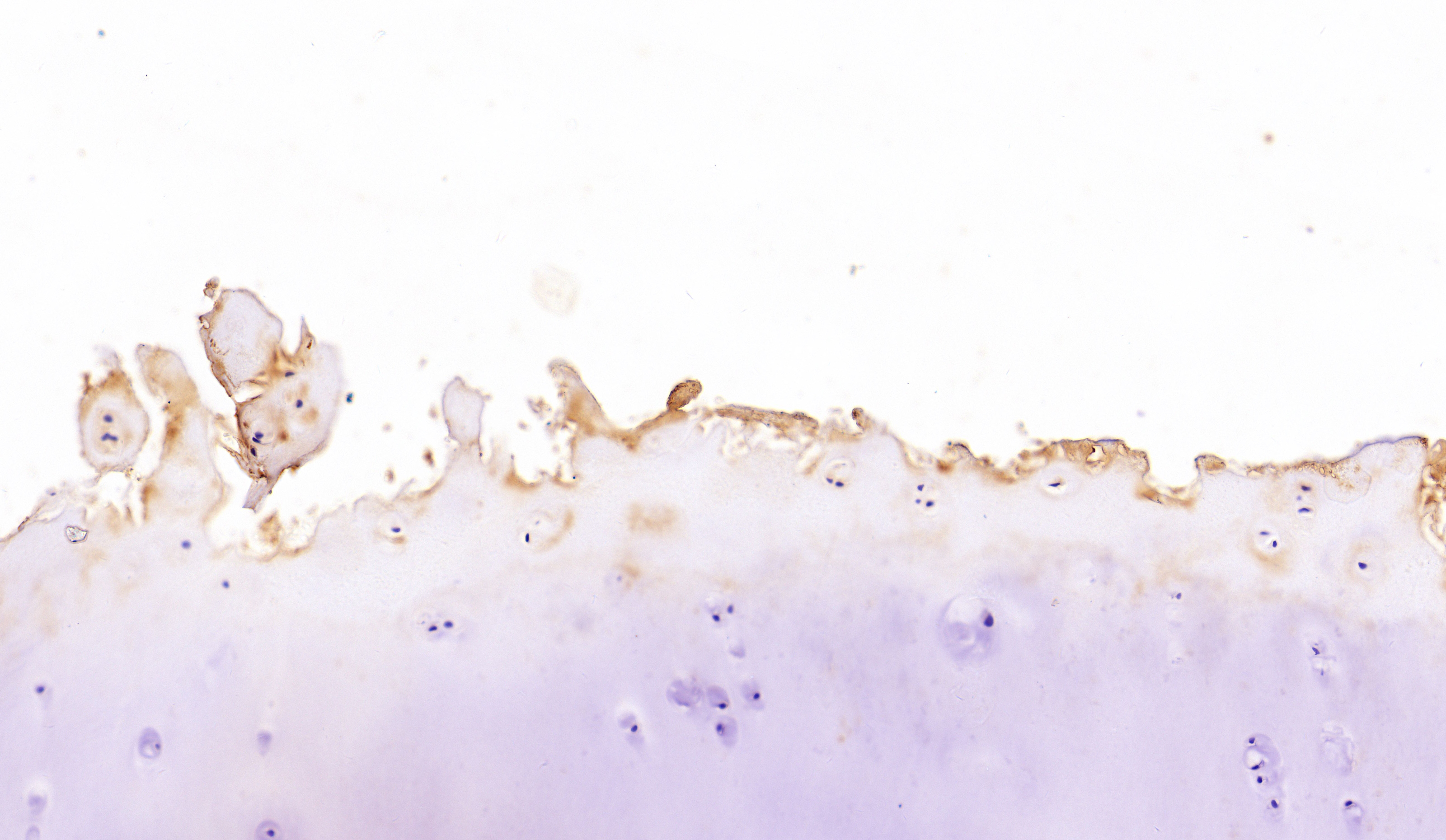

Supplement: Supplementary file 6 — Source data Fig. 2 [file 44321_2025_268_MOESM6_ESM.zip › Figure 2/2J/APOE North MC.tif]

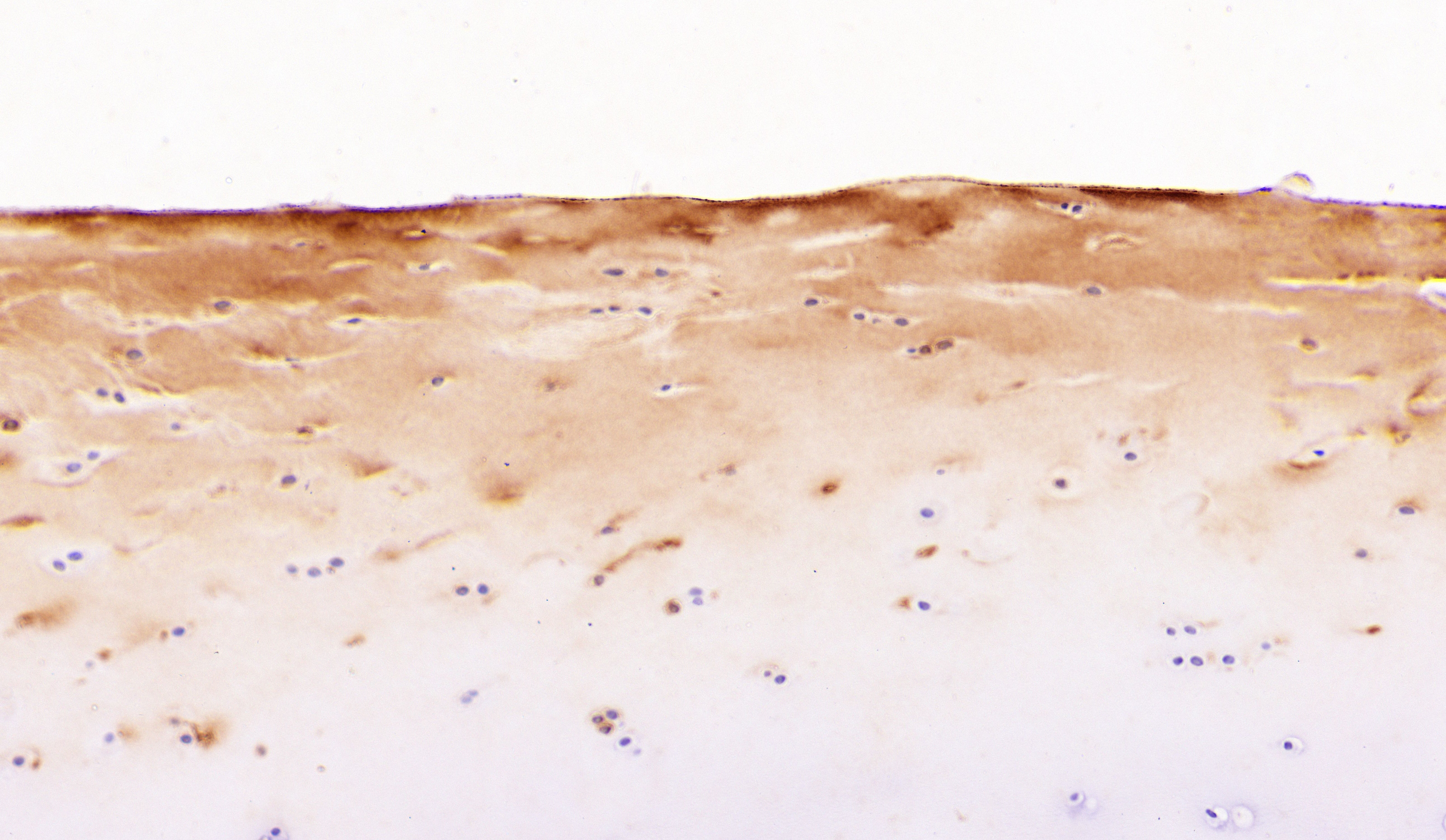

Supplement: Supplementary file 6 — Source data Fig. 2 [file 44321_2025_268_MOESM6_ESM.zip › Figure 2/2J/APOE South LC.tif]

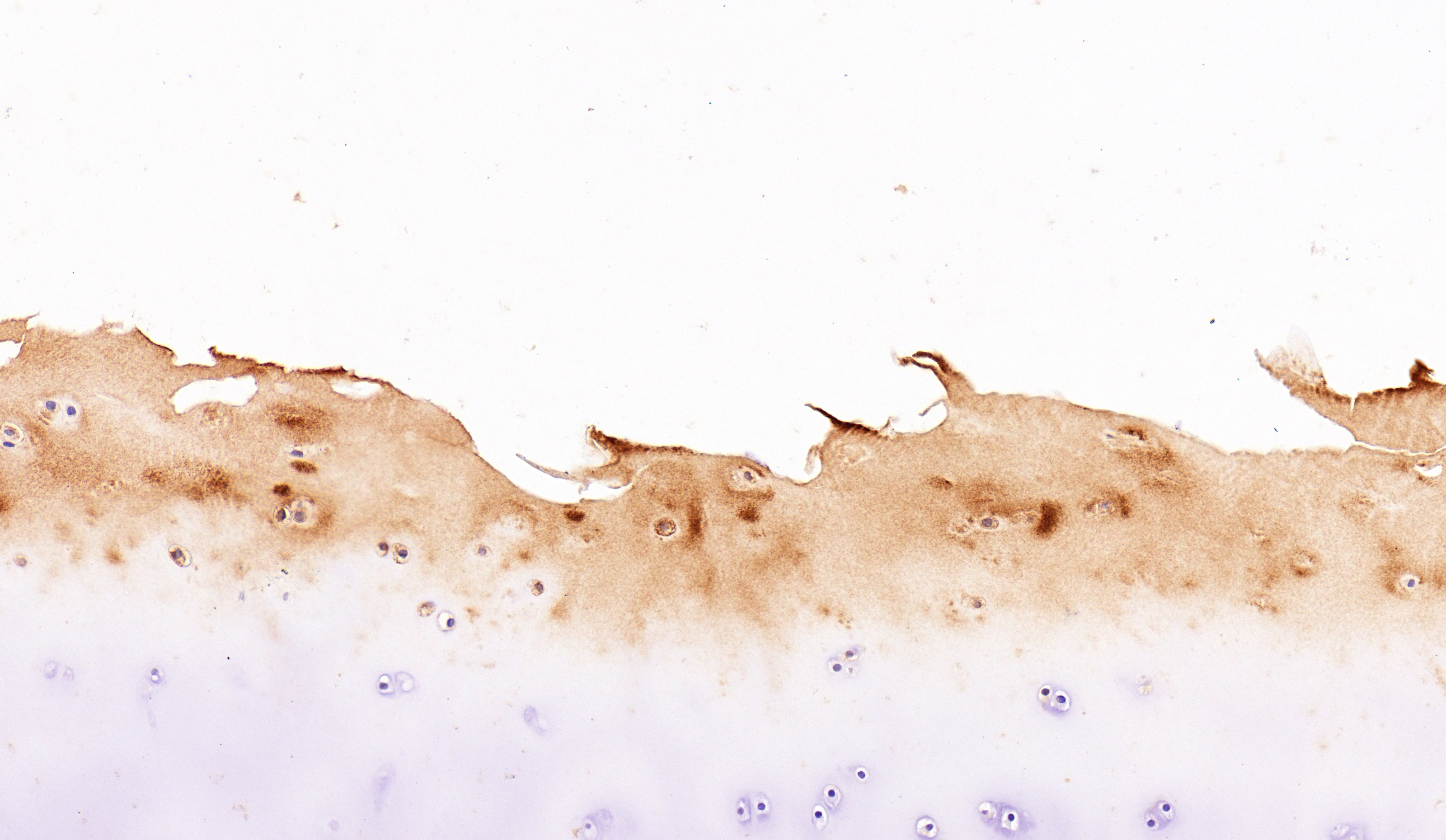

Supplement: Supplementary file 6 — Source data Fig. 2 [file 44321_2025_268_MOESM6_ESM.zip › Figure 2/2J/APOE South MC.tif]

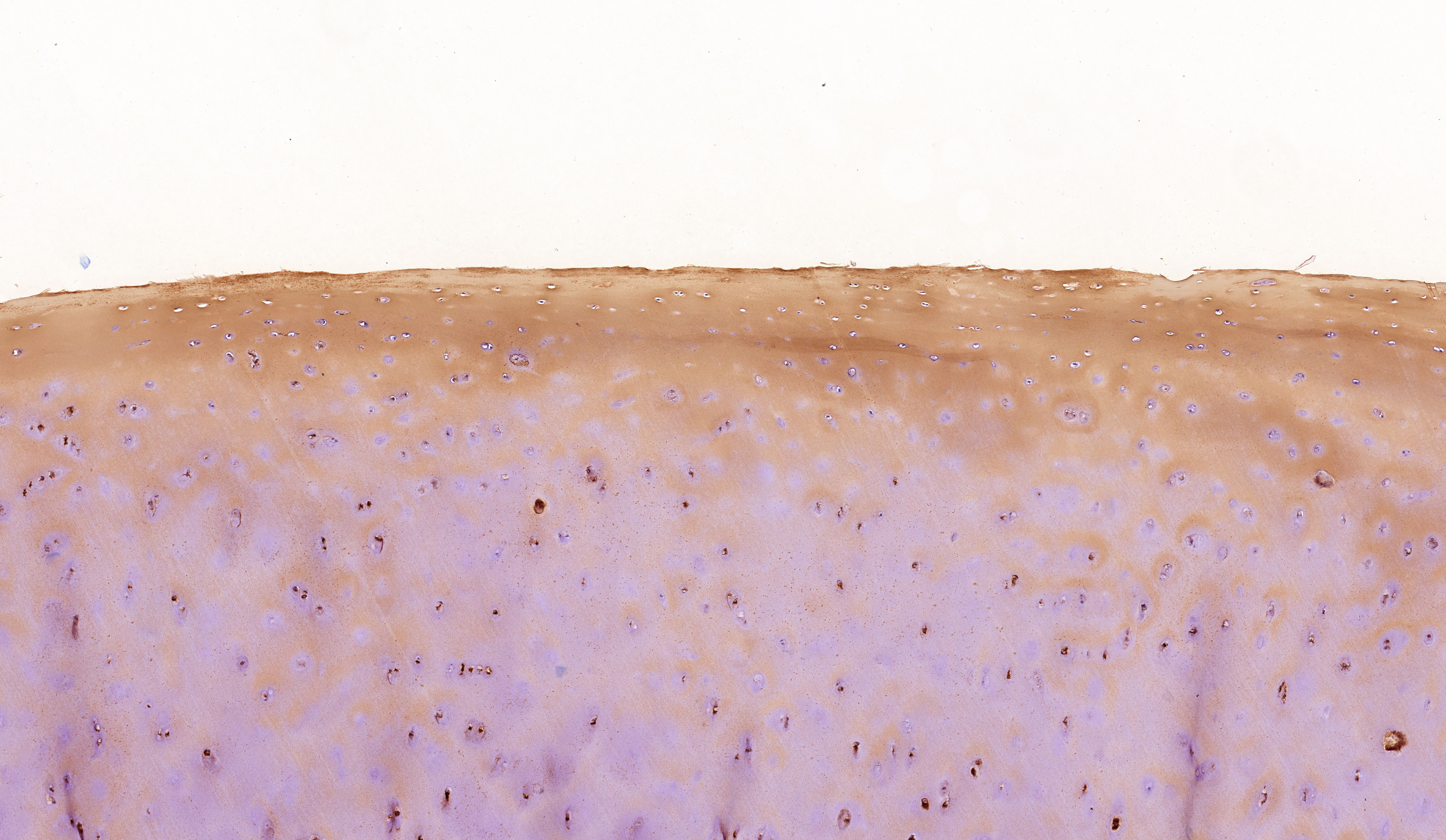

Supplement: Supplementary file 6 — Source data Fig. 2 [file 44321_2025_268_MOESM6_ESM.zip › Figure 2/2J/COLII North LC.tif]

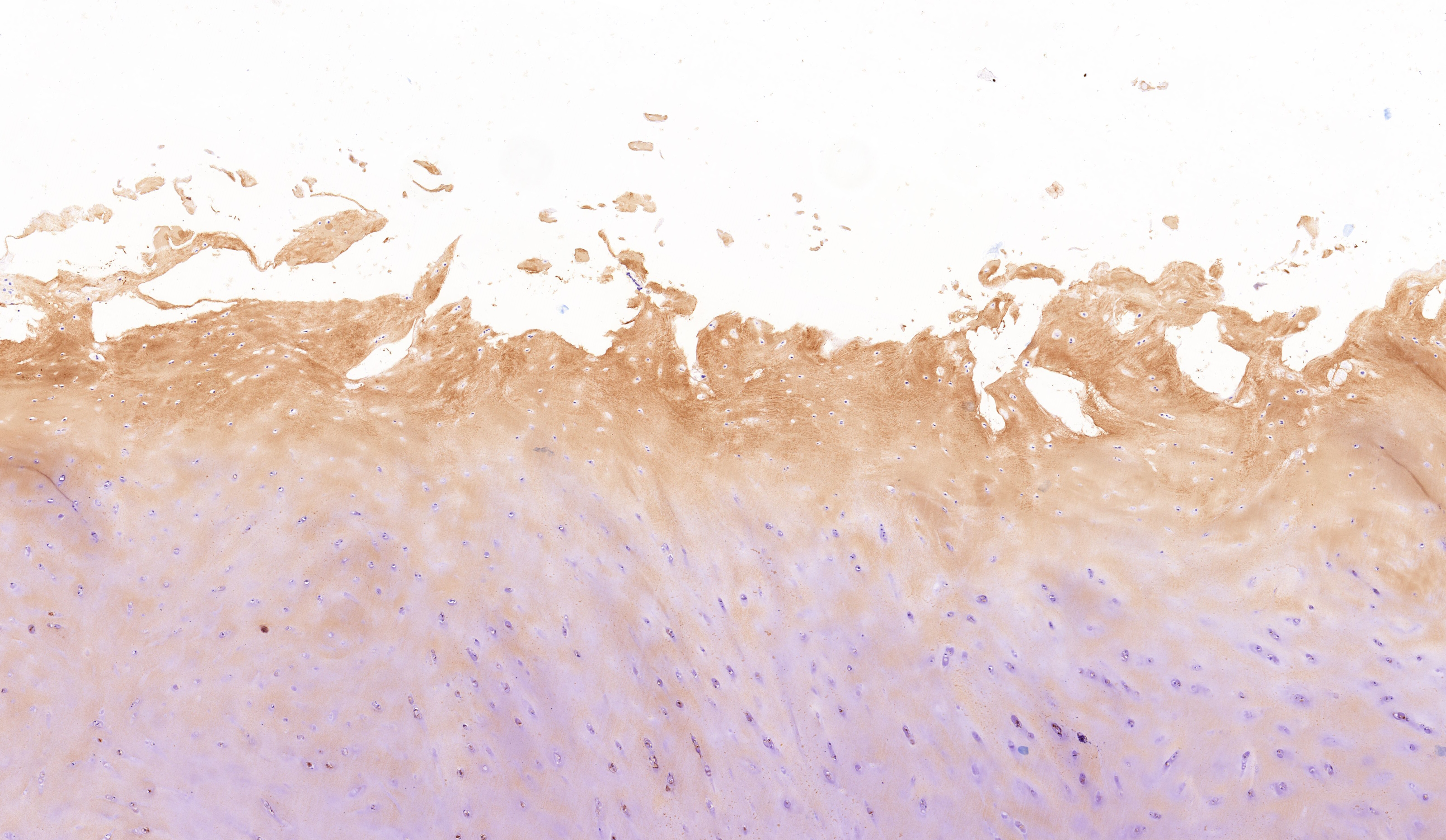

Supplement: Supplementary file 6 — Source data Fig. 2 [file 44321_2025_268_MOESM6_ESM.zip › Figure 2/2J/COLII North MC.tif]

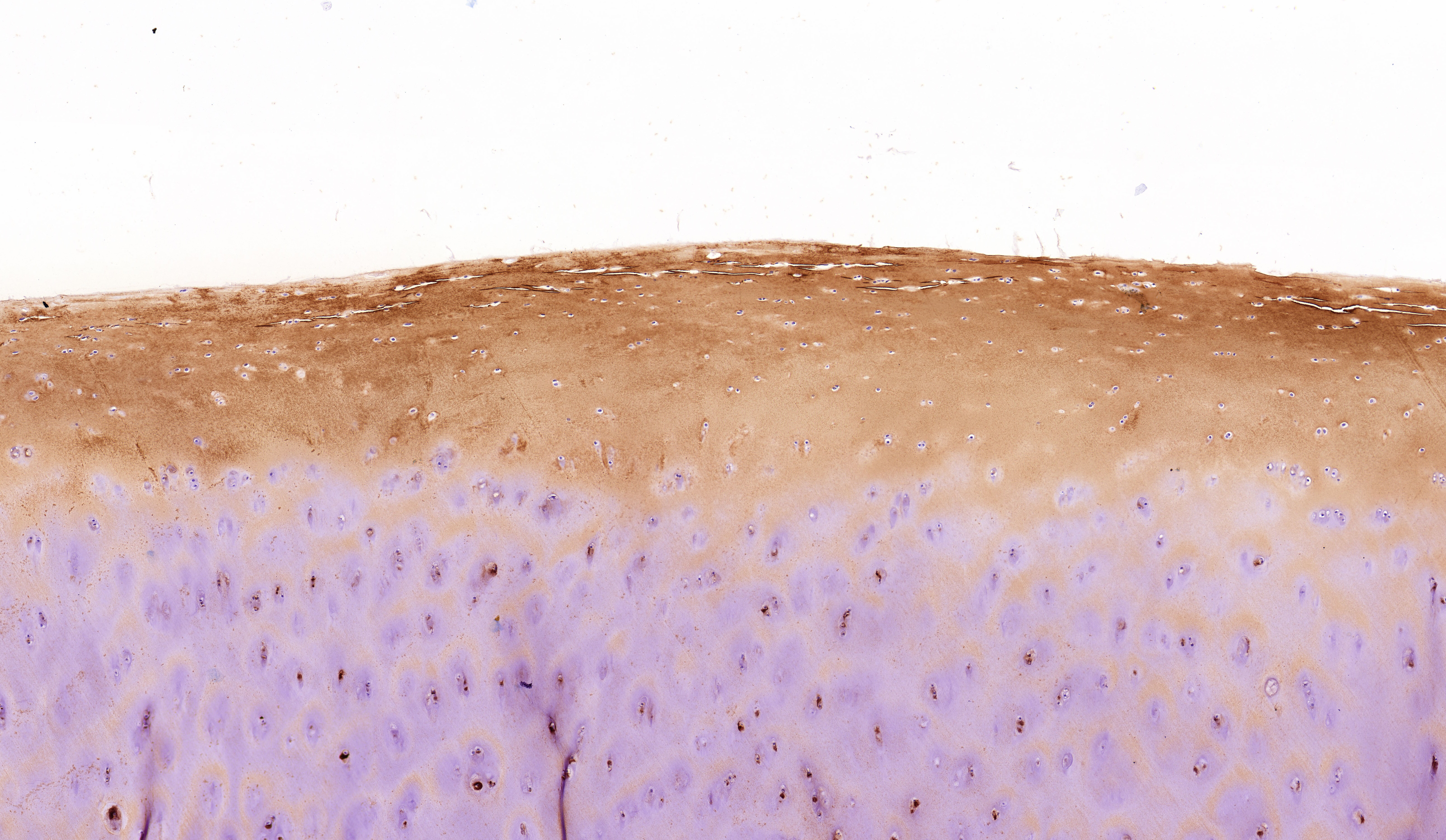

Supplement: Supplementary file 6 — Source data Fig. 2 [file 44321_2025_268_MOESM6_ESM.zip › Figure 2/2J/COLII South LC.tif]

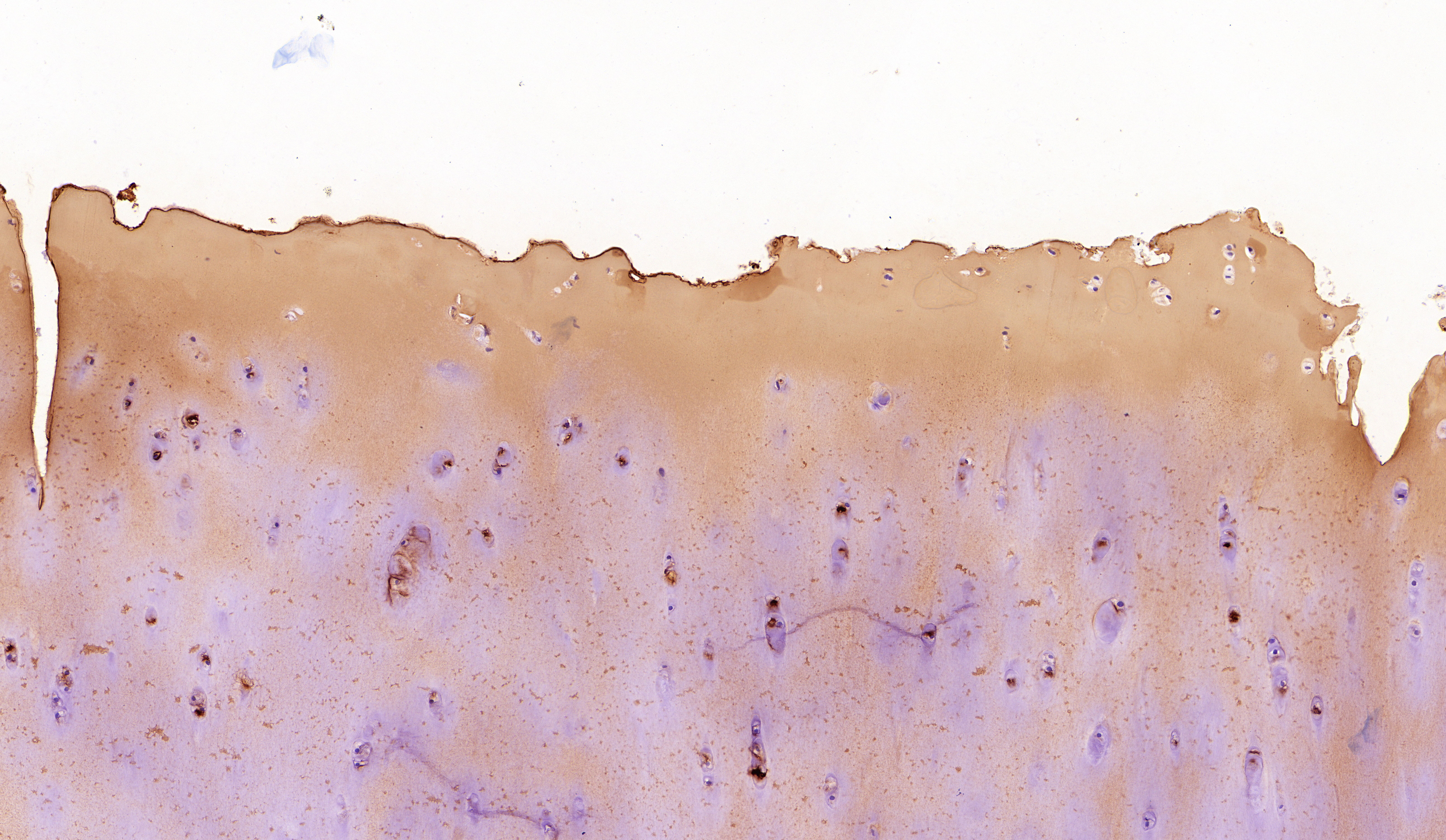

Supplement: Supplementary file 6 — Source data Fig. 2 [file 44321_2025_268_MOESM6_ESM.zip › Figure 2/2J/COLII South MC.tif]

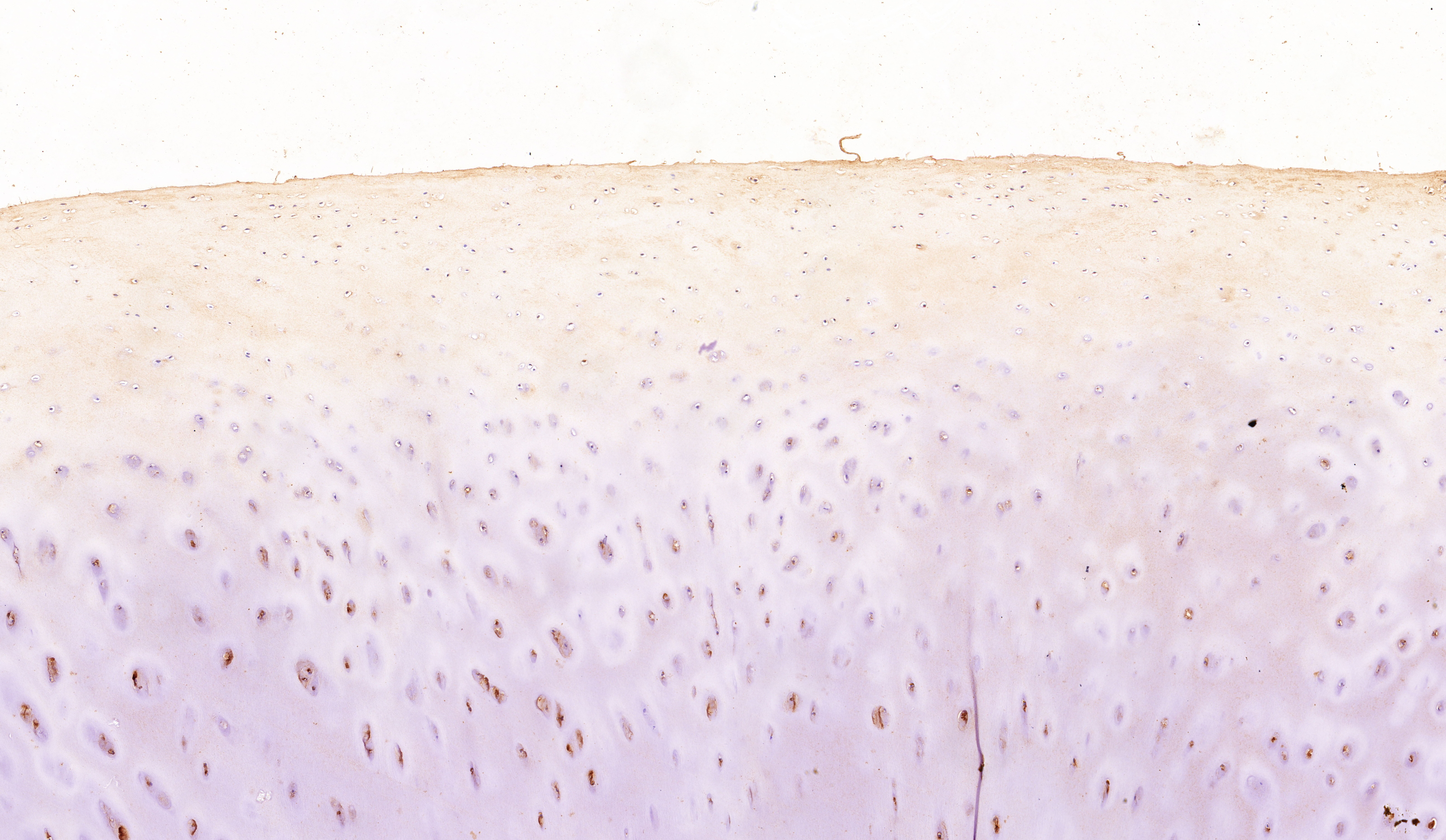

Supplement: Supplementary file 6 — Source data Fig. 2 [file 44321_2025_268_MOESM6_ESM.zip › Figure 2/2J/MMP13 North LC.tif]

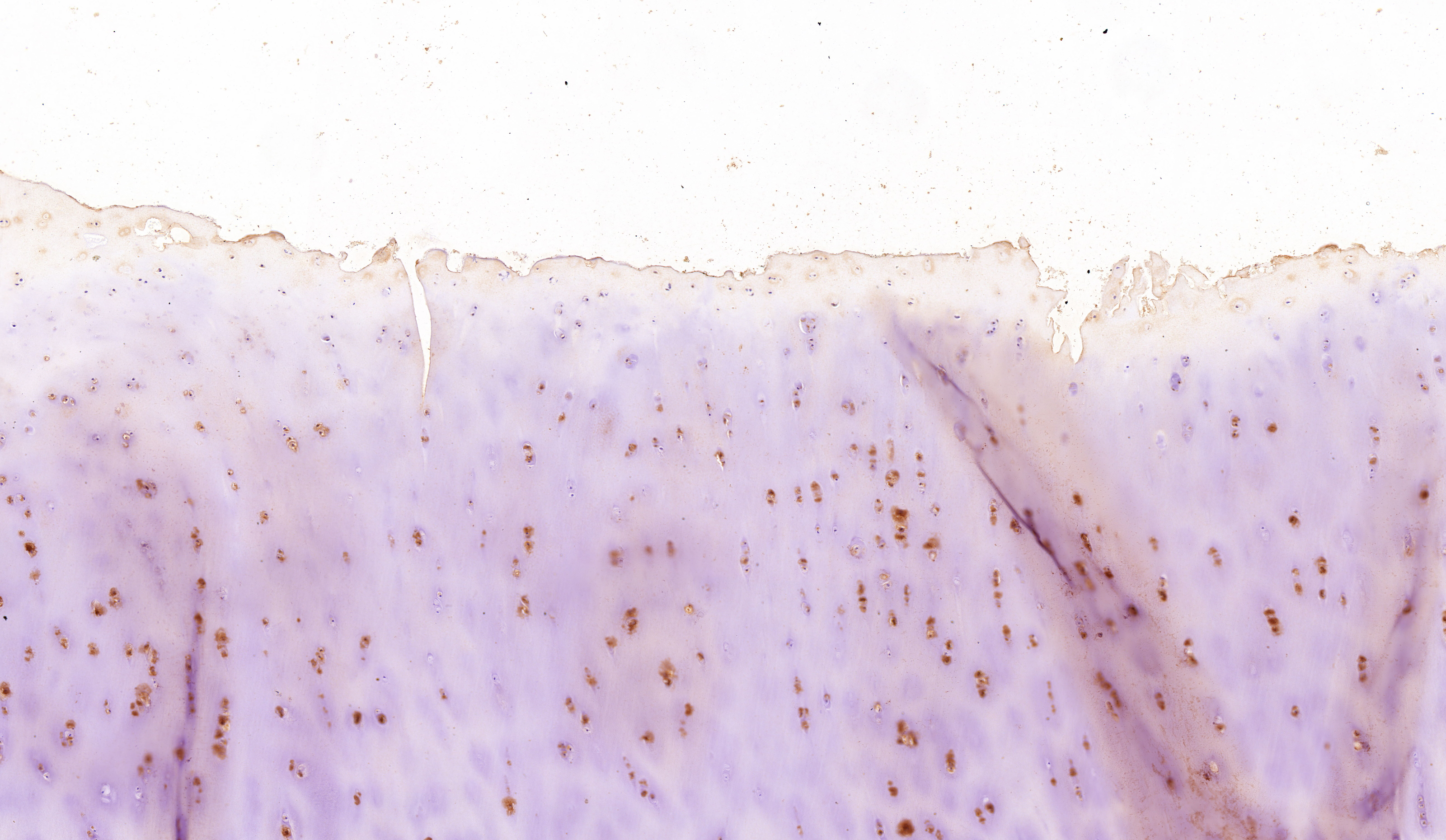

Supplement: Supplementary file 6 — Source data Fig. 2 [file 44321_2025_268_MOESM6_ESM.zip › Figure 2/2J/MMP13 North MC.tif]

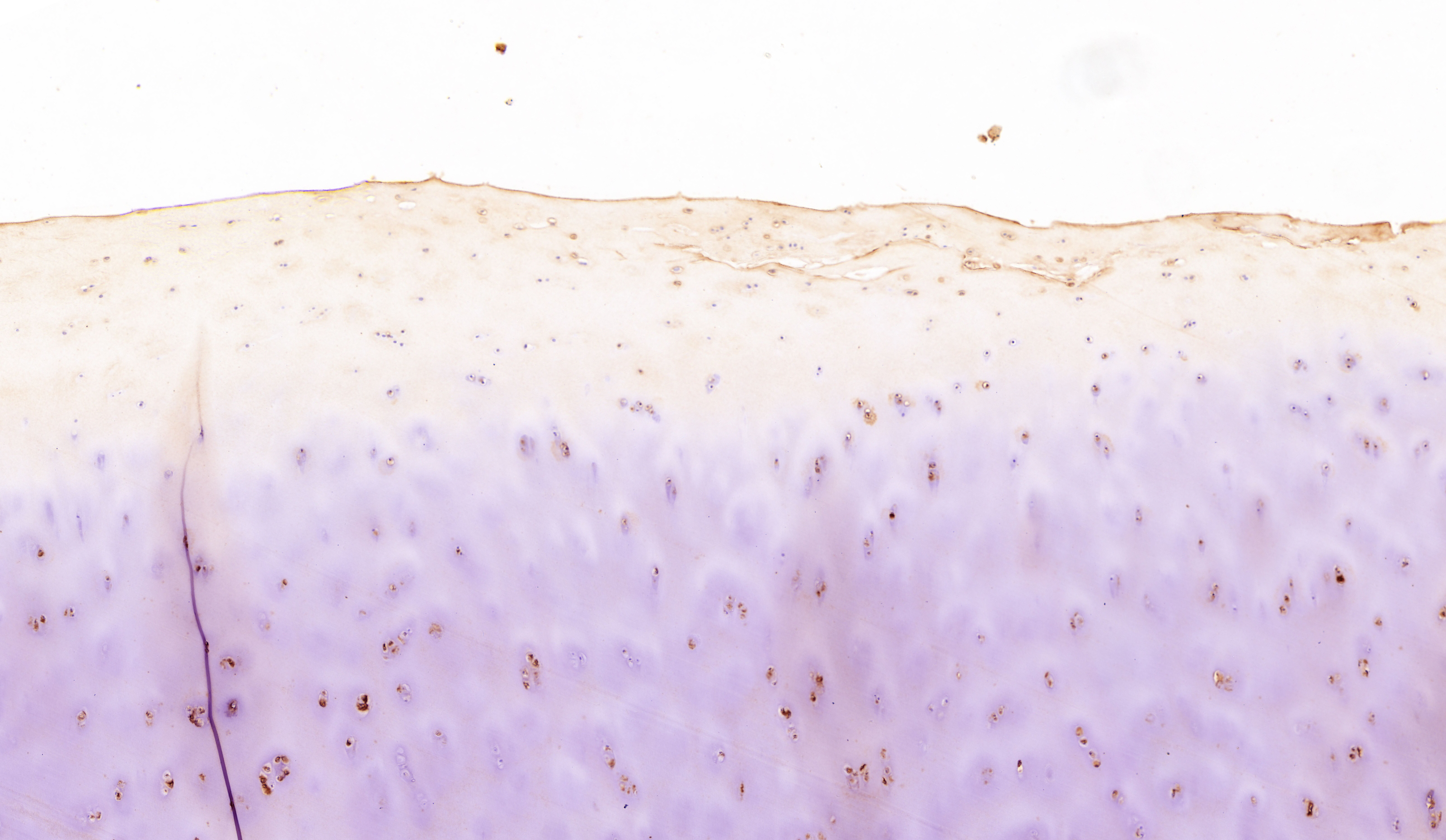

Supplement: Supplementary file 6 — Source data Fig. 2 [file 44321_2025_268_MOESM6_ESM.zip › Figure 2/2J/MMP13 South LC.tif]

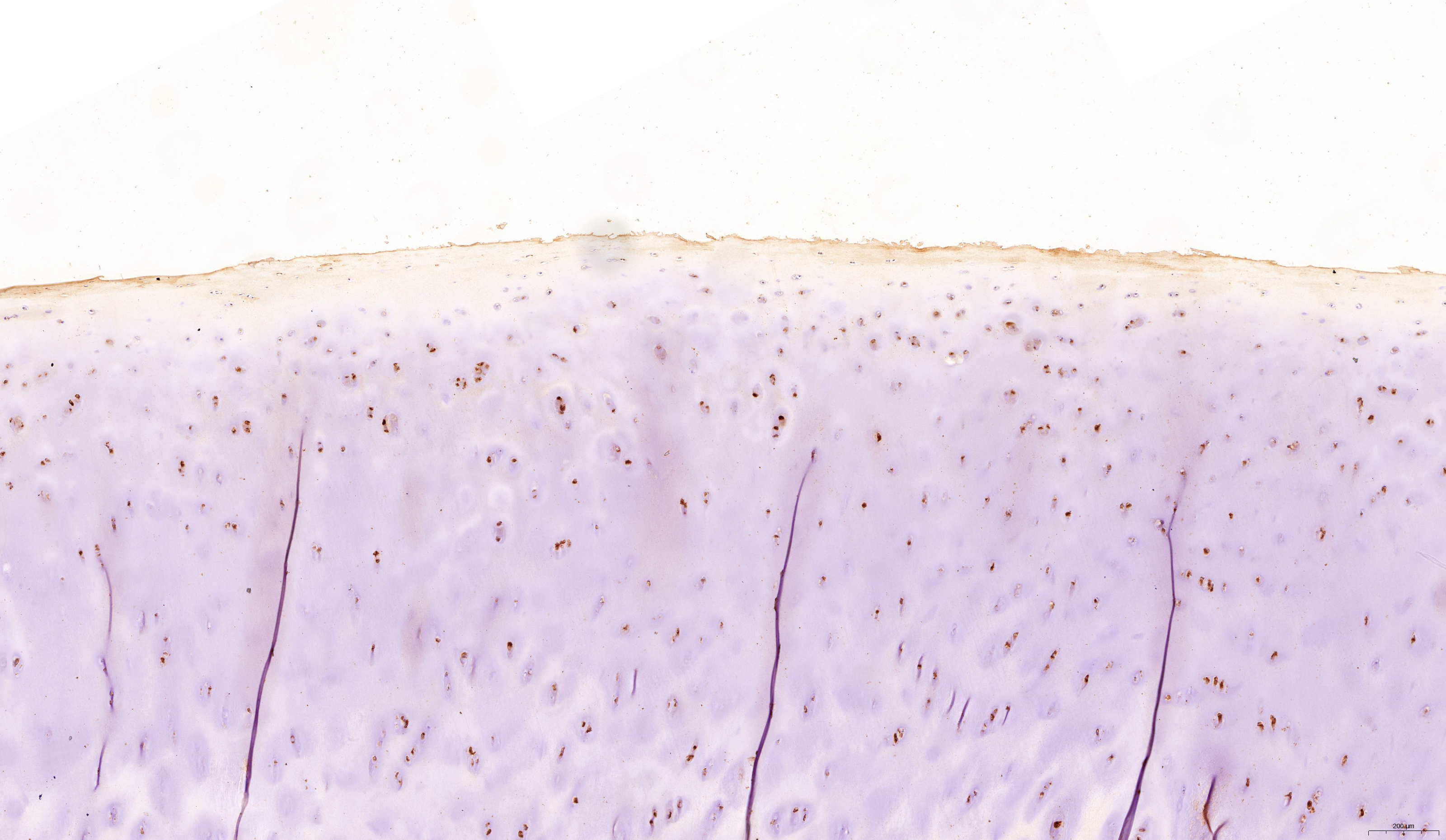

Supplement: Supplementary file 6 — Source data Fig. 2 [file 44321_2025_268_MOESM6_ESM.zip › Figure 2/2J/MMP13 South MC.tif]

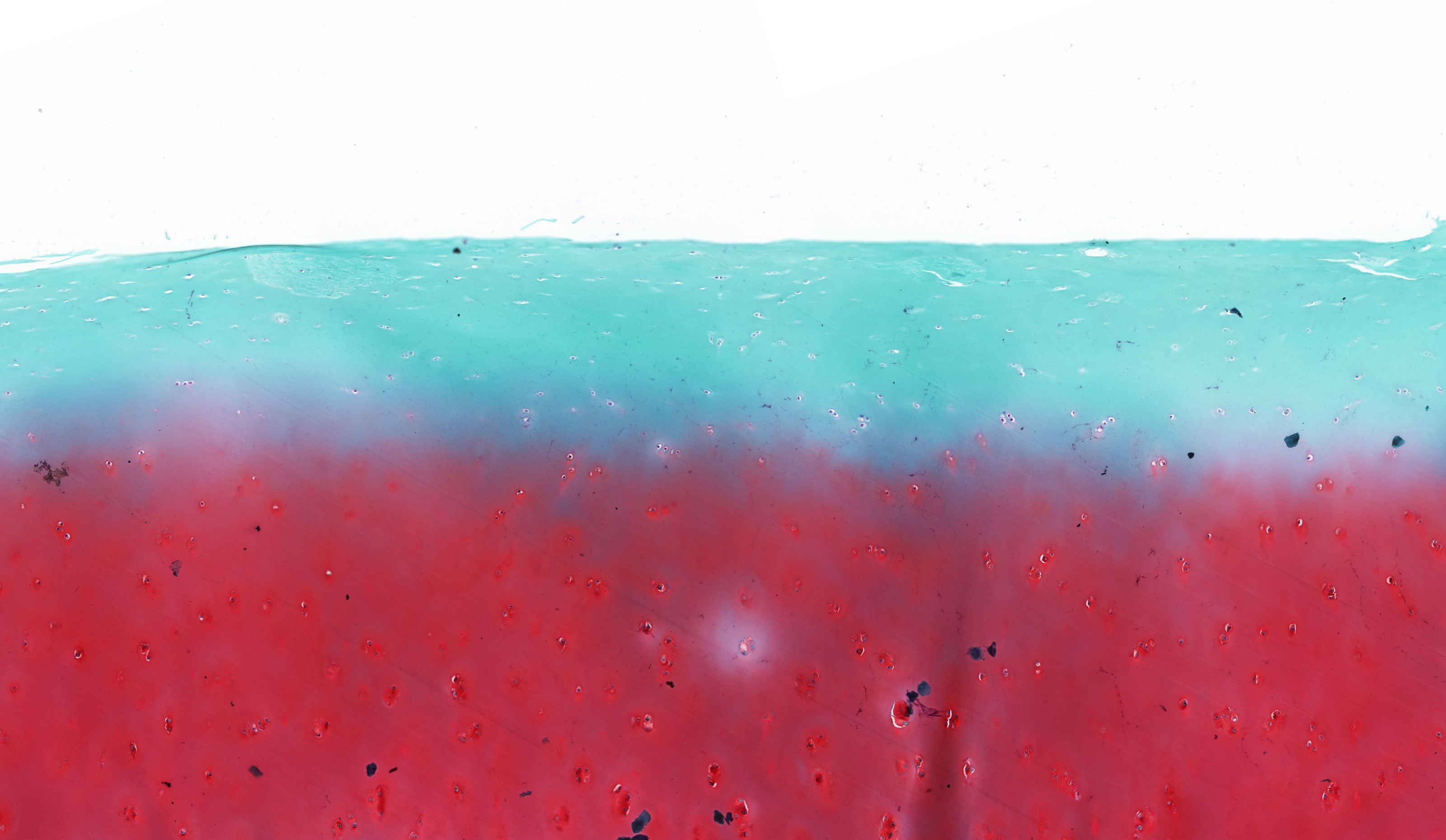

Supplement: Supplementary file 6 — Source data Fig. 2 [file 44321_2025_268_MOESM6_ESM.zip › Figure 2/2J/SO North LC.tif]

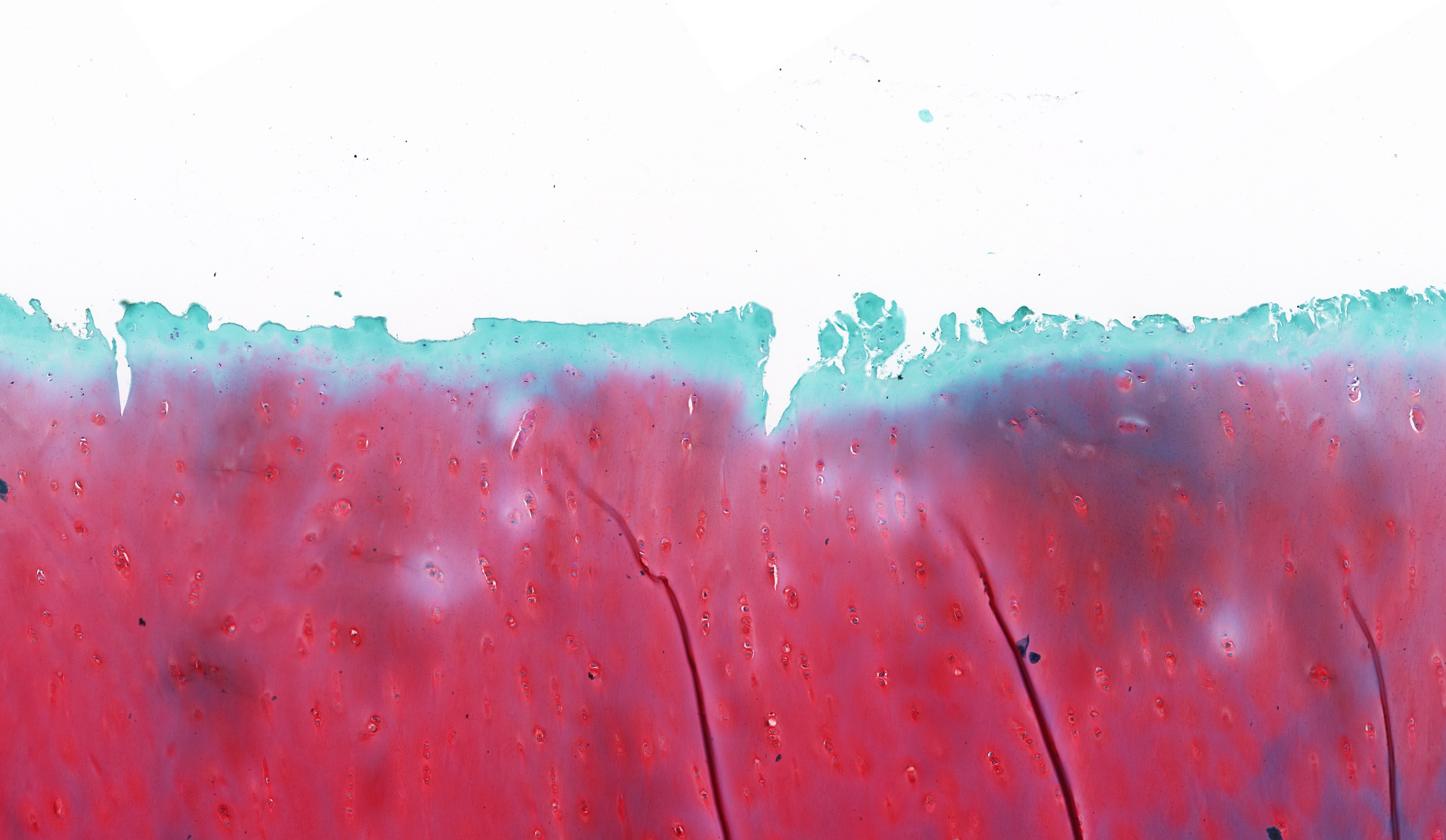

Supplement: Supplementary file 6 — Source data Fig. 2 [file 44321_2025_268_MOESM6_ESM.zip › Figure 2/2J/SO North MC.tif]

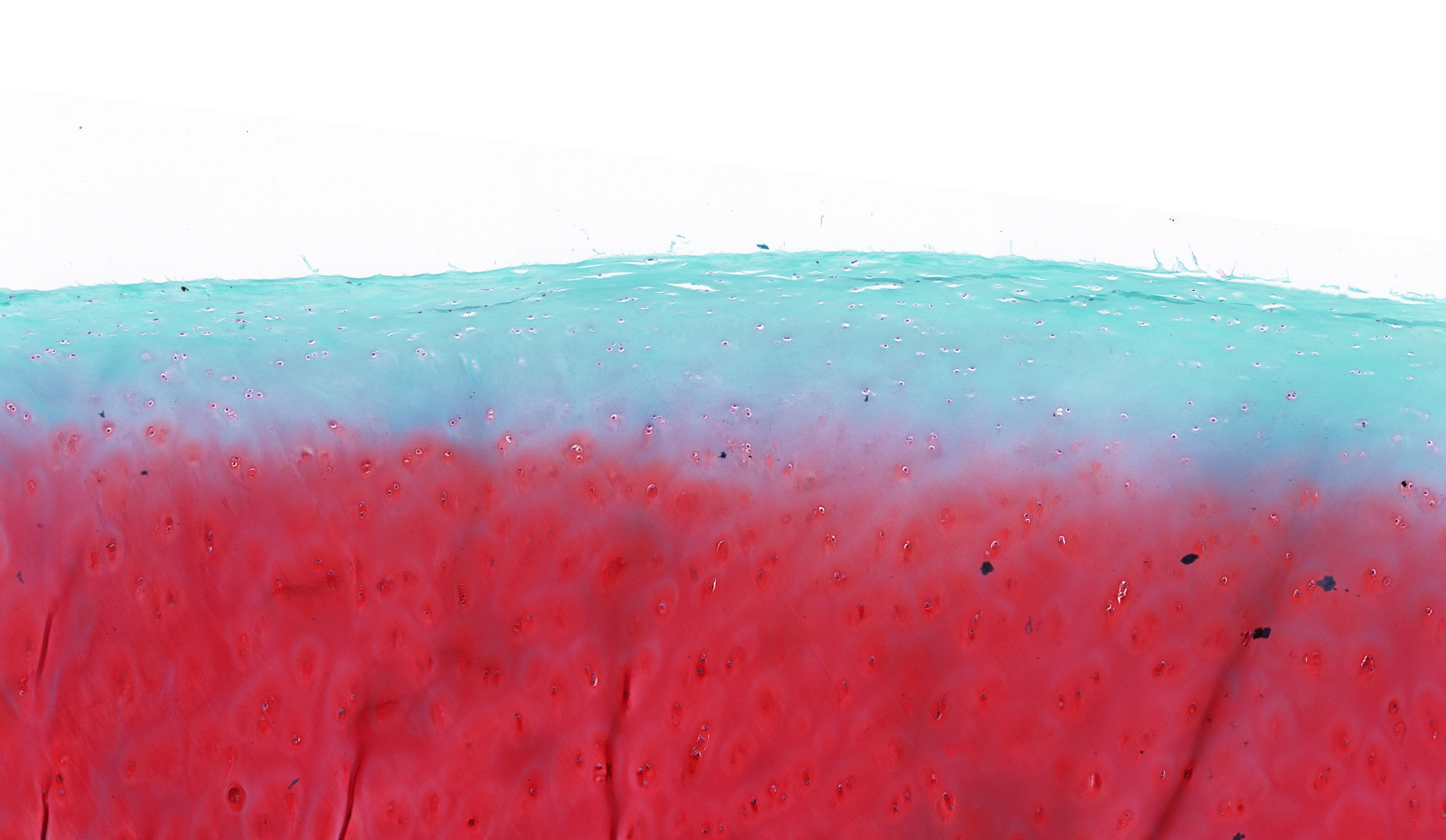

Supplement: Supplementary file 6 — Source data Fig. 2 [file 44321_2025_268_MOESM6_ESM.zip › Figure 2/2J/SO South LC.tif]

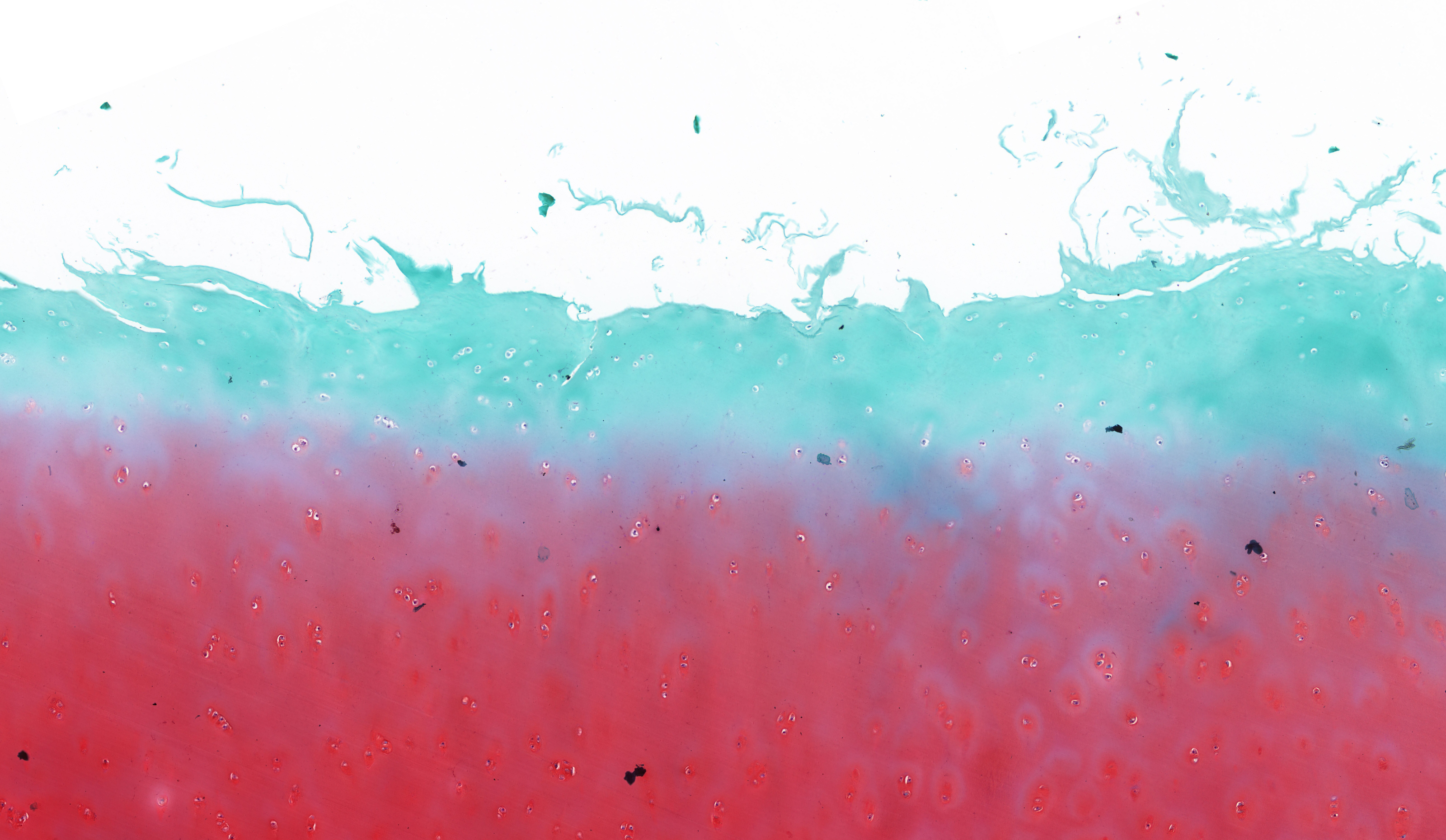

Supplement: Supplementary file 6 — Source data Fig. 2 [file 44321_2025_268_MOESM6_ESM.zip › Figure 2/2J/SO south MC.tif]

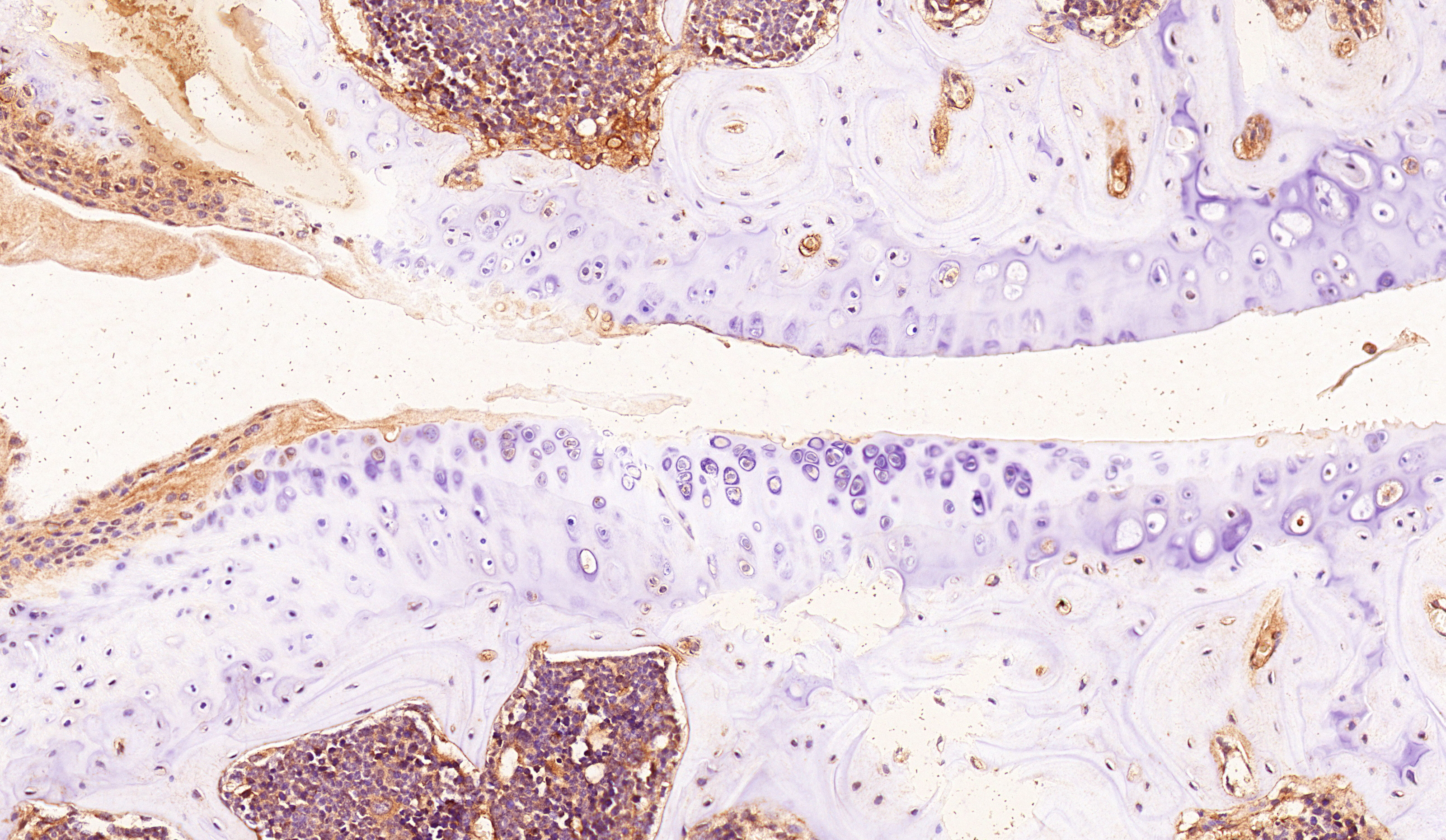

Supplement: Supplementary file 7 — Source data Fig. 3 [file 44321_2025_268_MOESM7_ESM.zip › Figure 3/3C/Apoe cko.tif]

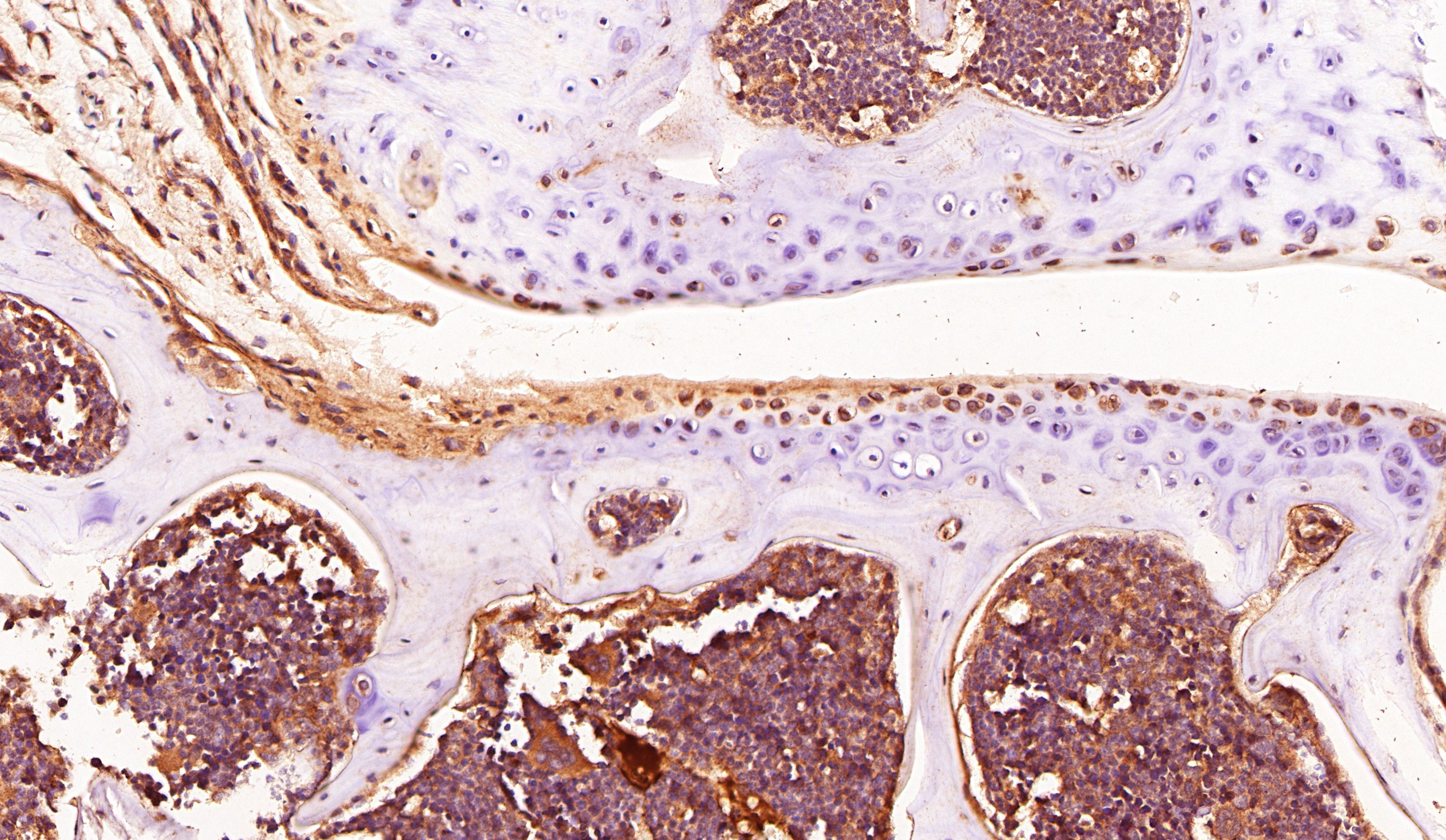

Supplement: Supplementary file 7 — Source data Fig. 3 [file 44321_2025_268_MOESM7_ESM.zip › Figure 3/3C/Apoe flox.tif]

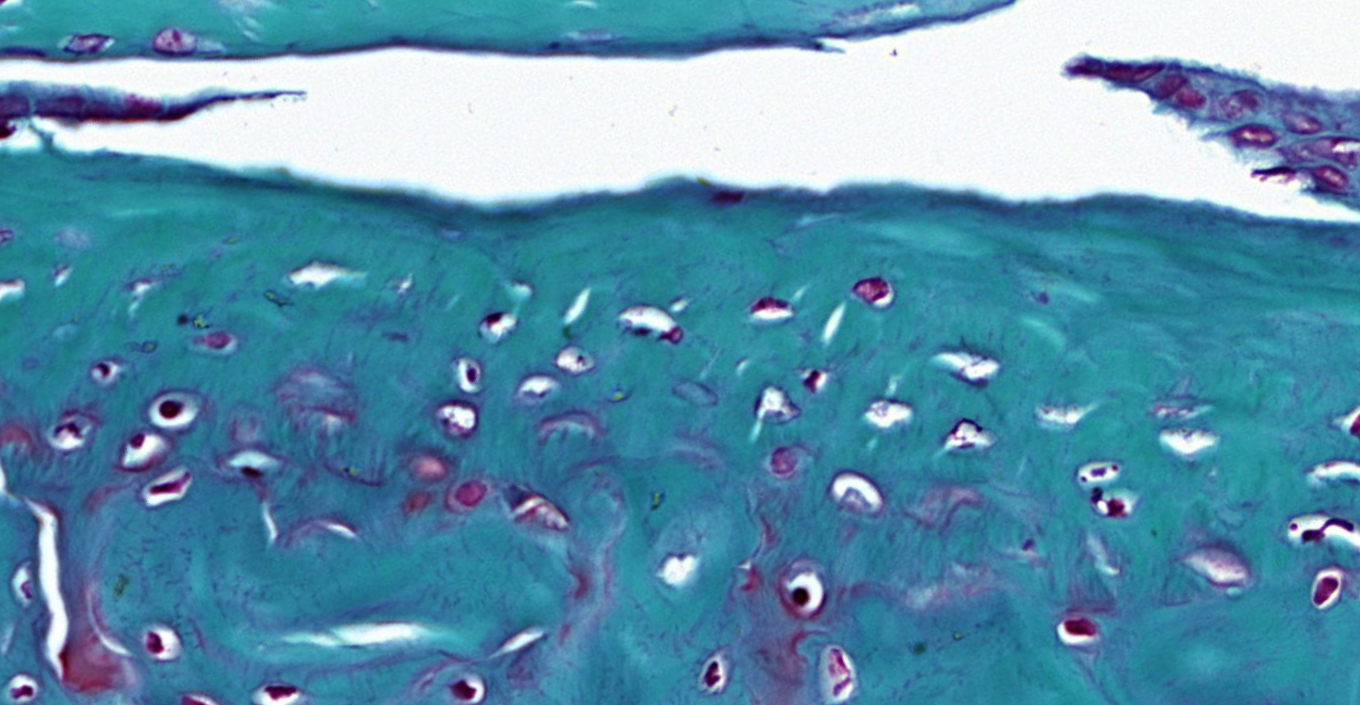

Supplement: Supplementary file 7 — Source data Fig. 3 [file 44321_2025_268_MOESM7_ESM.zip › Figure 3/3D/Apoe cko LTDMM.tif]

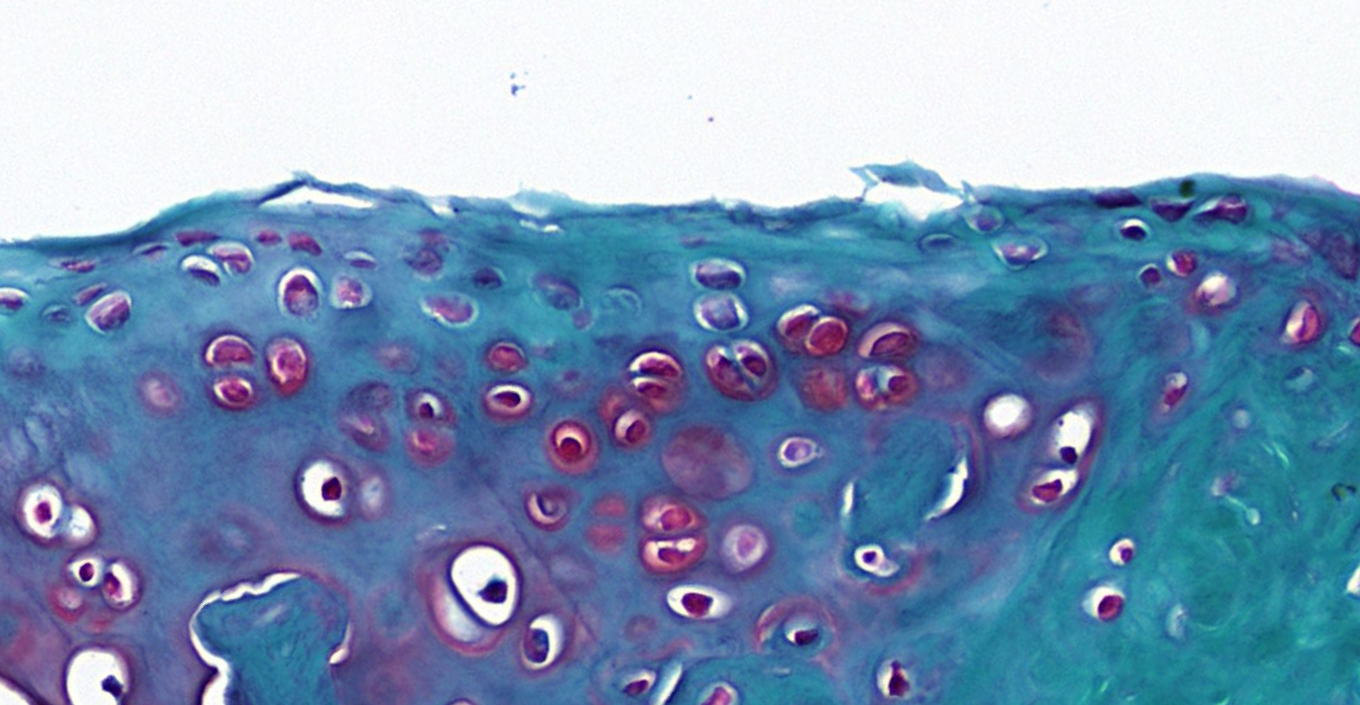

Supplement: Supplementary file 7 — Source data Fig. 3 [file 44321_2025_268_MOESM7_ESM.zip › Figure 3/3D/Apoe cko RTDMM.tif]

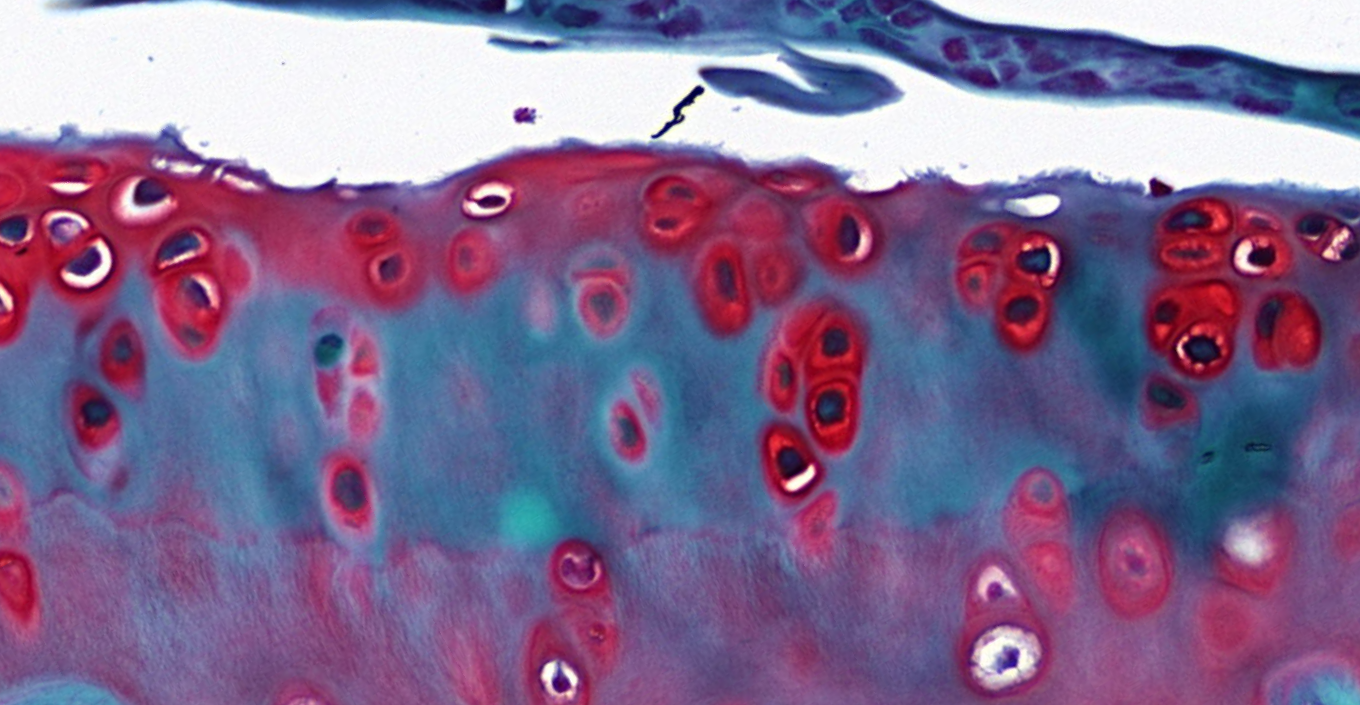

Supplement: Supplementary file 7 — Source data Fig. 3 [file 44321_2025_268_MOESM7_ESM.zip › Figure 3/3D/Apoe fl LTDMM.tif]

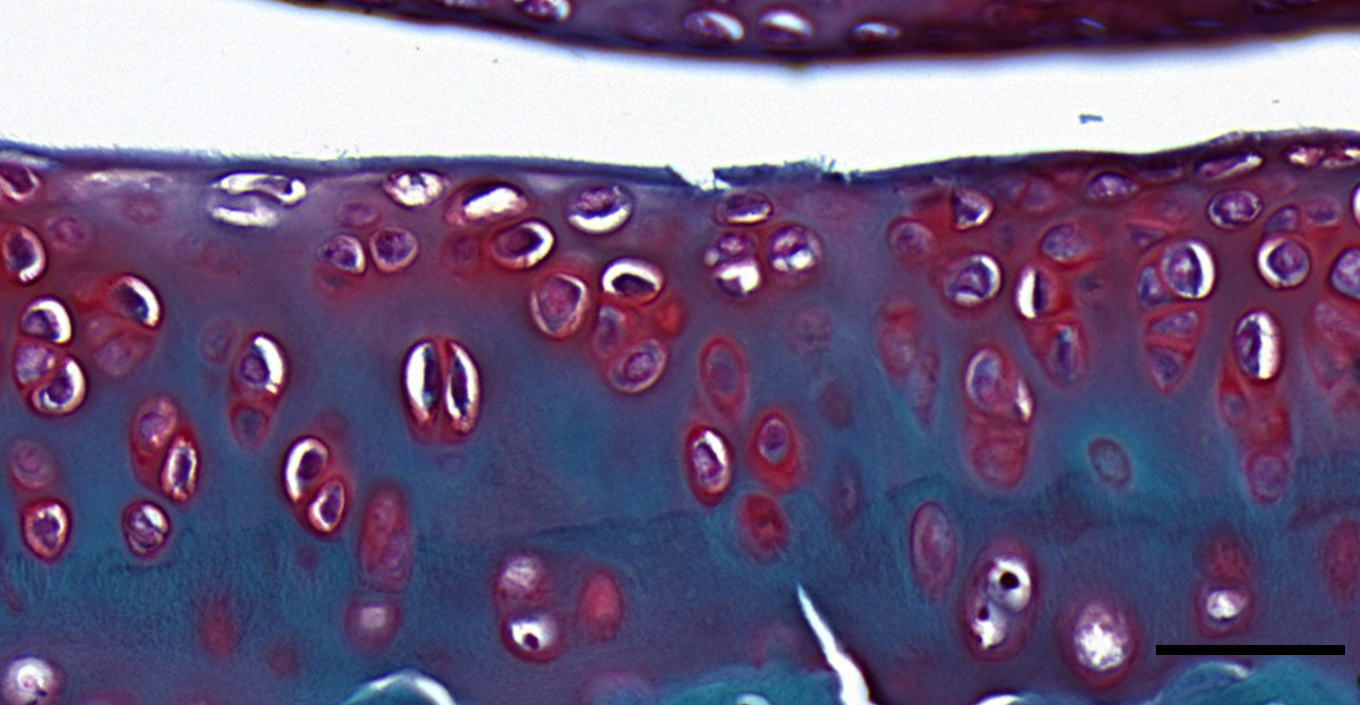

Supplement: Supplementary file 7 — Source data Fig. 3 [file 44321_2025_268_MOESM7_ESM.zip › Figure 3/3D/Apoe fl RTDMM.tif]

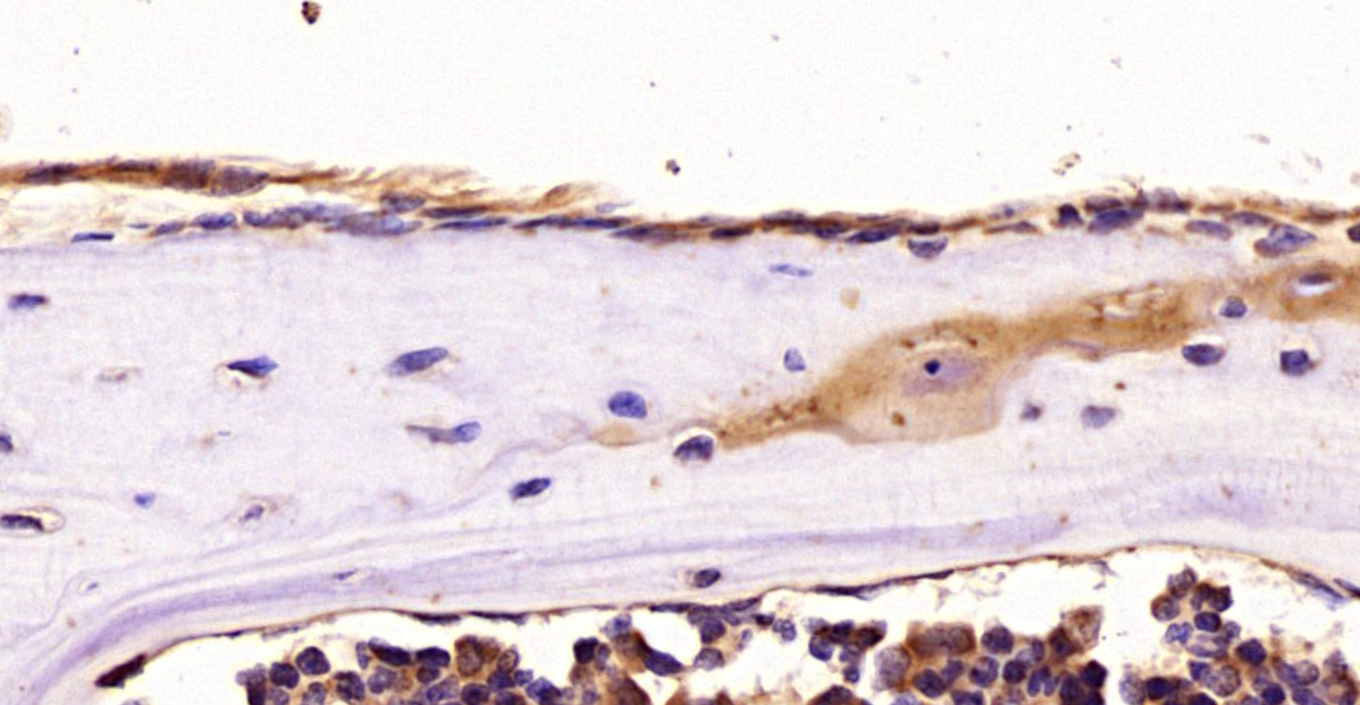

Supplement: Supplementary file 7 — Source data Fig. 3 [file 44321_2025_268_MOESM7_ESM.zip › Figure 3/3E/Apoe cko LTDMM.tif]

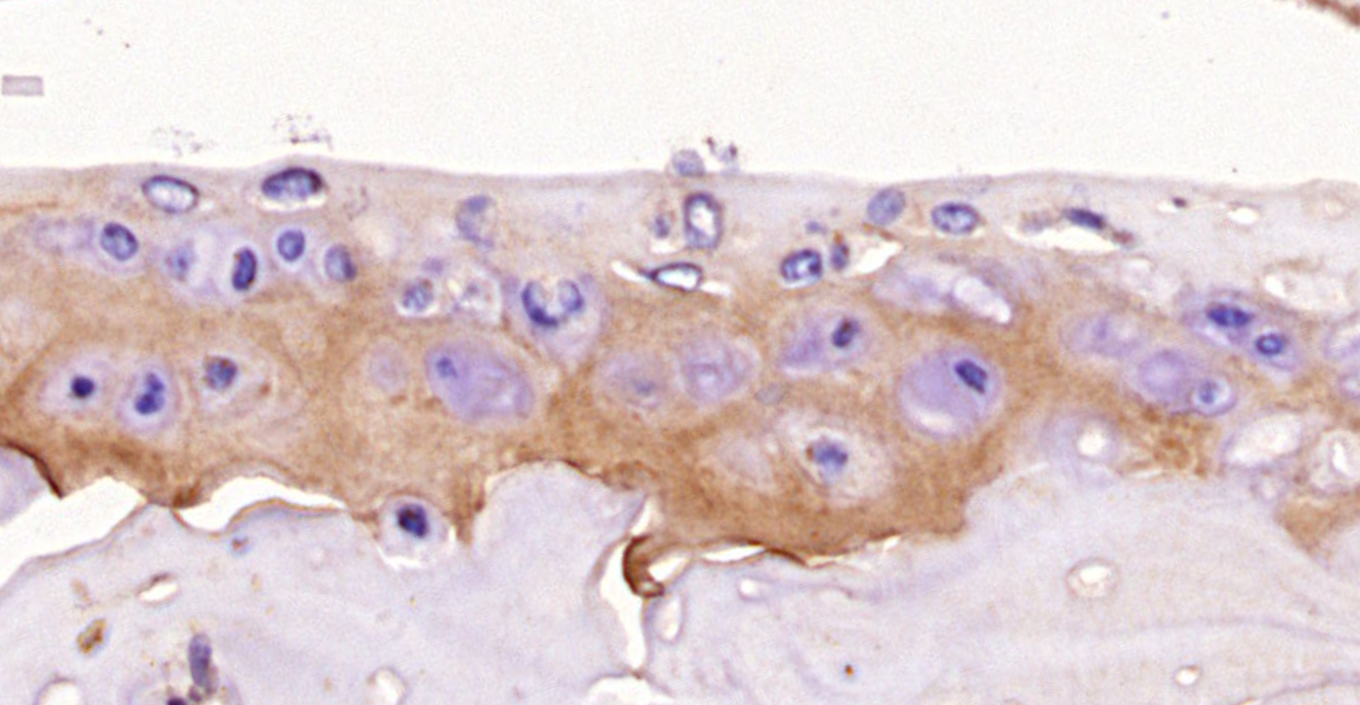

Supplement: Supplementary file 7 — Source data Fig. 3 [file 44321_2025_268_MOESM7_ESM.zip › Figure 3/3E/Apoe cko RTDMM.tif]

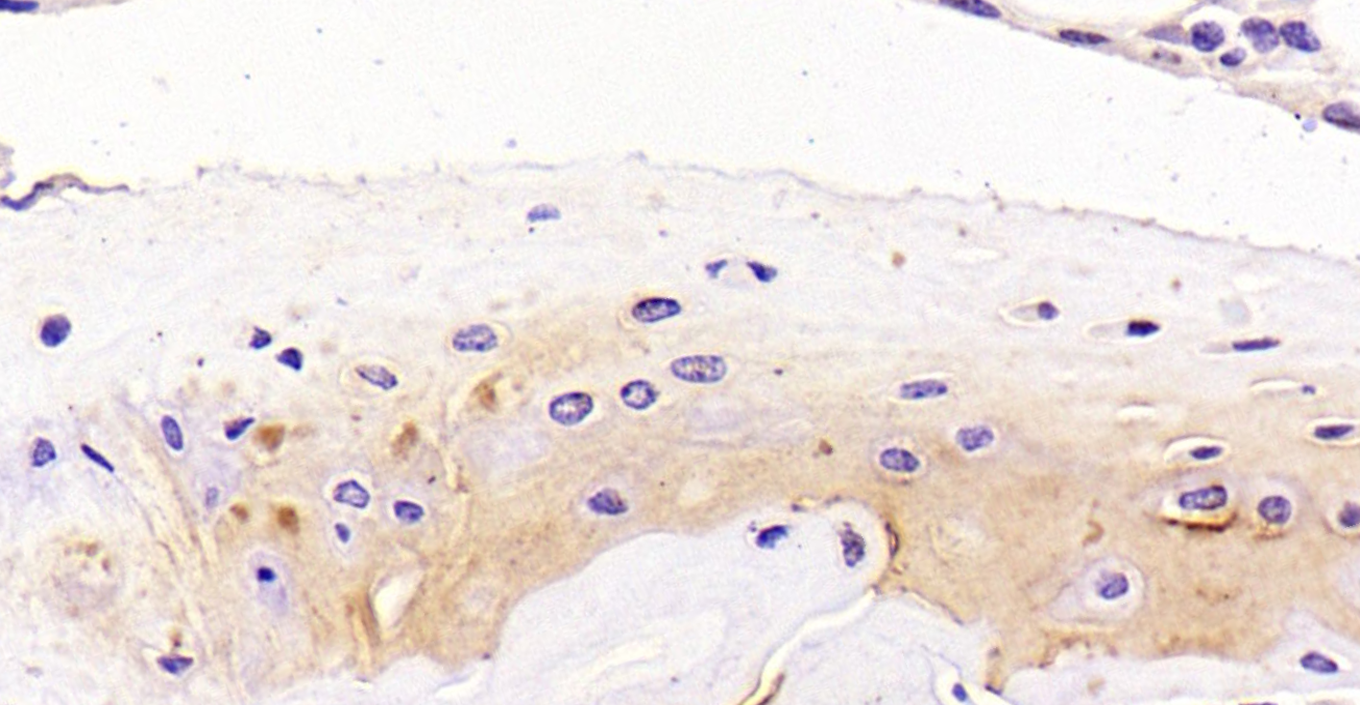

Supplement: Supplementary file 7 — Source data Fig. 3 [file 44321_2025_268_MOESM7_ESM.zip › Figure 3/3E/Apoe fl LTDMM.tif]

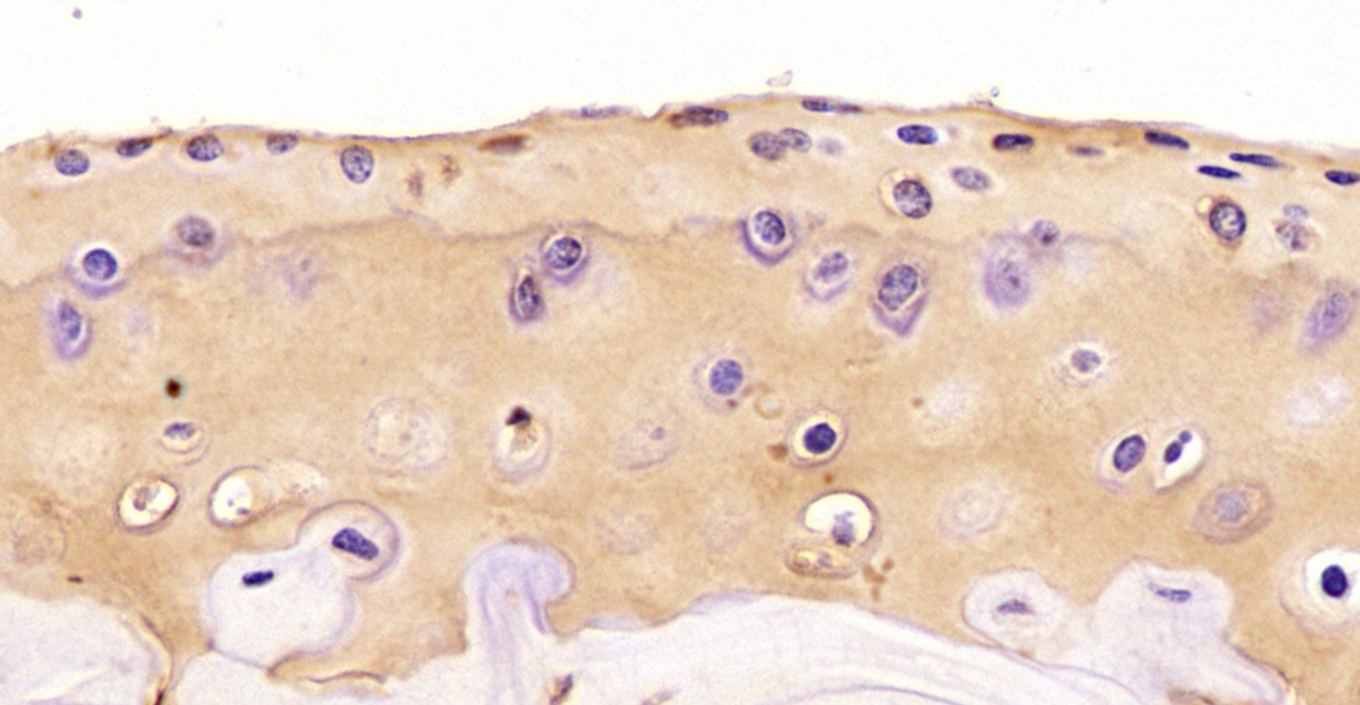

Supplement: Supplementary file 7 — Source data Fig. 3 [file 44321_2025_268_MOESM7_ESM.zip › Figure 3/3E/Apoe fl RTDMM.tif]

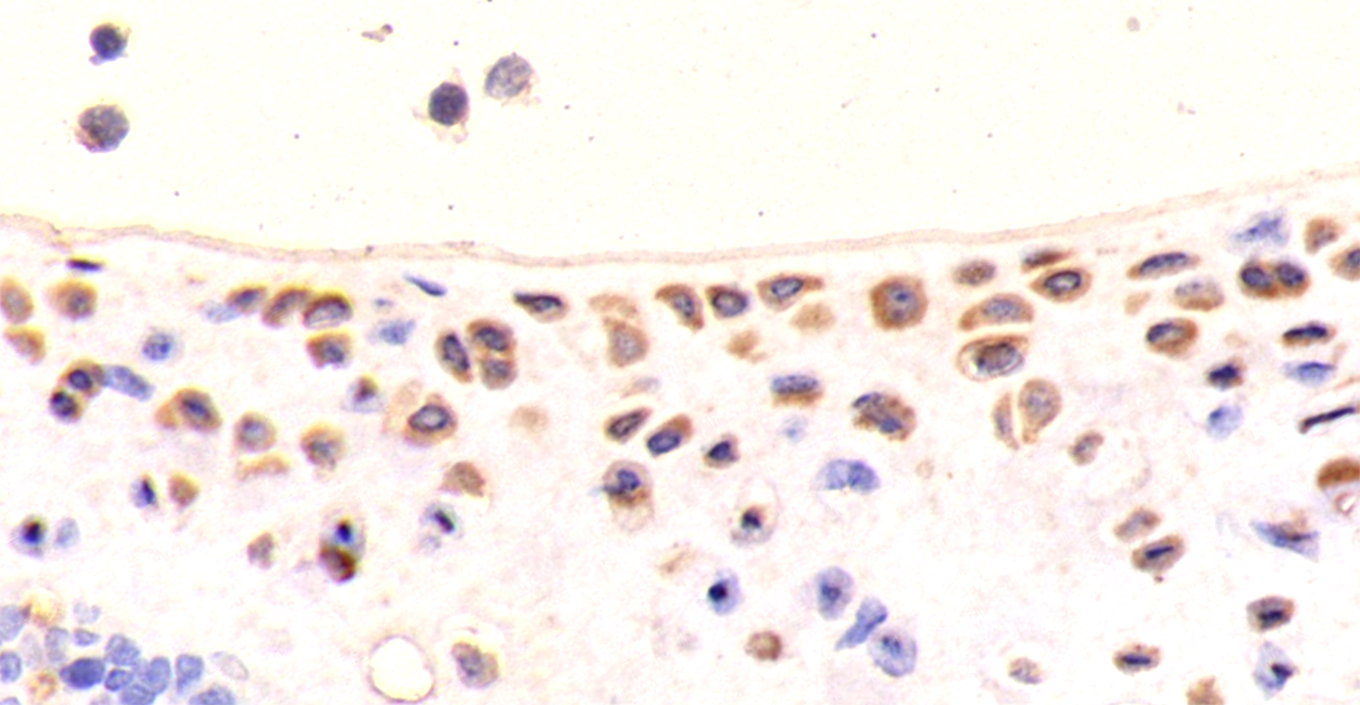

Supplement: Supplementary file 7 — Source data Fig. 3 [file 44321_2025_268_MOESM7_ESM.zip › Figure 3/3F/Apoe cko LTDMM.tif]

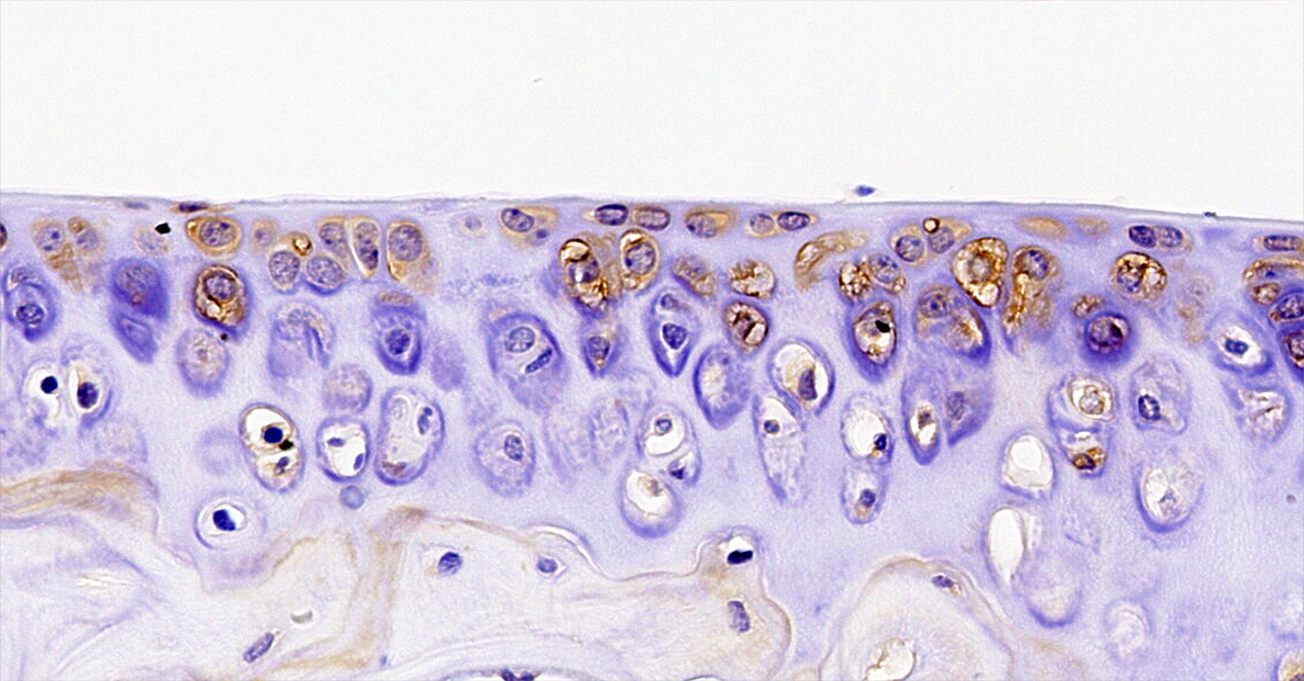

Supplement: Supplementary file 7 — Source data Fig. 3 [file 44321_2025_268_MOESM7_ESM.zip › Figure 3/3F/Apoe cko RTDMM.tif]

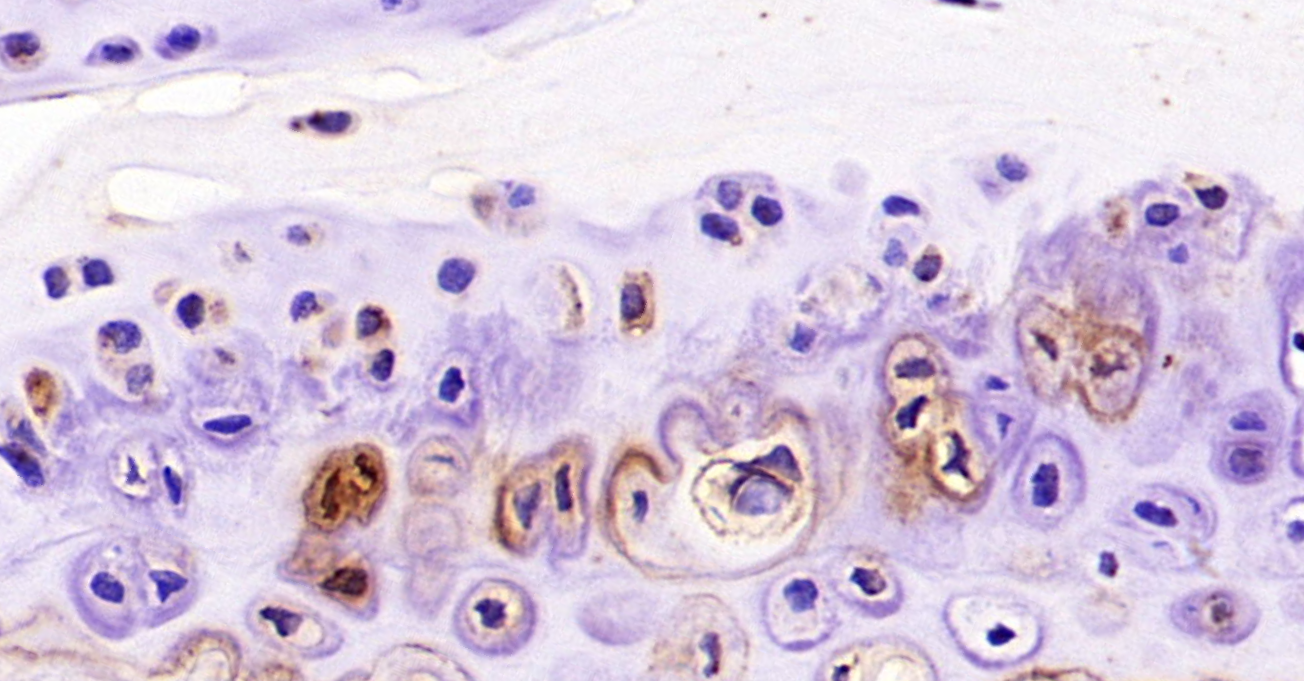

Supplement: Supplementary file 7 — Source data Fig. 3 [file 44321_2025_268_MOESM7_ESM.zip › Figure 3/3F/Apoe fl LTDMM.tif]

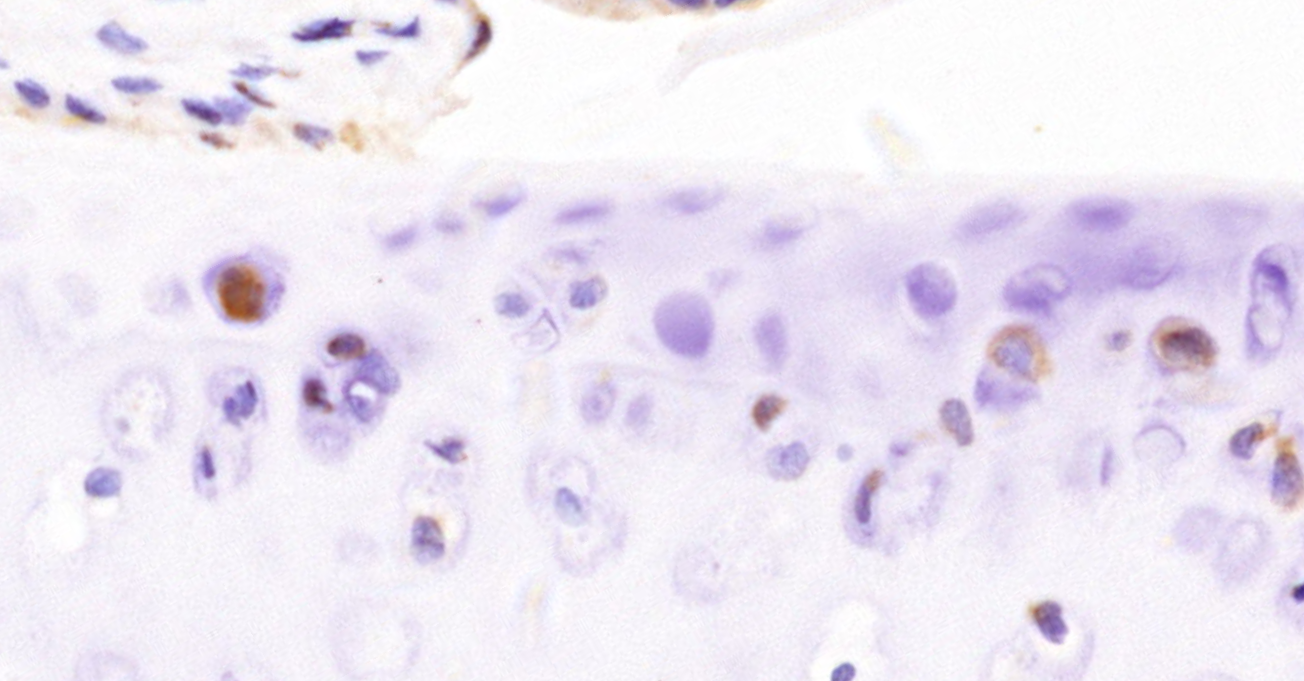

Supplement: Supplementary file 7 — Source data Fig. 3 [file 44321_2025_268_MOESM7_ESM.zip › Figure 3/3F/Apoe fl RTDMM.tif]

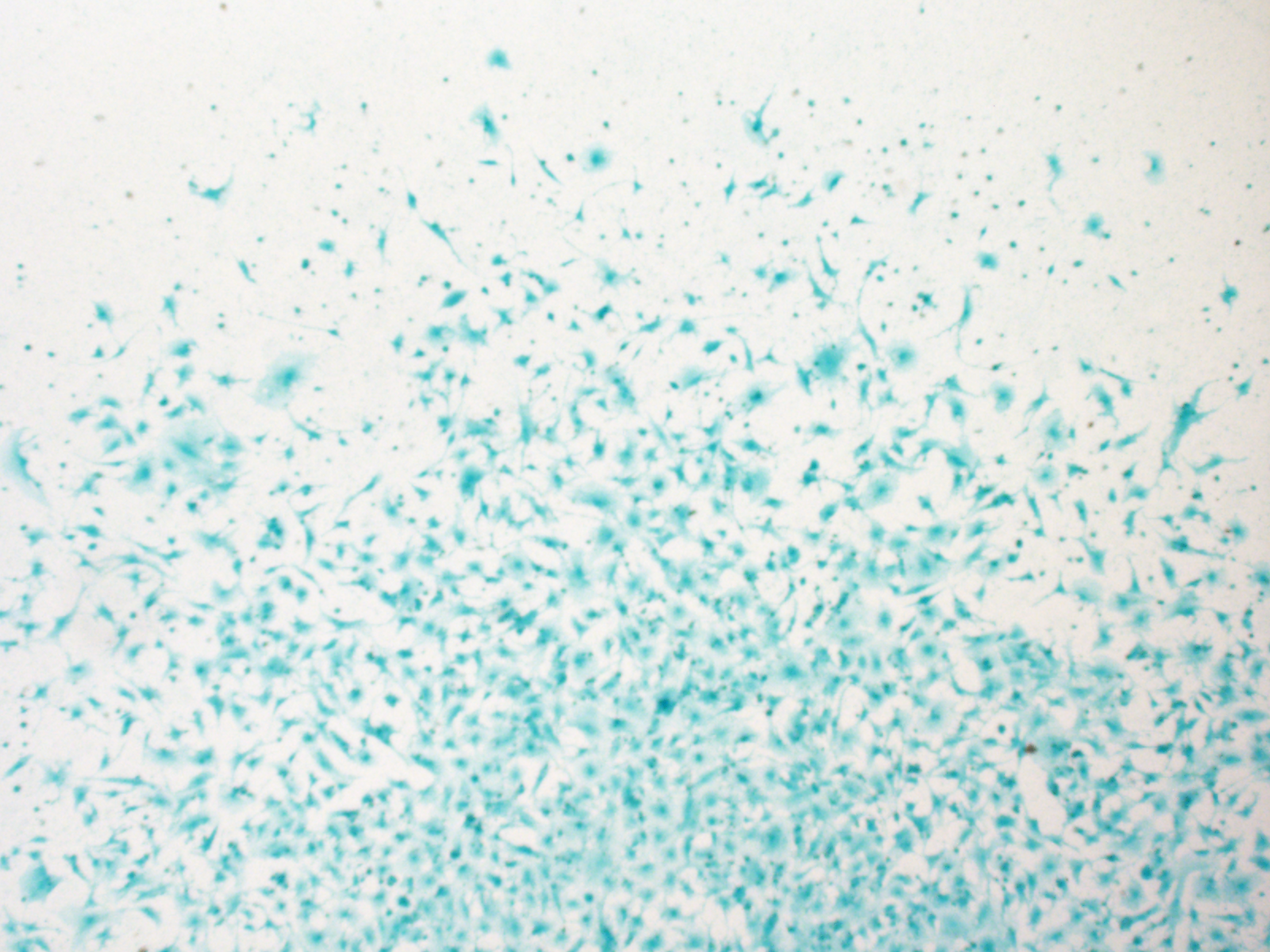

Supplement: Supplementary file 7 — Source data Fig. 3 [file 44321_2025_268_MOESM7_ESM.zip › Figure 3/3G/Apoe cko 33.tif]

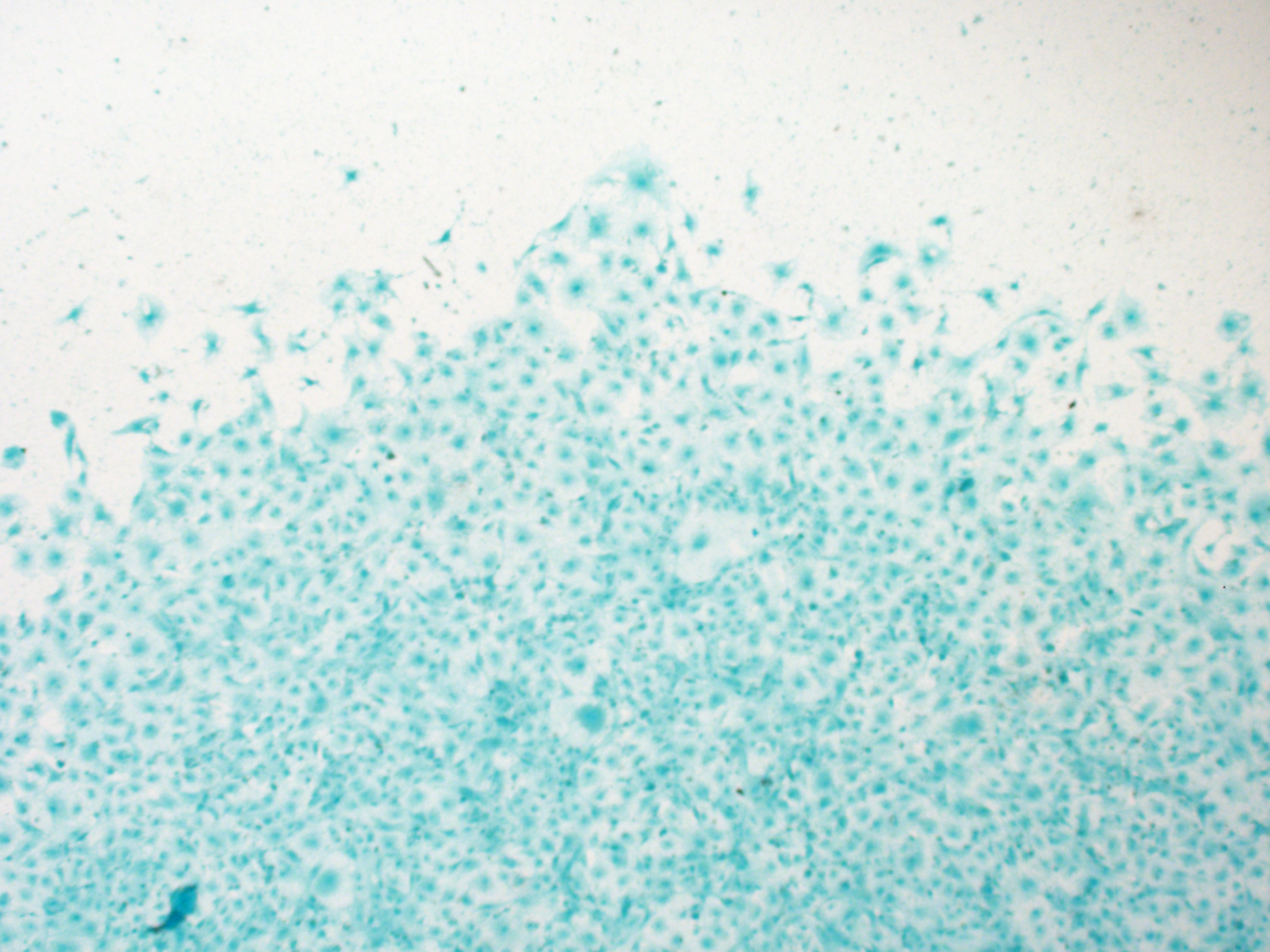

Supplement: Supplementary file 7 — Source data Fig. 3 [file 44321_2025_268_MOESM7_ESM.zip › Figure 3/3G/Apoe cko 37.tif]

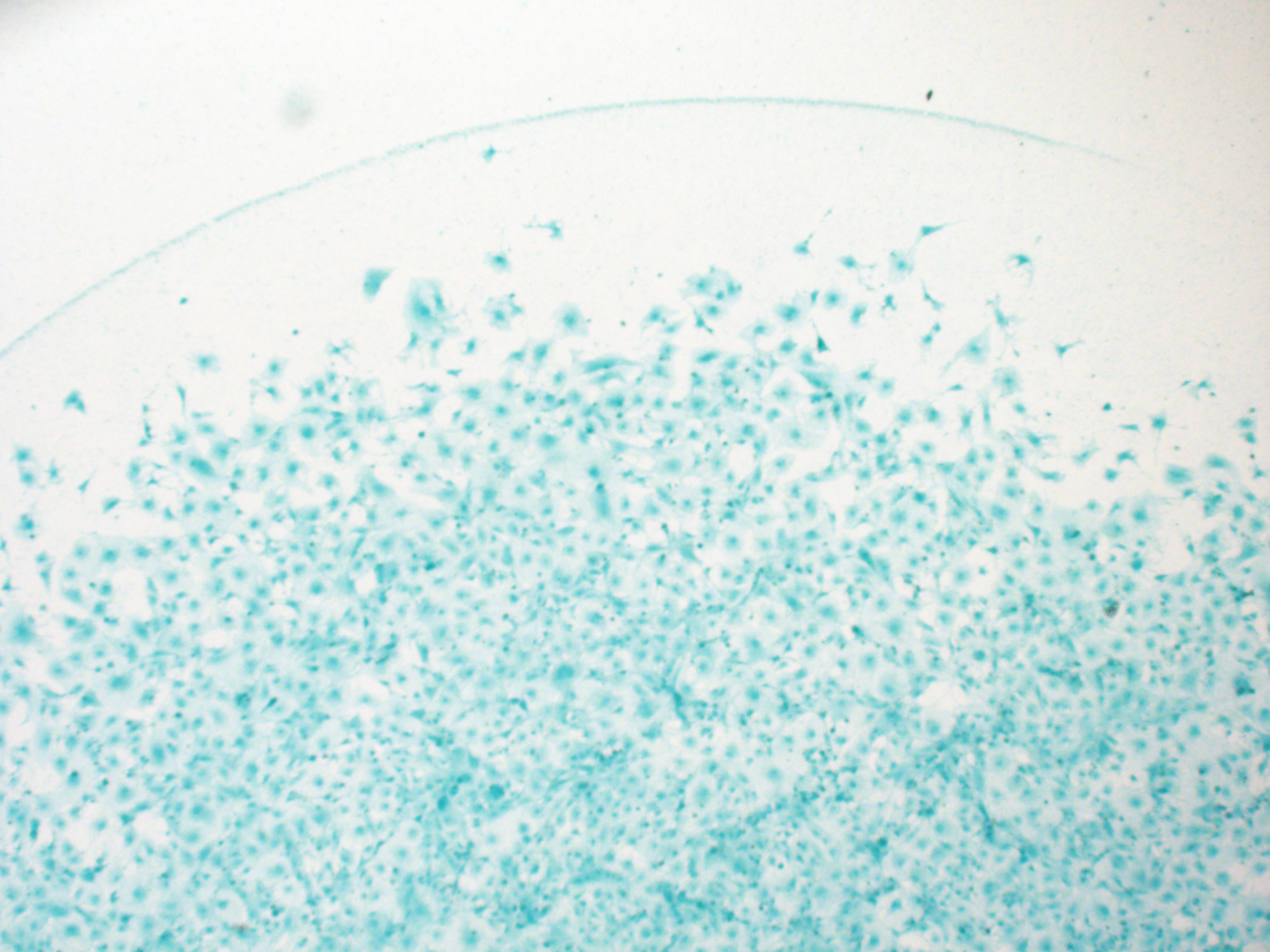

Supplement: Supplementary file 7 — Source data Fig. 3 [file 44321_2025_268_MOESM7_ESM.zip › Figure 3/3G/Apoe fl 33.tif]

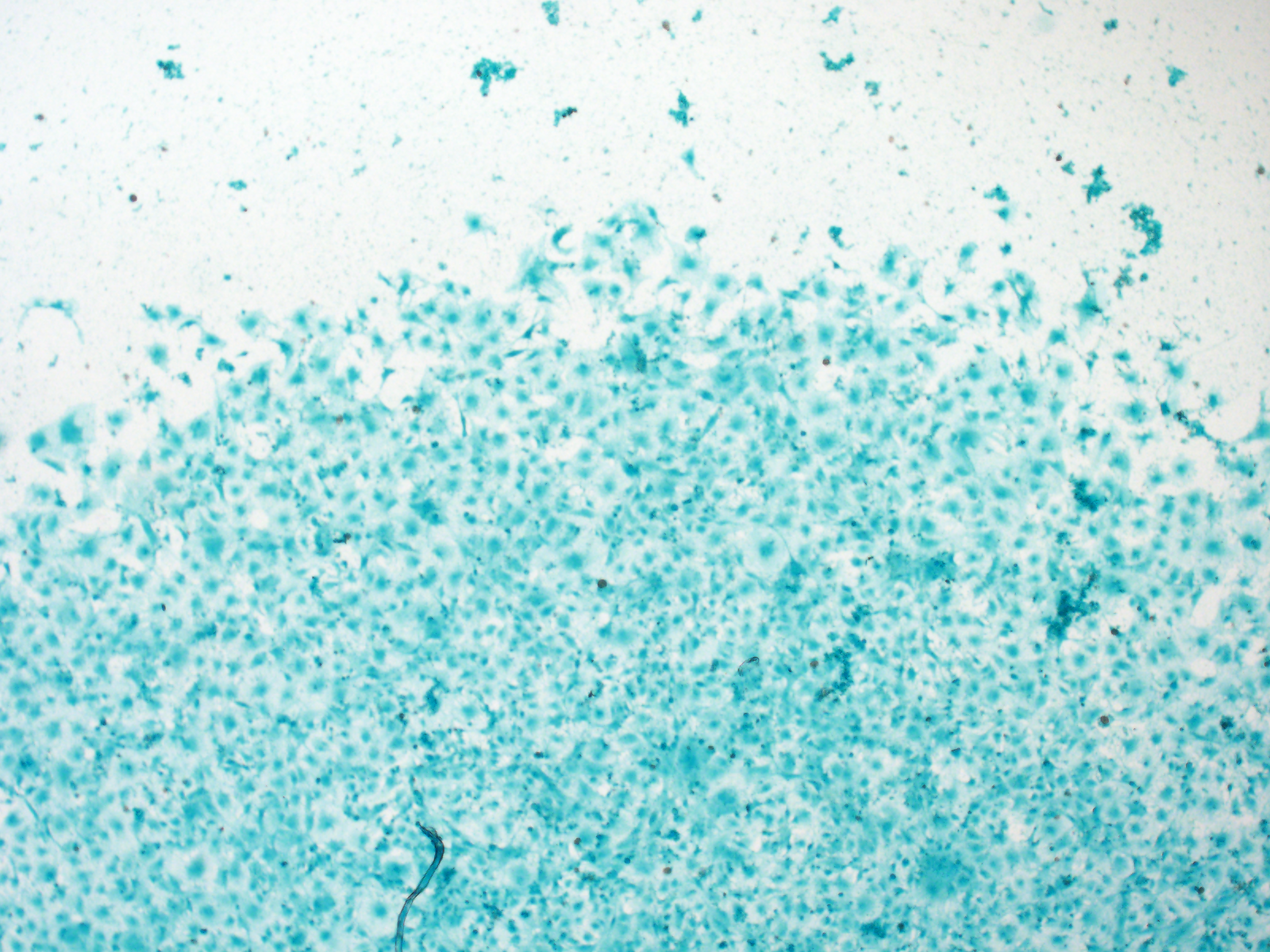

Supplement: Supplementary file 7 — Source data Fig. 3 [file 44321_2025_268_MOESM7_ESM.zip › Figure 3/3G/Apoe fl 37.tif]

Figure 3I

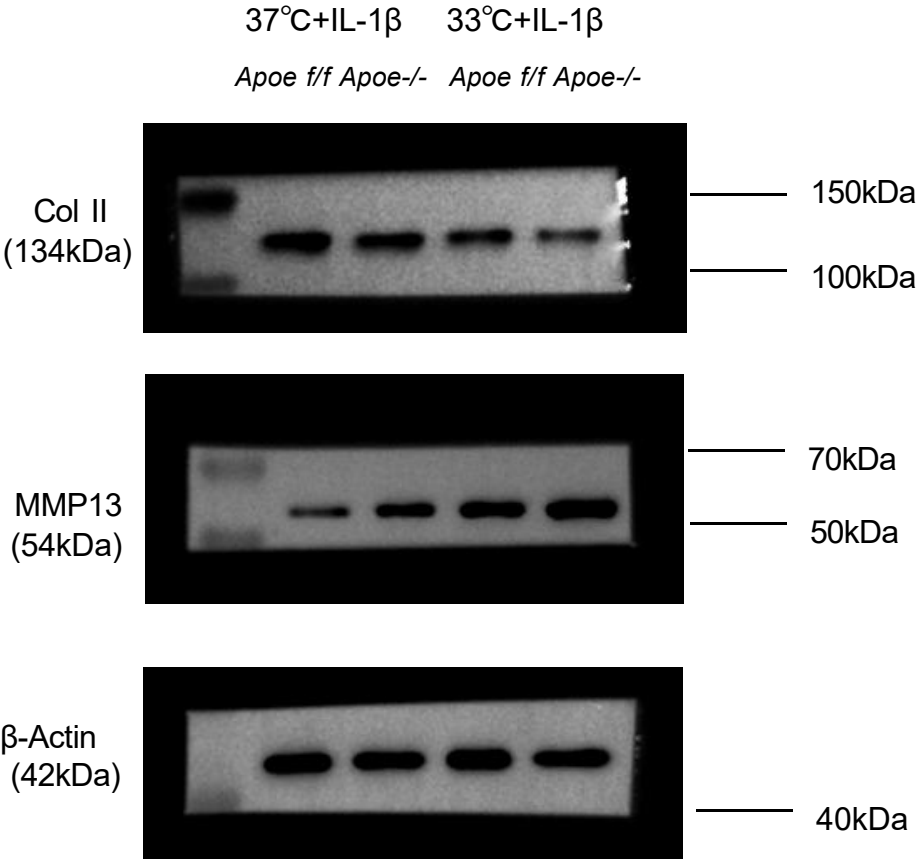

Supplement: Supplementary file 7 — Source data Fig. 3 [file 44321_2025_268_MOESM7_ESM.zip › Figure 3/3I/3I.pdf]

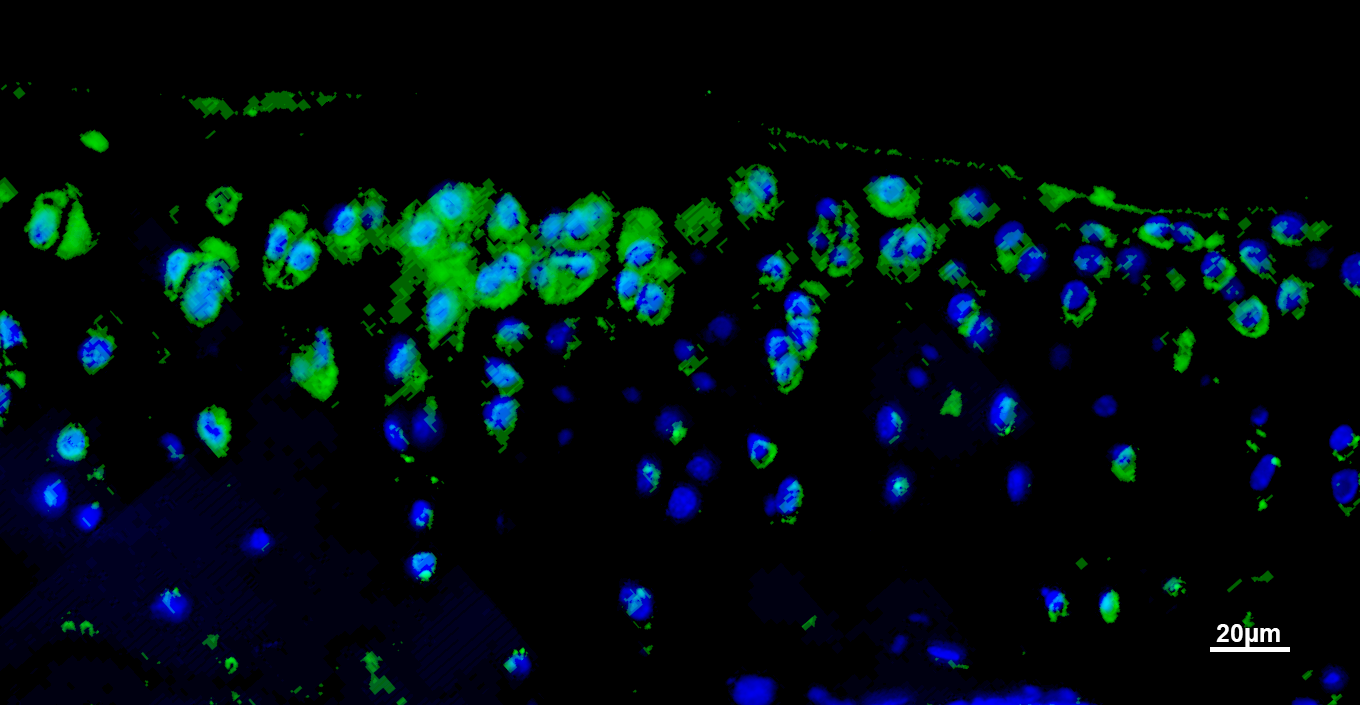

Supplement: Supplementary file 8 — Source data Fig. 4 [file 44321_2025_268_MOESM8_ESM.zip › Figure 4/4A/Cartilage/Apoe cko LTDMM.tif]

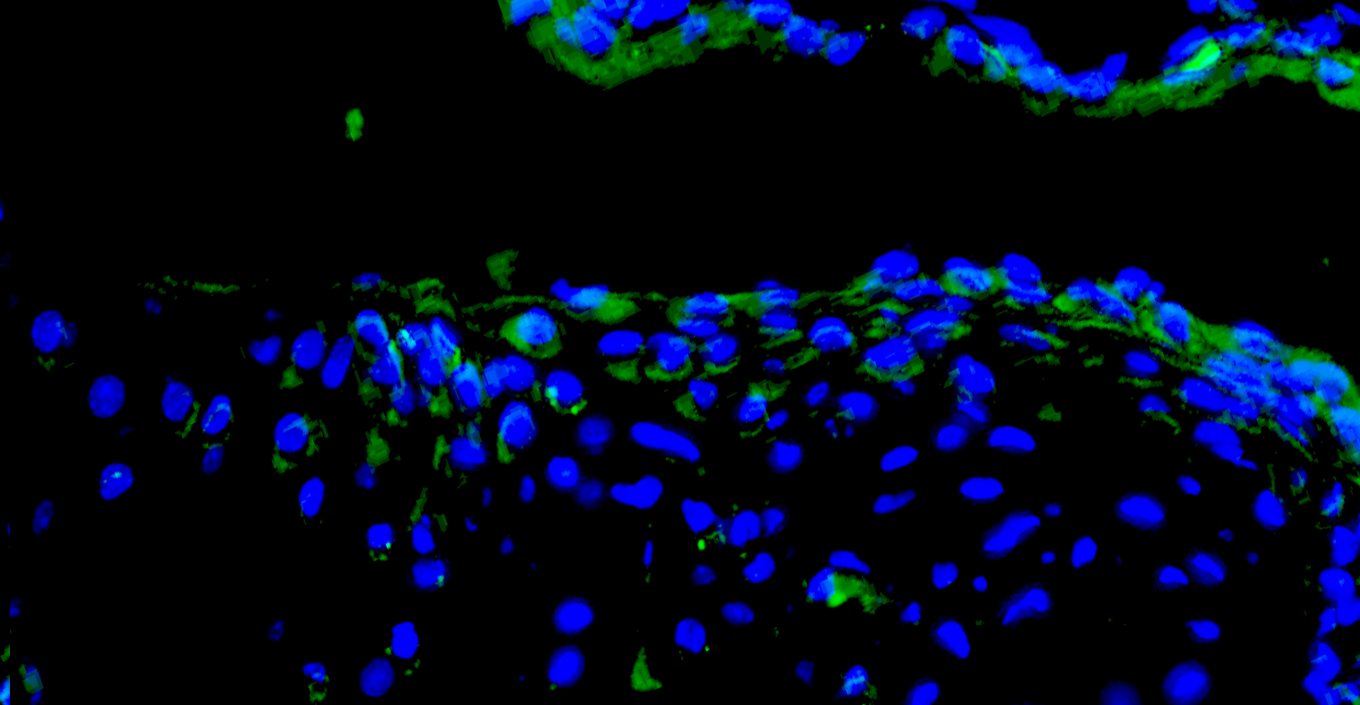

Supplement: Supplementary file 8 — Source data Fig. 4 [file 44321_2025_268_MOESM8_ESM.zip › Figure 4/4A/Cartilage/Apoe cko RTDMM.tif]

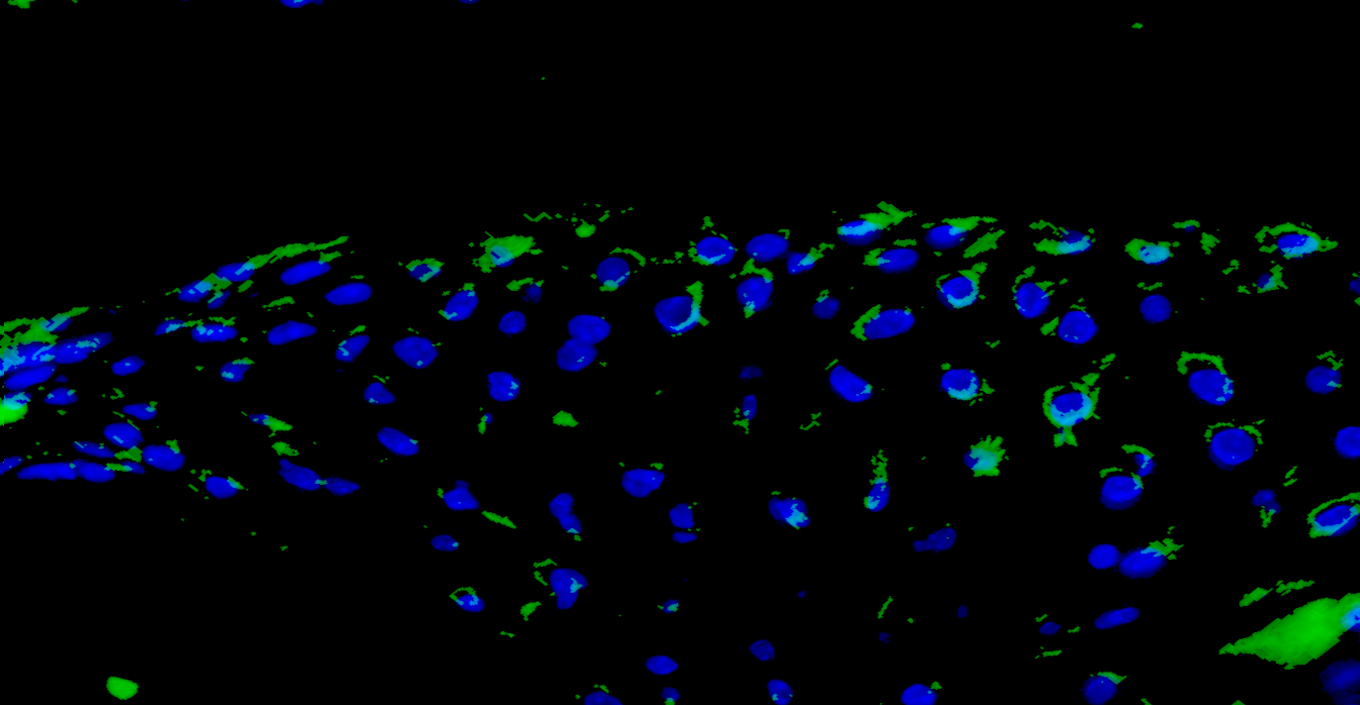

Supplement: Supplementary file 8 — Source data Fig. 4 [file 44321_2025_268_MOESM8_ESM.zip › Figure 4/4A/Cartilage/Apoe flox LTDMM.tif]

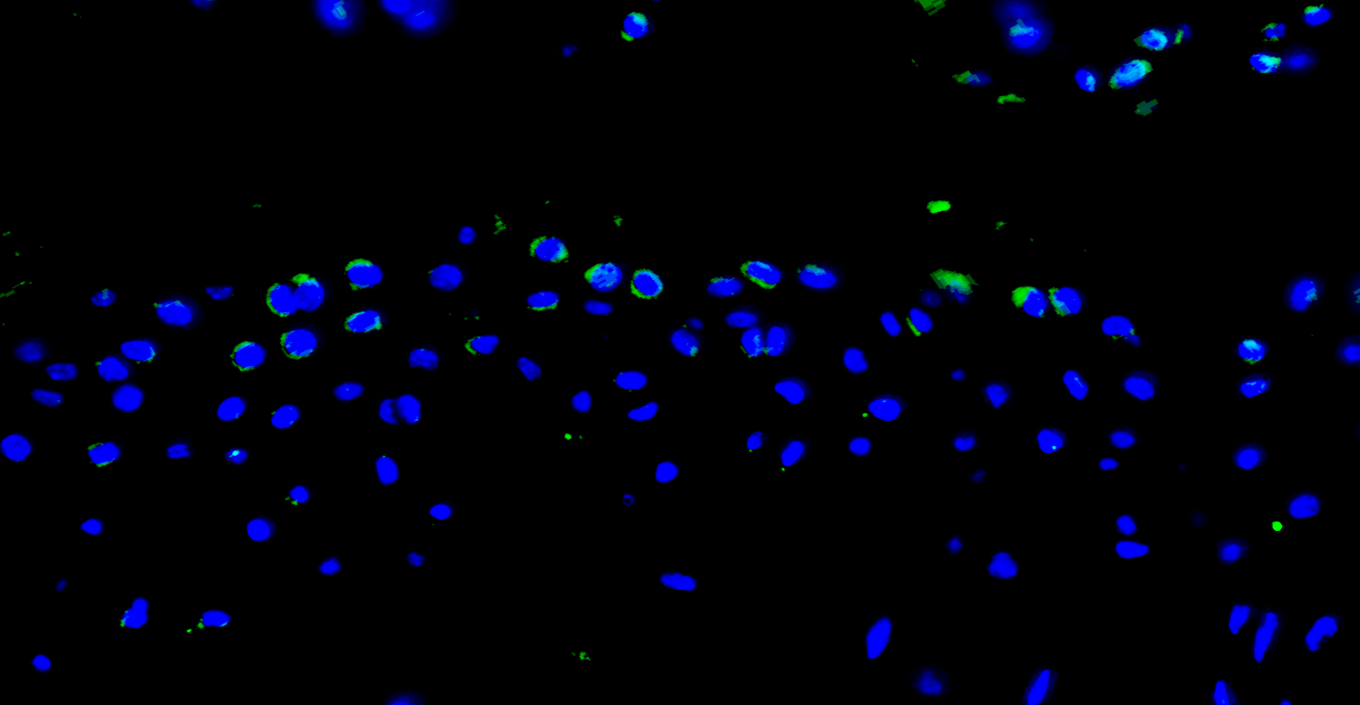

Supplement: Supplementary file 8 — Source data Fig. 4 [file 44321_2025_268_MOESM8_ESM.zip › Figure 4/4A/Cartilage/Apoe flox RTDMM.tif]

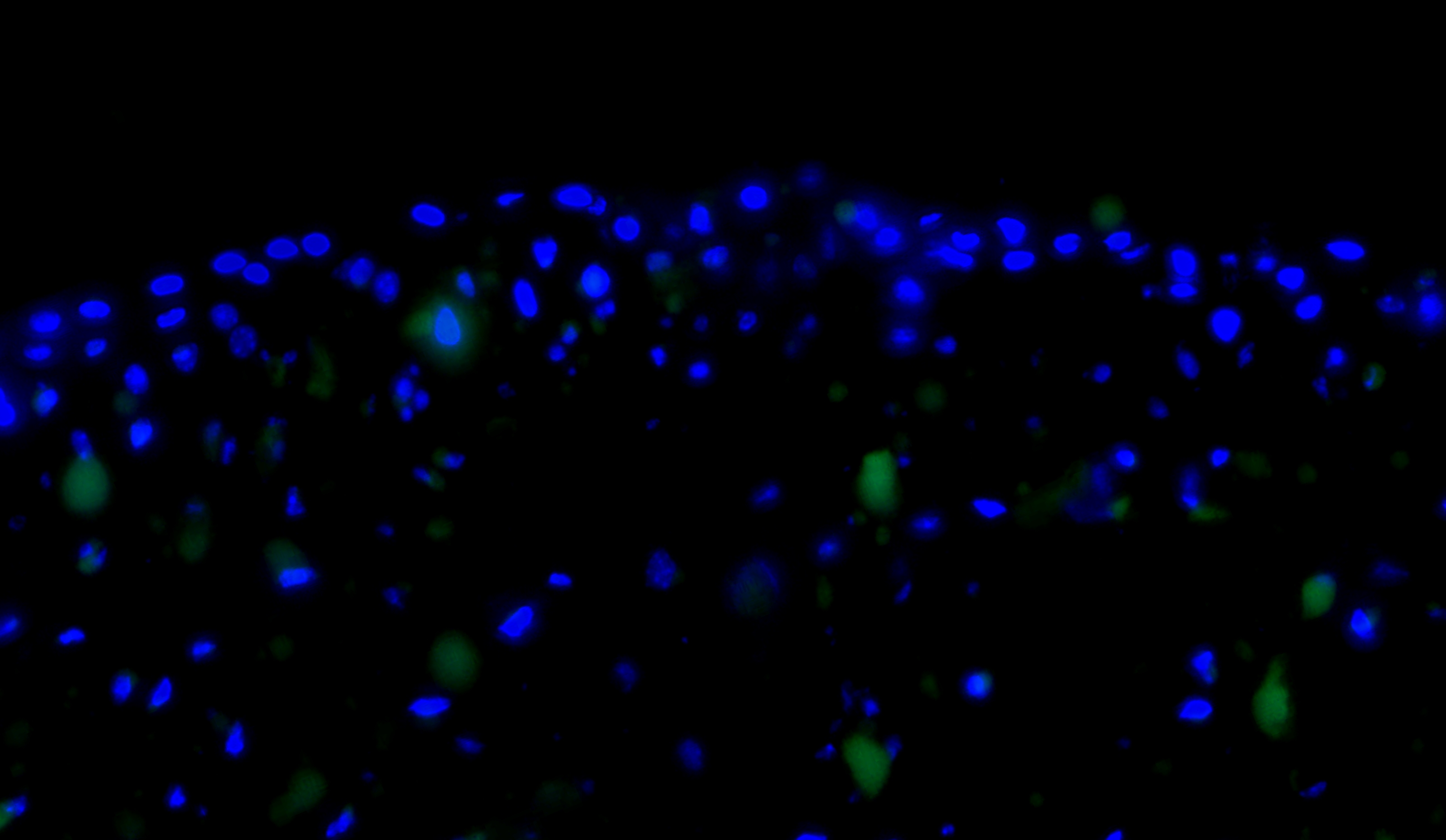

Supplement: Supplementary file 8 — Source data Fig. 4 [file 44321_2025_268_MOESM8_ESM.zip › Figure 4/4A/Cartilage/HFD.jpg]

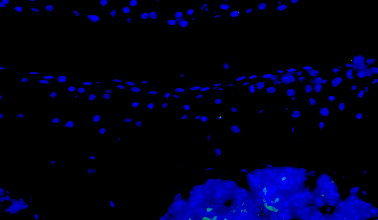

Supplement: Supplementary file 8 — Source data Fig. 4 [file 44321_2025_268_MOESM8_ESM.zip › Figure 4/4A/Cartilage/SHAM.tif]

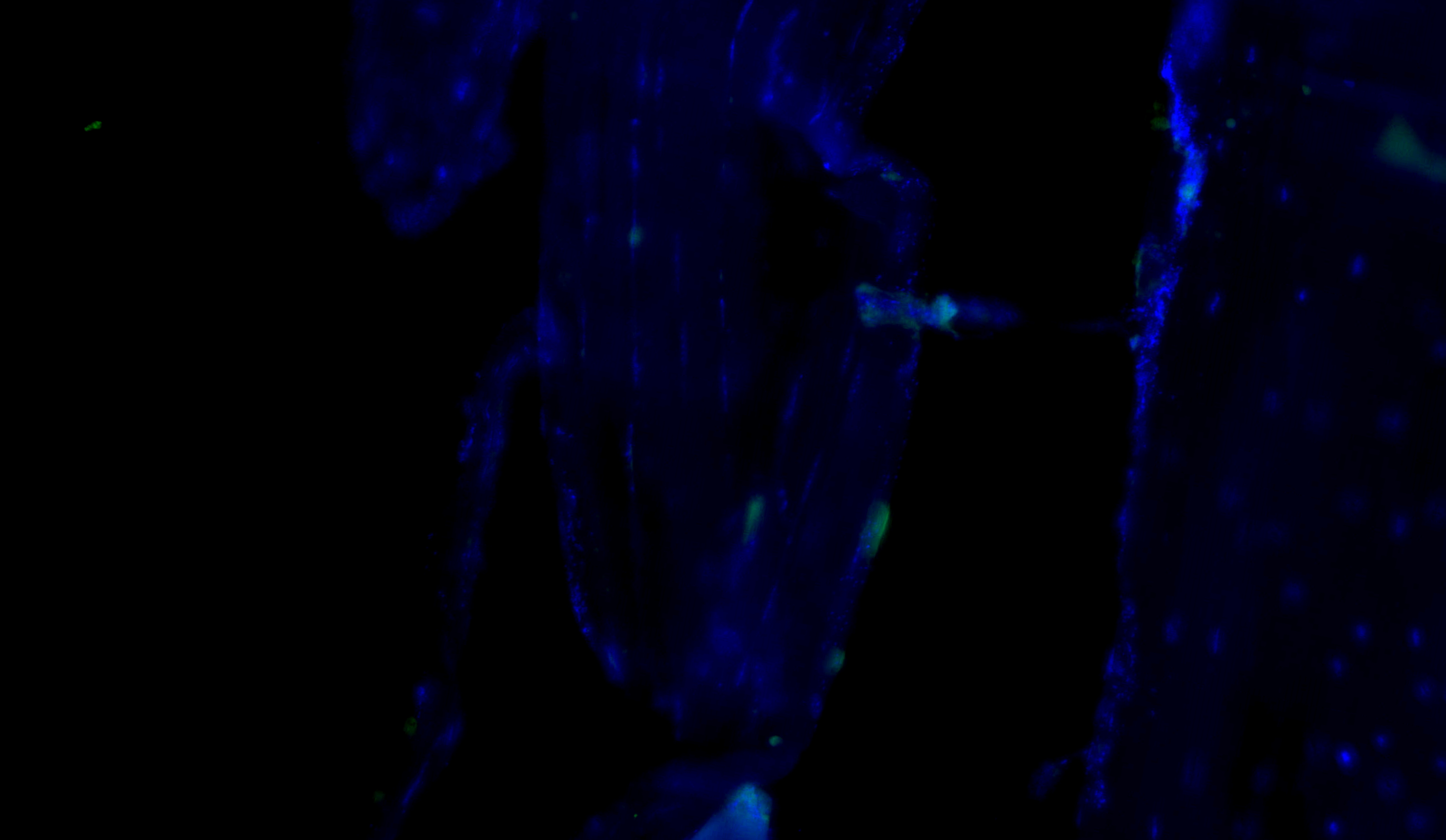

Supplement: Supplementary file 8 — Source data Fig. 4 [file 44321_2025_268_MOESM8_ESM.zip › Figure 4/4A/distal tibial/Apoe cko LTdmm.jpg]

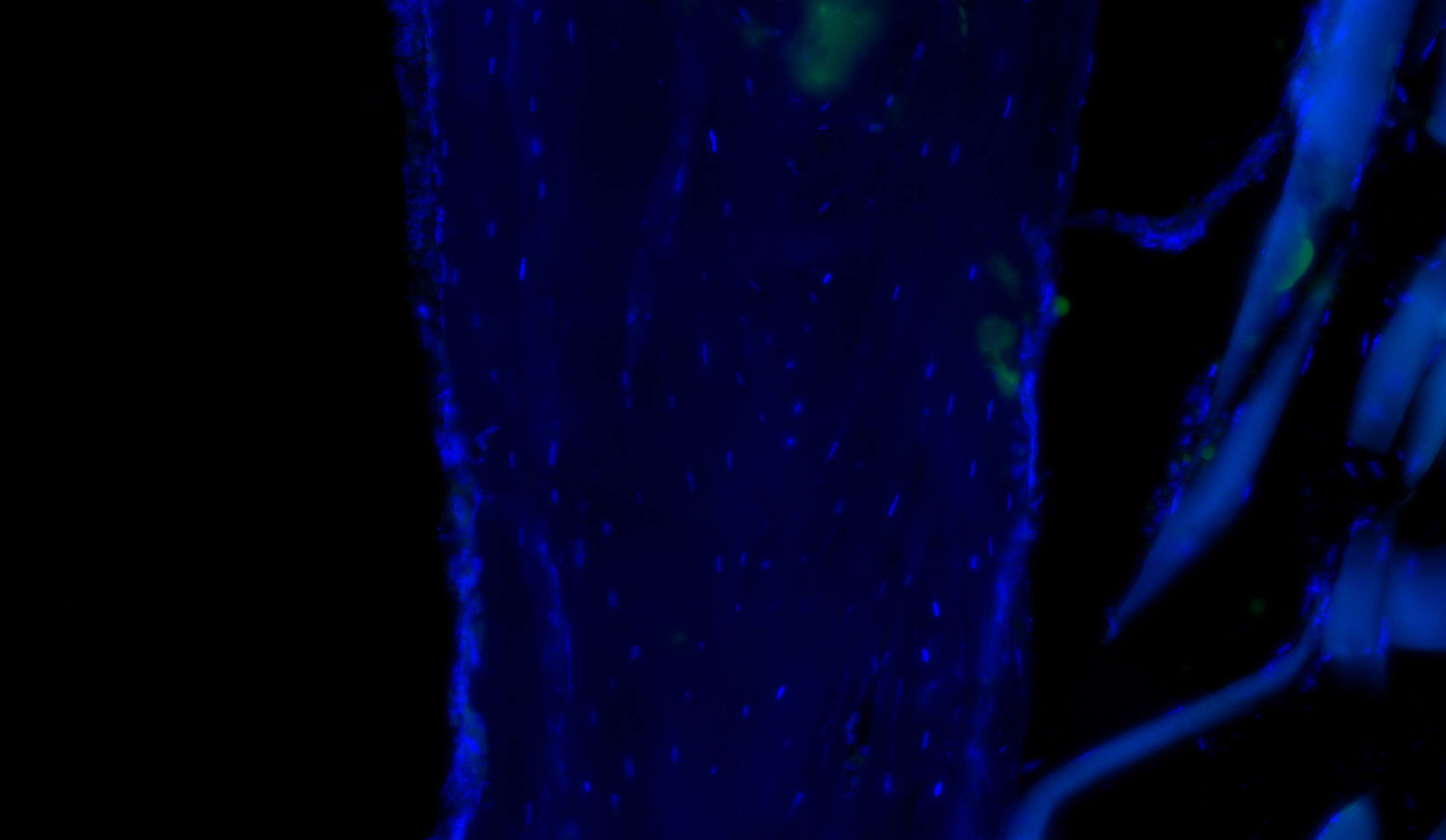

Supplement: Supplementary file 8 — Source data Fig. 4 [file 44321_2025_268_MOESM8_ESM.zip › Figure 4/4A/distal tibial/Apoe cko RTDMM.jpg]

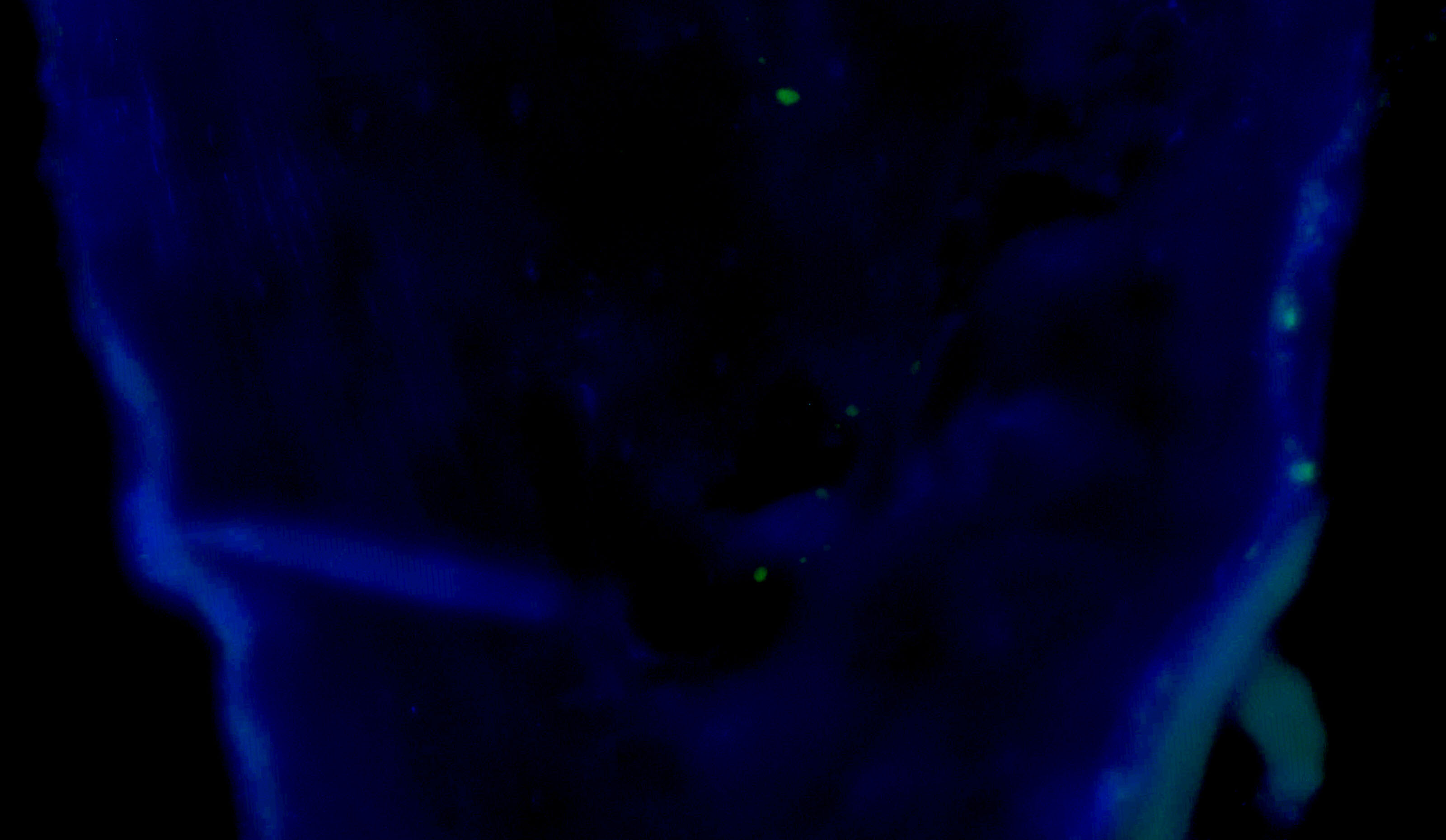

Supplement: Supplementary file 8 — Source data Fig. 4 [file 44321_2025_268_MOESM8_ESM.zip › Figure 4/4A/distal tibial/Apoe flox LTdmm.jpg]

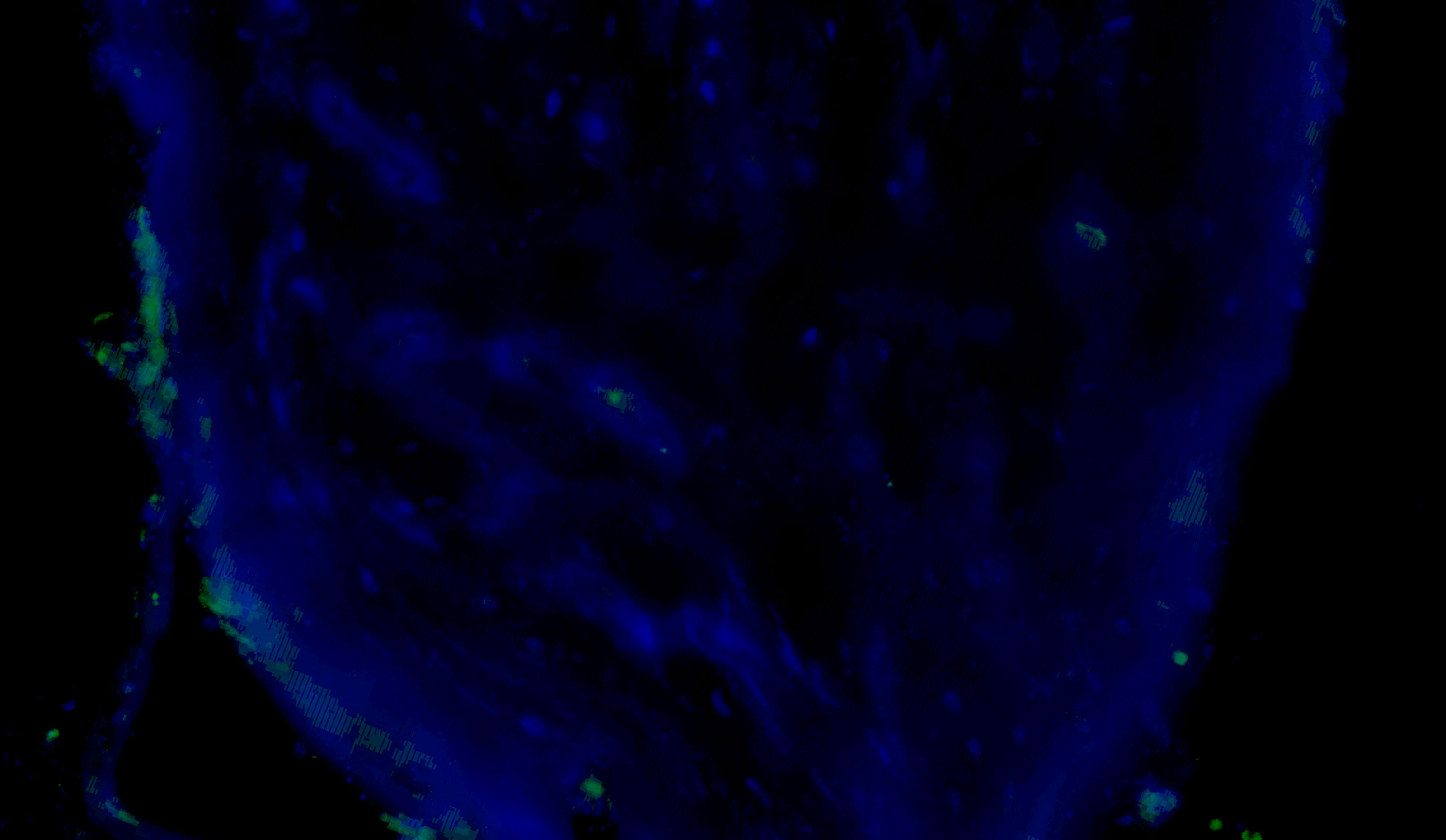

Supplement: Supplementary file 8 — Source data Fig. 4 [file 44321_2025_268_MOESM8_ESM.zip › Figure 4/4A/distal tibial/Apoe flox RTDMM.jpg]

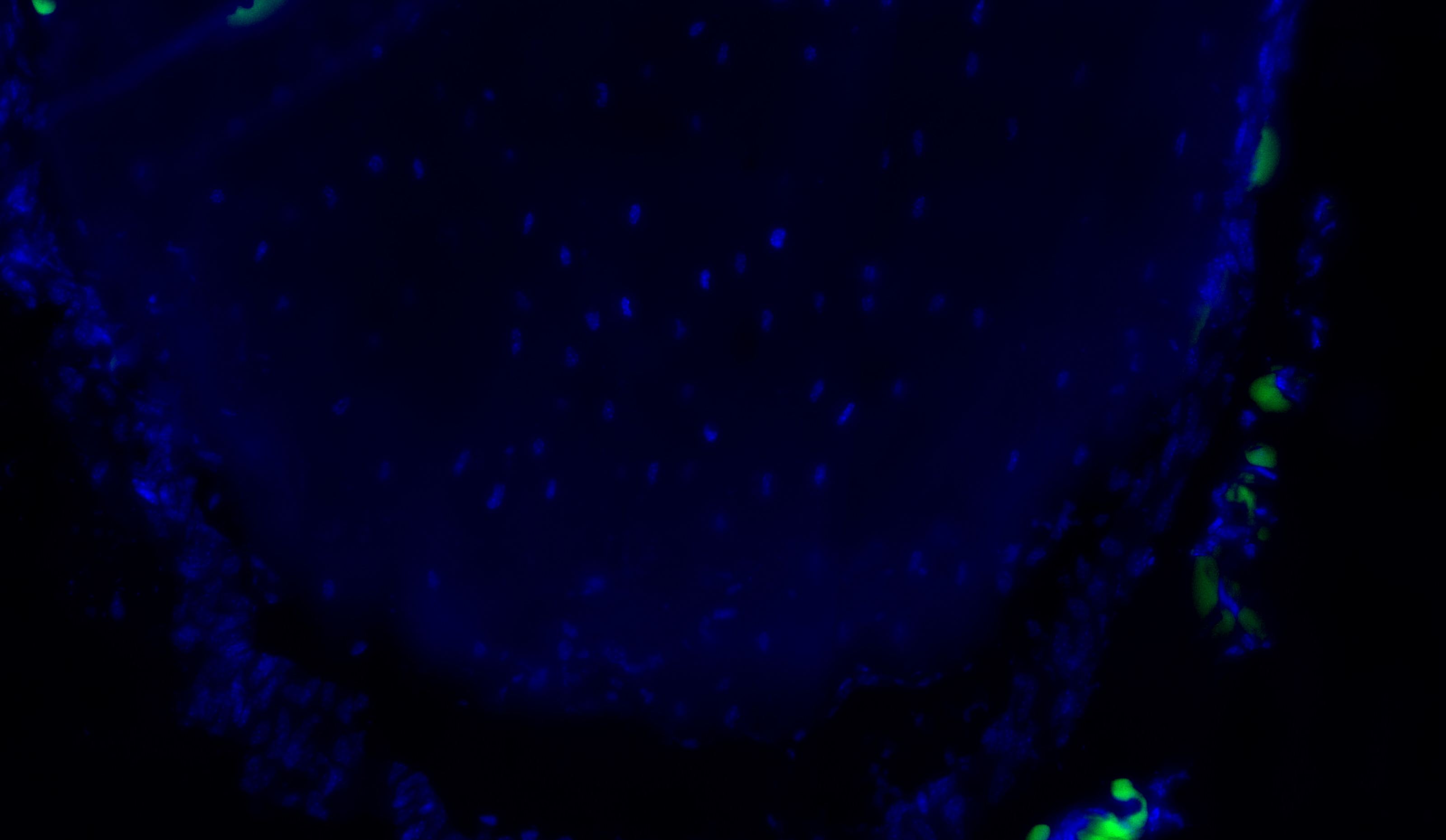

Supplement: Supplementary file 8 — Source data Fig. 4 [file 44321_2025_268_MOESM8_ESM.zip › Figure 4/4A/distal tibial/HFD.jpg]

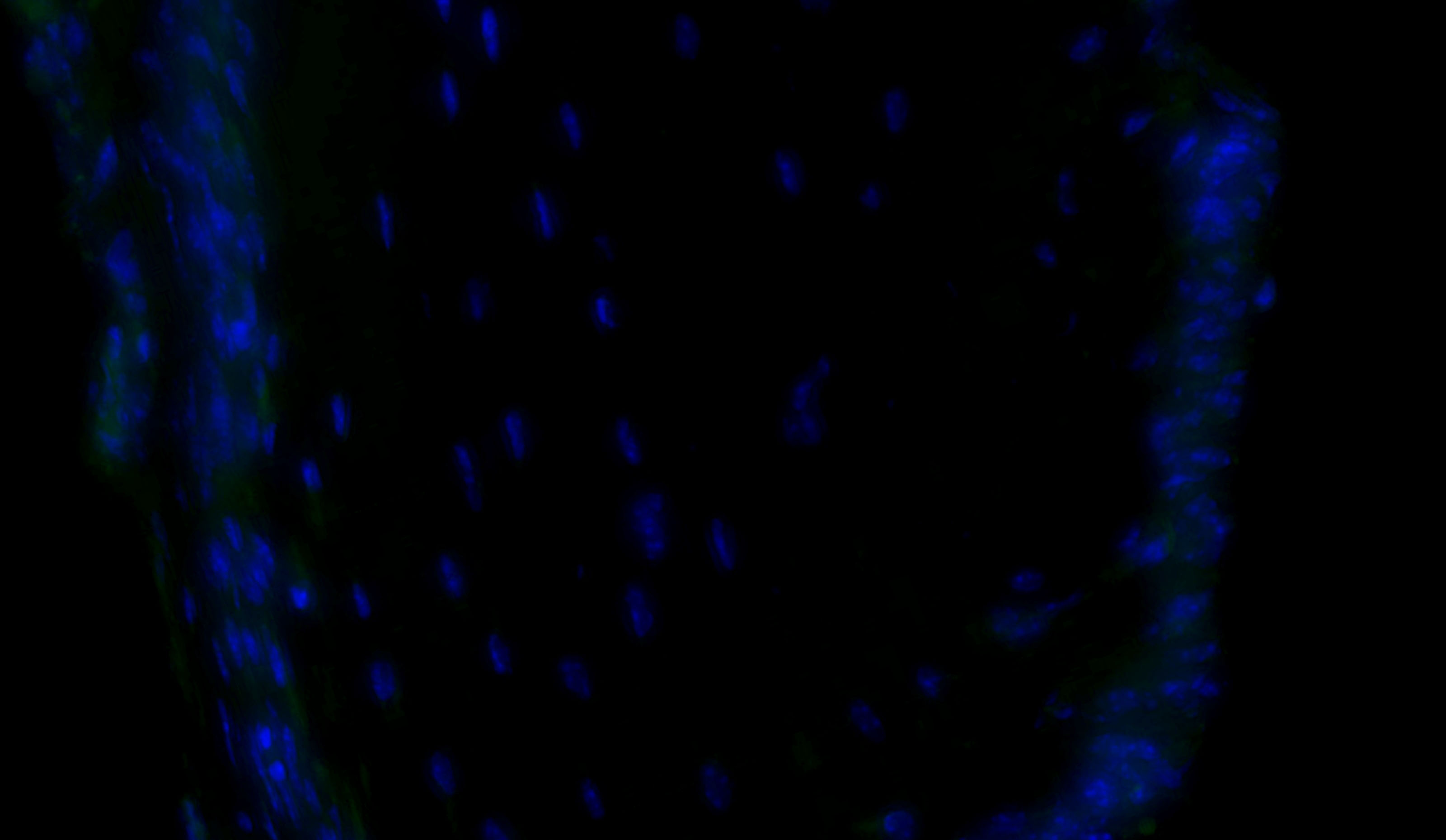

Supplement: Supplementary file 8 — Source data Fig. 4 [file 44321_2025_268_MOESM8_ESM.zip › Figure 4/4A/distal tibial/SHAM.jpg]

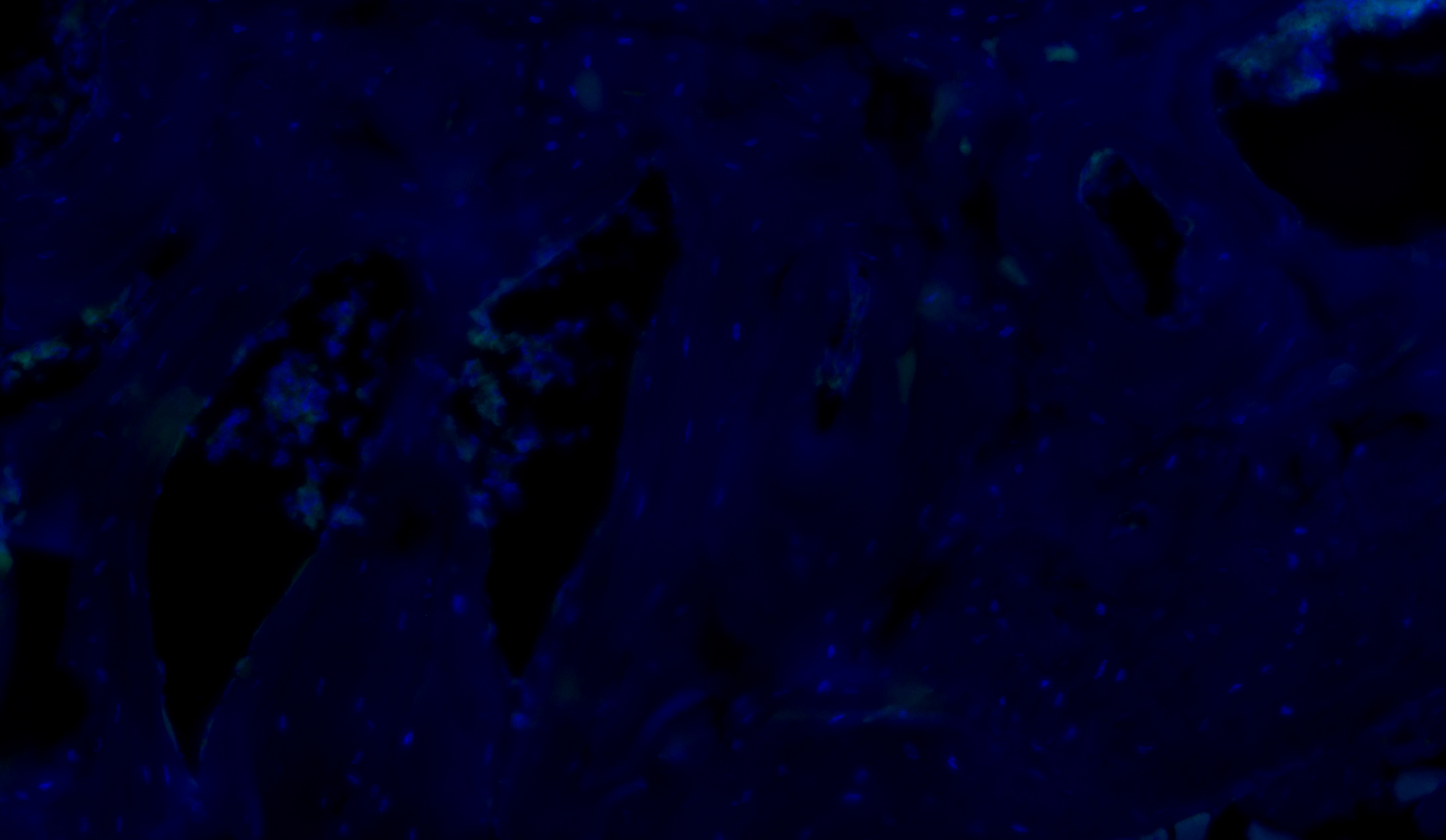

Supplement: Supplementary file 8 — Source data Fig. 4 [file 44321_2025_268_MOESM8_ESM.zip › Figure 4/4A/Proximal tibial/Apoe cko LTDMM.jpg]

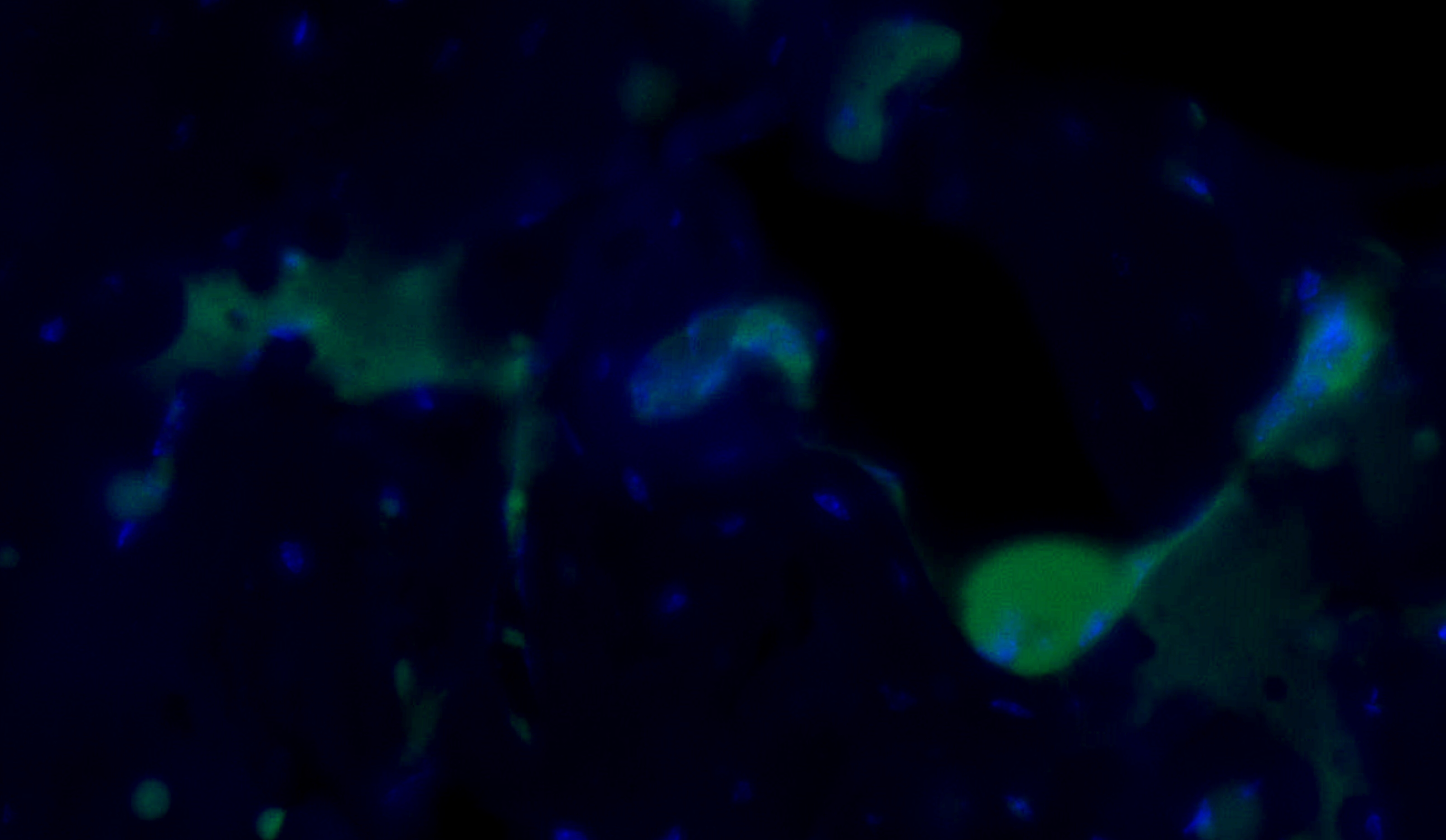

Supplement: Supplementary file 8 — Source data Fig. 4 [file 44321_2025_268_MOESM8_ESM.zip › Figure 4/4A/Proximal tibial/Apoe cko RTDMM.jpg]

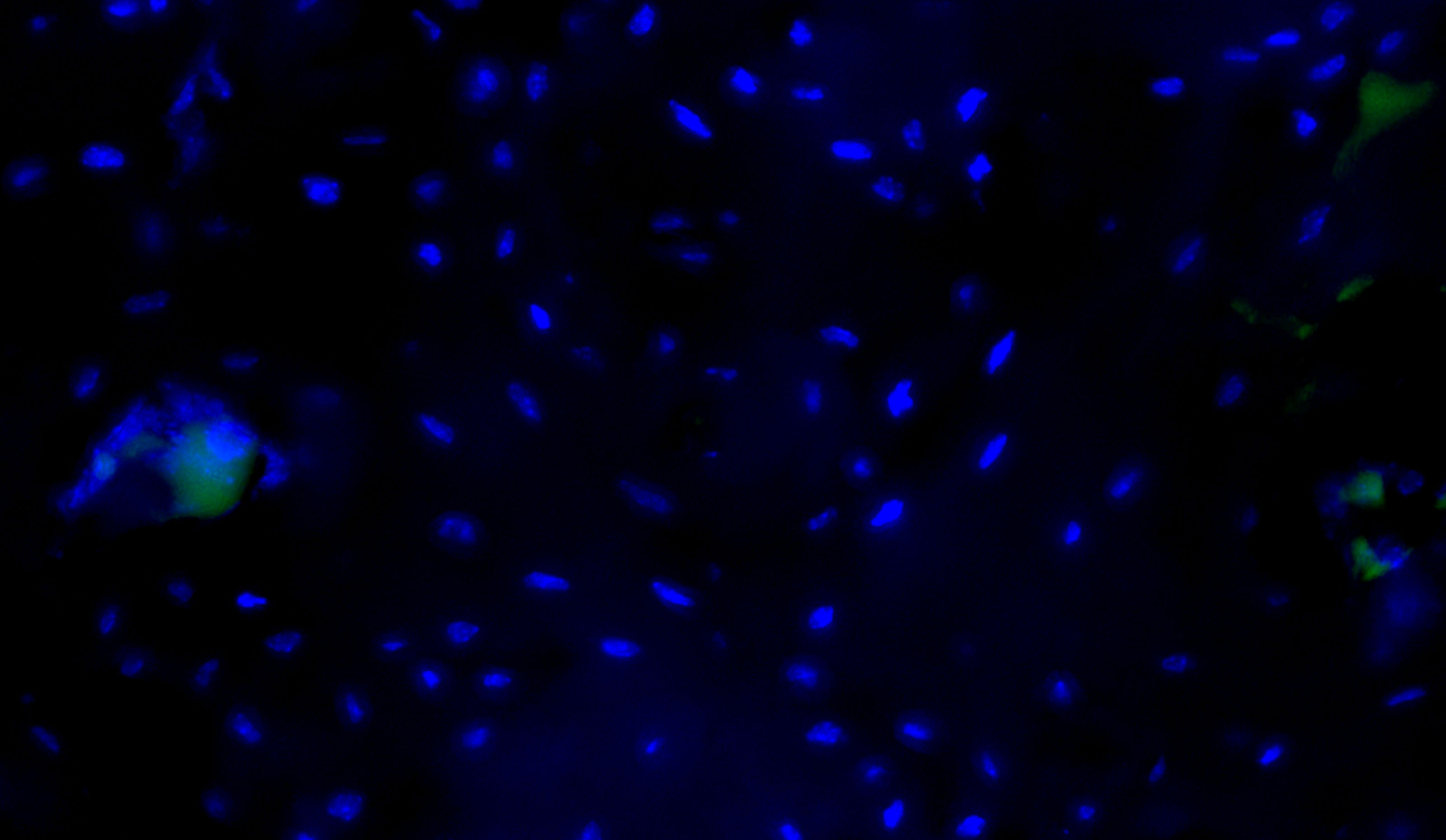

Supplement: Supplementary file 8 — Source data Fig. 4 [file 44321_2025_268_MOESM8_ESM.zip › Figure 4/4A/Proximal tibial/Apoe flox LTDMM.jpg]

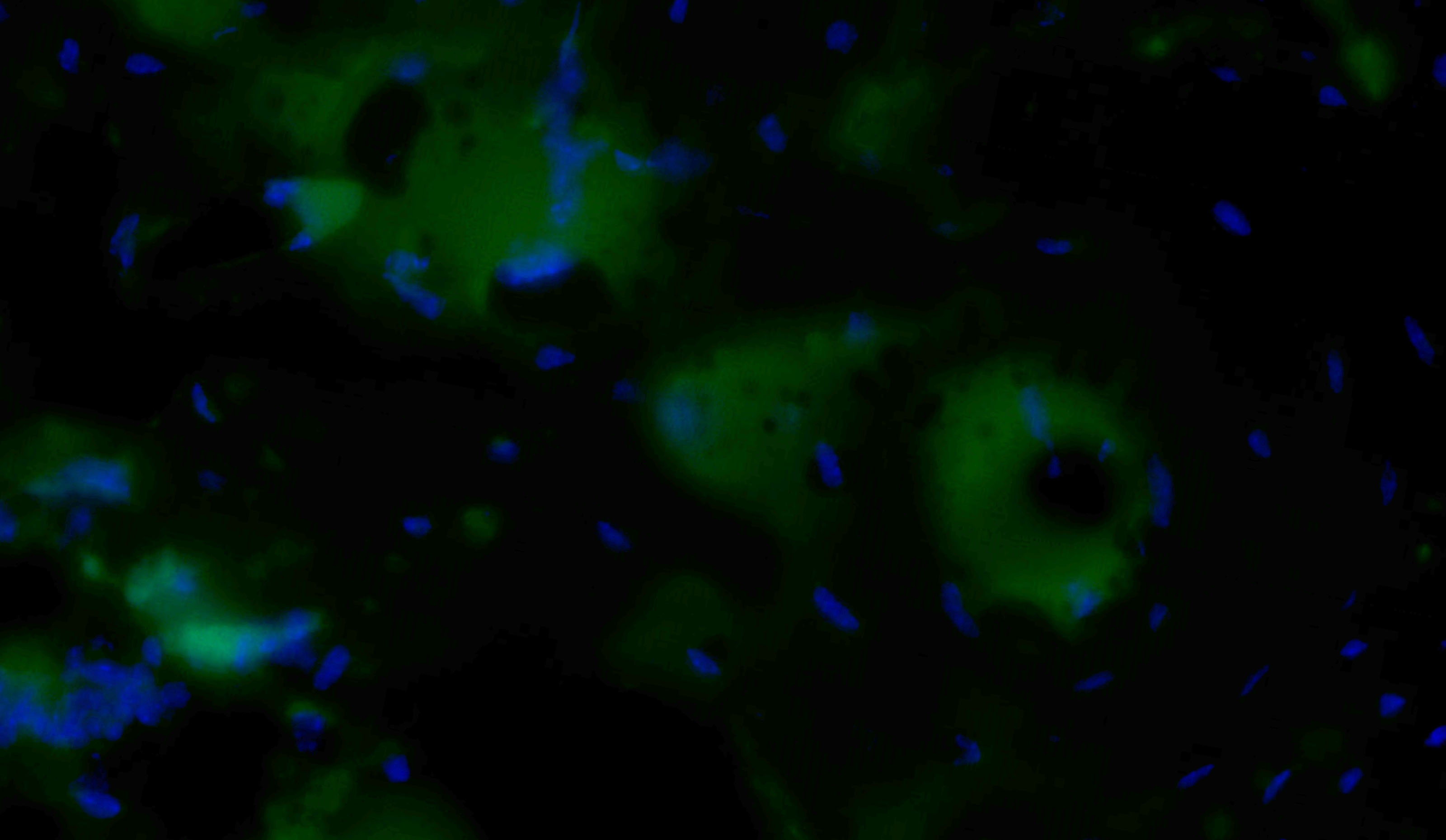

Supplement: Supplementary file 8 — Source data Fig. 4 [file 44321_2025_268_MOESM8_ESM.zip › Figure 4/4A/Proximal tibial/Apoe flox RTDMM.jpg]

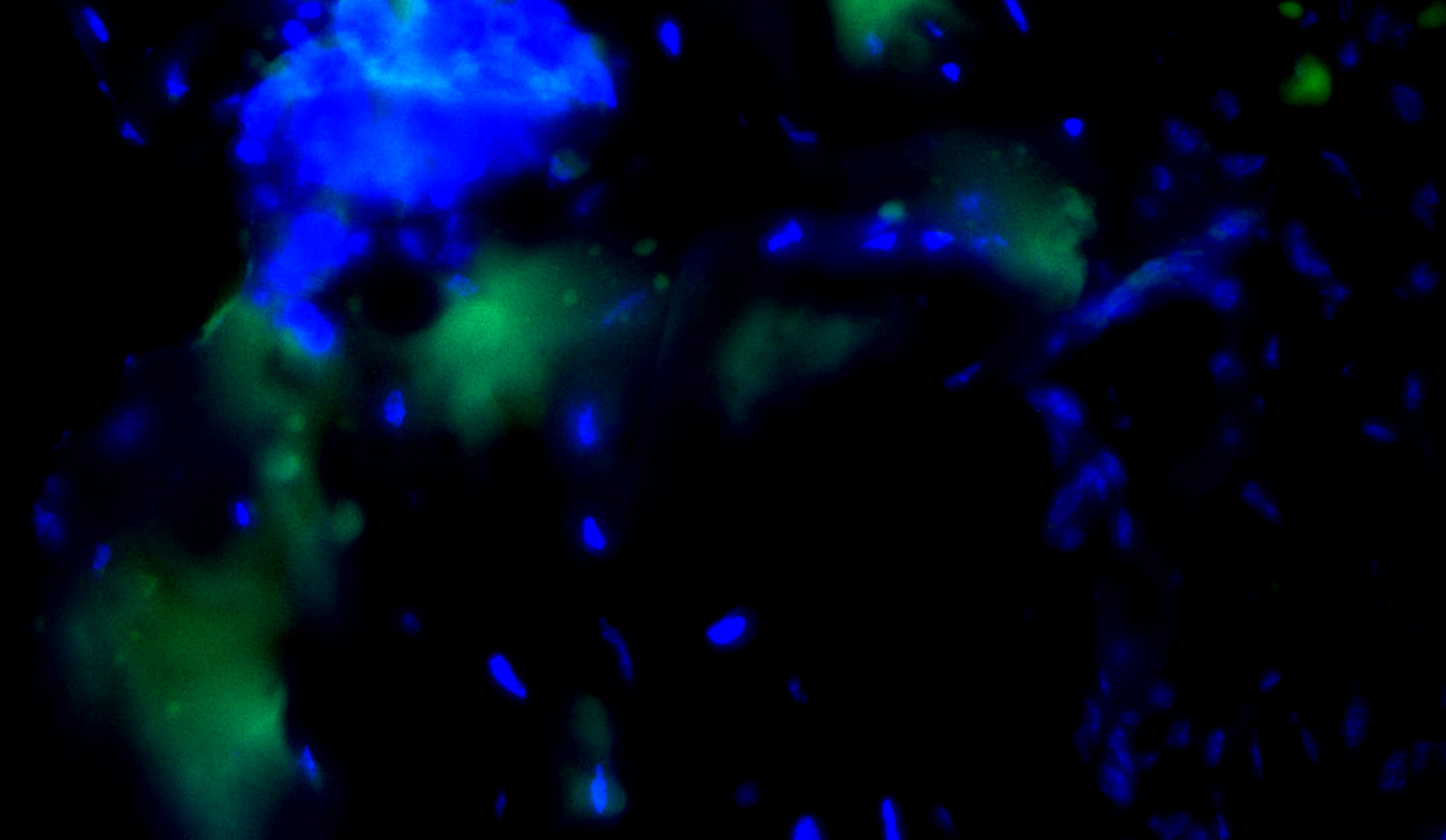

Supplement: Supplementary file 8 — Source data Fig. 4 [file 44321_2025_268_MOESM8_ESM.zip › Figure 4/4A/Proximal tibial/HFD.jpg]

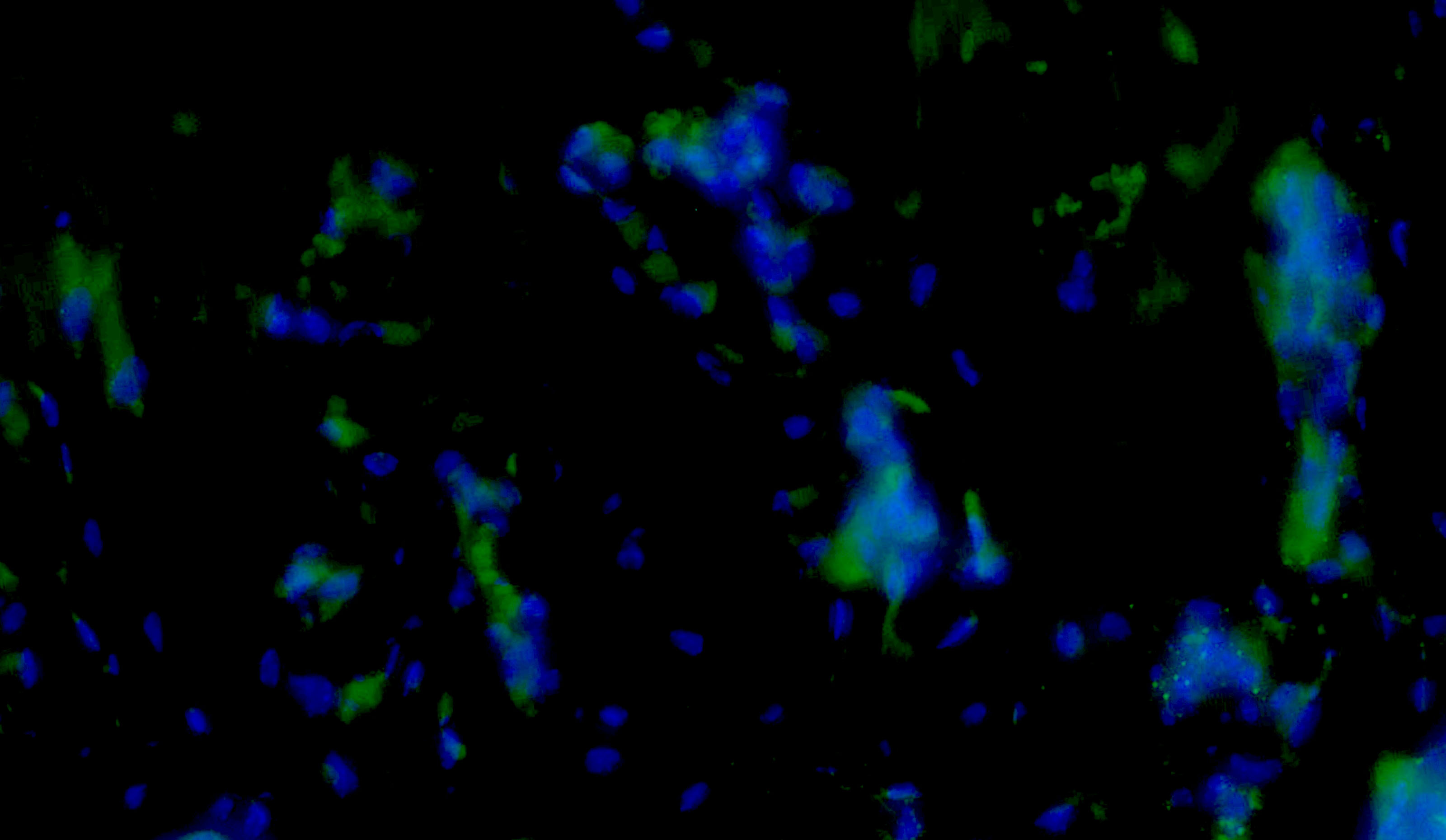

Supplement: Supplementary file 8 — Source data Fig. 4 [file 44321_2025_268_MOESM8_ESM.zip › Figure 4/4A/Proximal tibial/SHAM.jpg]

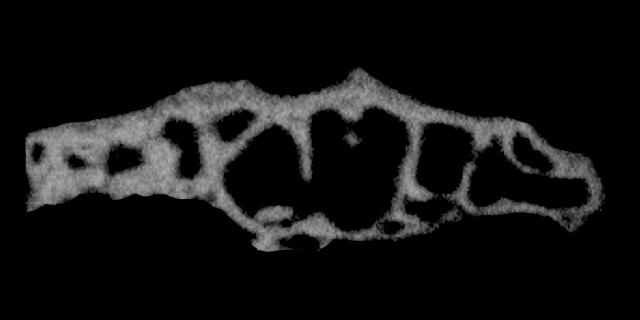

Supplement: Supplementary file 8 — Source data Fig. 4 [file 44321_2025_268_MOESM8_ESM.zip › Figure 4/4B/Apoe cko LTDMM.tif]

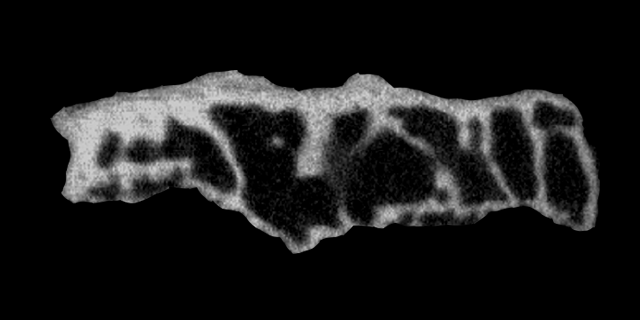

Supplement: Supplementary file 8 — Source data Fig. 4 [file 44321_2025_268_MOESM8_ESM.zip › Figure 4/4B/Apoe cko RTDMM.tif]

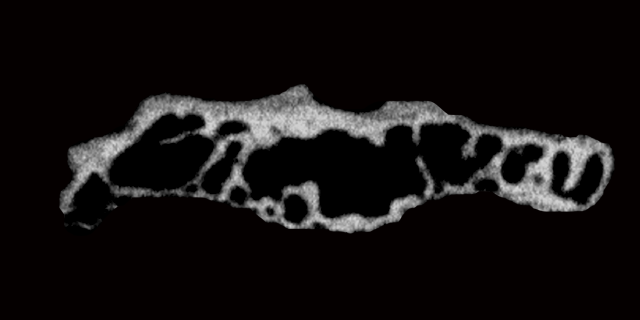

Supplement: Supplementary file 8 — Source data Fig. 4 [file 44321_2025_268_MOESM8_ESM.zip › Figure 4/4B/Apoe flox LTDMM.tif]

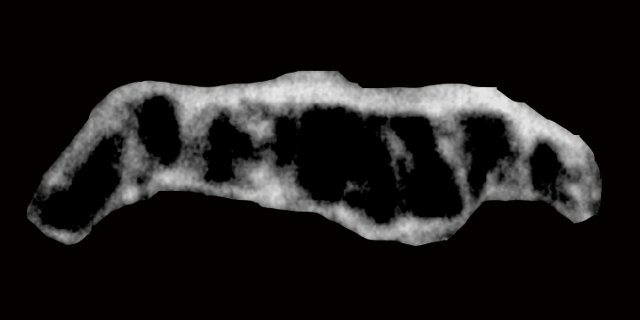

Supplement: Supplementary file 8 — Source data Fig. 4 [file 44321_2025_268_MOESM8_ESM.zip › Figure 4/4B/Apoe flox RTDMM.tif]

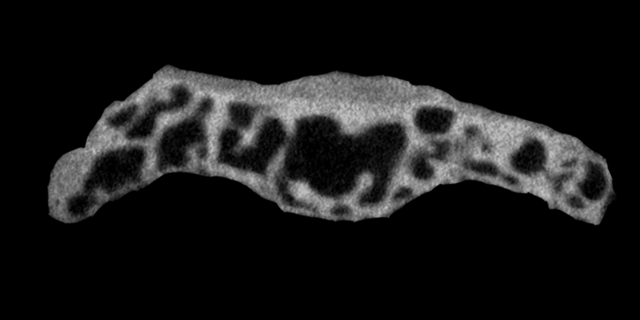

Supplement: Supplementary file 8 — Source data Fig. 4 [file 44321_2025_268_MOESM8_ESM.zip › Figure 4/4B/HFD.tif]

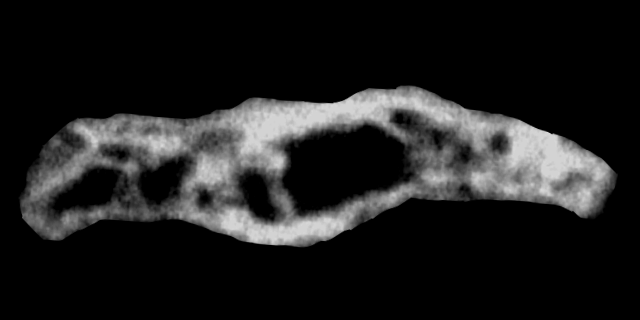

Supplement: Supplementary file 8 — Source data Fig. 4 [file 44321_2025_268_MOESM8_ESM.zip › Figure 4/4B/SHAM.tif]

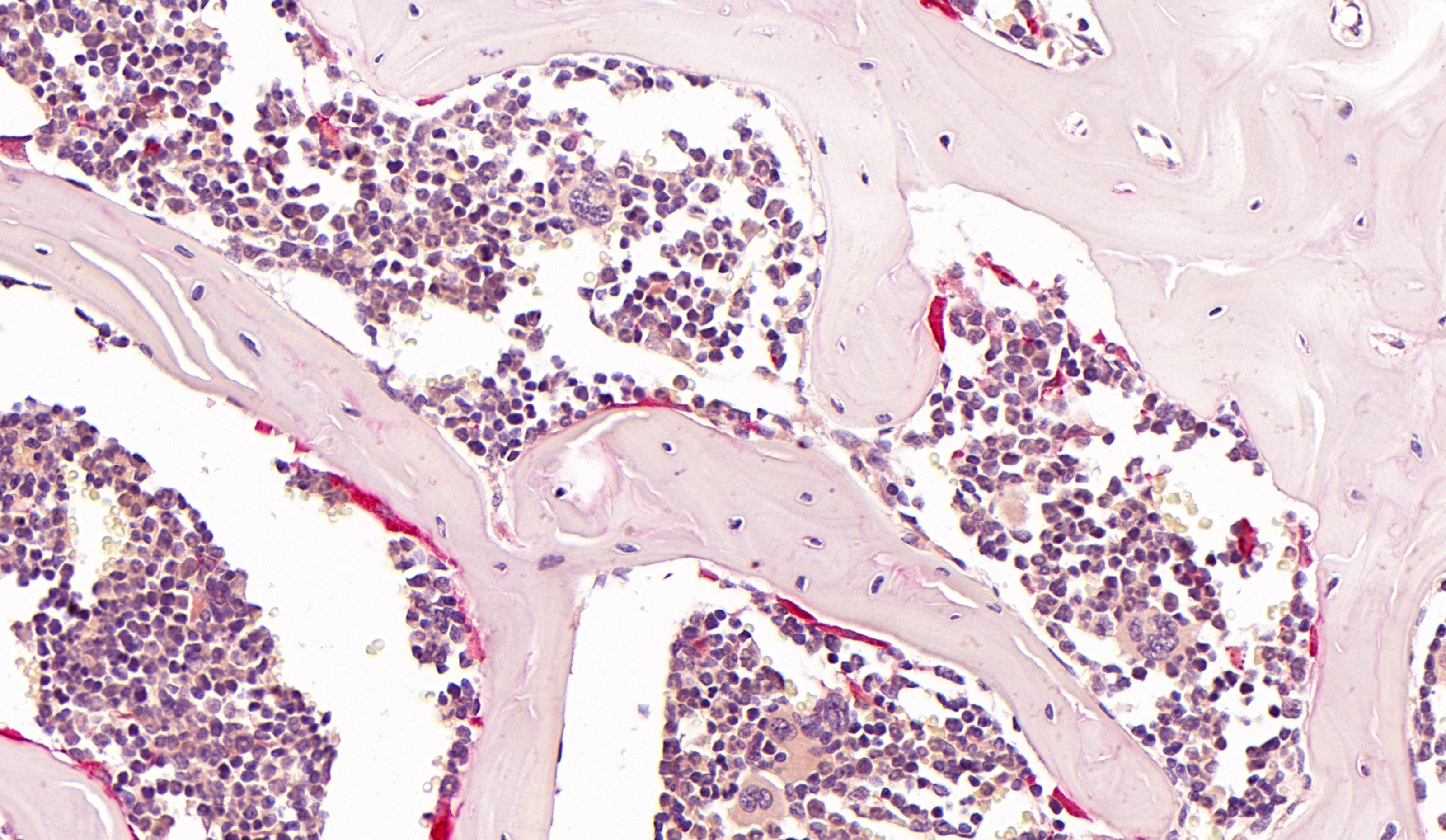

Supplement: Supplementary file 8 — Source data Fig. 4 [file 44321_2025_268_MOESM8_ESM.zip › Figure 4/4C/Apoe cko LTDMM.jpg]

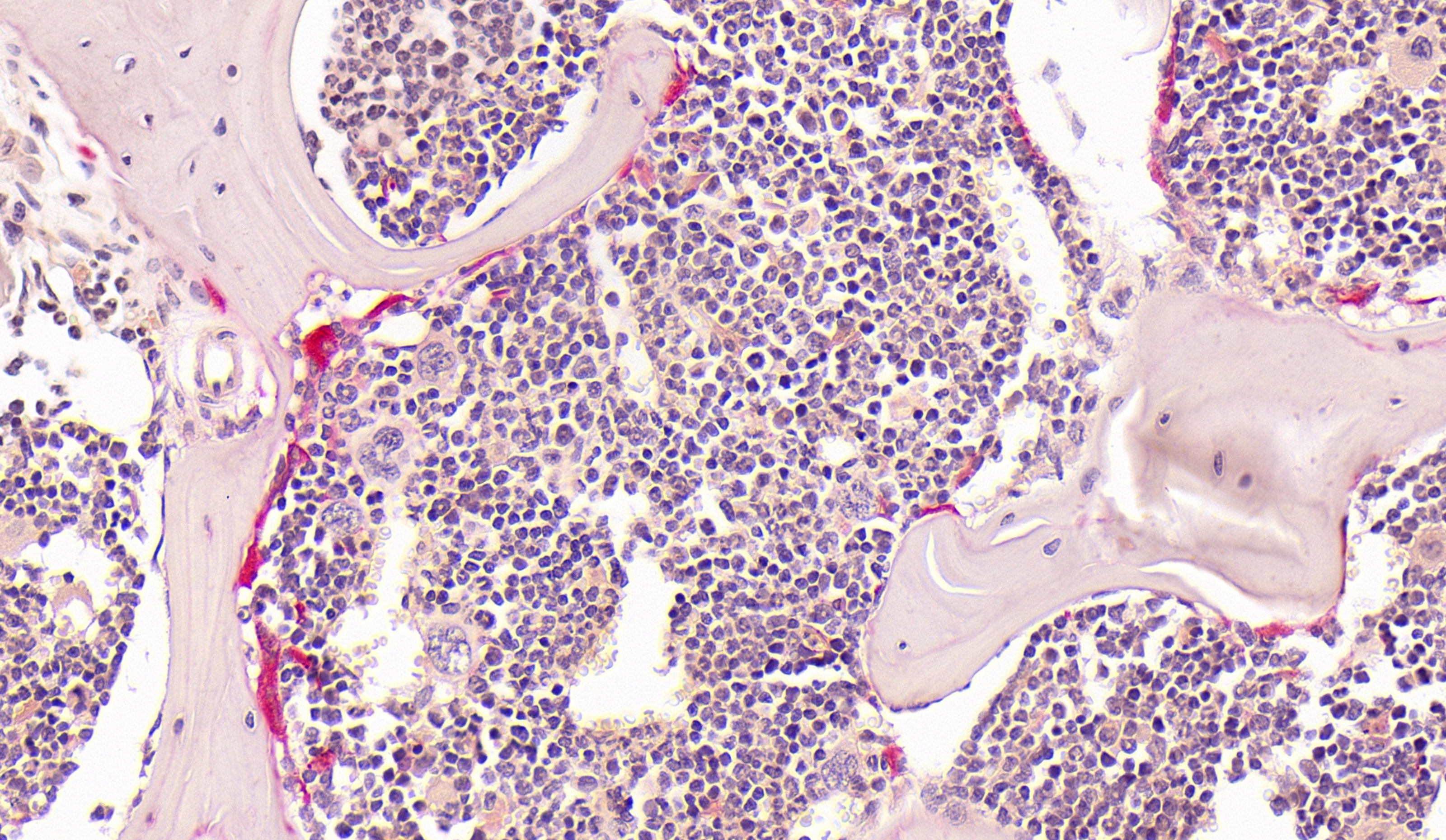

Supplement: Supplementary file 8 — Source data Fig. 4 [file 44321_2025_268_MOESM8_ESM.zip › Figure 4/4C/Apoe cko RTDMM.jpg]

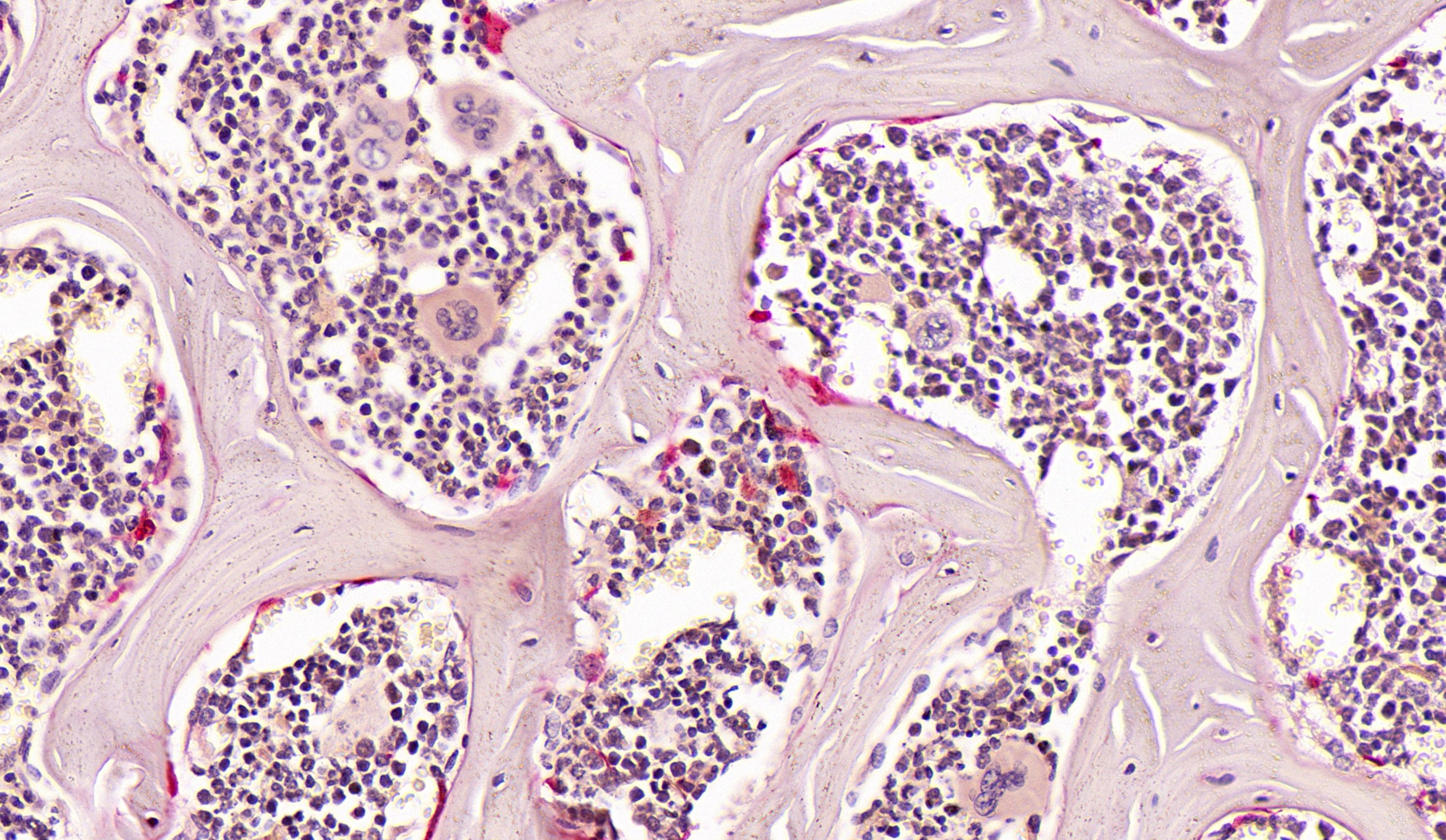

Supplement: Supplementary file 8 — Source data Fig. 4 [file 44321_2025_268_MOESM8_ESM.zip › Figure 4/4C/Apoe flox LTDMM.jpg]

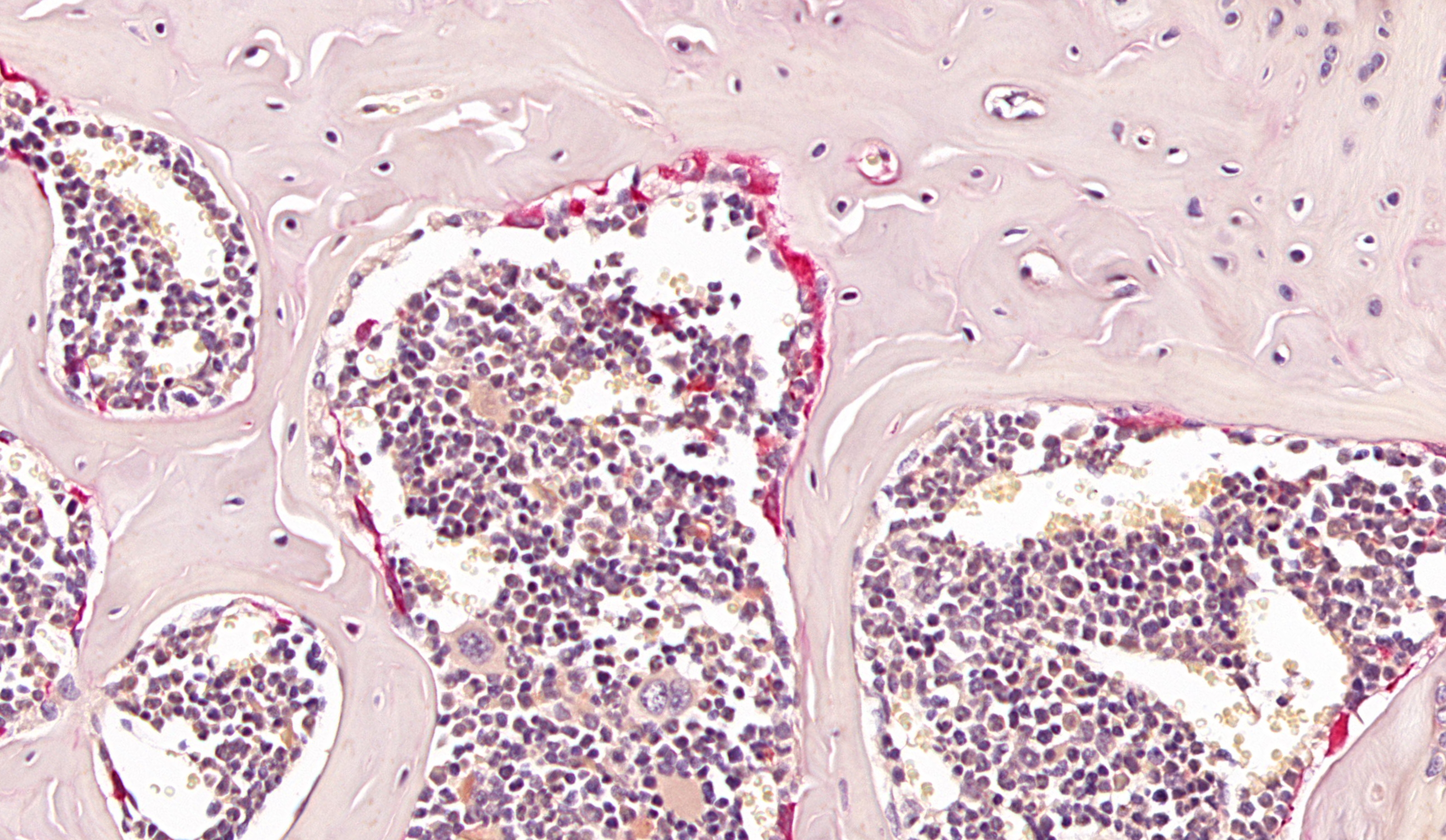

Supplement: Supplementary file 8 — Source data Fig. 4 [file 44321_2025_268_MOESM8_ESM.zip › Figure 4/4C/Apoe flox RTDMM.jpg]

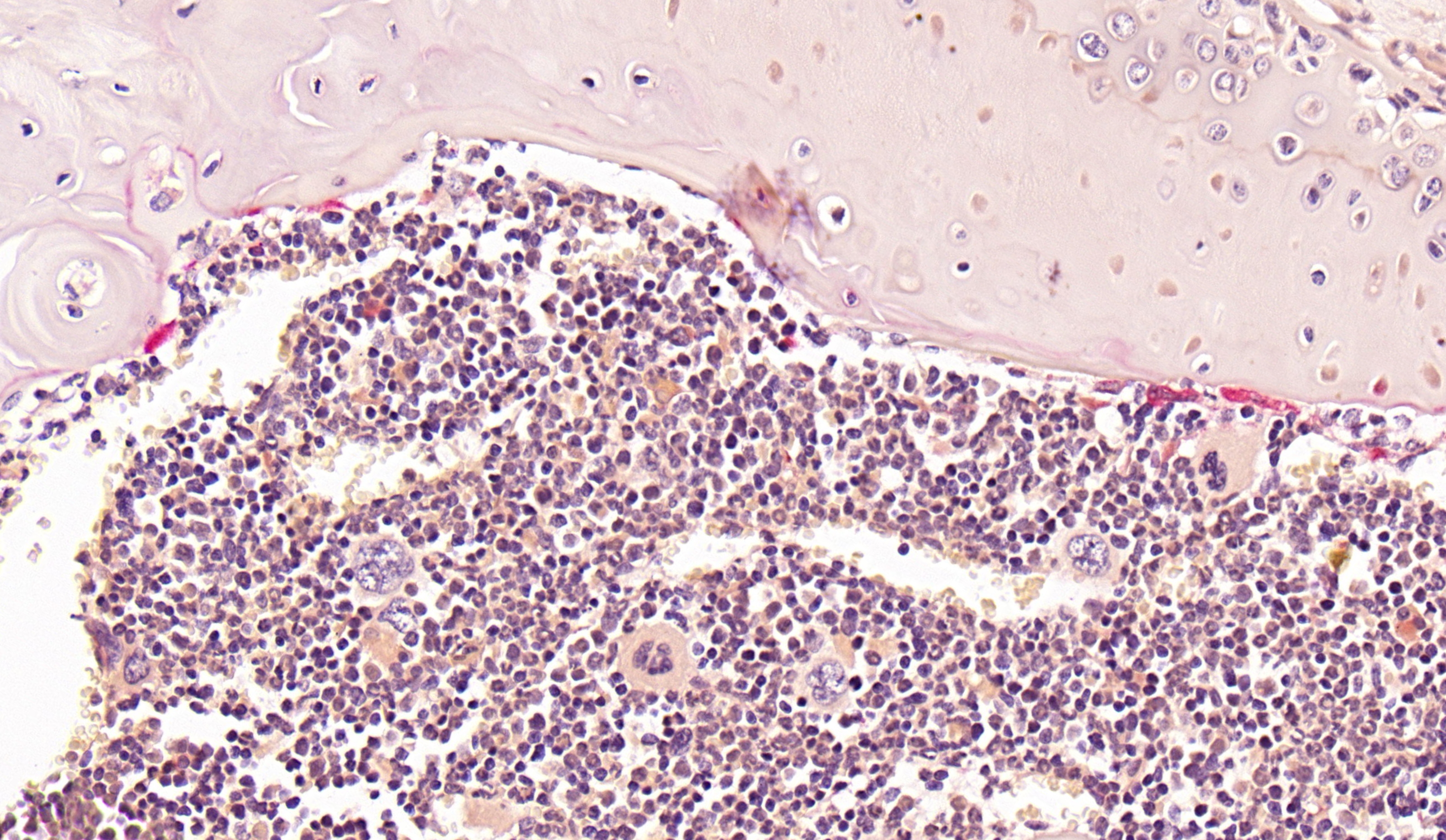

Supplement: Supplementary file 8 — Source data Fig. 4 [file 44321_2025_268_MOESM8_ESM.zip › Figure 4/4C/HFD.jpg]

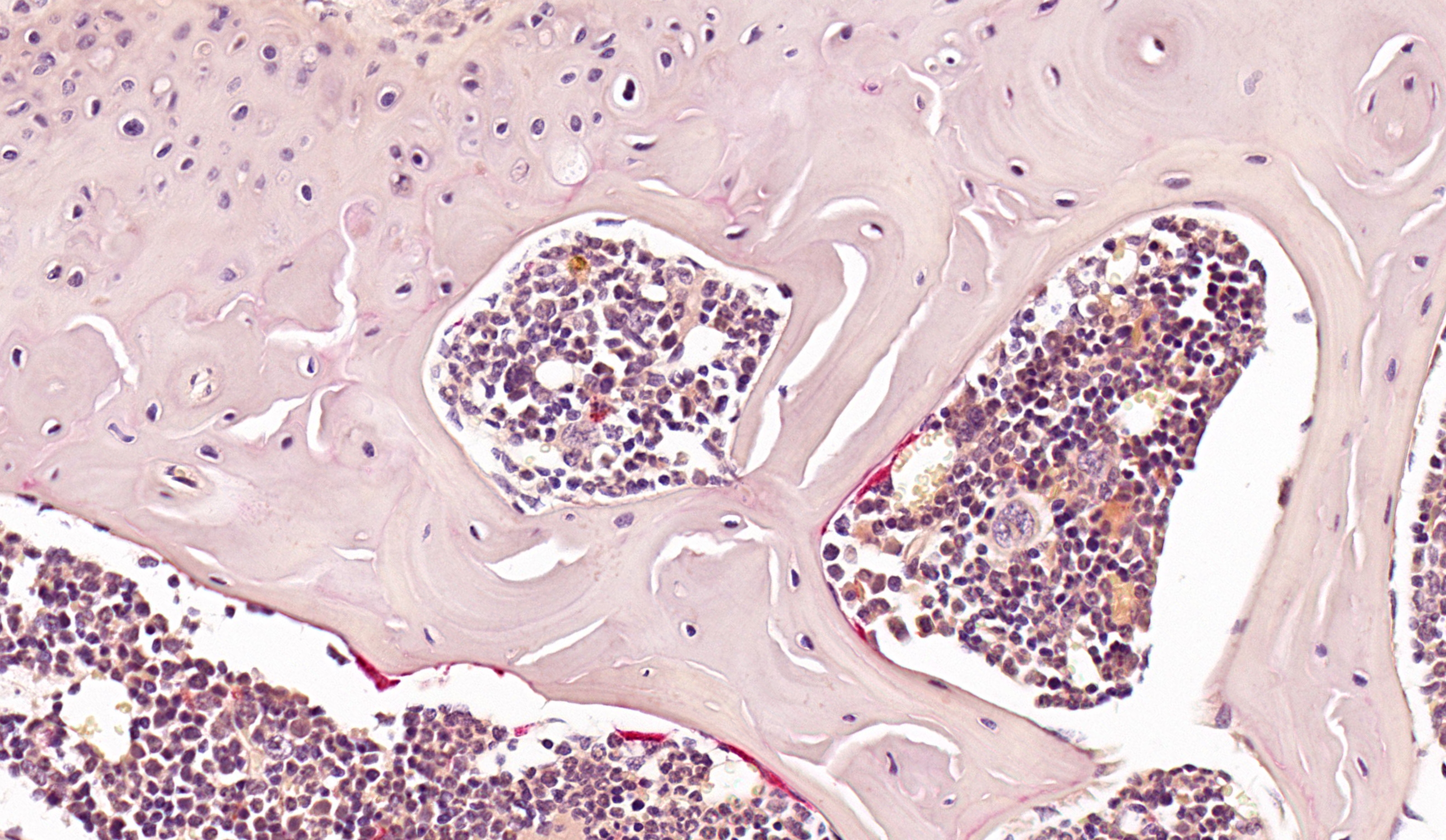

Supplement: Supplementary file 8 — Source data Fig. 4 [file 44321_2025_268_MOESM8_ESM.zip › Figure 4/4C/SHAM.jpg]

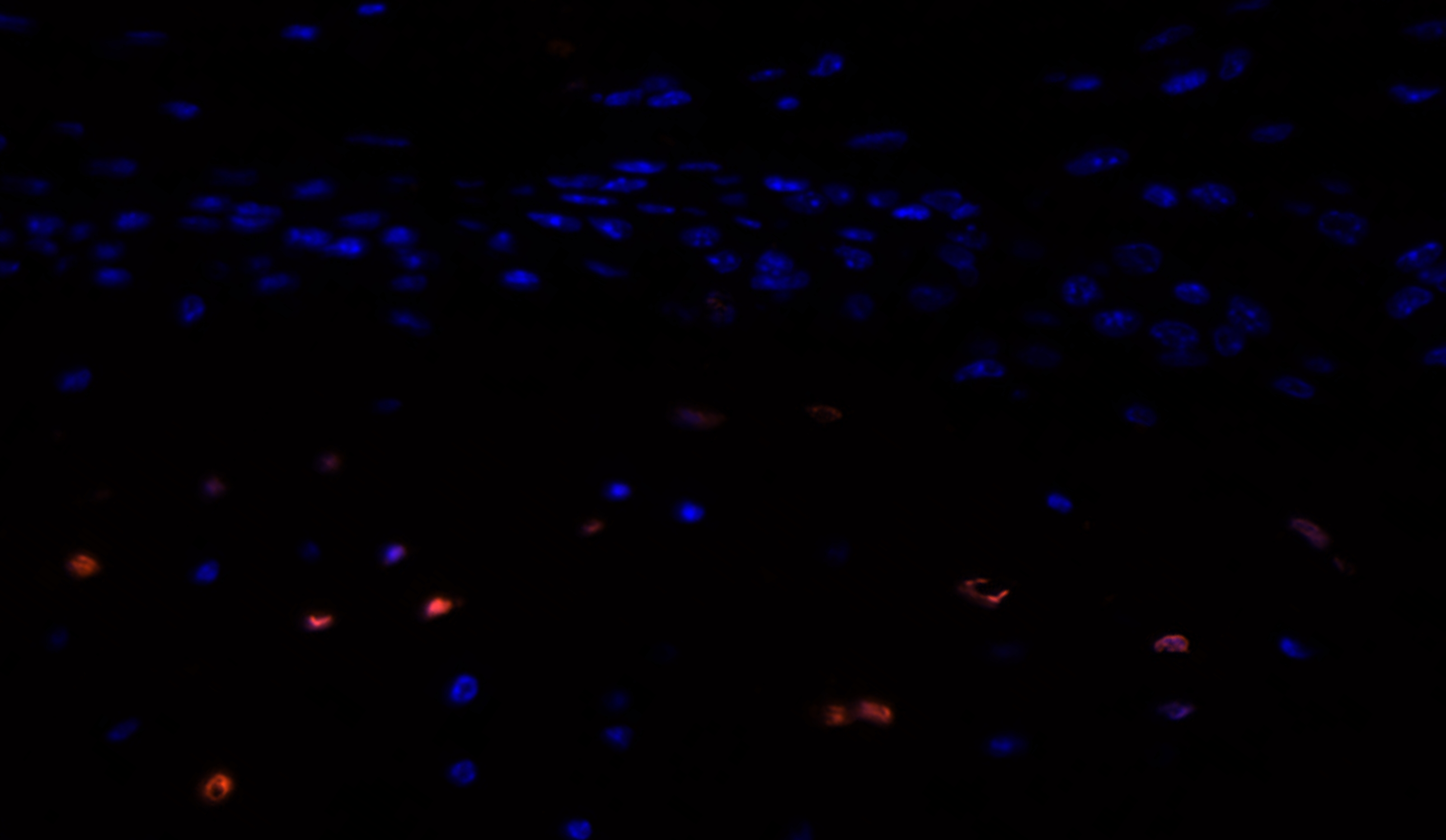

Supplement: Supplementary file 8 — Source data Fig. 4 [file 44321_2025_268_MOESM8_ESM.zip › Figure 4/4D/Apoe cko LTDMM.jpg]

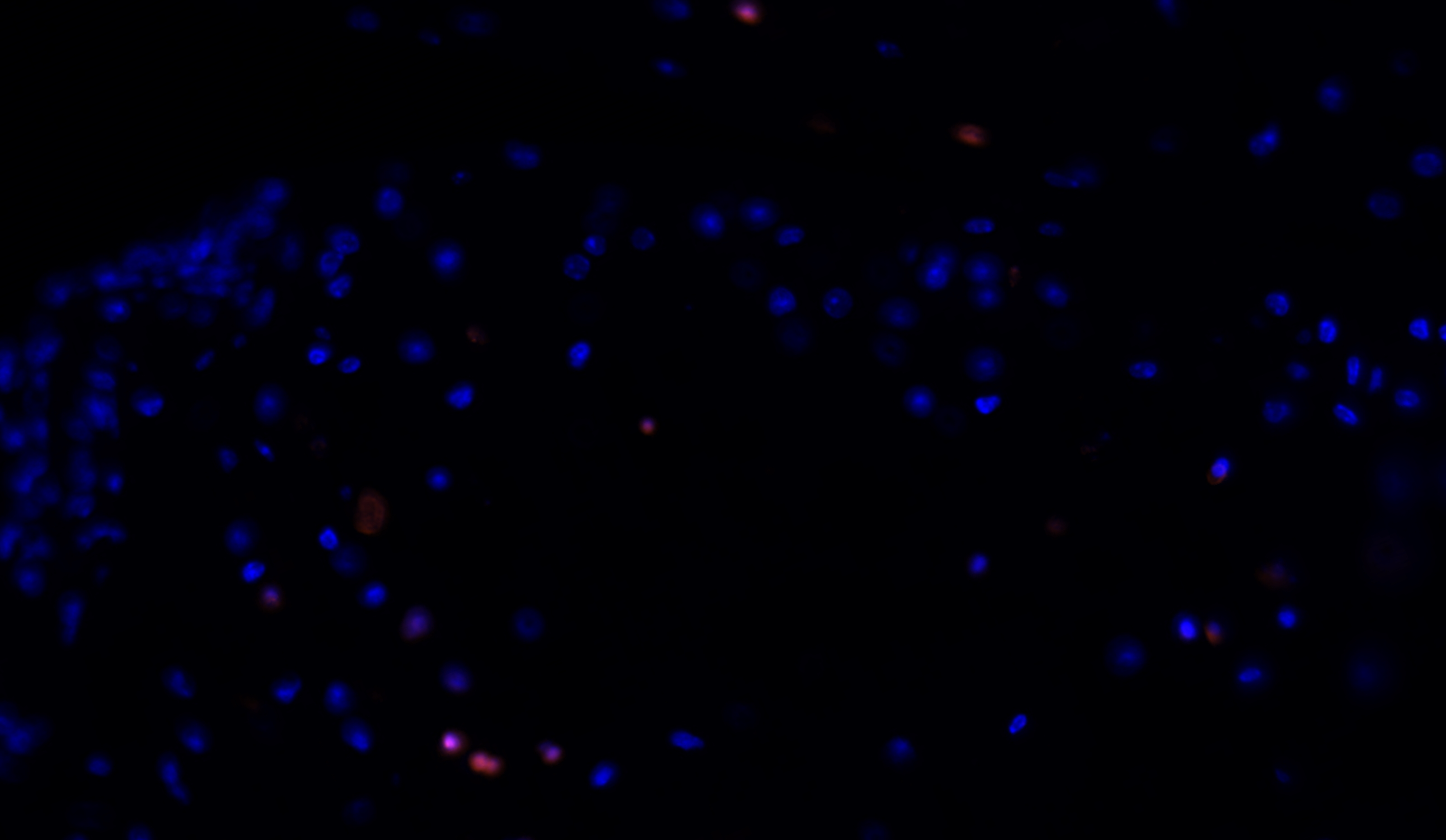

Supplement: Supplementary file 8 — Source data Fig. 4 [file 44321_2025_268_MOESM8_ESM.zip › Figure 4/4D/Apoe cko RTDMM.jpg]

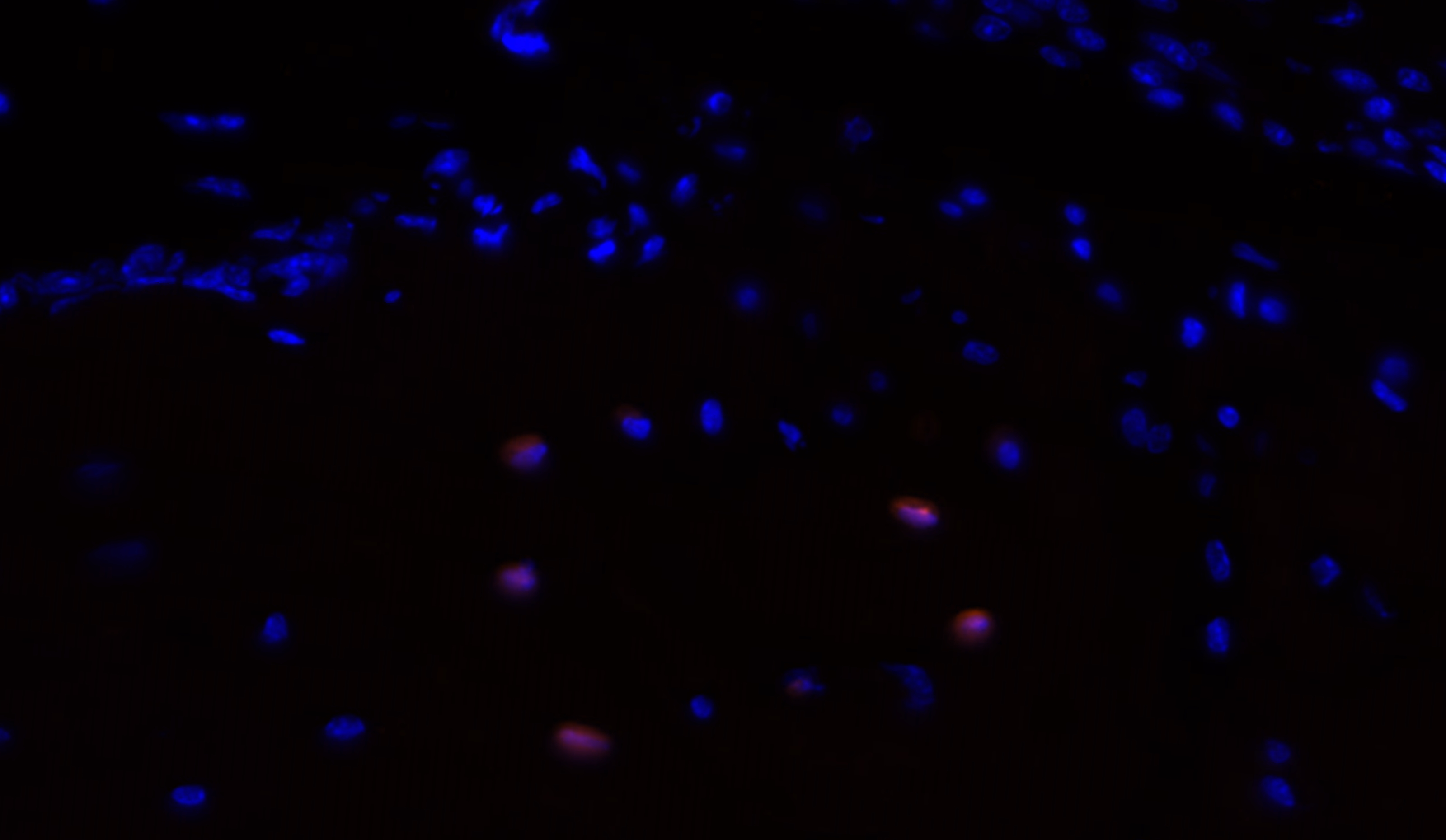

Supplement: Supplementary file 8 — Source data Fig. 4 [file 44321_2025_268_MOESM8_ESM.zip › Figure 4/4D/Apoe flox LTDMM.jpg]

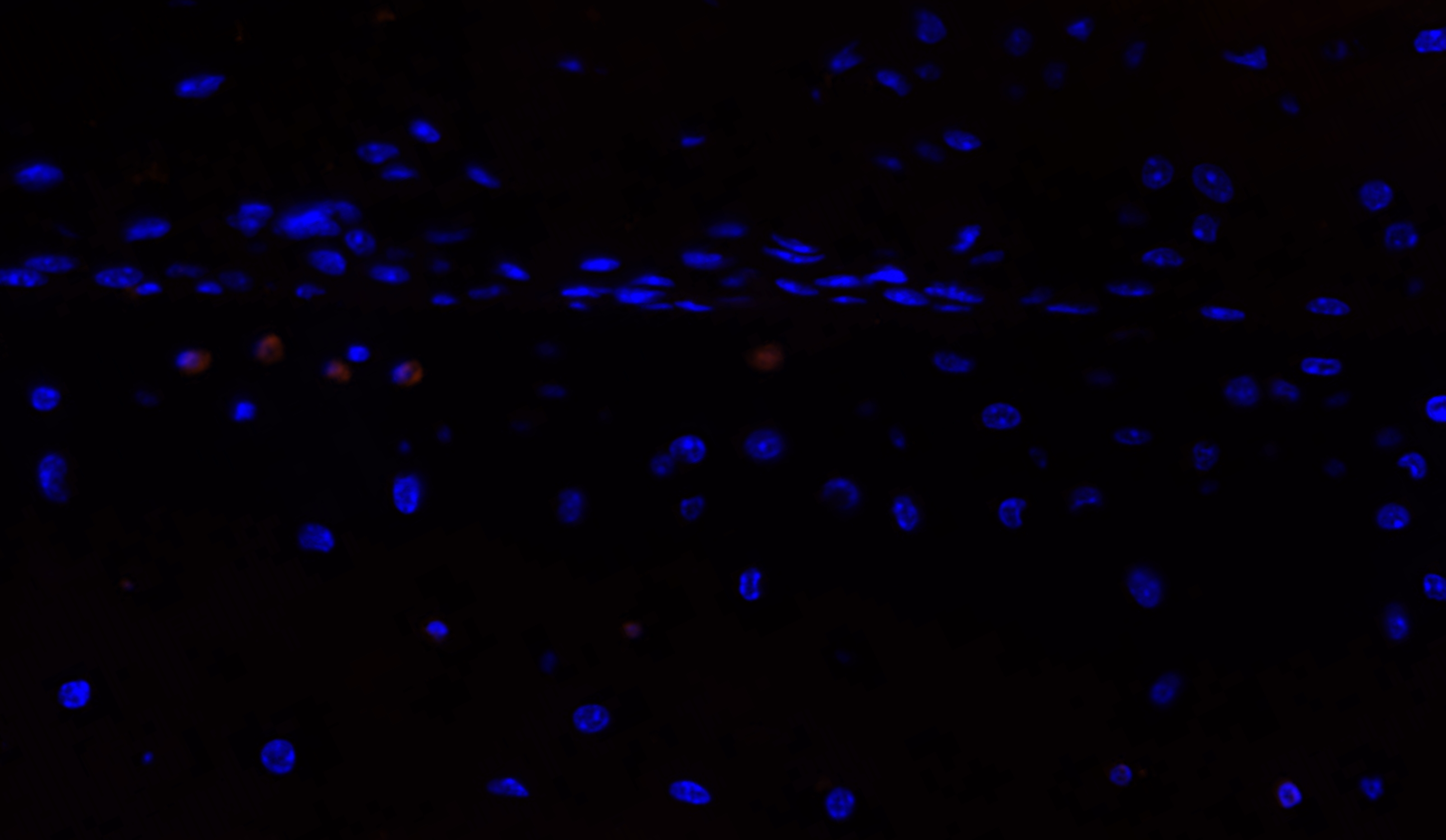

Supplement: Supplementary file 8 — Source data Fig. 4 [file 44321_2025_268_MOESM8_ESM.zip › Figure 4/4D/Apoe flox RTDMM.jpg]

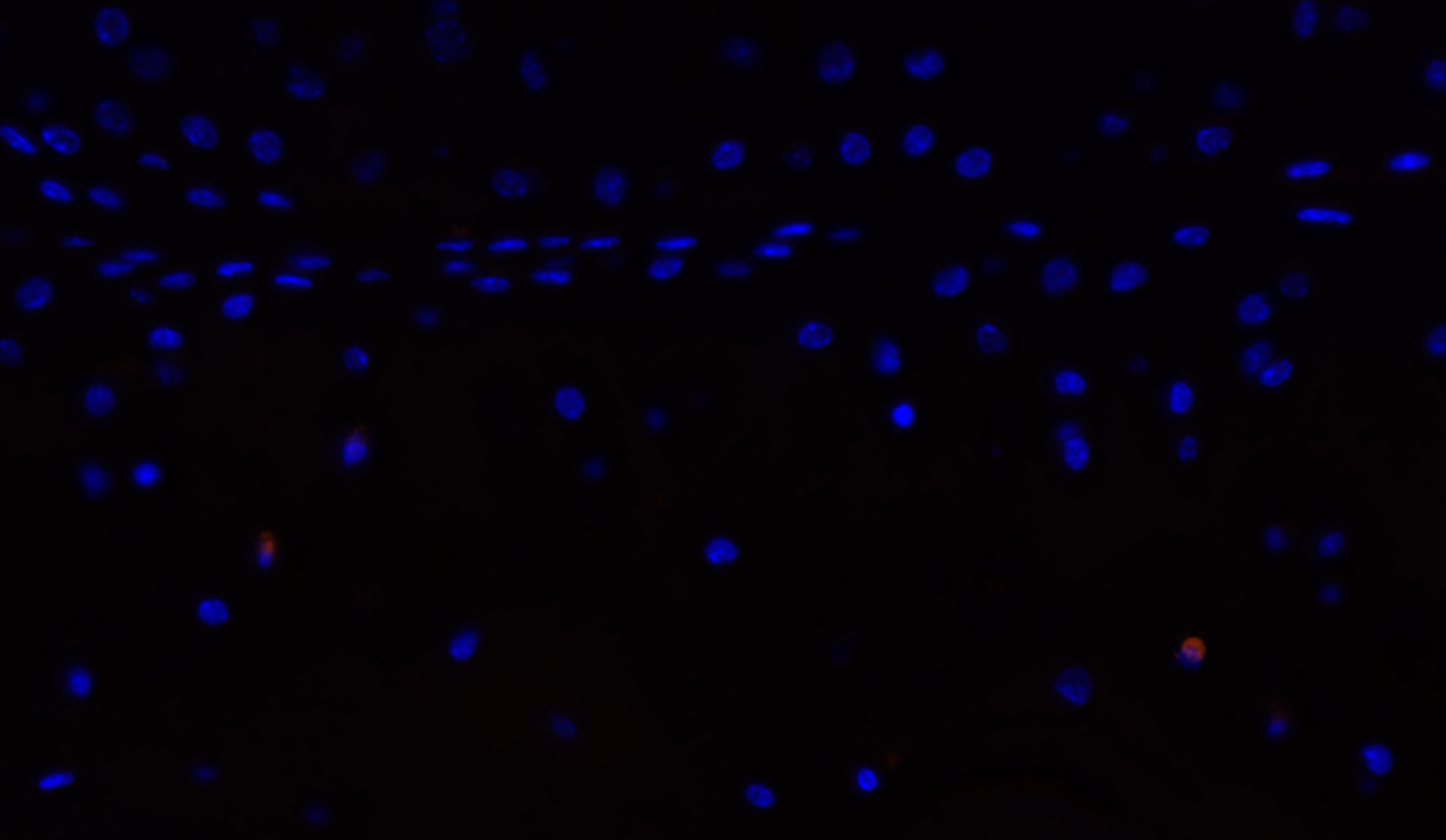

Supplement: Supplementary file 8 — Source data Fig. 4 [file 44321_2025_268_MOESM8_ESM.zip › Figure 4/4D/HFD.jpg]

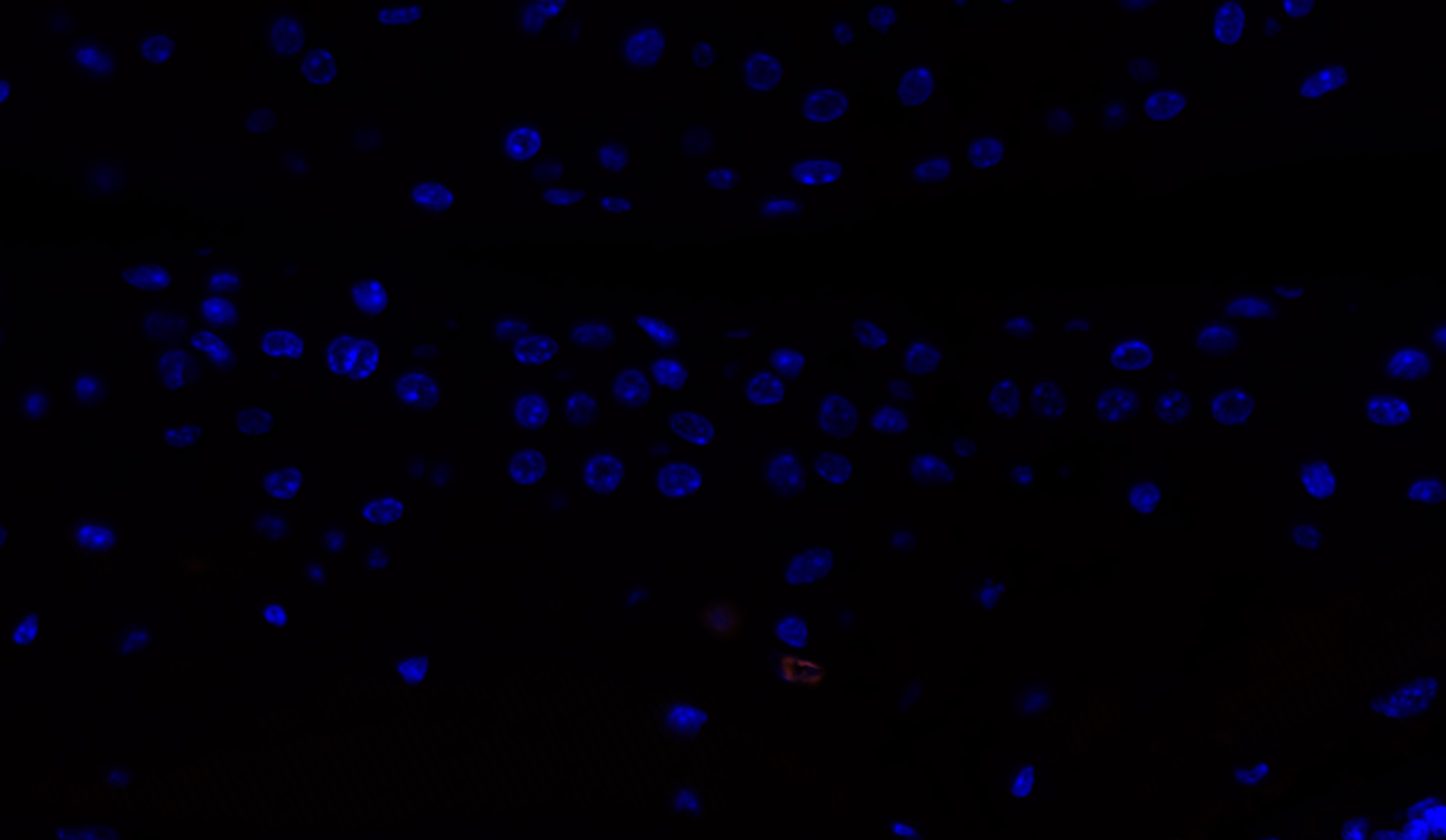

Supplement: Supplementary file 8 — Source data Fig. 4 [file 44321_2025_268_MOESM8_ESM.zip › Figure 4/4D/SHAM.jpg]

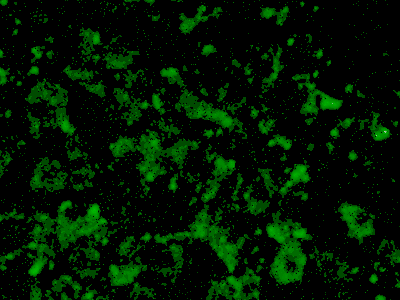

Supplement: Supplementary file 8 — Source data Fig. 4 [file 44321_2025_268_MOESM8_ESM.zip › Figure 4/4I/Apoe cko 33 bodipy.tif]

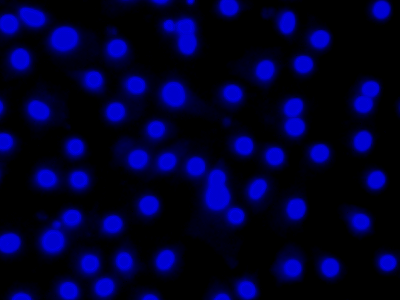

Supplement: Supplementary file 8 — Source data Fig. 4 [file 44321_2025_268_MOESM8_ESM.zip › Figure 4/4I/Apoe cko 33 dapi.tif]

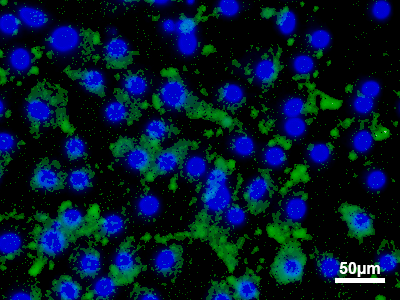

Supplement: Supplementary file 8 — Source data Fig. 4 [file 44321_2025_268_MOESM8_ESM.zip › Figure 4/4I/Apoe cko 33 merge.tif]

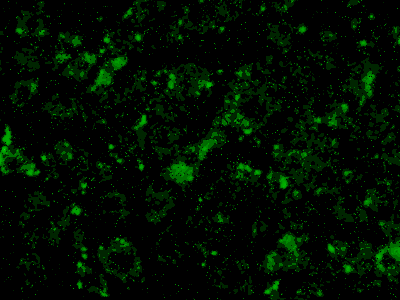

Supplement: Supplementary file 8 — Source data Fig. 4 [file 44321_2025_268_MOESM8_ESM.zip › Figure 4/4I/Apoe cko 37 bodipy.tif]

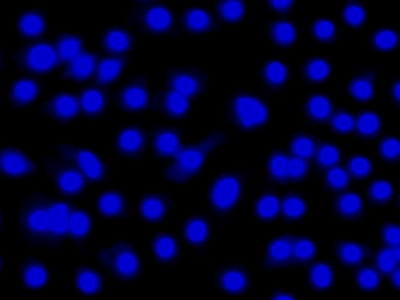

Supplement: Supplementary file 8 — Source data Fig. 4 [file 44321_2025_268_MOESM8_ESM.zip › Figure 4/4I/Apoe cko 37 dapi.tif]

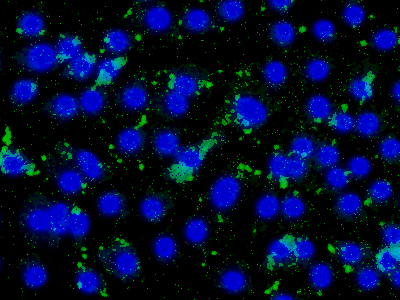

Supplement: Supplementary file 8 — Source data Fig. 4 [file 44321_2025_268_MOESM8_ESM.zip › Figure 4/4I/Apoe cko 37 merge.tif]

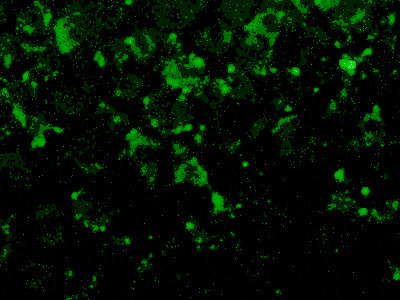

Supplement: Supplementary file 8 — Source data Fig. 4 [file 44321_2025_268_MOESM8_ESM.zip › Figure 4/4I/Apoe flox 33 bodipy.tif]

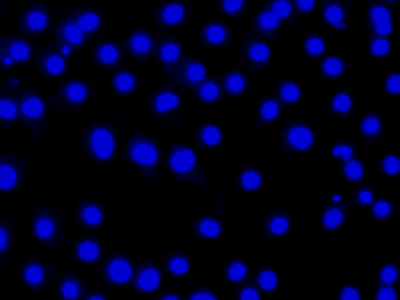

Supplement: Supplementary file 8 — Source data Fig. 4 [file 44321_2025_268_MOESM8_ESM.zip › Figure 4/4I/Apoe flox 33 dapi.tif]

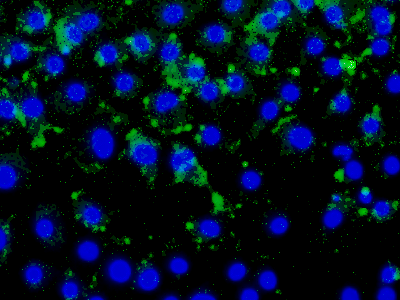

Supplement: Supplementary file 8 — Source data Fig. 4 [file 44321_2025_268_MOESM8_ESM.zip › Figure 4/4I/Apoe flox 33 merge.tif]

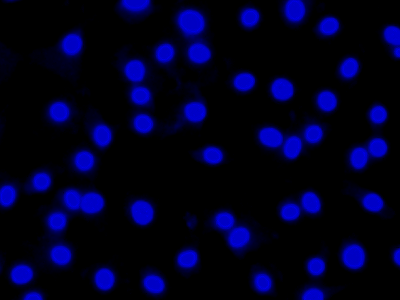

Supplement: Supplementary file 8 — Source data Fig. 4 [file 44321_2025_268_MOESM8_ESM.zip › Figure 4/4I/Apoe flox 37 dapi.tif]
