# Supplementary figures and images for: Cold exposure promotes the progression of osteoarthritis through downregulating APOE in cartilage (part 2 of 3)
Source: EMBO Mol Med. 2025 Jul 15;17(8):2137–62. doi: 10.1038/s44321-025-00268-6 (PMC12340072; doi:10.1038/s44321-025-00268-6)

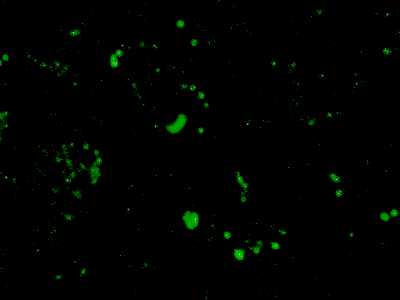

Supplement: Supplementary file 8 — Source data Fig. 4 [file 44321_2025_268_MOESM8_ESM.zip › Figure 4/4I/Apoe flox 37 bodipy.tif]

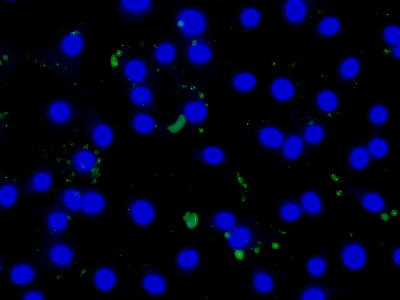

Supplement: Supplementary file 8 — Source data Fig. 4 [file 44321_2025_268_MOESM8_ESM.zip › Figure 4/4I/Apoe flox 37 merge.tif]

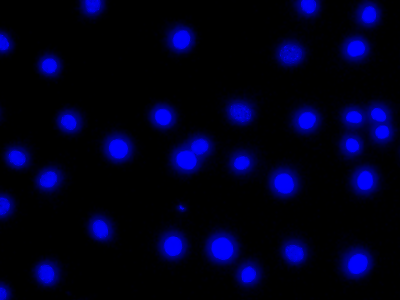

Supplement: Supplementary file 8 — Source data Fig. 4 [file 44321_2025_268_MOESM8_ESM.zip › Figure 4/4J/Apoe cko 33 hoechst.tif]

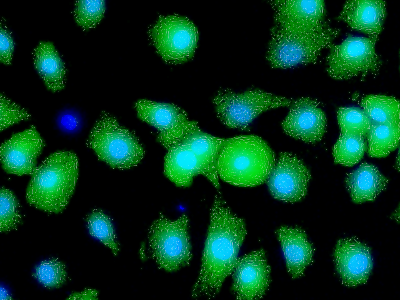

Supplement: Supplementary file 8 — Source data Fig. 4 [file 44321_2025_268_MOESM8_ESM.zip › Figure 4/4J/Apoe cko 33 merge.tif]

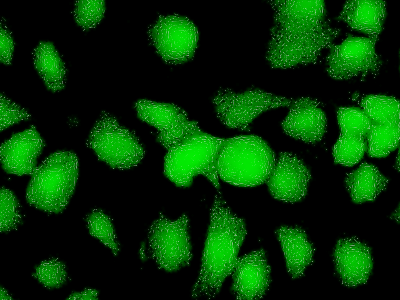

Supplement: Supplementary file 8 — Source data Fig. 4 [file 44321_2025_268_MOESM8_ESM.zip › Figure 4/4J/Apoe cko 33 ROS.tif]

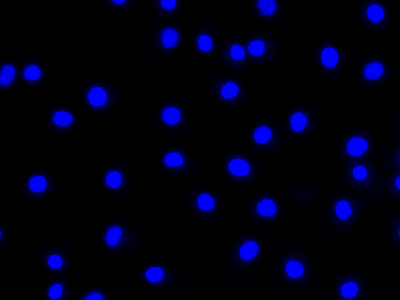

Supplement: Supplementary file 8 — Source data Fig. 4 [file 44321_2025_268_MOESM8_ESM.zip › Figure 4/4J/Apoe cko 37 hoechst.tif]

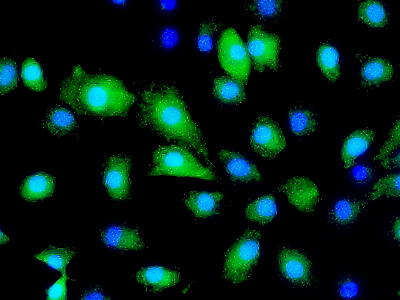

Supplement: Supplementary file 8 — Source data Fig. 4 [file 44321_2025_268_MOESM8_ESM.zip › Figure 4/4J/Apoe cko 37 merge.tif]

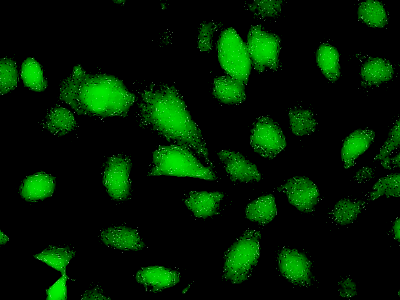

Supplement: Supplementary file 8 — Source data Fig. 4 [file 44321_2025_268_MOESM8_ESM.zip › Figure 4/4J/Apoe cko 37 ROS.tif]

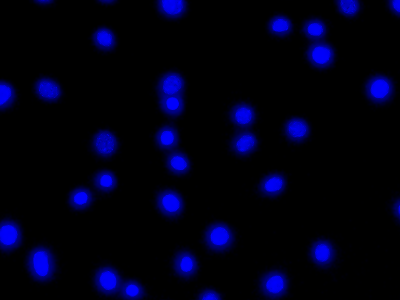

Supplement: Supplementary file 8 — Source data Fig. 4 [file 44321_2025_268_MOESM8_ESM.zip › Figure 4/4J/Apoe flox 33 hoechst.tif]

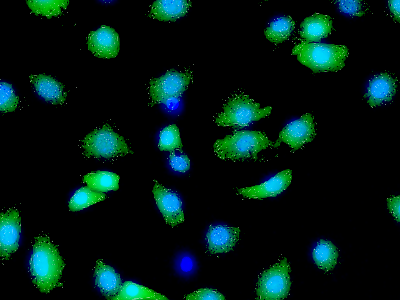

Supplement: Supplementary file 8 — Source data Fig. 4 [file 44321_2025_268_MOESM8_ESM.zip › Figure 4/4J/Apoe flox 33 merge.tif]

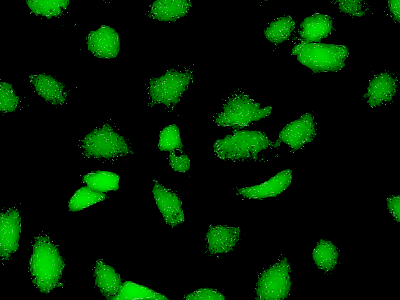

Supplement: Supplementary file 8 — Source data Fig. 4 [file 44321_2025_268_MOESM8_ESM.zip › Figure 4/4J/Apoe flox 33 ROS.tif]

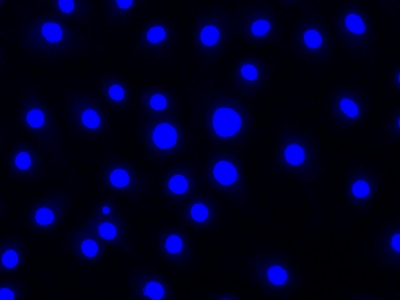

Supplement: Supplementary file 8 — Source data Fig. 4 [file 44321_2025_268_MOESM8_ESM.zip › Figure 4/4J/Apoe flox 37 hoechst.tif]

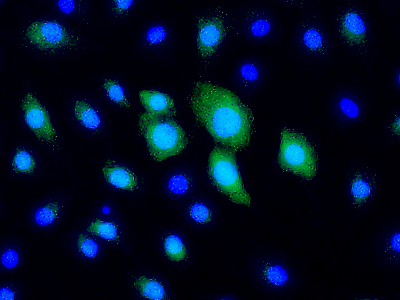

Supplement: Supplementary file 8 — Source data Fig. 4 [file 44321_2025_268_MOESM8_ESM.zip › Figure 4/4J/Apoe flox 37 merge.tif]

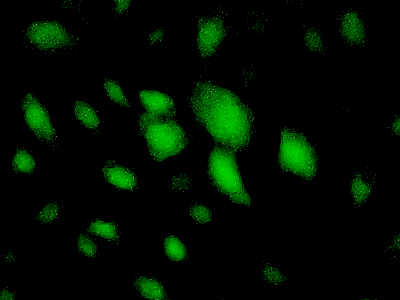

Supplement: Supplementary file 8 — Source data Fig. 4 [file 44321_2025_268_MOESM8_ESM.zip › Figure 4/4J/Apoe flox 37 ROS.tif]

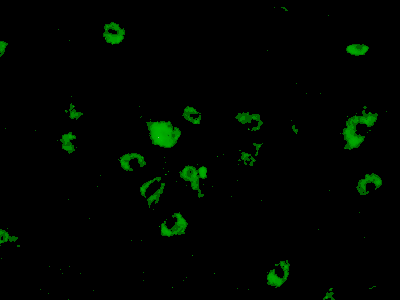

Supplement: Supplementary file 8 — Source data Fig. 4 [file 44321_2025_268_MOESM8_ESM.zip › Figure 4/4K/Apoe cko 33 green.tif]

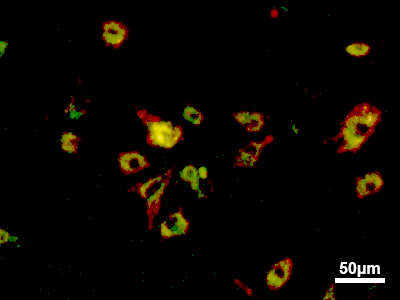

Supplement: Supplementary file 8 — Source data Fig. 4 [file 44321_2025_268_MOESM8_ESM.zip › Figure 4/4K/Apoe cko 33 merge.tif]

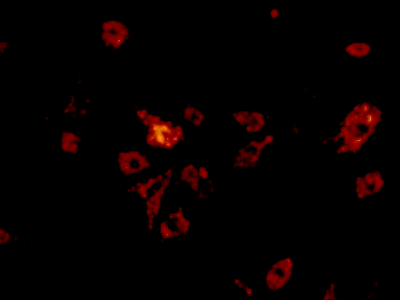

Supplement: Supplementary file 8 — Source data Fig. 4 [file 44321_2025_268_MOESM8_ESM.zip › Figure 4/4K/Apoe cko 33 red.tif]

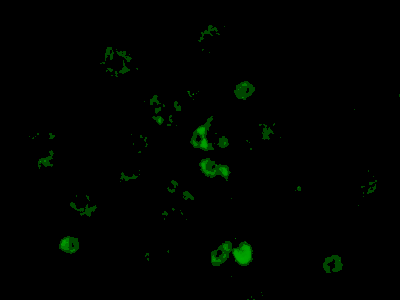

Supplement: Supplementary file 8 — Source data Fig. 4 [file 44321_2025_268_MOESM8_ESM.zip › Figure 4/4K/Apoe cko 37 green.tif]

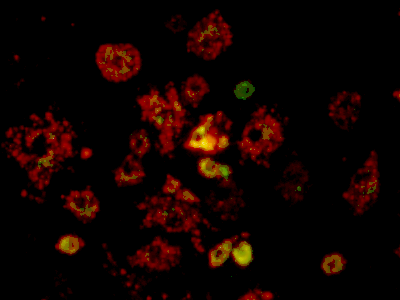

Supplement: Supplementary file 8 — Source data Fig. 4 [file 44321_2025_268_MOESM8_ESM.zip › Figure 4/4K/Apoe cko 37 merge.tif]

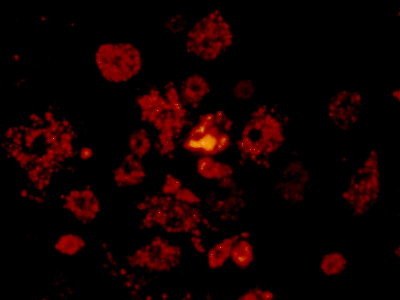

Supplement: Supplementary file 8 — Source data Fig. 4 [file 44321_2025_268_MOESM8_ESM.zip › Figure 4/4K/Apoe cko 37 red.tif]

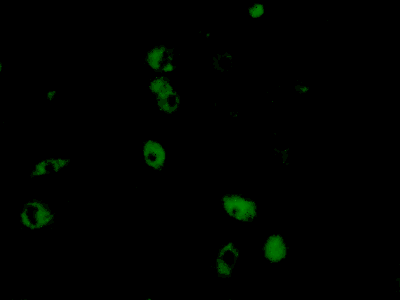

Supplement: Supplementary file 8 — Source data Fig. 4 [file 44321_2025_268_MOESM8_ESM.zip › Figure 4/4K/Apoe flox 33 green.tif]

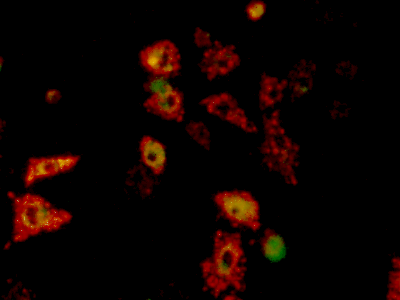

Supplement: Supplementary file 8 — Source data Fig. 4 [file 44321_2025_268_MOESM8_ESM.zip › Figure 4/4K/Apoe flox 33 merge.tif]

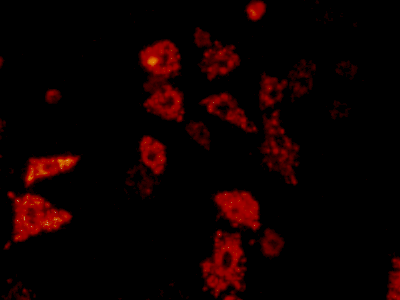

Supplement: Supplementary file 8 — Source data Fig. 4 [file 44321_2025_268_MOESM8_ESM.zip › Figure 4/4K/Apoe flox 33 red.tif]

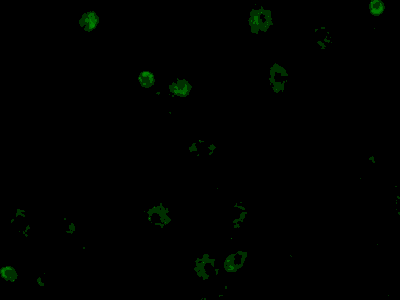

Supplement: Supplementary file 8 — Source data Fig. 4 [file 44321_2025_268_MOESM8_ESM.zip › Figure 4/4K/Apoe flox 37 green.tif]

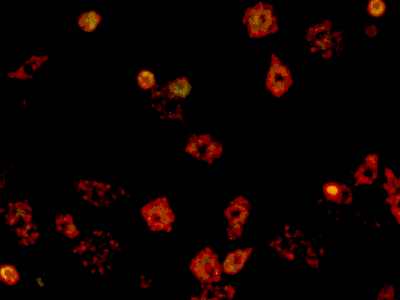

Supplement: Supplementary file 8 — Source data Fig. 4 [file 44321_2025_268_MOESM8_ESM.zip › Figure 4/4K/Apoe flox 37 merge.tif]

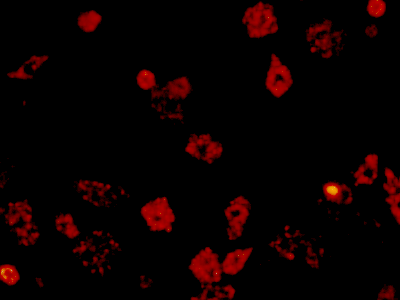

Supplement: Supplementary file 8 — Source data Fig. 4 [file 44321_2025_268_MOESM8_ESM.zip › Figure 4/4K/Apoe flox 37 red.tif]

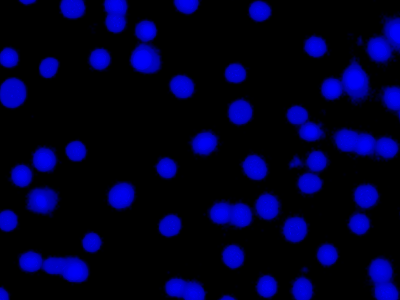

Supplement: Supplementary file 8 — Source data Fig. 4 [file 44321_2025_268_MOESM8_ESM.zip › Figure 4/4L/Apoe cko 33 dapi.tif]

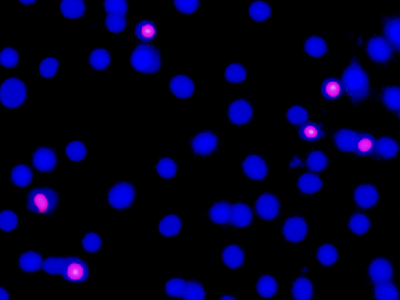

Supplement: Supplementary file 8 — Source data Fig. 4 [file 44321_2025_268_MOESM8_ESM.zip › Figure 4/4L/Apoe cko 33 merge.tif]

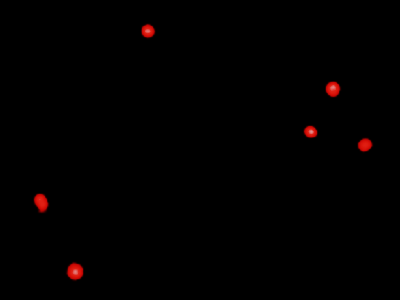

Supplement: Supplementary file 8 — Source data Fig. 4 [file 44321_2025_268_MOESM8_ESM.zip › Figure 4/4L/Apoe cko 33 tunel.tif]

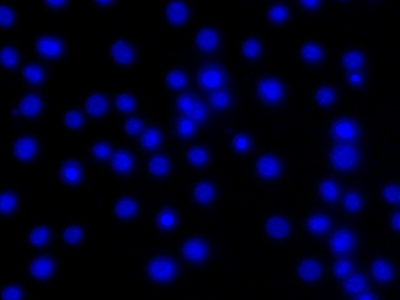

Supplement: Supplementary file 8 — Source data Fig. 4 [file 44321_2025_268_MOESM8_ESM.zip › Figure 4/4L/Apoe cko 37 dapi.tif]

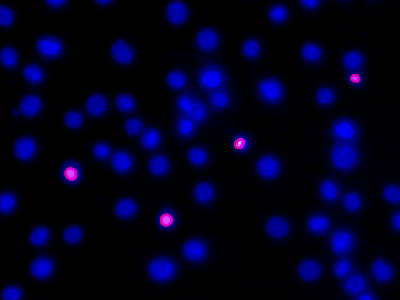

Supplement: Supplementary file 8 — Source data Fig. 4 [file 44321_2025_268_MOESM8_ESM.zip › Figure 4/4L/Apoe cko 37 merge.tif]

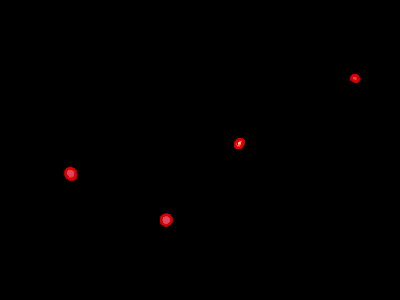

Supplement: Supplementary file 8 — Source data Fig. 4 [file 44321_2025_268_MOESM8_ESM.zip › Figure 4/4L/Apoe cko 37 tuenl.tif]

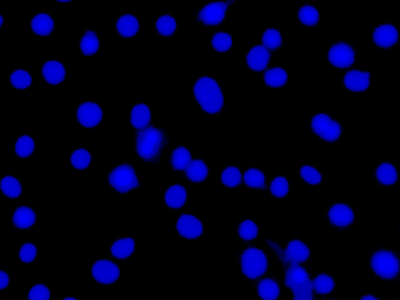

Supplement: Supplementary file 8 — Source data Fig. 4 [file 44321_2025_268_MOESM8_ESM.zip › Figure 4/4L/Apoe flox 33 dapi.tif]

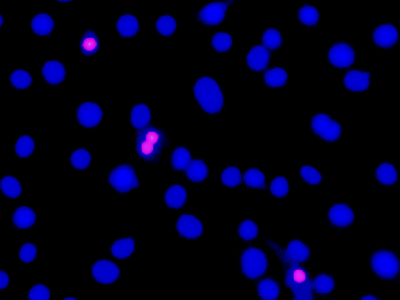

Supplement: Supplementary file 8 — Source data Fig. 4 [file 44321_2025_268_MOESM8_ESM.zip › Figure 4/4L/Apoe flox 33 merge.tif]

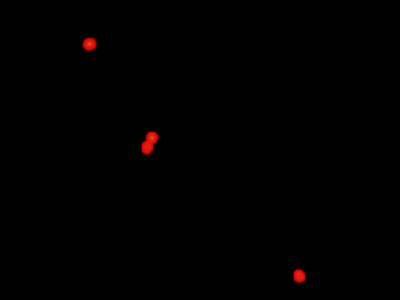

Supplement: Supplementary file 8 — Source data Fig. 4 [file 44321_2025_268_MOESM8_ESM.zip › Figure 4/4L/Apoe flox 33 tunel.tif]

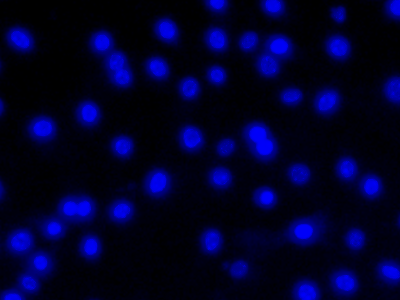

Supplement: Supplementary file 8 — Source data Fig. 4 [file 44321_2025_268_MOESM8_ESM.zip › Figure 4/4L/Apoe flox 37 dapi.tif]

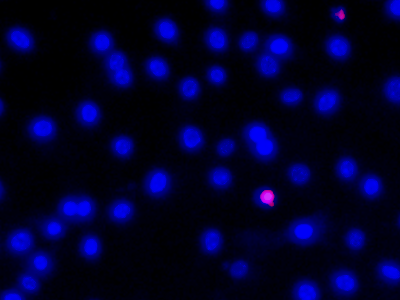

Supplement: Supplementary file 8 — Source data Fig. 4 [file 44321_2025_268_MOESM8_ESM.zip › Figure 4/4L/Apoe flox 37 merge.tif]

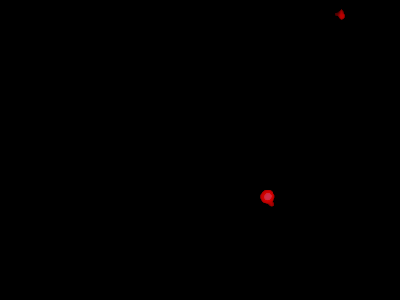

Supplement: Supplementary file 8 — Source data Fig. 4 [file 44321_2025_268_MOESM8_ESM.zip › Figure 4/4L/Apoe flox 37 tunel.tif]

Figure 5A

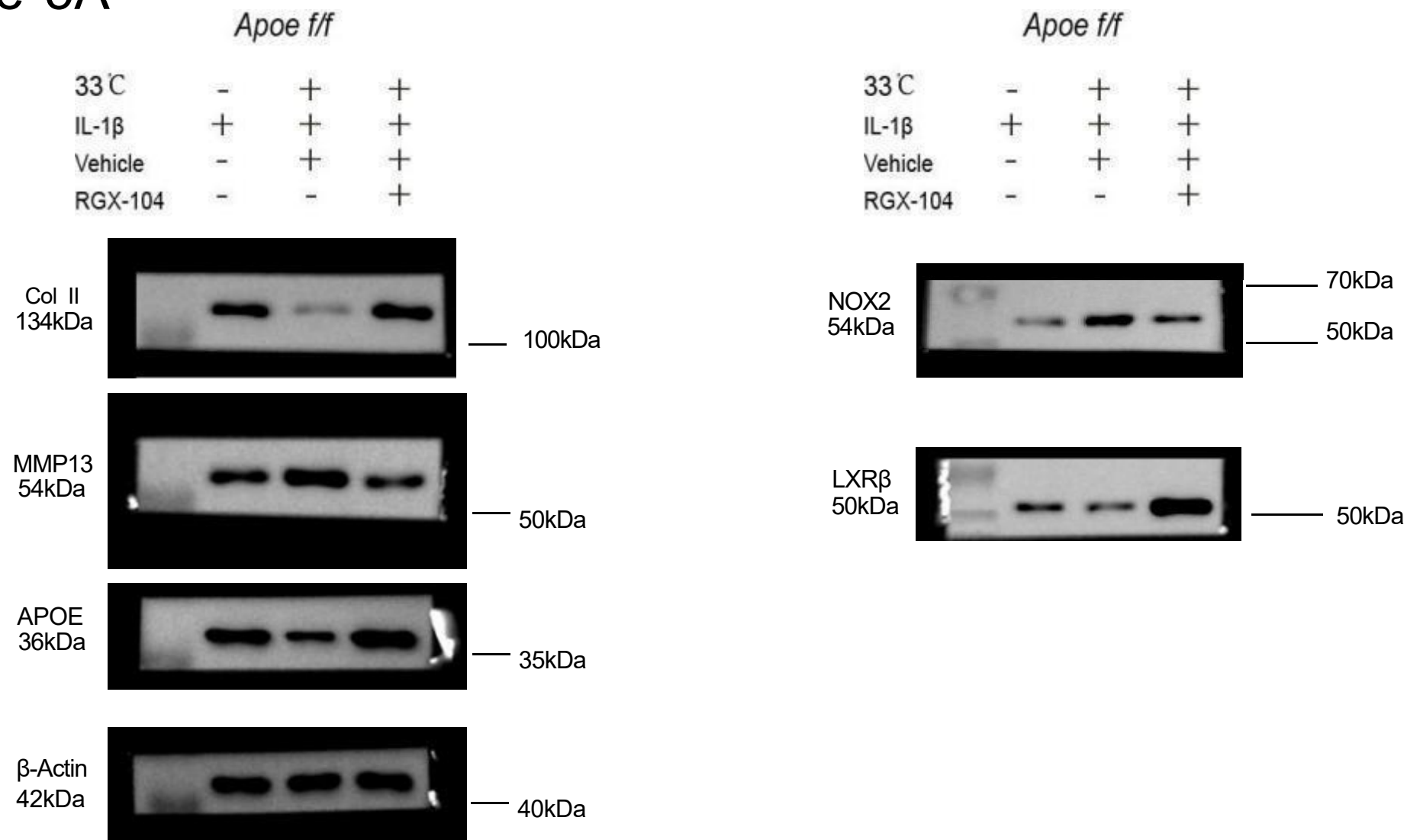

Supplement: Supplementary file 9 — Source data Fig. 5 [file 44321_2025_268_MOESM9_ESM.zip › Figure 5/5A/5A.pdf]

Figure 5B

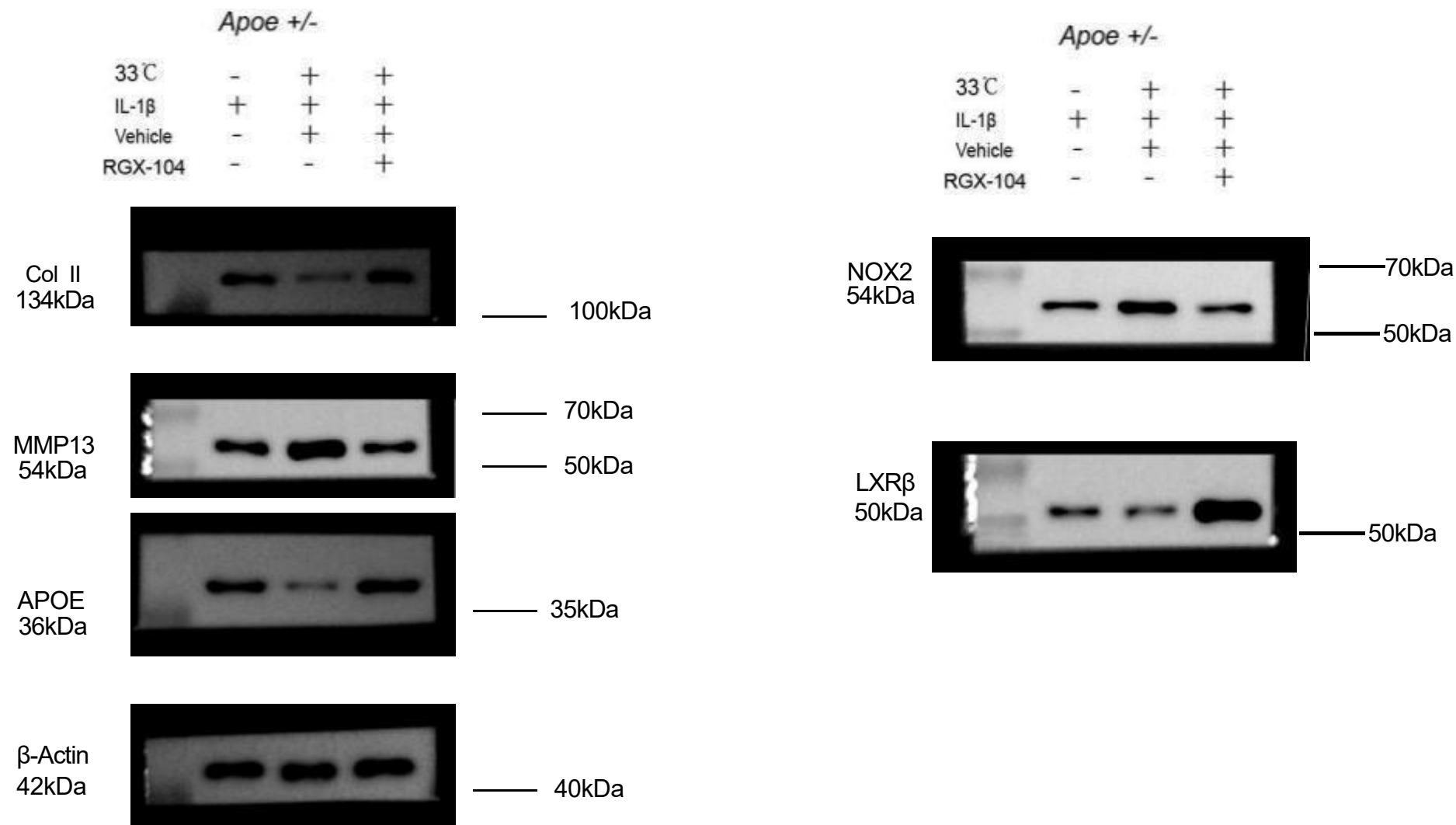

Supplement: Supplementary file 9 — Source data Fig. 5 [file 44321_2025_268_MOESM9_ESM.zip › Figure 5/5B/5B.pdf]

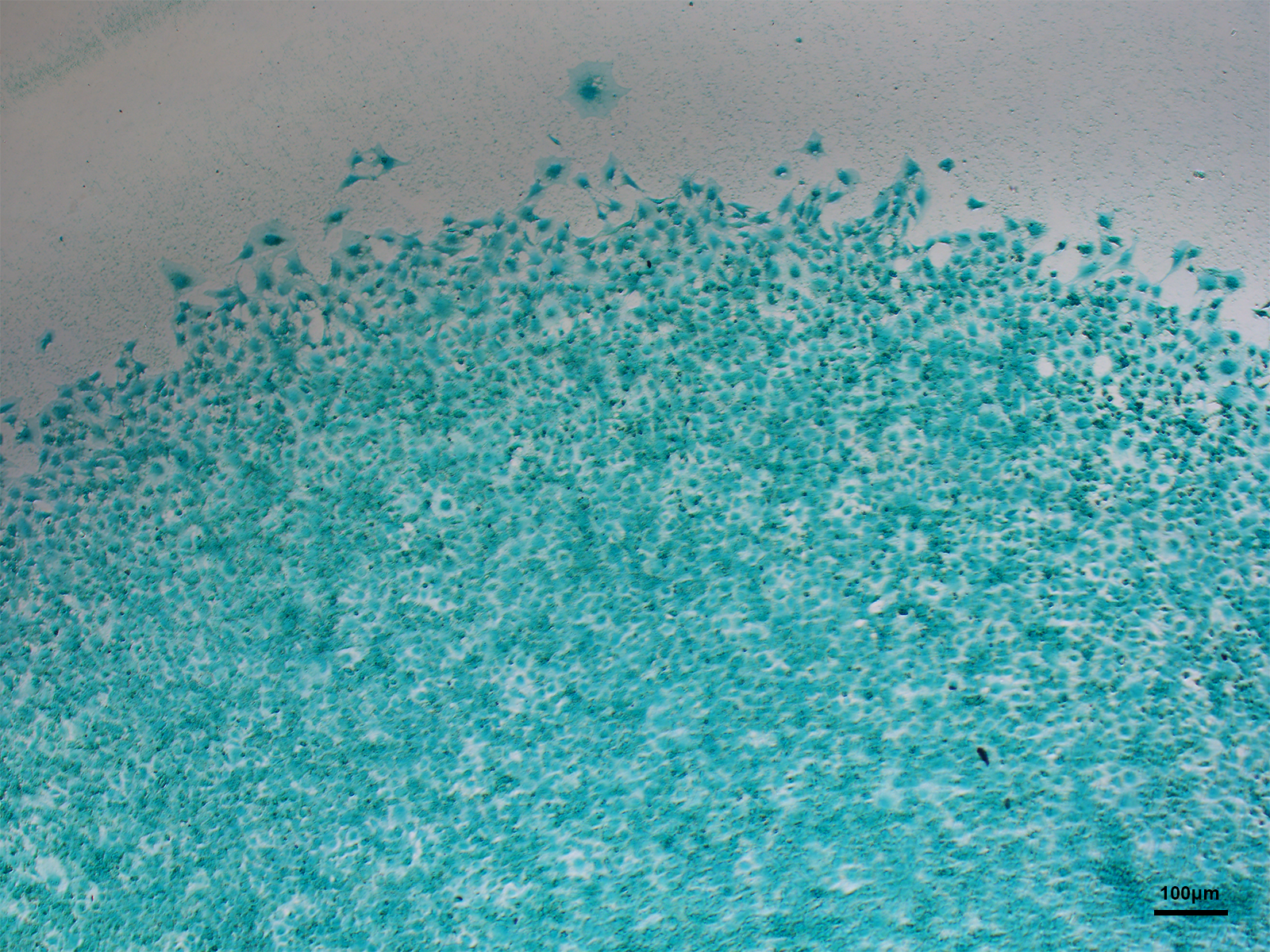

Supplement: Supplementary file 9 — Source data Fig. 5 [file 44321_2025_268_MOESM9_ESM.zip › Figure 5/5E/Apoe +-/IL-1+33+RGX-104.tif]

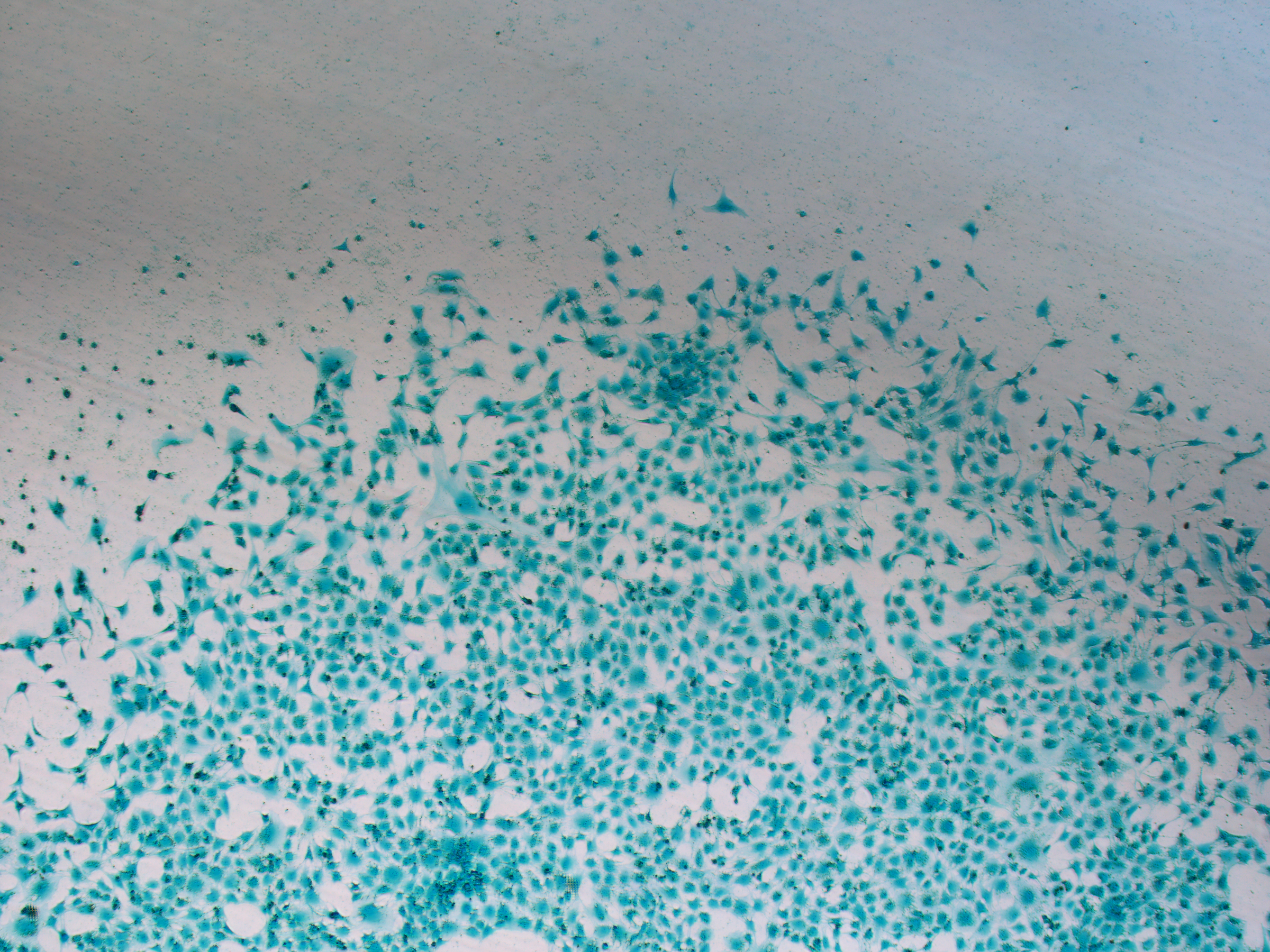

Supplement: Supplementary file 9 — Source data Fig. 5 [file 44321_2025_268_MOESM9_ESM.zip › Figure 5/5E/Apoe +-/IL-1+33.tif]

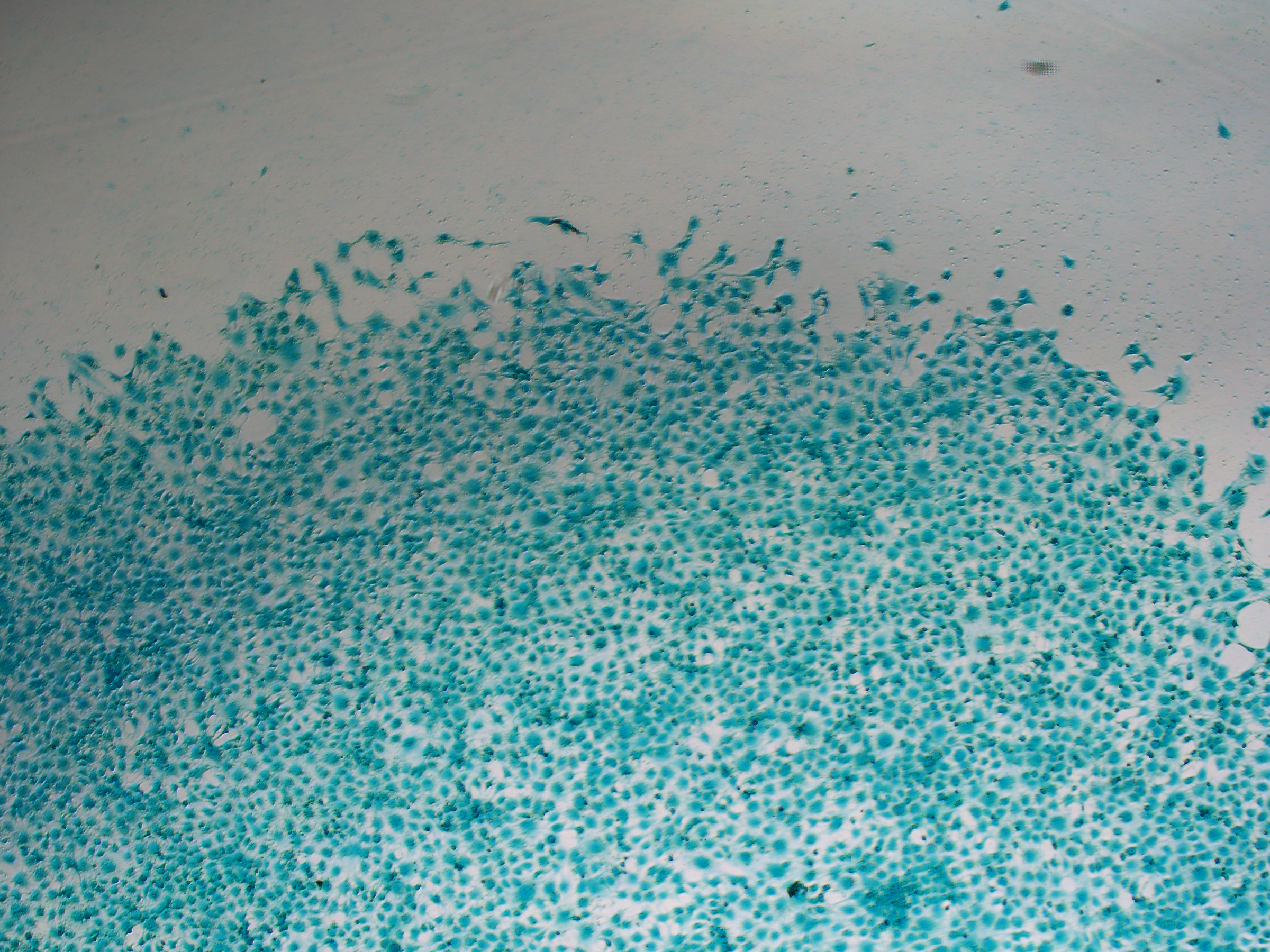

Supplement: Supplementary file 9 — Source data Fig. 5 [file 44321_2025_268_MOESM9_ESM.zip › Figure 5/5E/Apoe +-/IL-1.tif]

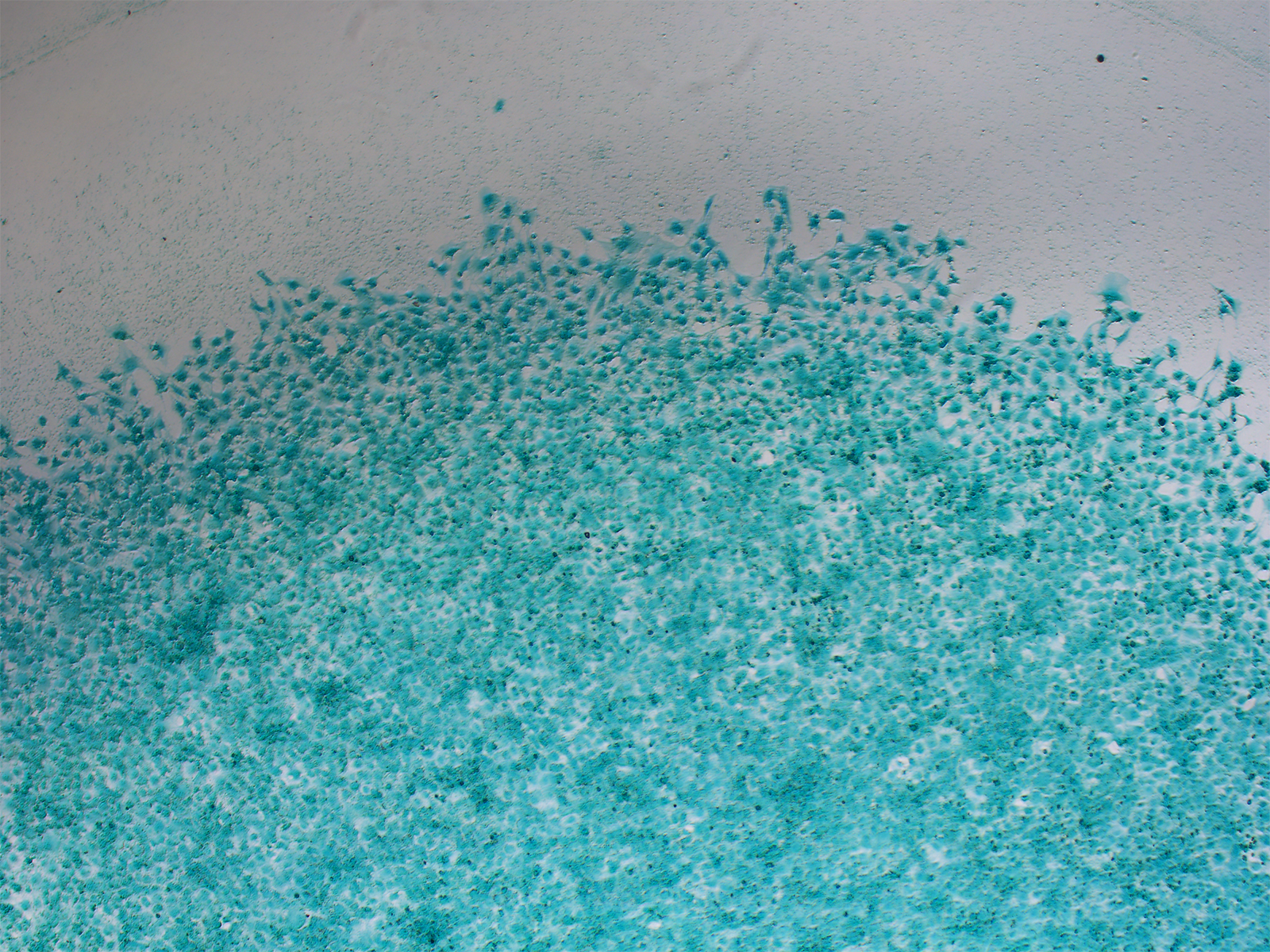

Supplement: Supplementary file 9 — Source data Fig. 5 [file 44321_2025_268_MOESM9_ESM.zip › Figure 5/5E/Apoe flox/IL-1+33+RGX-104.tif]

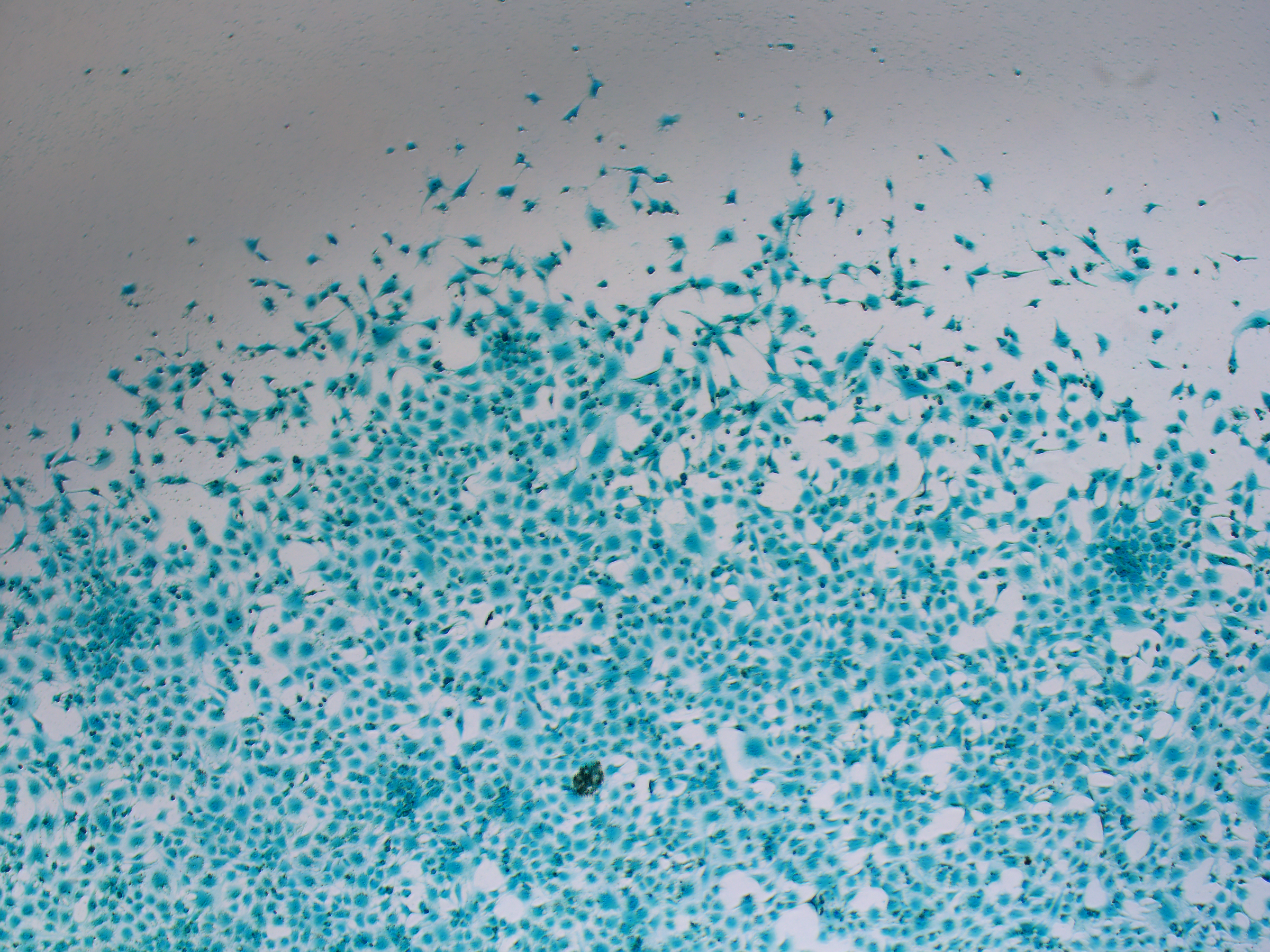

Supplement: Supplementary file 9 — Source data Fig. 5 [file 44321_2025_268_MOESM9_ESM.zip › Figure 5/5E/Apoe flox/IL-1+33.tif]

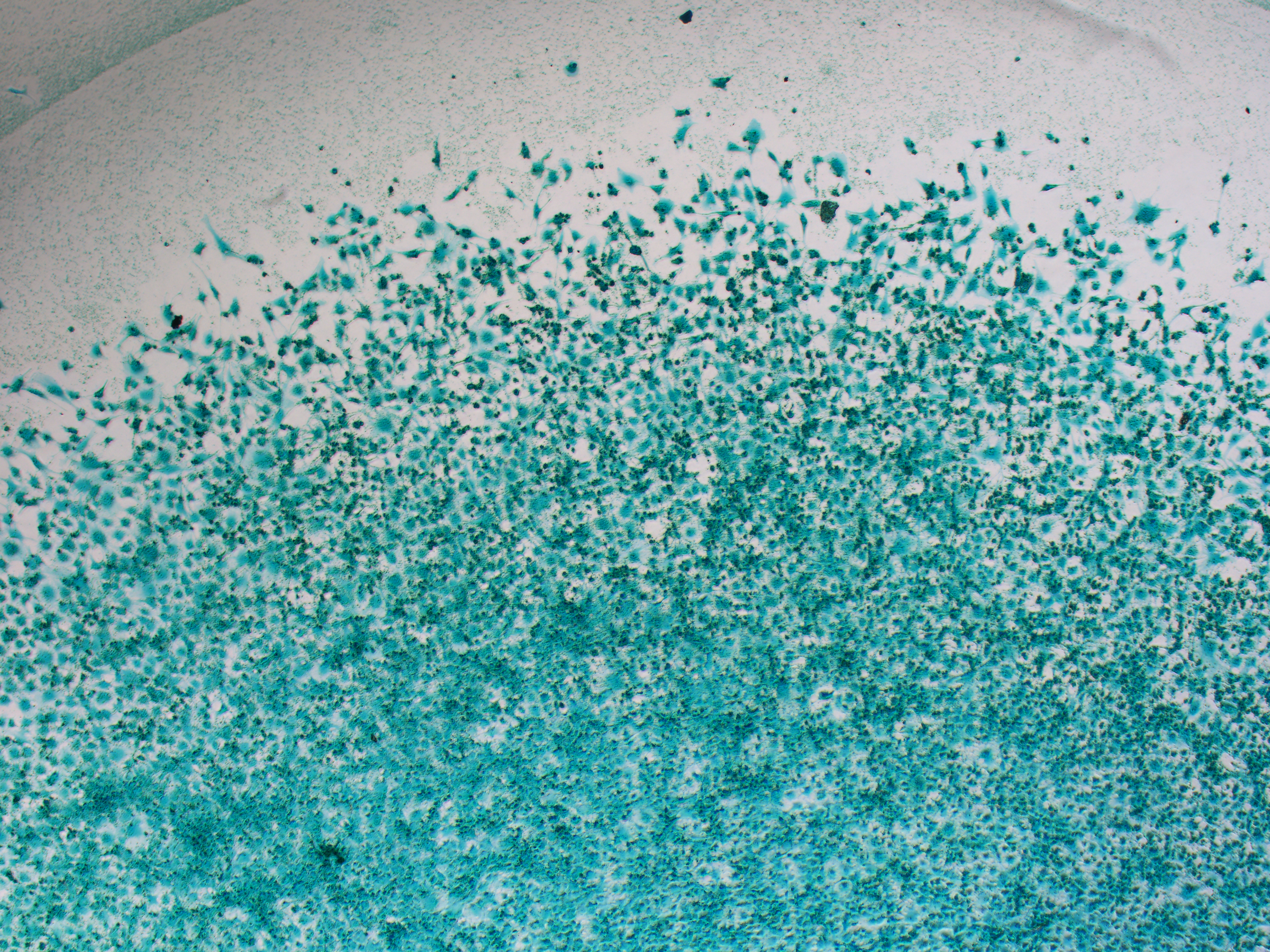

Supplement: Supplementary file 9 — Source data Fig. 5 [file 44321_2025_268_MOESM9_ESM.zip › Figure 5/5E/Apoe flox/IL-1.tif]

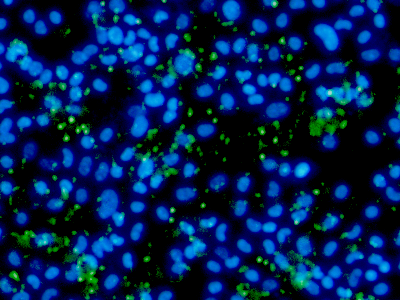

Supplement: Supplementary file 9 — Source data Fig. 5 [file 44321_2025_268_MOESM9_ESM.zip › Figure 5/5F/Apoe +-/IL-1+33+RGX.tif]

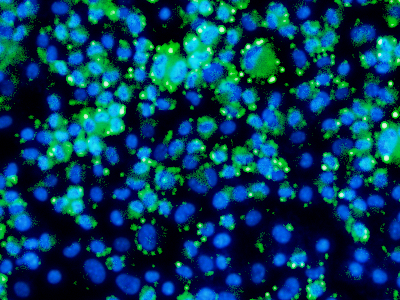

Supplement: Supplementary file 9 — Source data Fig. 5 [file 44321_2025_268_MOESM9_ESM.zip › Figure 5/5F/Apoe +-/IL-1+33.tif]

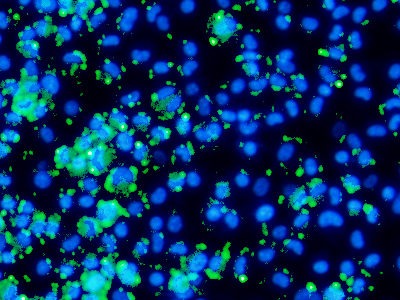

Supplement: Supplementary file 9 — Source data Fig. 5 [file 44321_2025_268_MOESM9_ESM.zip › Figure 5/5F/Apoe +-/IL-1.tif]

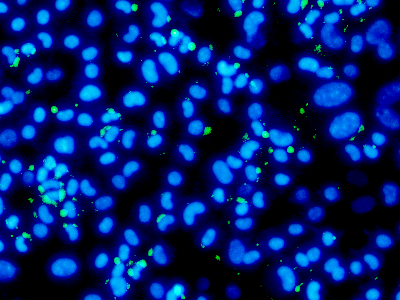

Supplement: Supplementary file 9 — Source data Fig. 5 [file 44321_2025_268_MOESM9_ESM.zip › Figure 5/5F/Apoe flox/IL-1+33+RGX.tif]

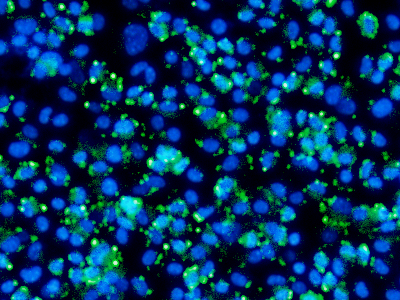

Supplement: Supplementary file 9 — Source data Fig. 5 [file 44321_2025_268_MOESM9_ESM.zip › Figure 5/5F/Apoe flox/IL-1+33.tif]

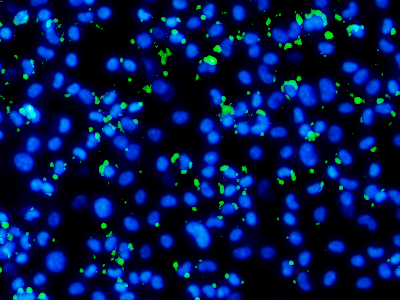

Supplement: Supplementary file 9 — Source data Fig. 5 [file 44321_2025_268_MOESM9_ESM.zip › Figure 5/5F/Apoe flox/IL-1.tif]

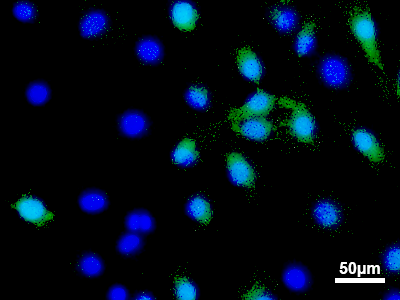

Supplement: Supplementary file 9 — Source data Fig. 5 [file 44321_2025_268_MOESM9_ESM.zip › Figure 5/5G/Apoe +-/IL-1+33+RGX-104.tif]

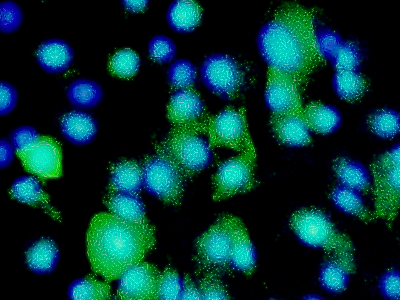

Supplement: Supplementary file 9 — Source data Fig. 5 [file 44321_2025_268_MOESM9_ESM.zip › Figure 5/5G/Apoe +-/IL-1+33.tif]

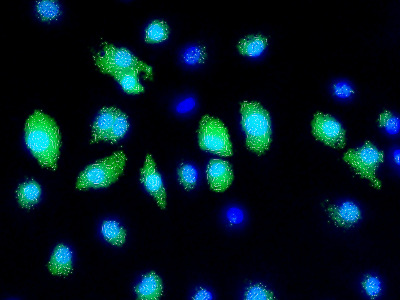

Supplement: Supplementary file 9 — Source data Fig. 5 [file 44321_2025_268_MOESM9_ESM.zip › Figure 5/5G/Apoe +-/IL-1.tif]

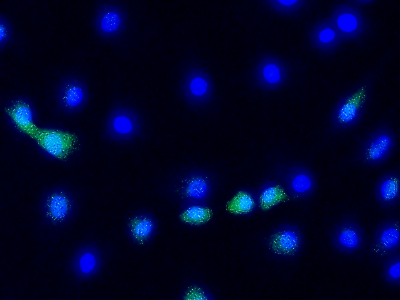

Supplement: Supplementary file 9 — Source data Fig. 5 [file 44321_2025_268_MOESM9_ESM.zip › Figure 5/5G/Apoe flox/IL-1+33+RGX-104.tif]

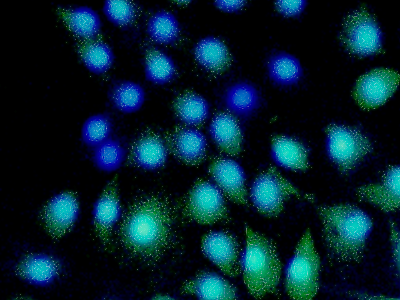

Supplement: Supplementary file 9 — Source data Fig. 5 [file 44321_2025_268_MOESM9_ESM.zip › Figure 5/5G/Apoe flox/IL-1+33.tif]

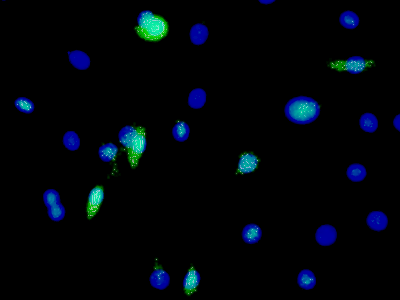

Supplement: Supplementary file 9 — Source data Fig. 5 [file 44321_2025_268_MOESM9_ESM.zip › Figure 5/5G/Apoe flox/IL-1.tif]

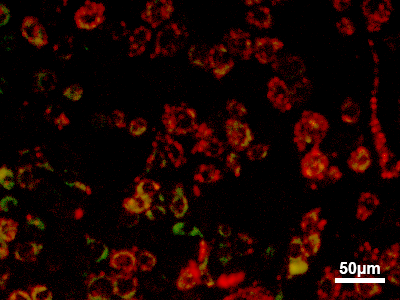

Supplement: Supplementary file 9 — Source data Fig. 5 [file 44321_2025_268_MOESM9_ESM.zip › Figure 5/5H/Apoe +-/IL-1+33+RGX-104.tif]

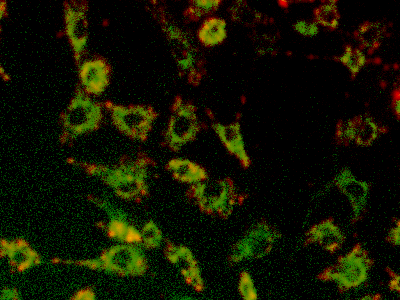

Supplement: Supplementary file 9 — Source data Fig. 5 [file 44321_2025_268_MOESM9_ESM.zip › Figure 5/5H/Apoe +-/IL-1+33.tif]

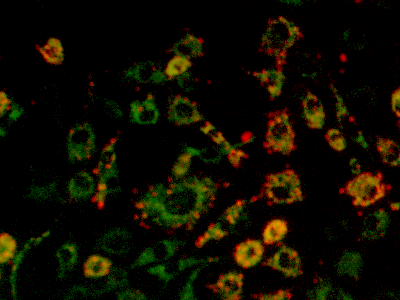

Supplement: Supplementary file 9 — Source data Fig. 5 [file 44321_2025_268_MOESM9_ESM.zip › Figure 5/5H/Apoe +-/IL-1.tif]

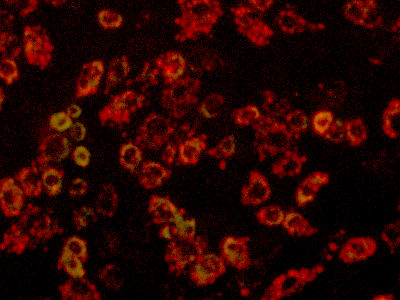

Supplement: Supplementary file 9 — Source data Fig. 5 [file 44321_2025_268_MOESM9_ESM.zip › Figure 5/5H/Apoe flox/IL-1+33+RGX-104.tif]

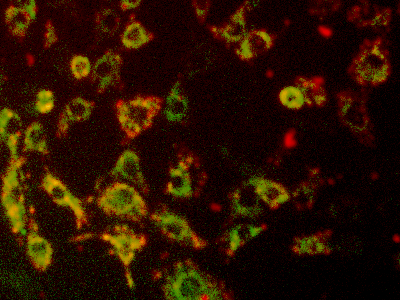

Supplement: Supplementary file 9 — Source data Fig. 5 [file 44321_2025_268_MOESM9_ESM.zip › Figure 5/5H/Apoe flox/IL-1+33.tif]

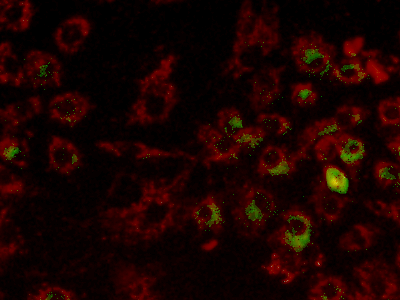

Supplement: Supplementary file 9 — Source data Fig. 5 [file 44321_2025_268_MOESM9_ESM.zip › Figure 5/5H/Apoe flox/IL-1.tif]

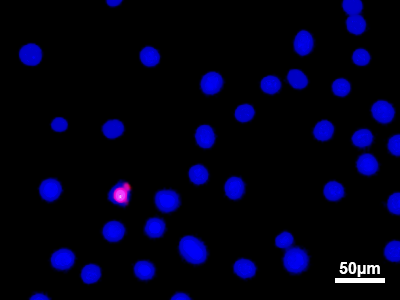

Supplement: Supplementary file 9 — Source data Fig. 5 [file 44321_2025_268_MOESM9_ESM.zip › Figure 5/5I/Apoe +-/IL-1+33+RGX-104.tif]

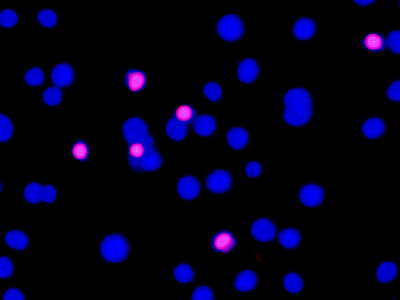

Supplement: Supplementary file 9 — Source data Fig. 5 [file 44321_2025_268_MOESM9_ESM.zip › Figure 5/5I/Apoe +-/IL-1+33.tif]

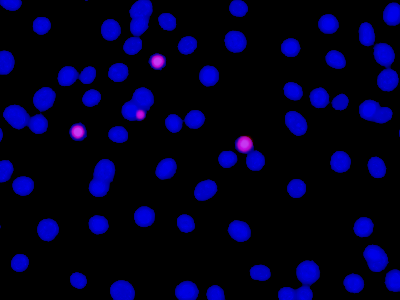

Supplement: Supplementary file 9 — Source data Fig. 5 [file 44321_2025_268_MOESM9_ESM.zip › Figure 5/5I/Apoe +-/IL-1.tif]

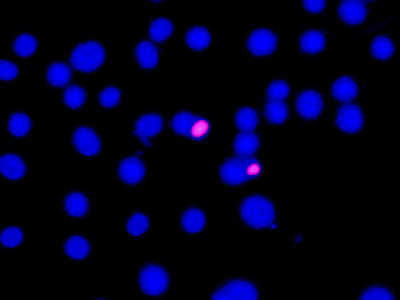

Supplement: Supplementary file 9 — Source data Fig. 5 [file 44321_2025_268_MOESM9_ESM.zip › Figure 5/5I/Apoe flox/IL-1+33+RGX-104.tif]

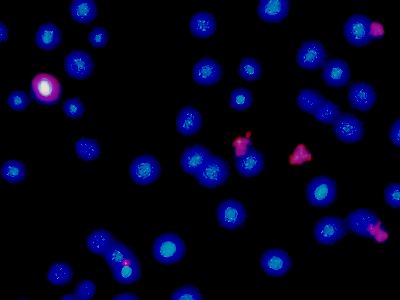

Supplement: Supplementary file 9 — Source data Fig. 5 [file 44321_2025_268_MOESM9_ESM.zip › Figure 5/5I/Apoe flox/IL-1+33.tif]

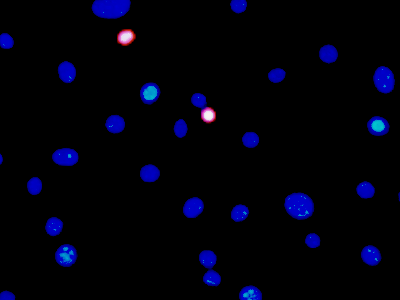

Supplement: Supplementary file 9 — Source data Fig. 5 [file 44321_2025_268_MOESM9_ESM.zip › Figure 5/5I/Apoe flox/IL-1.tif]

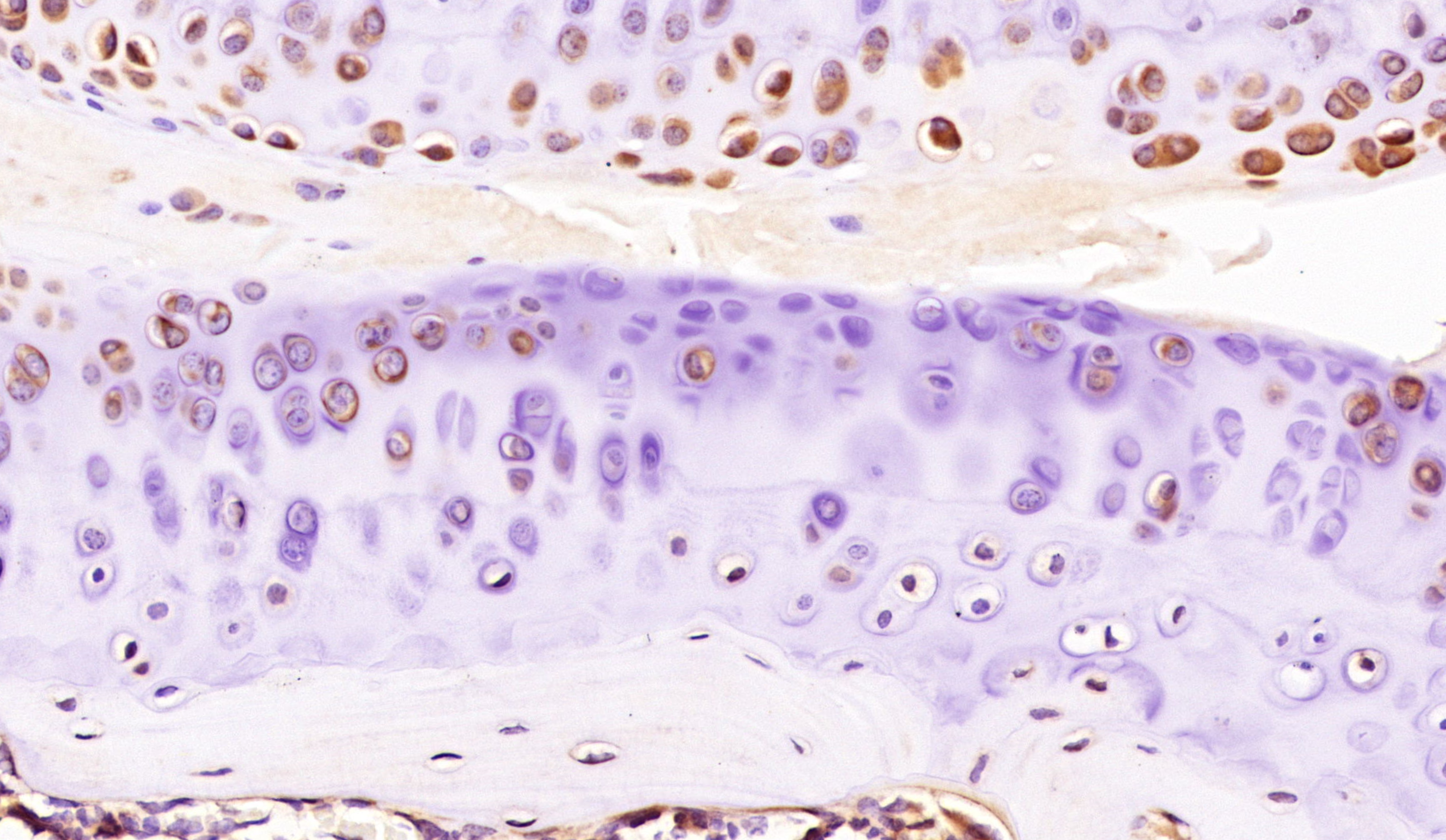

Supplement: Supplementary file 10 — Source data Fig. 6 [file 44321_2025_268_MOESM10_ESM.zip › Figure 6/6B/Apoe +-.tif]

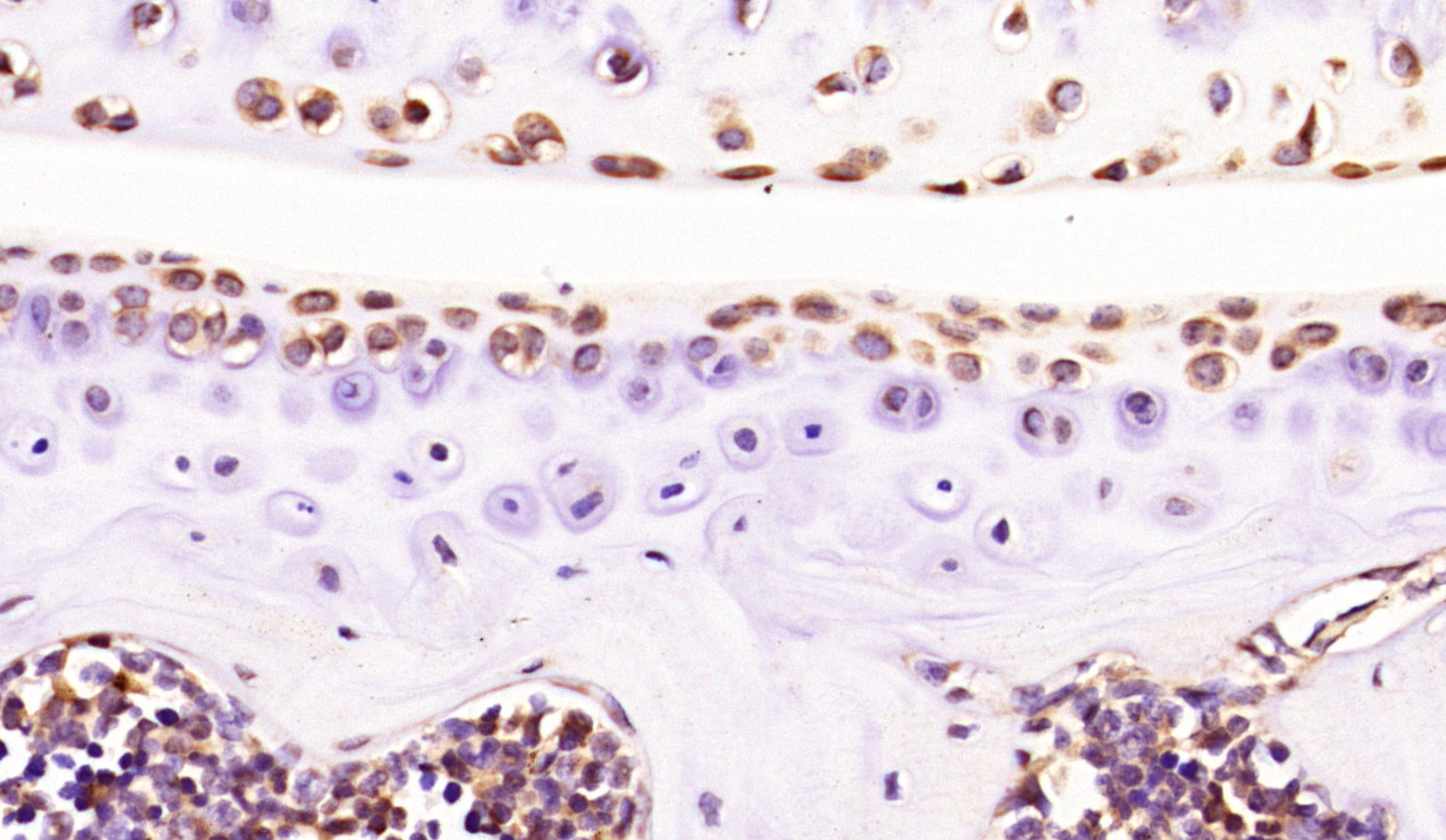

Supplement: Supplementary file 10 — Source data Fig. 6 [file 44321_2025_268_MOESM10_ESM.zip › Figure 6/6B/Apoe flox.tif]

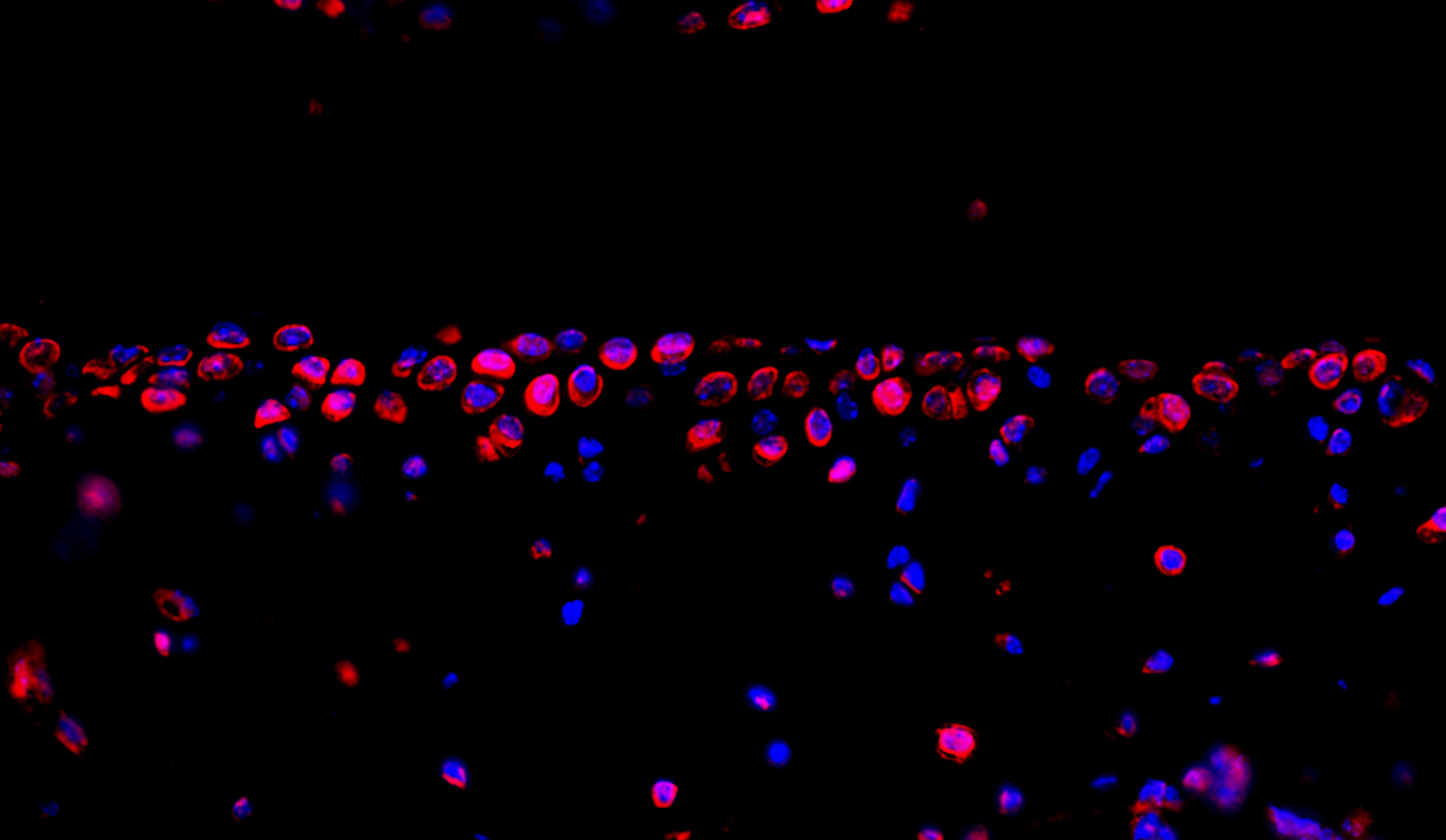

Supplement: Supplementary file 10 — Source data Fig. 6 [file 44321_2025_268_MOESM10_ESM.zip › Figure 6/6C/LTDMM RGX-104 Apoe +-.jpg]

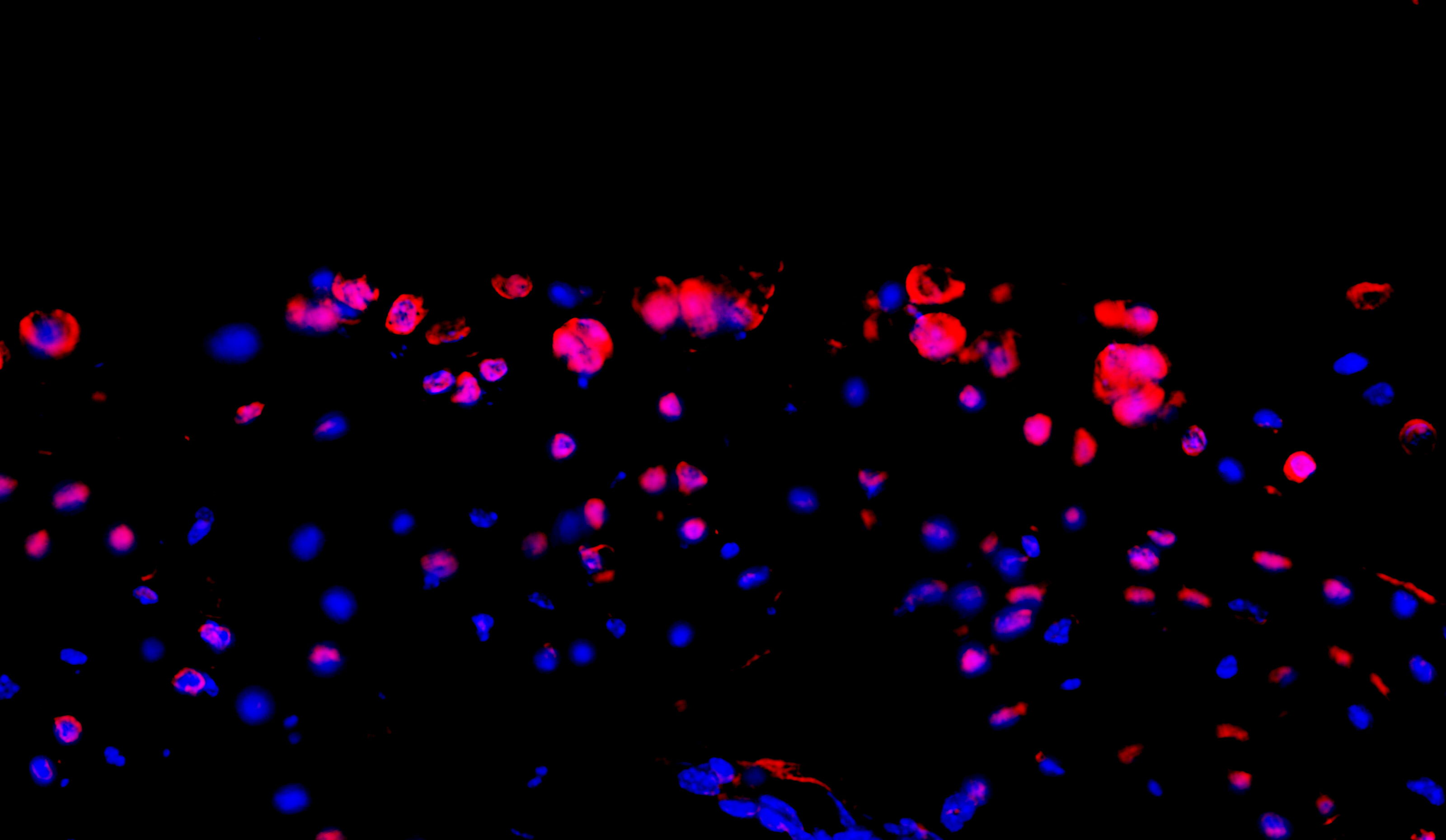

Supplement: Supplementary file 10 — Source data Fig. 6 [file 44321_2025_268_MOESM10_ESM.zip › Figure 6/6C/LTDMM RGX-104 Apoe fl.jpg]

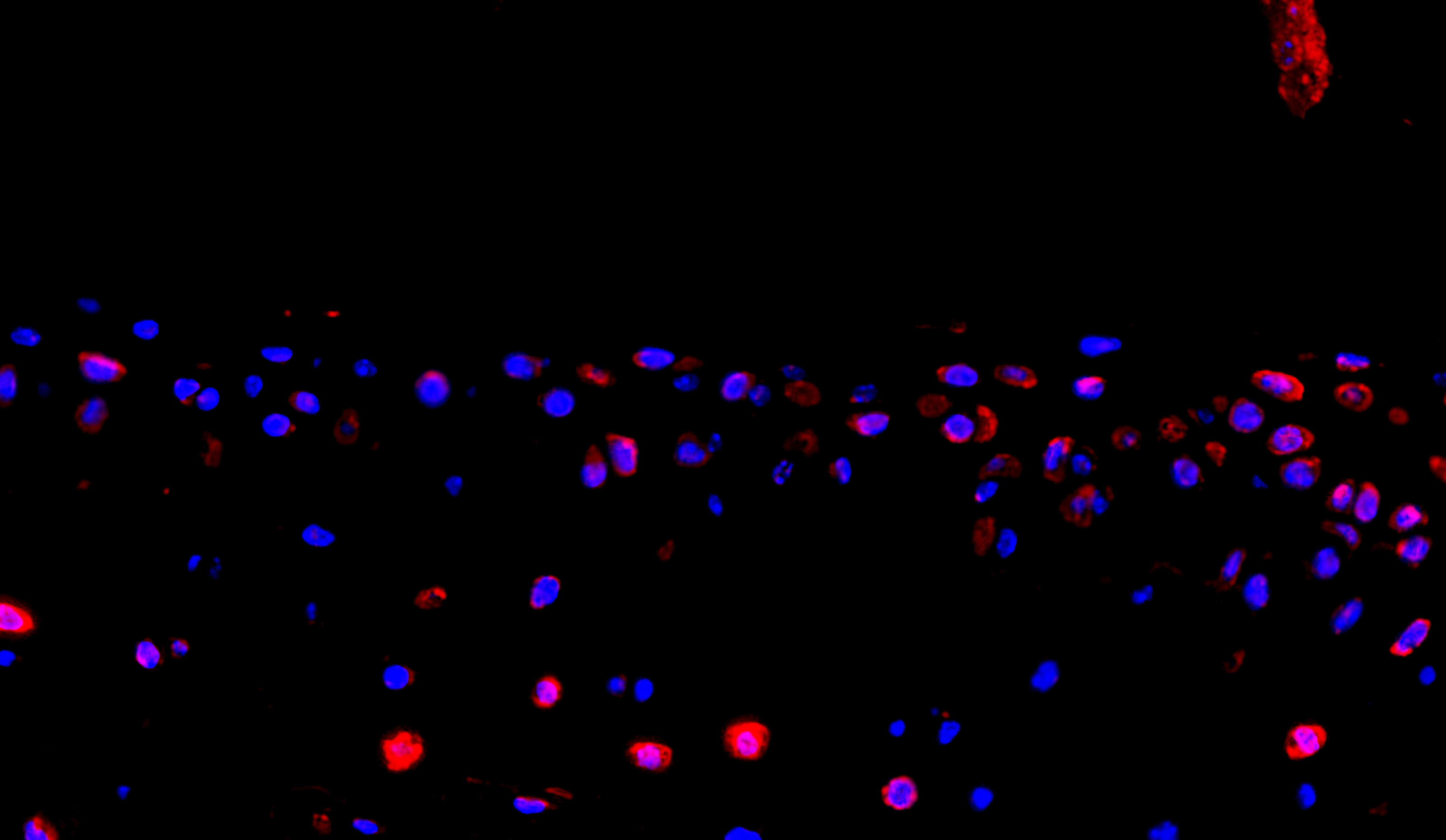

Supplement: Supplementary file 10 — Source data Fig. 6 [file 44321_2025_268_MOESM10_ESM.zip › Figure 6/6C/LTDMM vehicle Apoe +-.jpg]

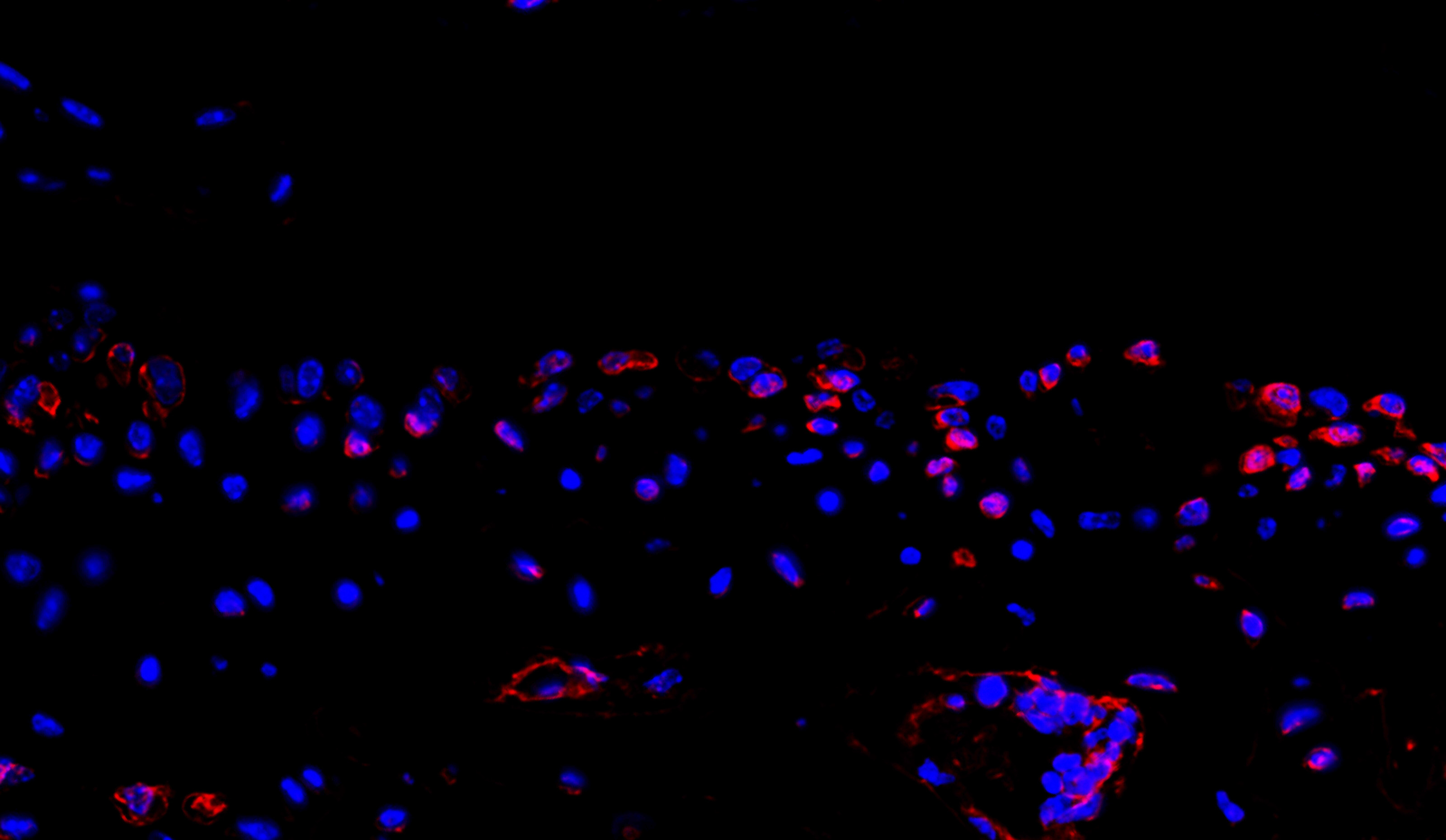

Supplement: Supplementary file 10 — Source data Fig. 6 [file 44321_2025_268_MOESM10_ESM.zip › Figure 6/6C/LTDMM Vehicle Apoe fl.jpg]

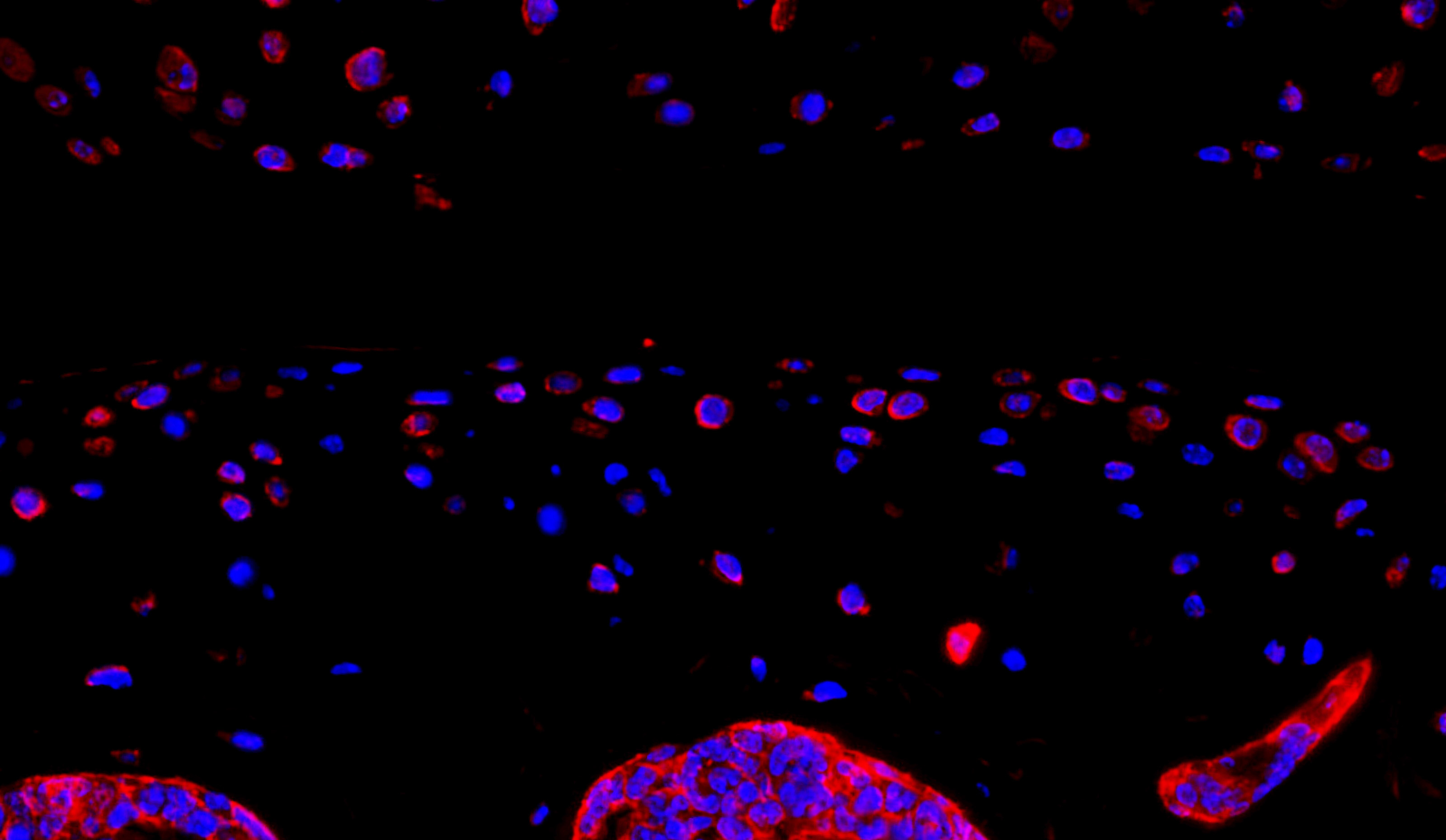

Supplement: Supplementary file 10 — Source data Fig. 6 [file 44321_2025_268_MOESM10_ESM.zip › Figure 6/6C/RTDMM Apoe +-.jpg]

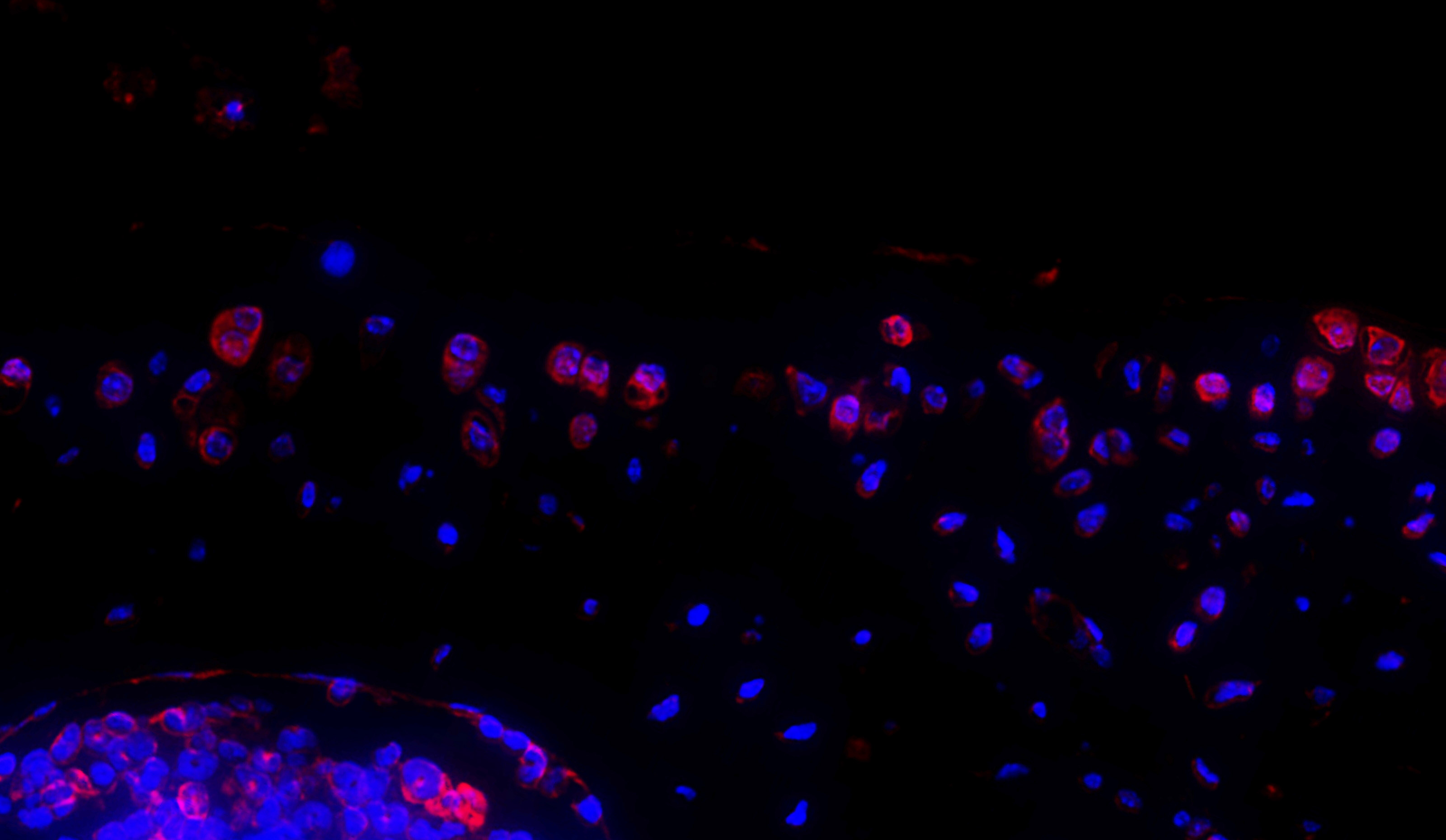

Supplement: Supplementary file 10 — Source data Fig. 6 [file 44321_2025_268_MOESM10_ESM.zip › Figure 6/6C/RTDMM Apoe fl.jpg]

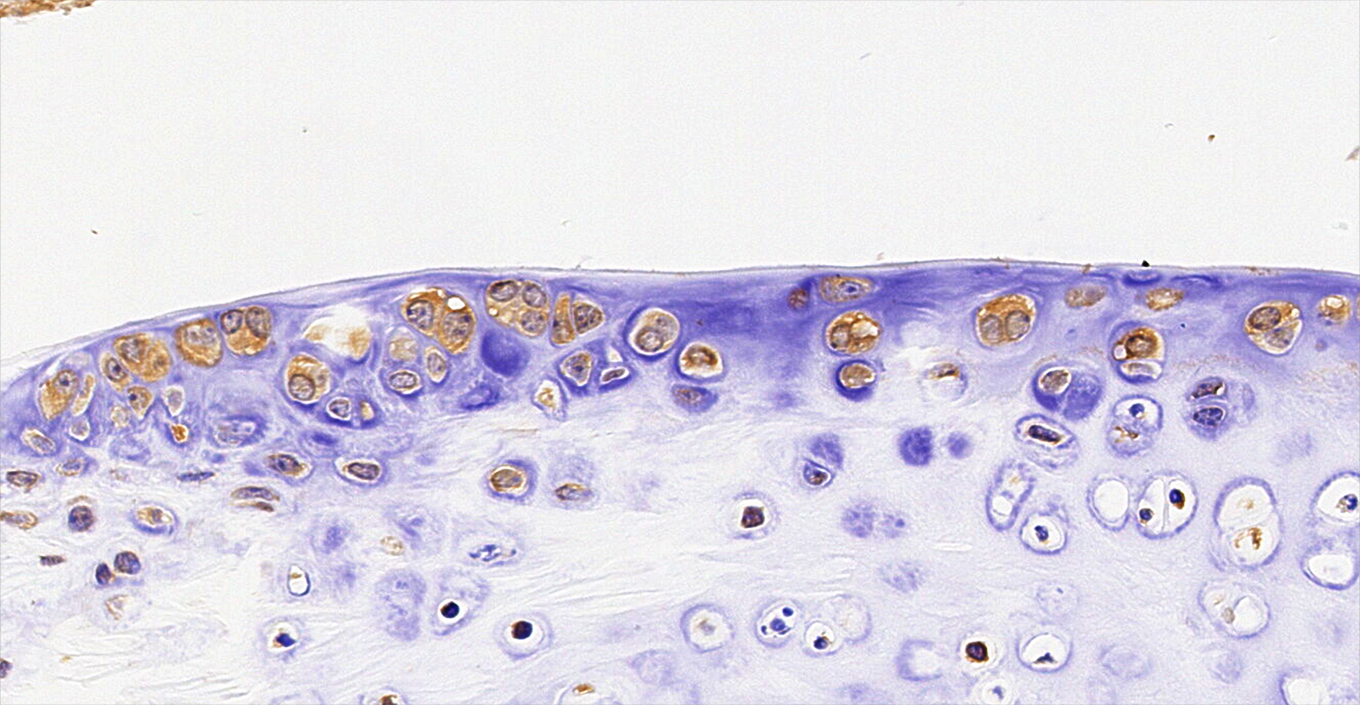

Supplement: Supplementary file 10 — Source data Fig. 6 [file 44321_2025_268_MOESM10_ESM.zip › Figure 6/6D/LTDMM RGX-104 Apoe +-.tiff]

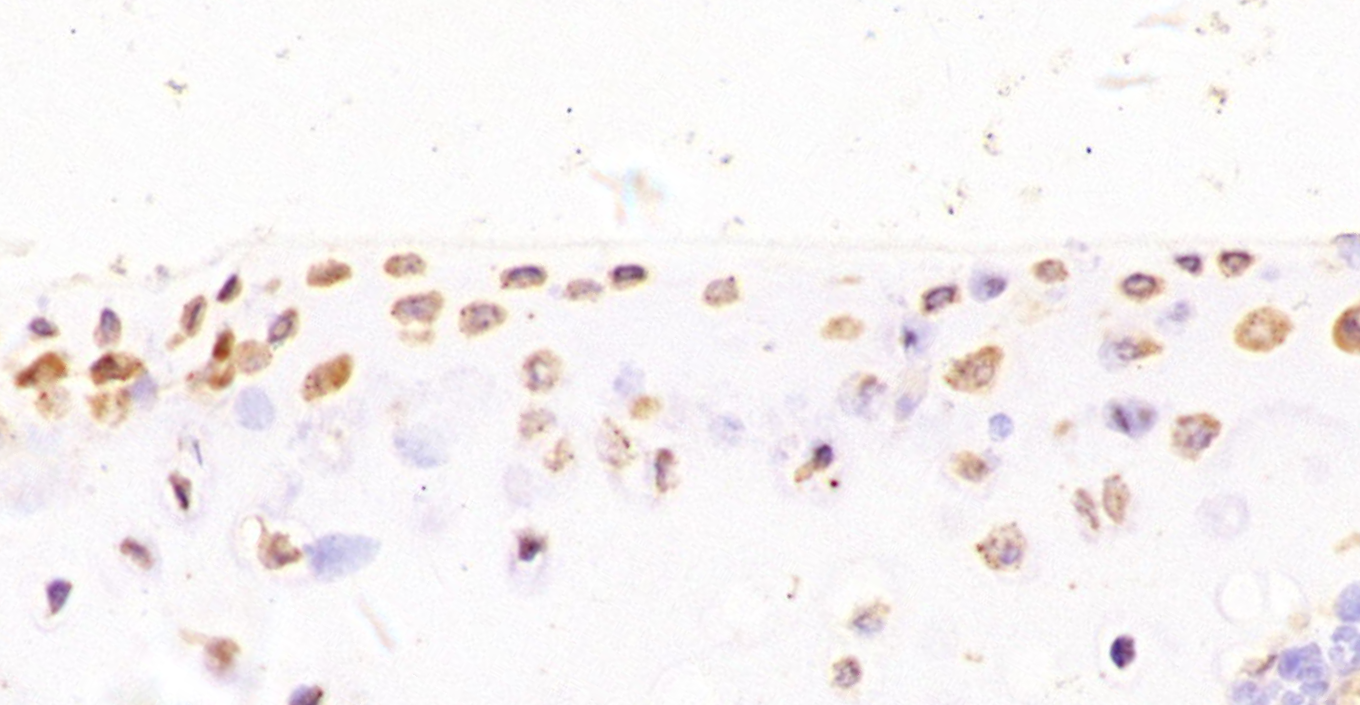

Supplement: Supplementary file 10 — Source data Fig. 6 [file 44321_2025_268_MOESM10_ESM.zip › Figure 6/6D/LTDMM RGX-104 Apoe flox.tif]

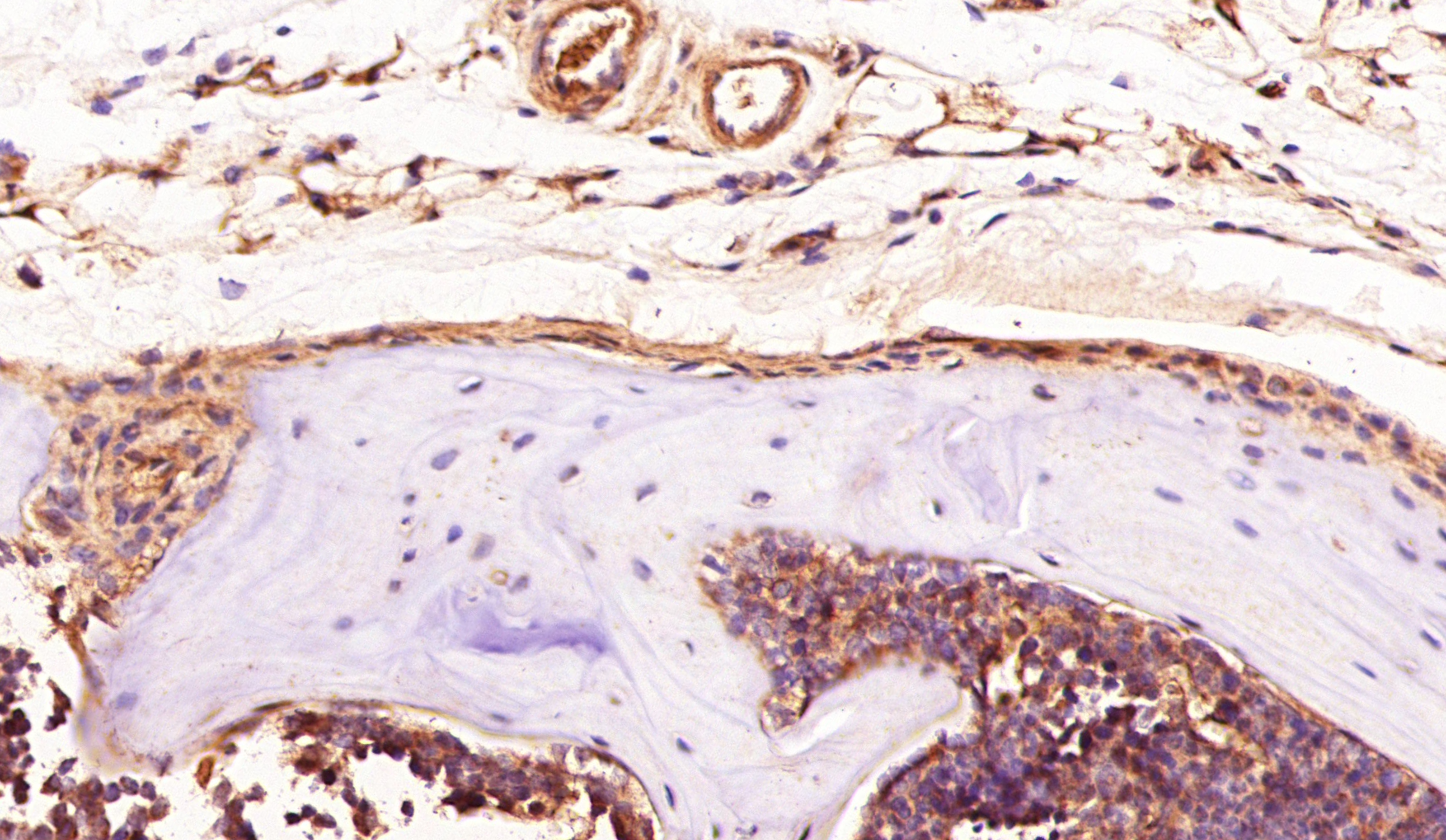

Supplement: Supplementary file 10 — Source data Fig. 6 [file 44321_2025_268_MOESM10_ESM.zip › Figure 6/6D/LTDMM Vehicle Apoe +-.tif]

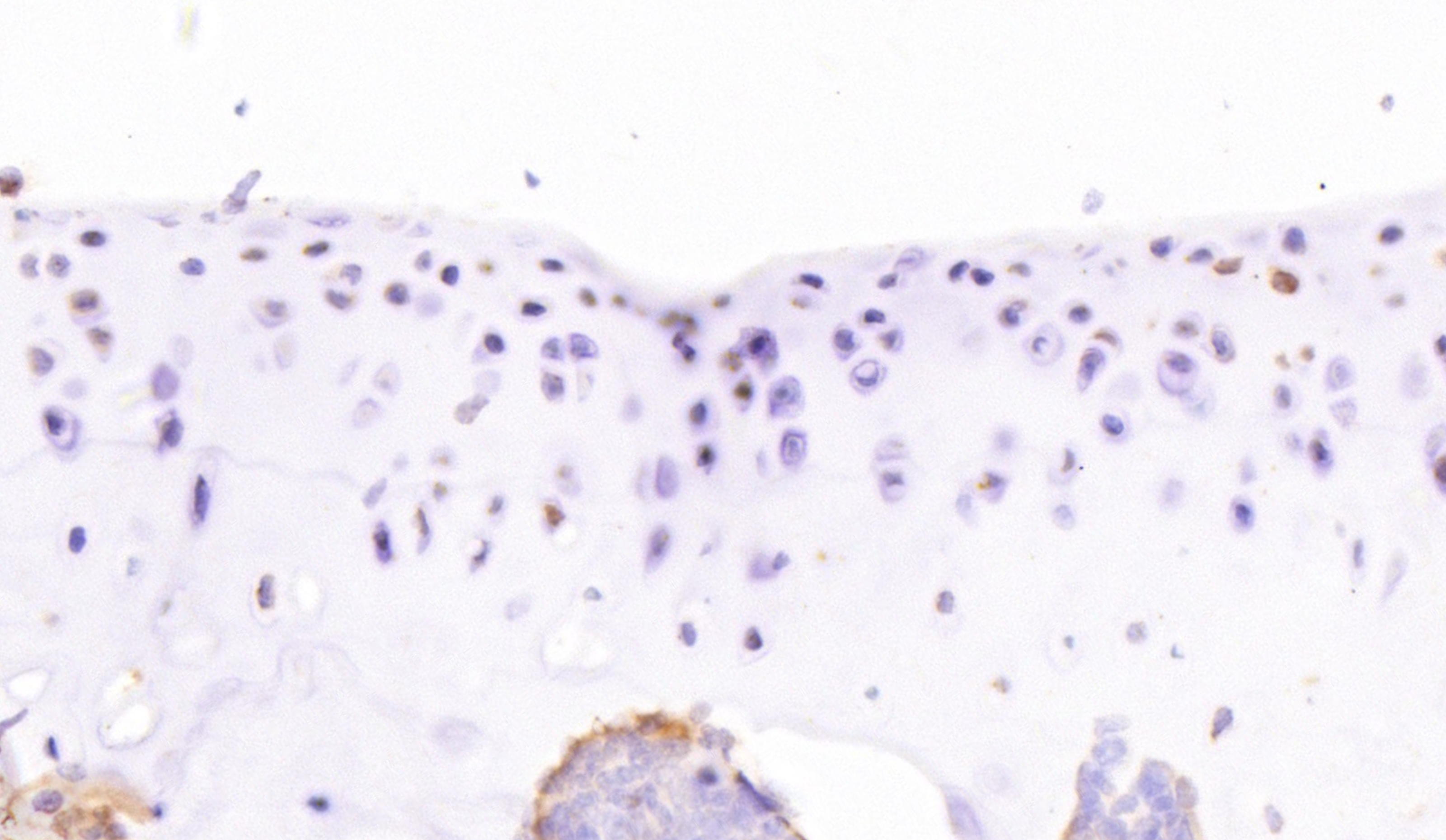

Supplement: Supplementary file 10 — Source data Fig. 6 [file 44321_2025_268_MOESM10_ESM.zip › Figure 6/6D/LTDMM Vehicle Apoe flox.tif]

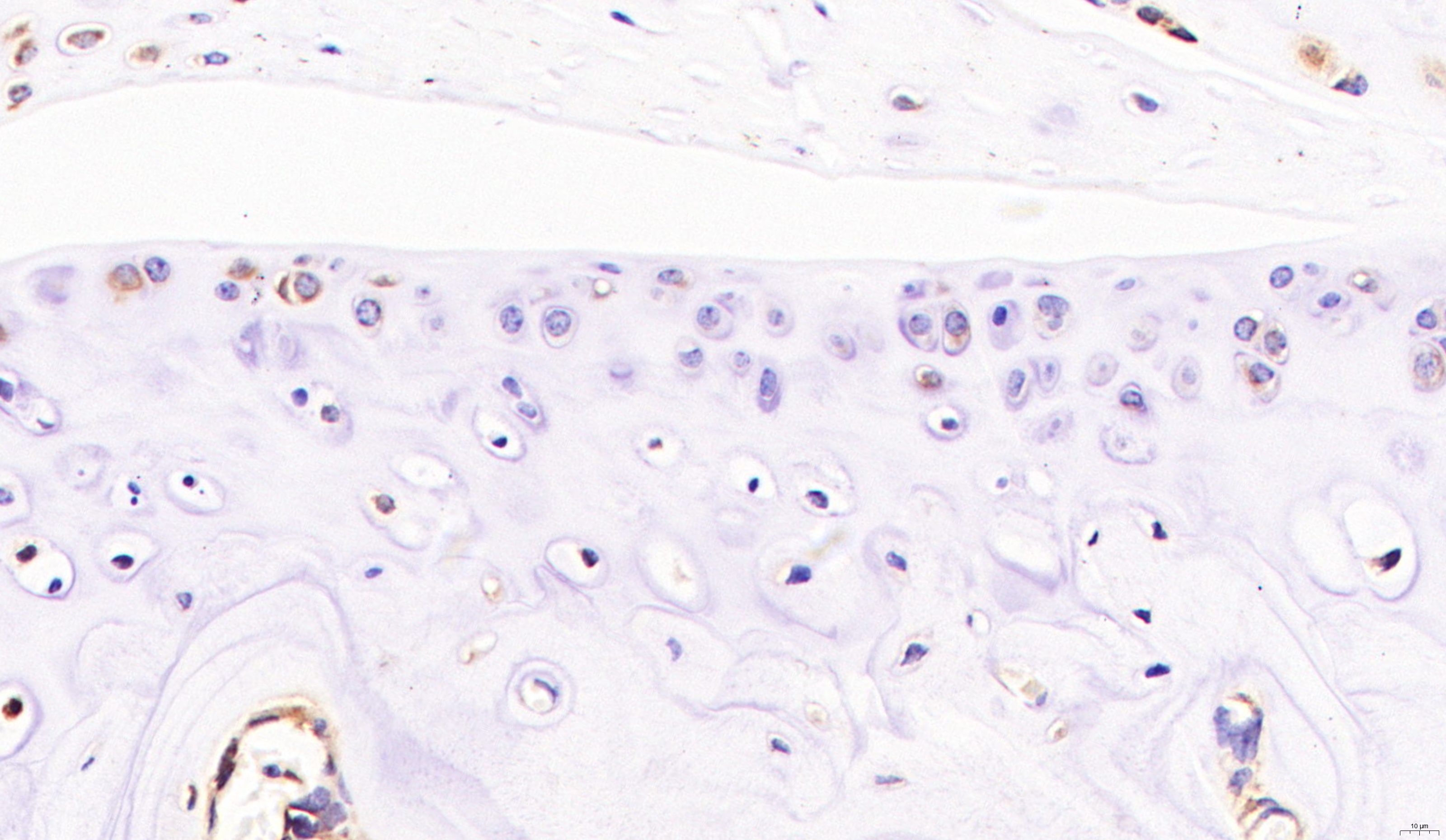

Supplement: Supplementary file 10 — Source data Fig. 6 [file 44321_2025_268_MOESM10_ESM.zip › Figure 6/6D/RTDMM Apoe +-.tif]

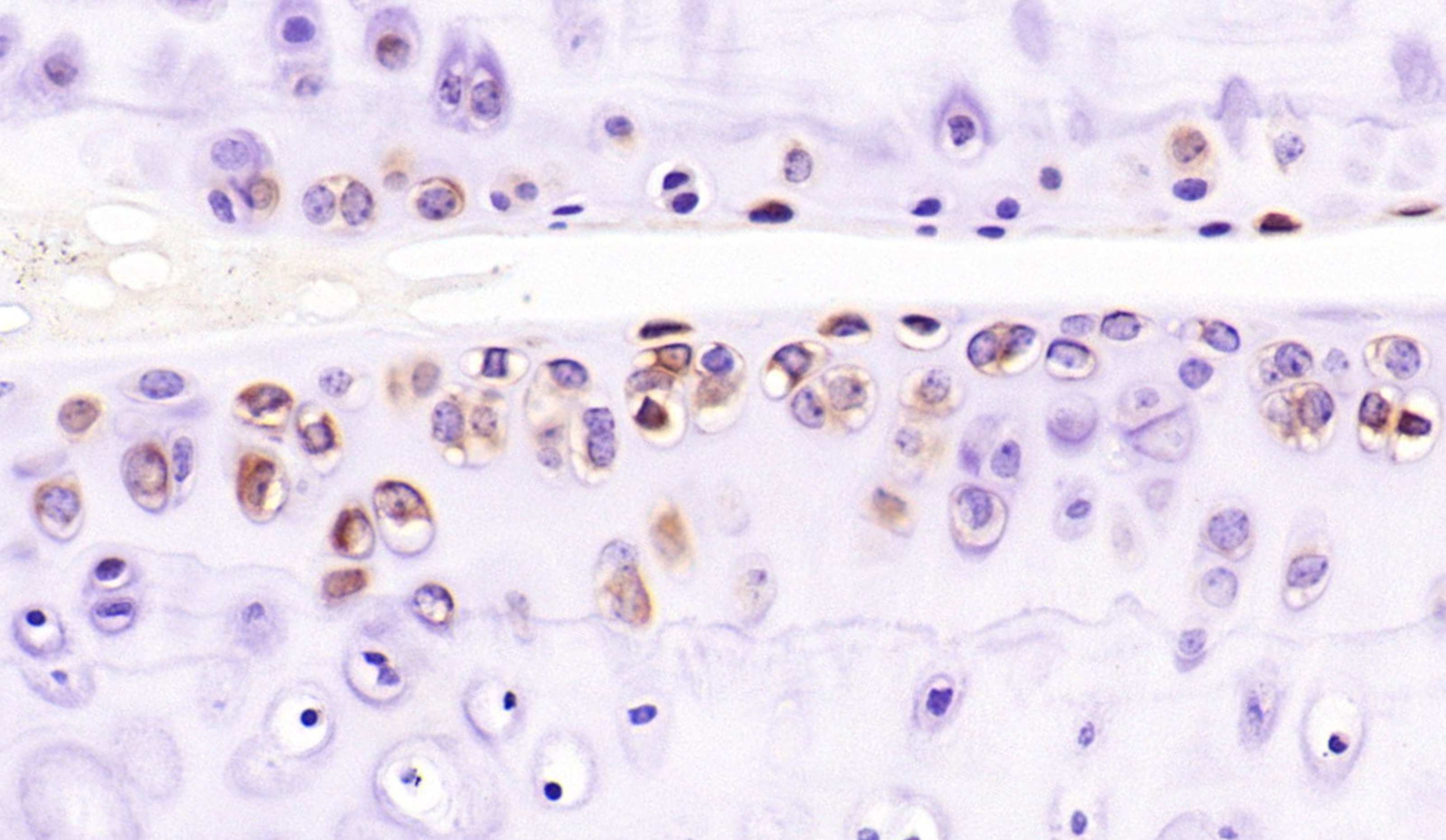

Supplement: Supplementary file 10 — Source data Fig. 6 [file 44321_2025_268_MOESM10_ESM.zip › Figure 6/6D/RTDMM Apoe flox.tif]

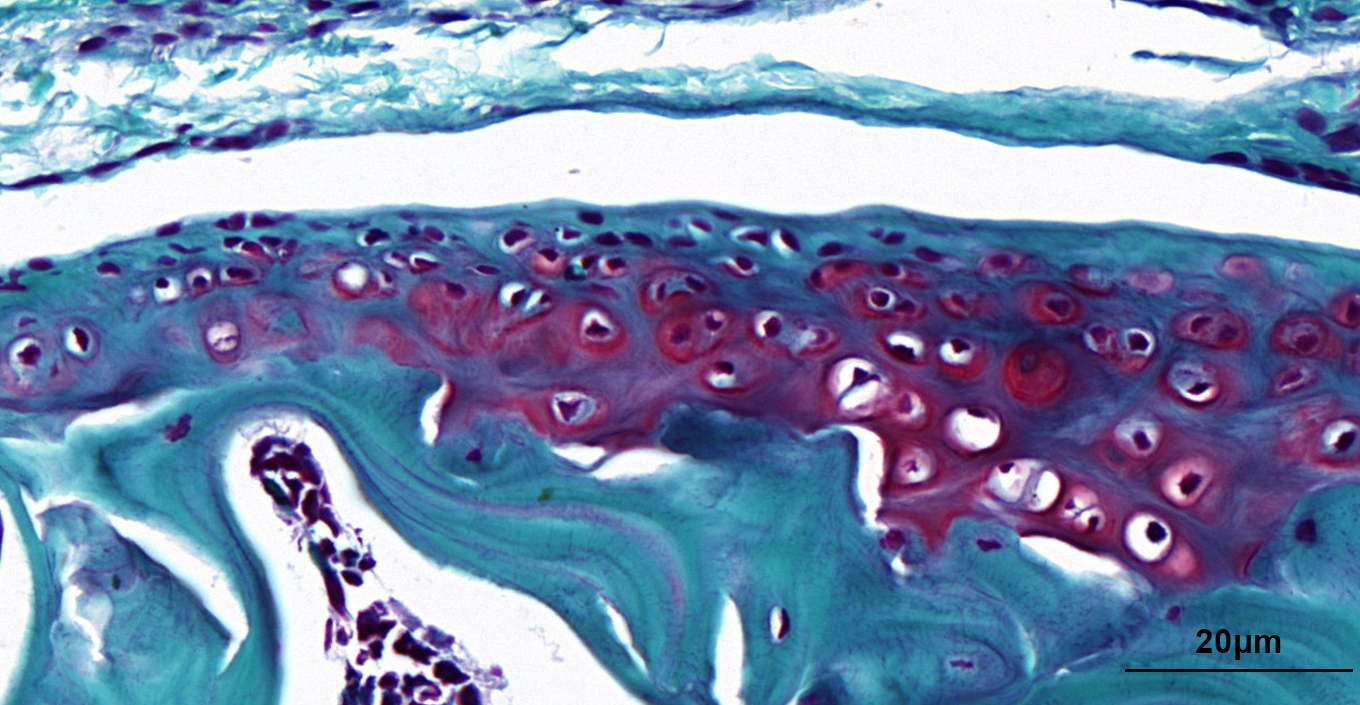

Supplement: Supplementary file 10 — Source data Fig. 6 [file 44321_2025_268_MOESM10_ESM.zip › Figure 6/6E/LTDMM RGX-104 Apoe +-.tif]

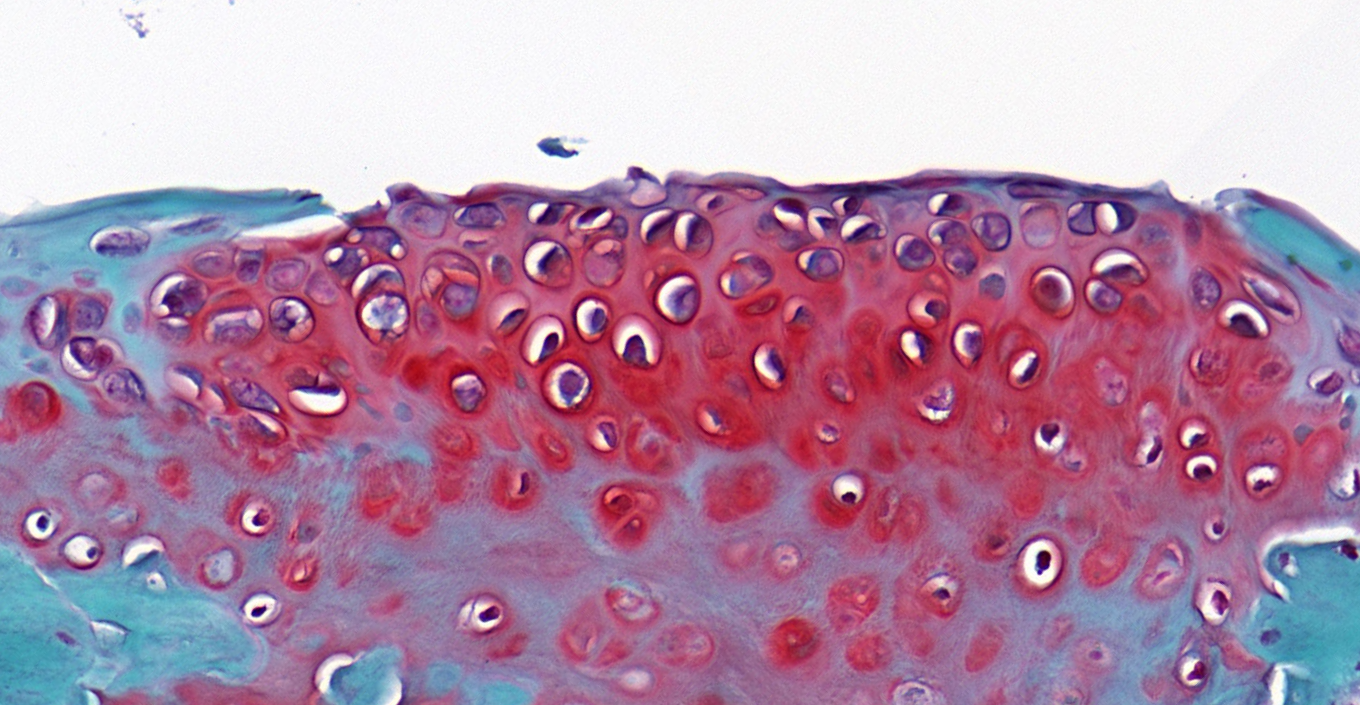

Supplement: Supplementary file 10 — Source data Fig. 6 [file 44321_2025_268_MOESM10_ESM.zip › Figure 6/6E/LTDMM RGX-104 Apoe flox.tif]

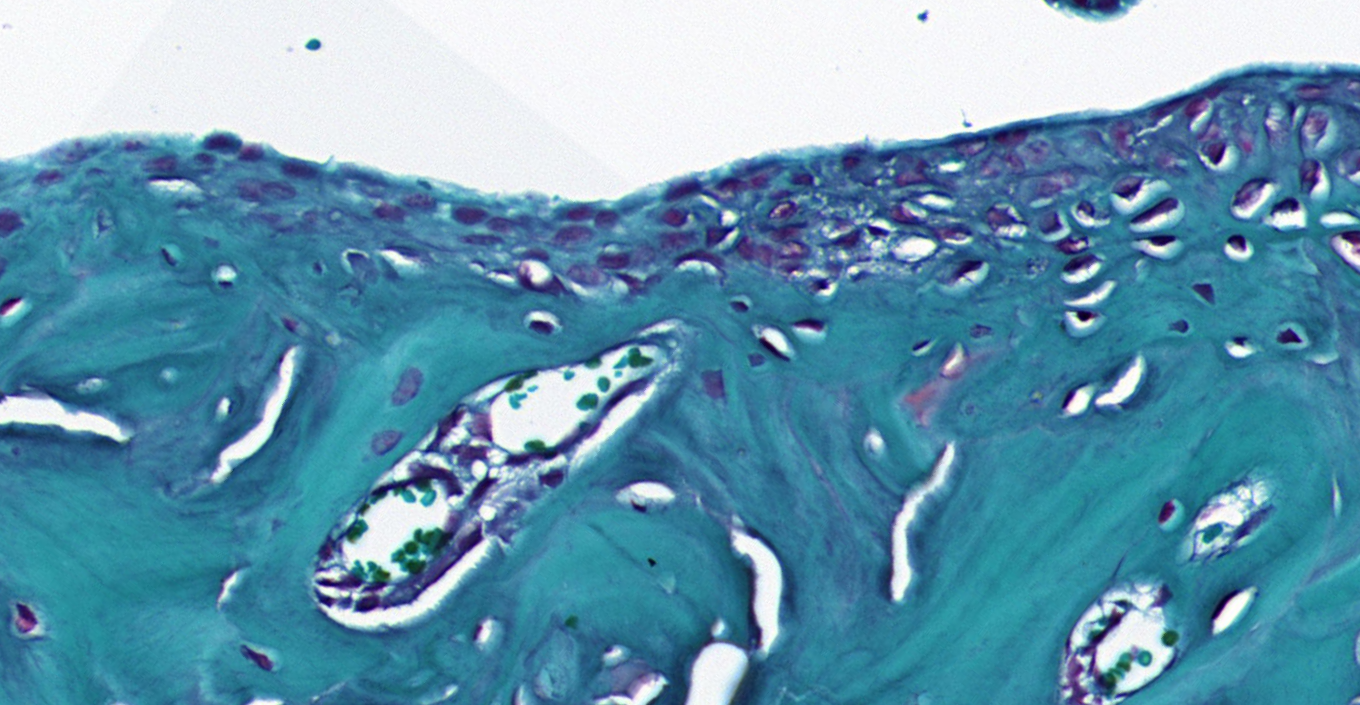

Supplement: Supplementary file 10 — Source data Fig. 6 [file 44321_2025_268_MOESM10_ESM.zip › Figure 6/6E/LTDMM Vehicle Apoe +-.tif]

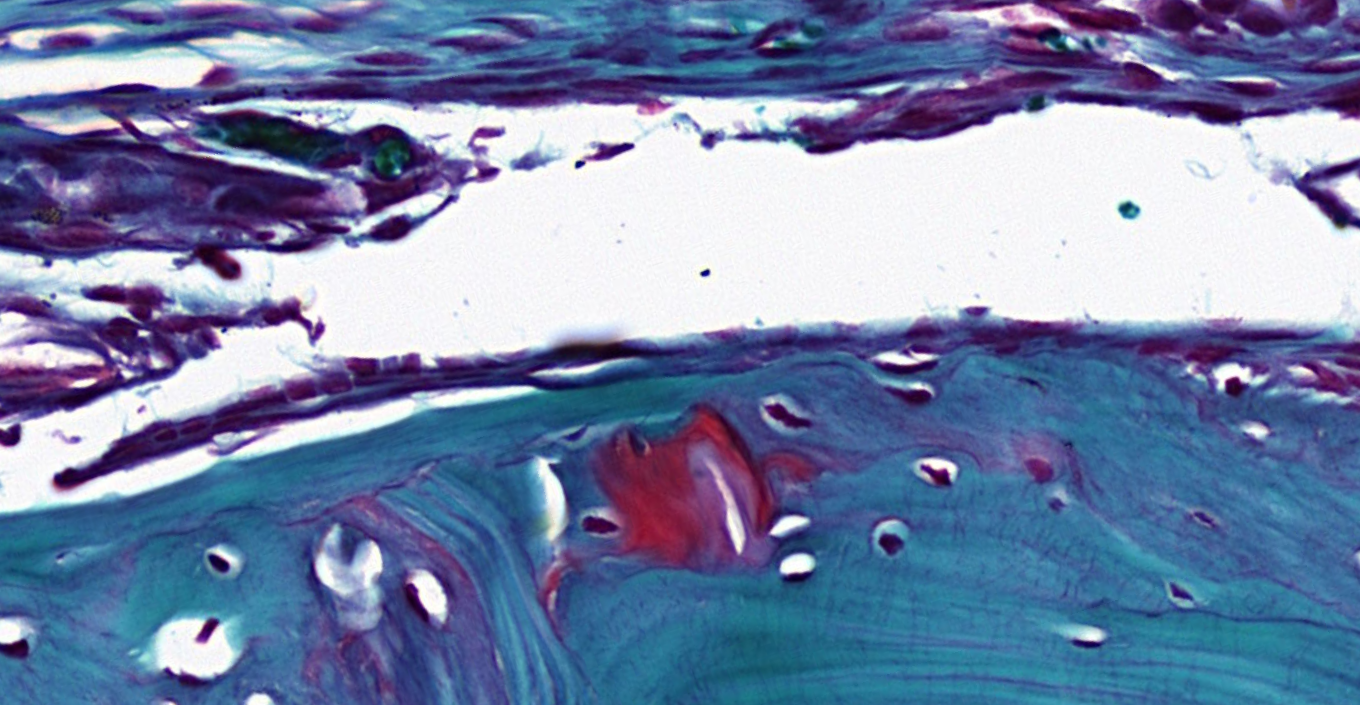

Supplement: Supplementary file 10 — Source data Fig. 6 [file 44321_2025_268_MOESM10_ESM.zip › Figure 6/6E/LTDMM vehicle Apoeflox.tif]

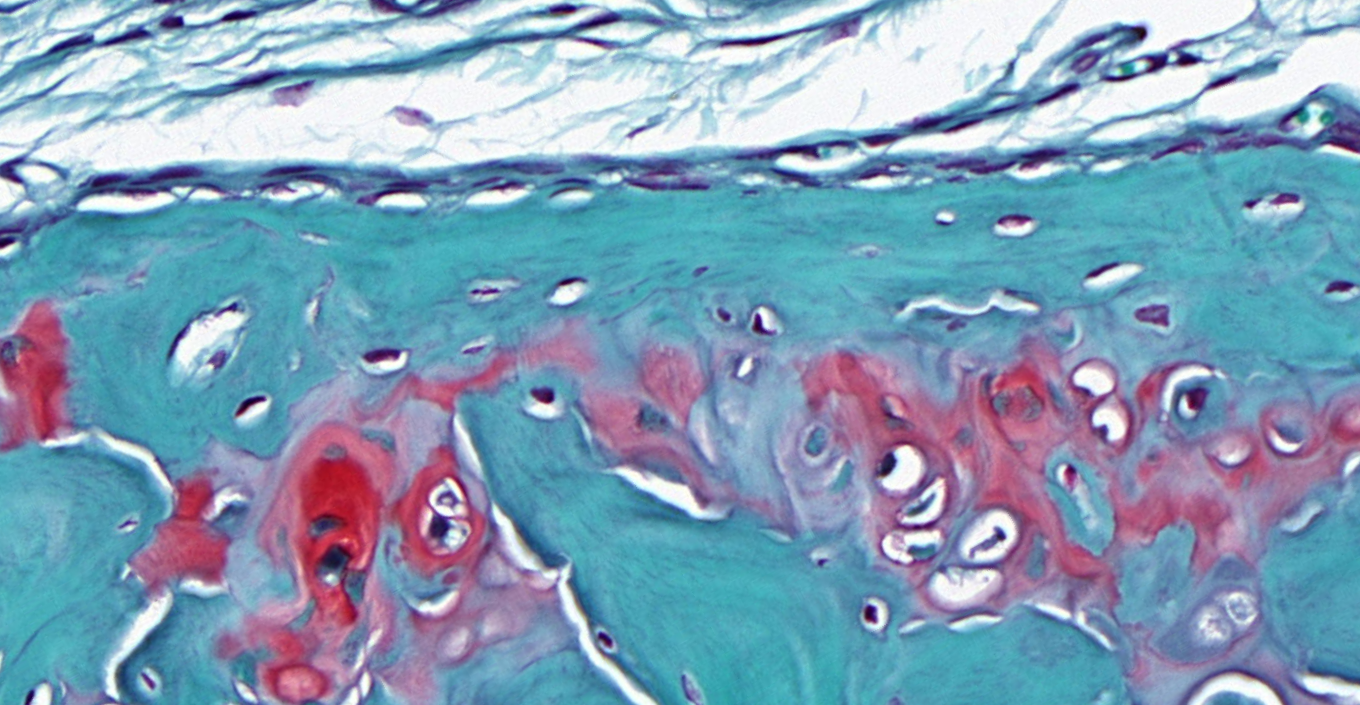

Supplement: Supplementary file 10 — Source data Fig. 6 [file 44321_2025_268_MOESM10_ESM.zip › Figure 6/6E/RT DMM Apoe +-.tif]

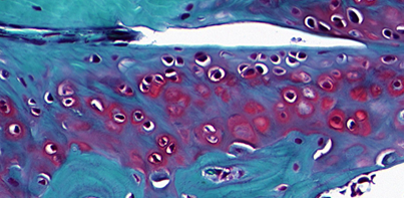

Supplement: Supplementary file 10 — Source data Fig. 6 [file 44321_2025_268_MOESM10_ESM.zip › Figure 6/6E/RT DMM Apoeflox.tif]

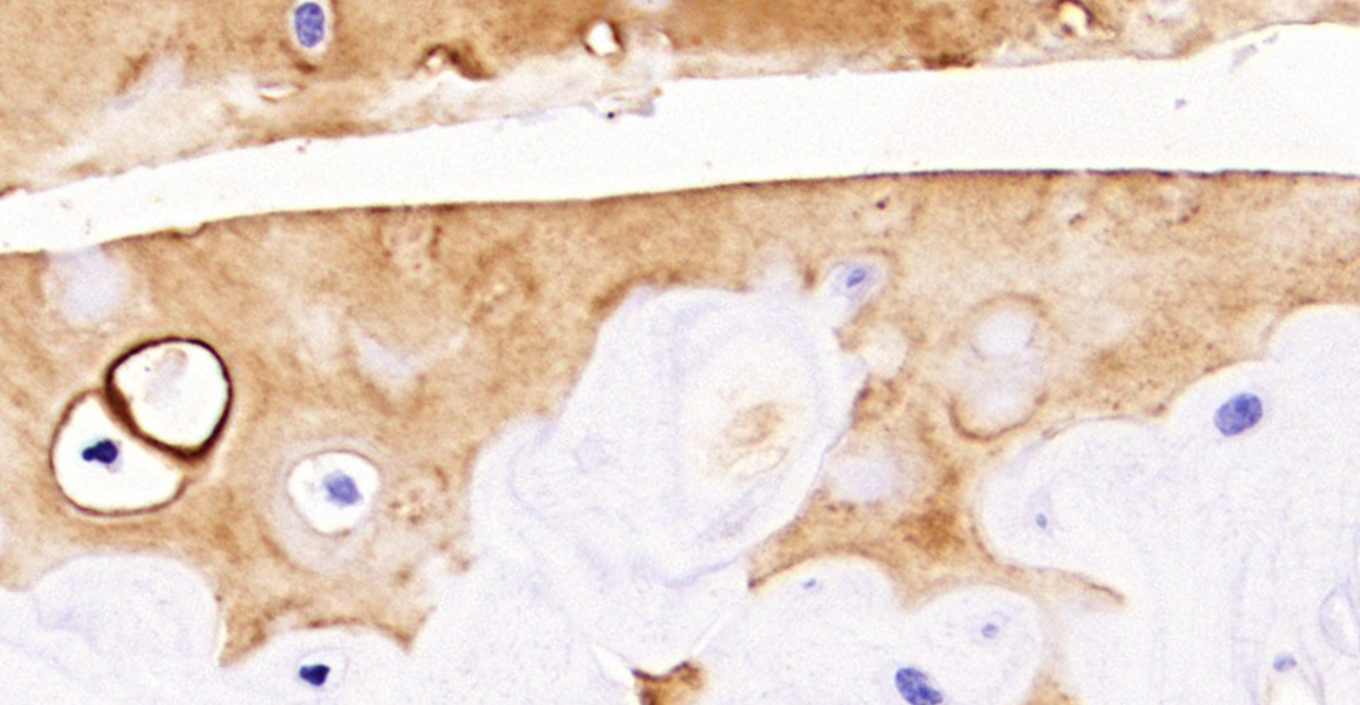

Supplement: Supplementary file 10 — Source data Fig. 6 [file 44321_2025_268_MOESM10_ESM.zip › Figure 6/6F/LTDMM RGX-104 Apoe +-.tif]

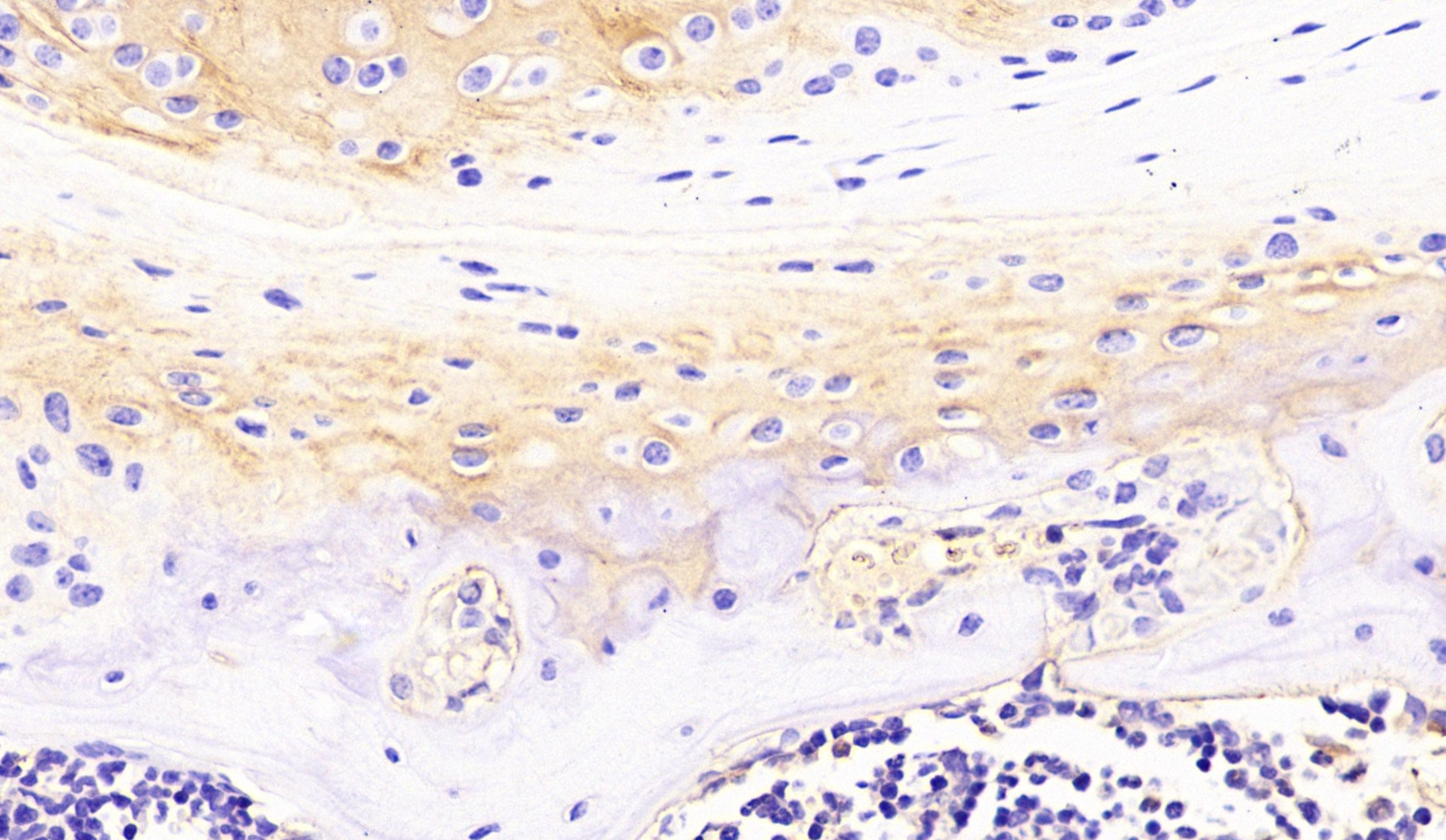

Supplement: Supplementary file 10 — Source data Fig. 6 [file 44321_2025_268_MOESM10_ESM.zip › Figure 6/6F/LTDMM RGX-104 Apoe flox.tif]

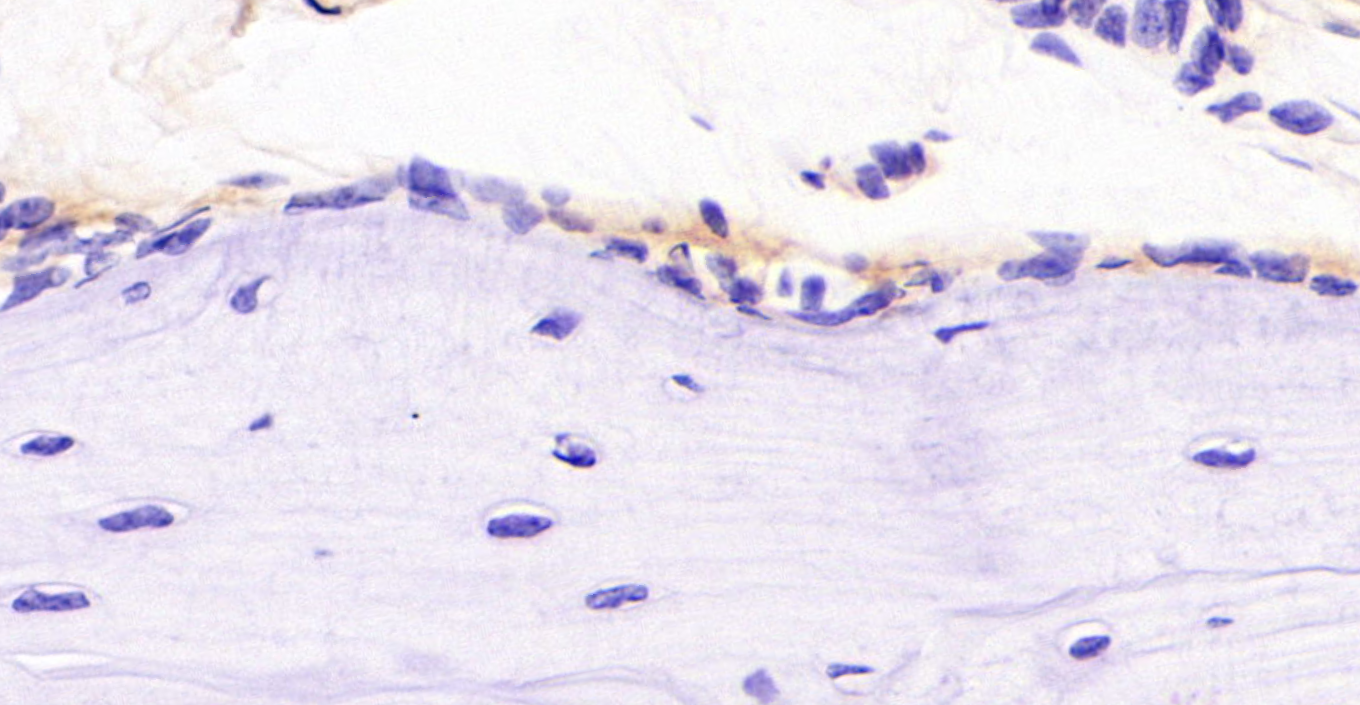

Supplement: Supplementary file 10 — Source data Fig. 6 [file 44321_2025_268_MOESM10_ESM.zip › Figure 6/6F/LTDMM Vehicle Apoe +-.tif]

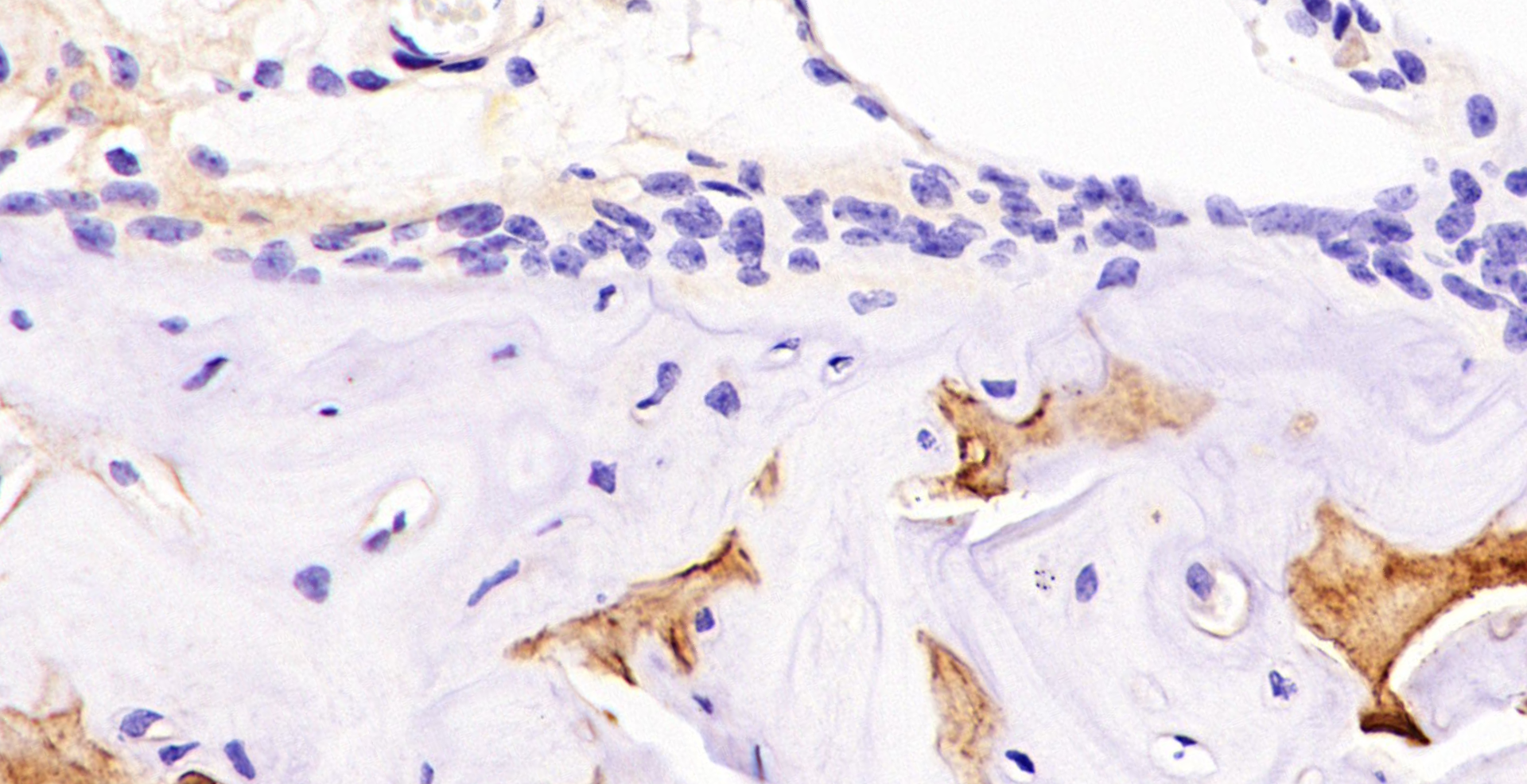

Supplement: Supplementary file 10 — Source data Fig. 6 [file 44321_2025_268_MOESM10_ESM.zip › Figure 6/6F/LTDMM Vehicle Apoe flox.tif]

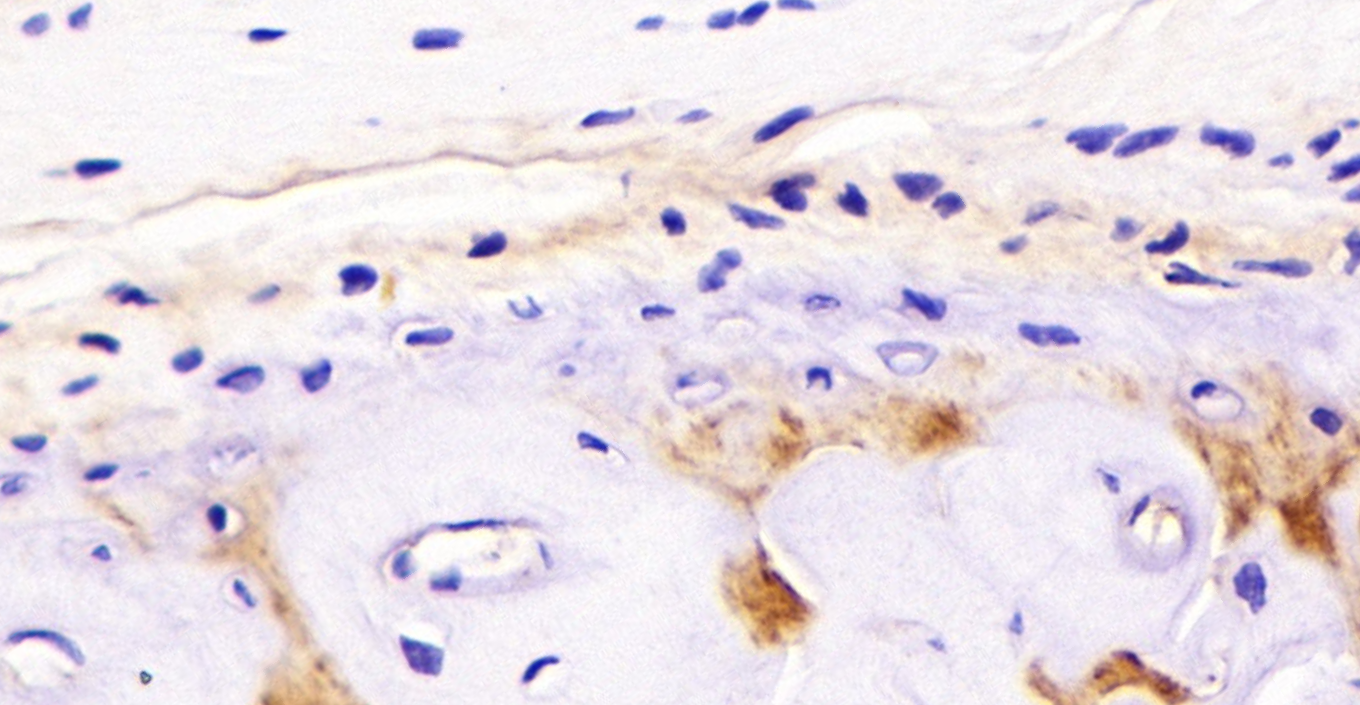

Supplement: Supplementary file 10 — Source data Fig. 6 [file 44321_2025_268_MOESM10_ESM.zip › Figure 6/6F/RTDMM Apoe +-.tif]

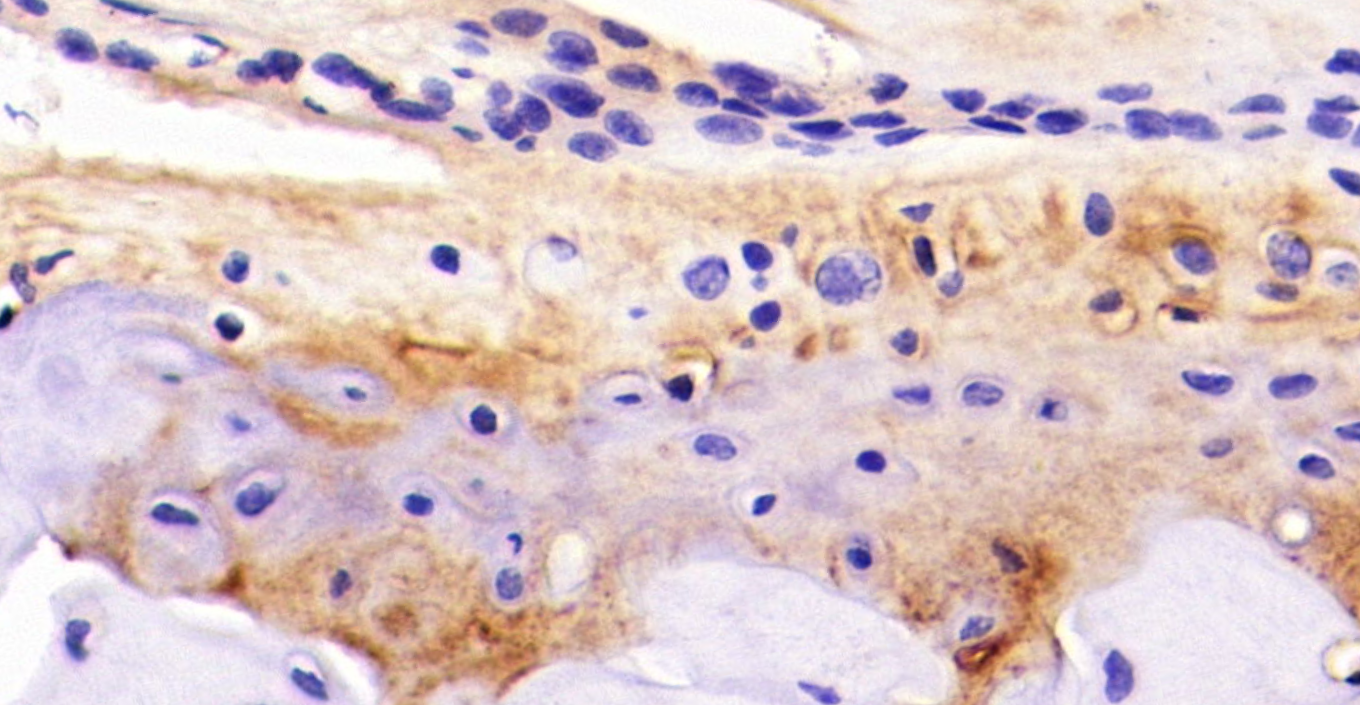

Supplement: Supplementary file 10 — Source data Fig. 6 [file 44321_2025_268_MOESM10_ESM.zip › Figure 6/6F/RTDMM Apoe flox.tif]

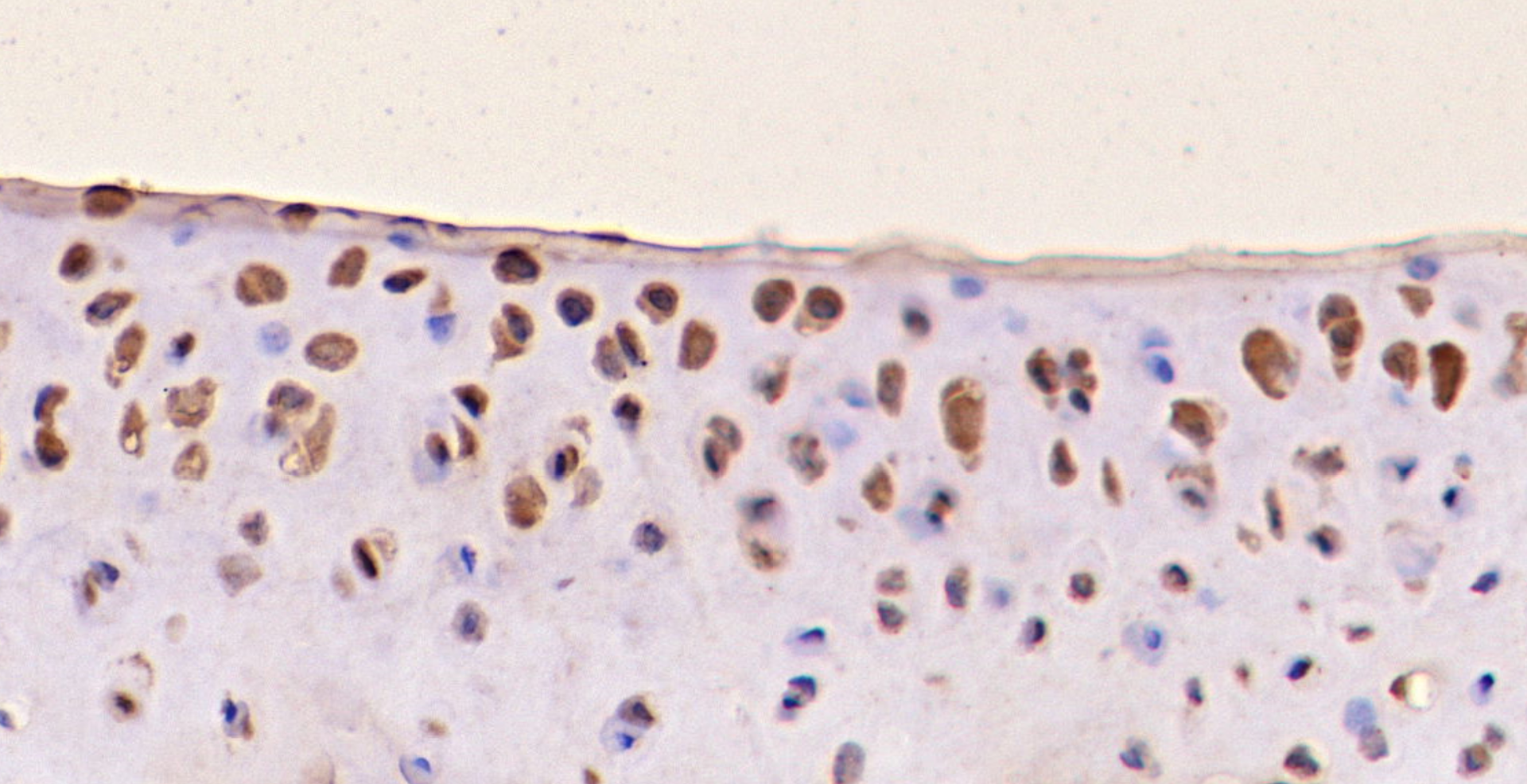

Supplement: Supplementary file 10 — Source data Fig. 6 [file 44321_2025_268_MOESM10_ESM.zip › Figure 6/6G/LTDMM Vehicle Apoe +-.tif]

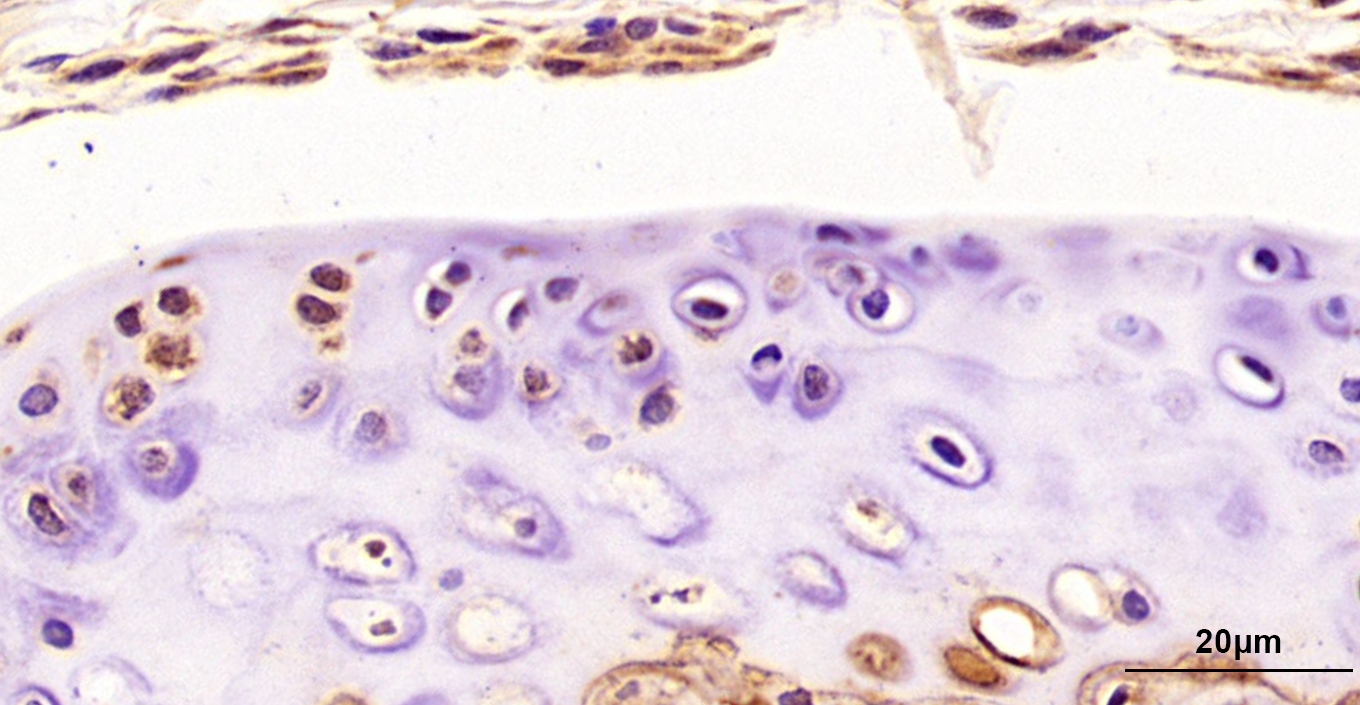

Supplement: Supplementary file 10 — Source data Fig. 6 [file 44321_2025_268_MOESM10_ESM.zip › Figure 6/6G/LTDMM RGX-104 Apoe +-.tif]

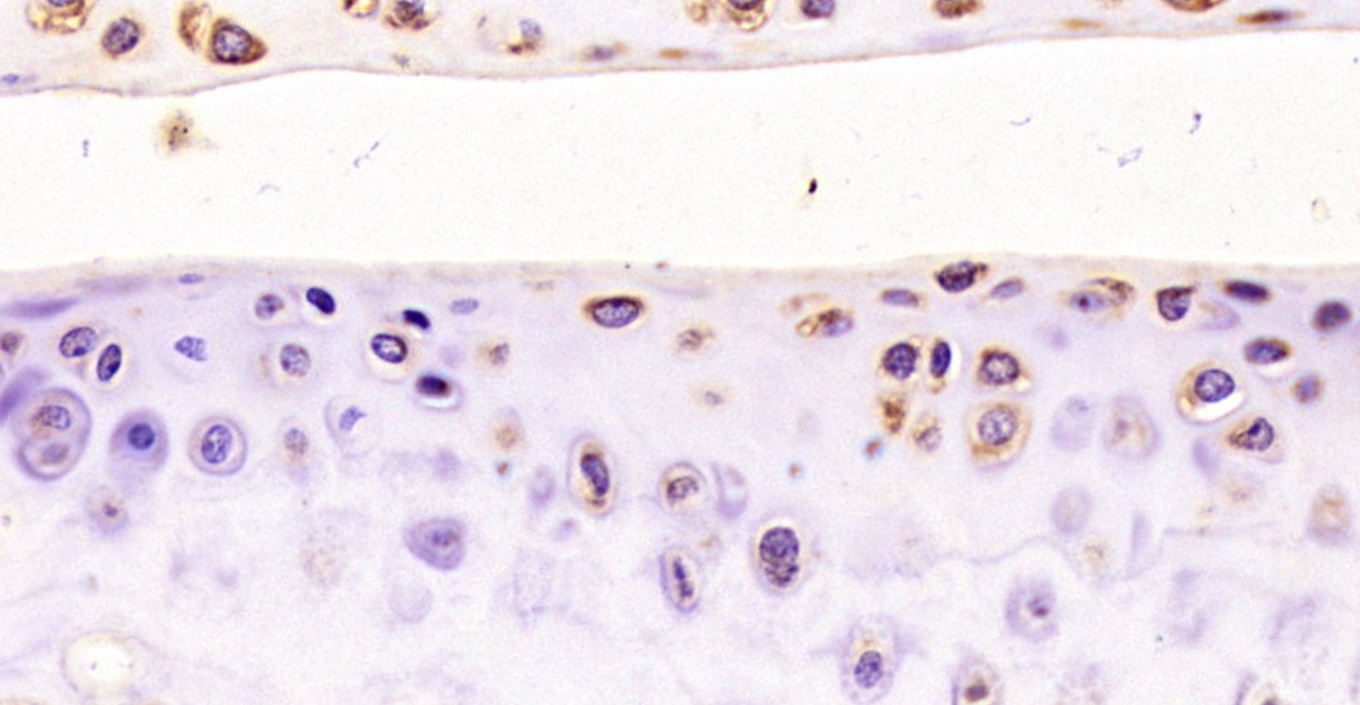

Supplement: Supplementary file 10 — Source data Fig. 6 [file 44321_2025_268_MOESM10_ESM.zip › Figure 6/6G/LTDMM RGX-104 Apoe flox.tif]

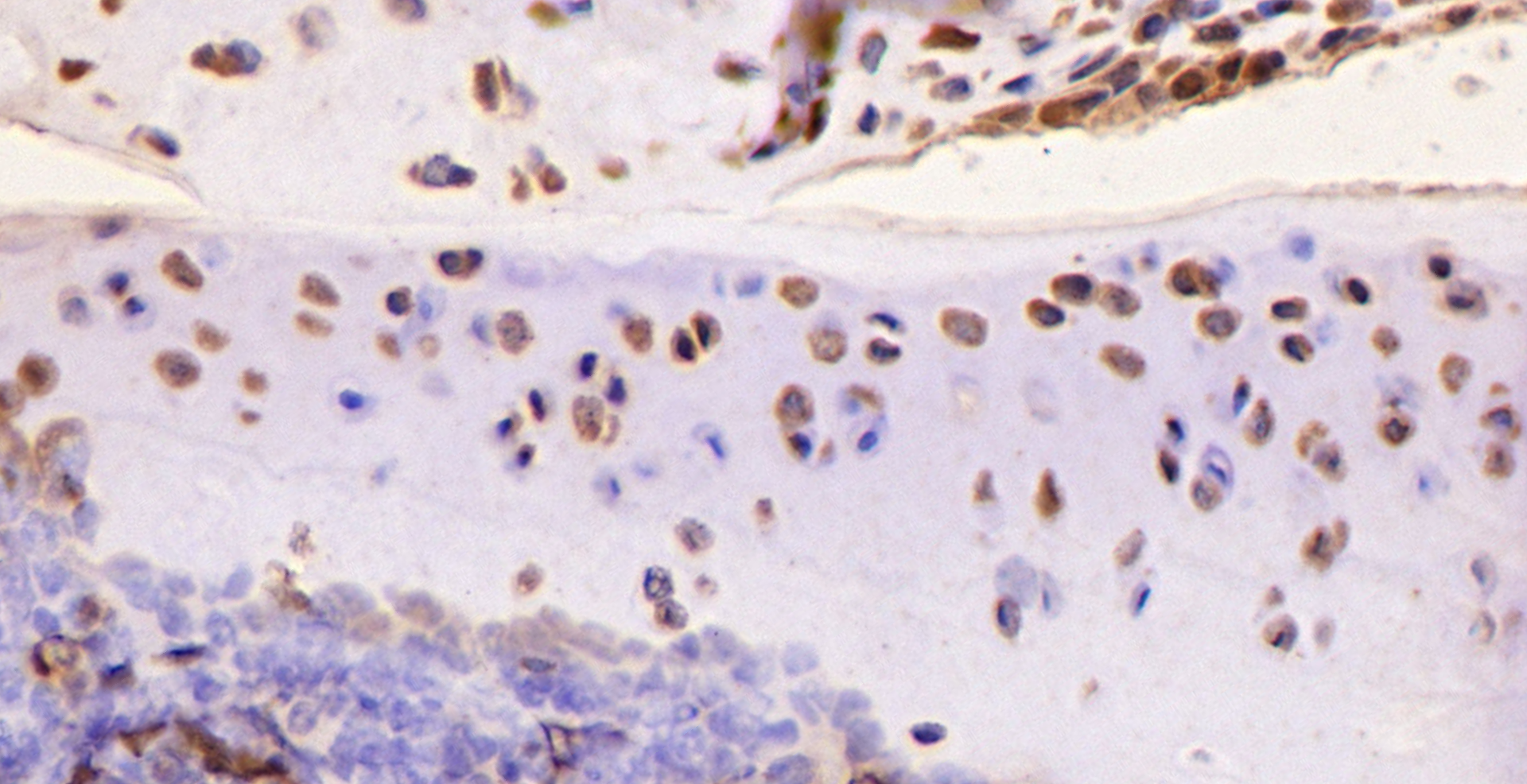

Supplement: Supplementary file 10 — Source data Fig. 6 [file 44321_2025_268_MOESM10_ESM.zip › Figure 6/6G/LTDMM Vehicle Apoe flox.tif]
