# Supplementary figures and images for: Cold exposure promotes the progression of osteoarthritis through downregulating APOE in cartilage (part 3 of 3)
Source: EMBO Mol Med. 2025 Jul 15;17(8):2137–62. doi: 10.1038/s44321-025-00268-6 (PMC12340072; doi:10.1038/s44321-025-00268-6)

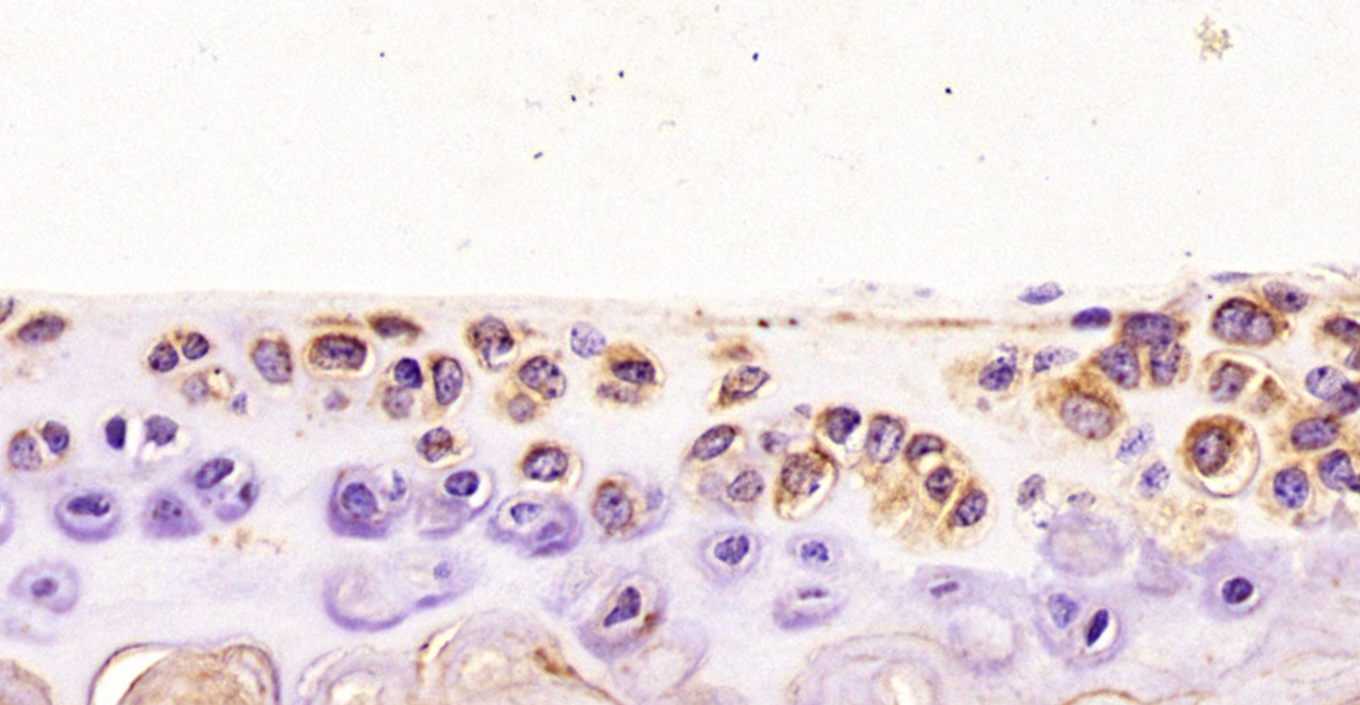

Supplement: Supplementary file 10 — Source data Fig. 6 [file 44321_2025_268_MOESM10_ESM.zip › Figure 6/6G/RTDMM Apoe +-.tif]

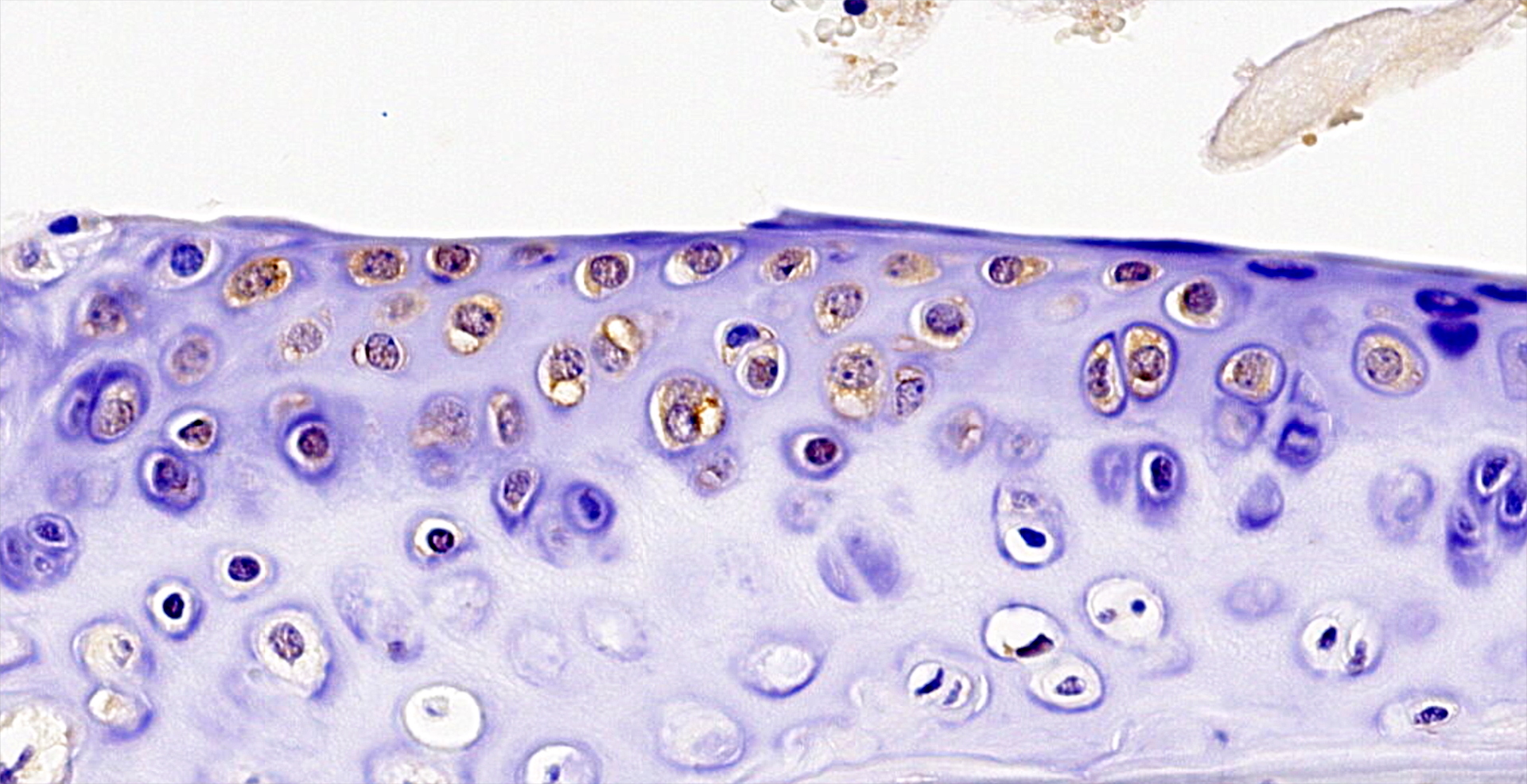

Supplement: Supplementary file 10 — Source data Fig. 6 [file 44321_2025_268_MOESM10_ESM.zip › Figure 6/6G/RTDMM Apoe flox.tif]

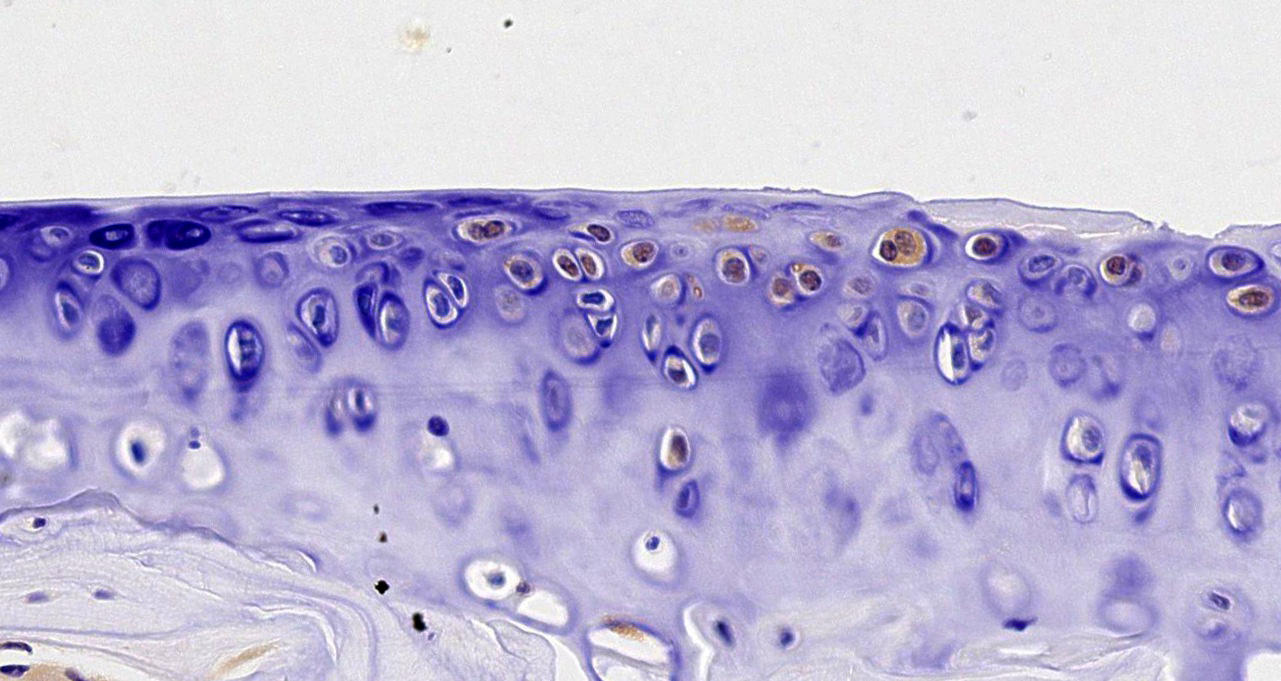

Supplement: Supplementary file 10 — Source data Fig. 6 [file 44321_2025_268_MOESM10_ESM.zip › Figure 6/6H/LTDMM RGX-104 Apoe +-.jpg]

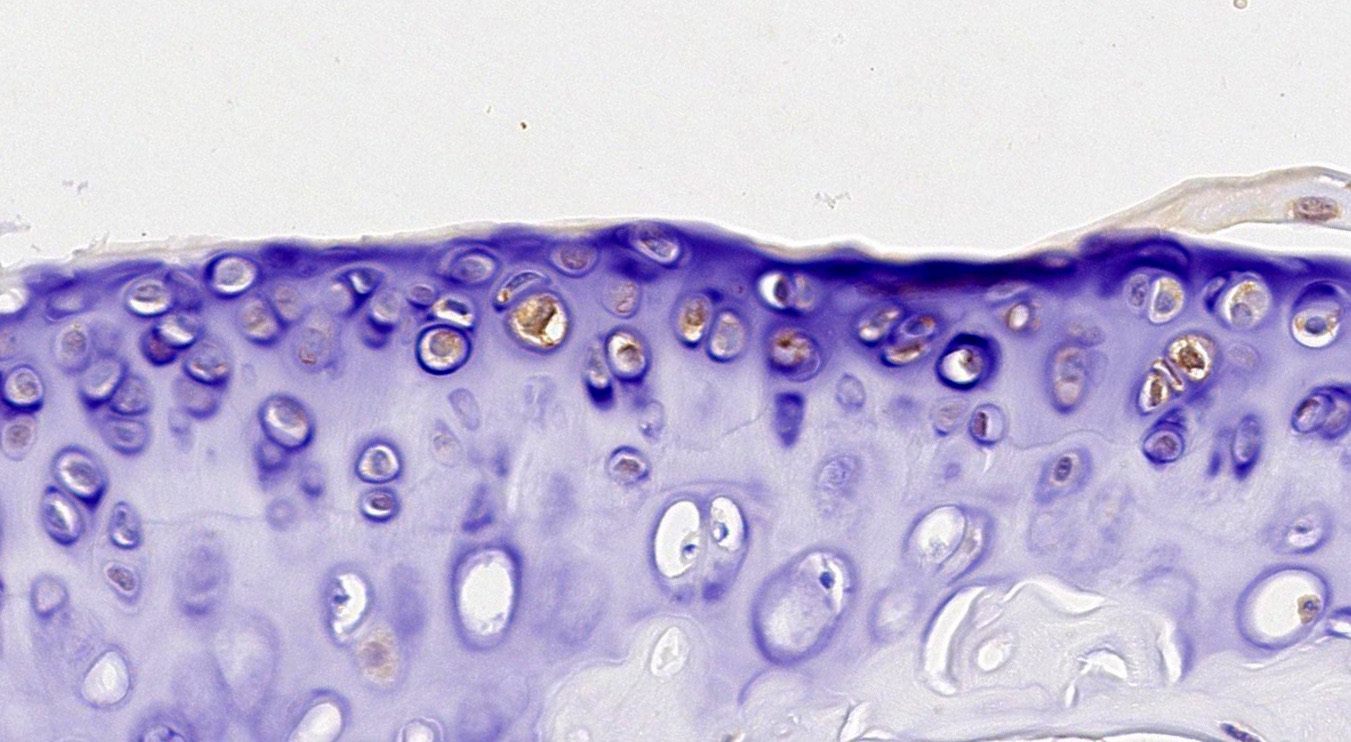

Supplement: Supplementary file 10 — Source data Fig. 6 [file 44321_2025_268_MOESM10_ESM.zip › Figure 6/6H/LTDMM RGX-104 Apoe fl.jpg]

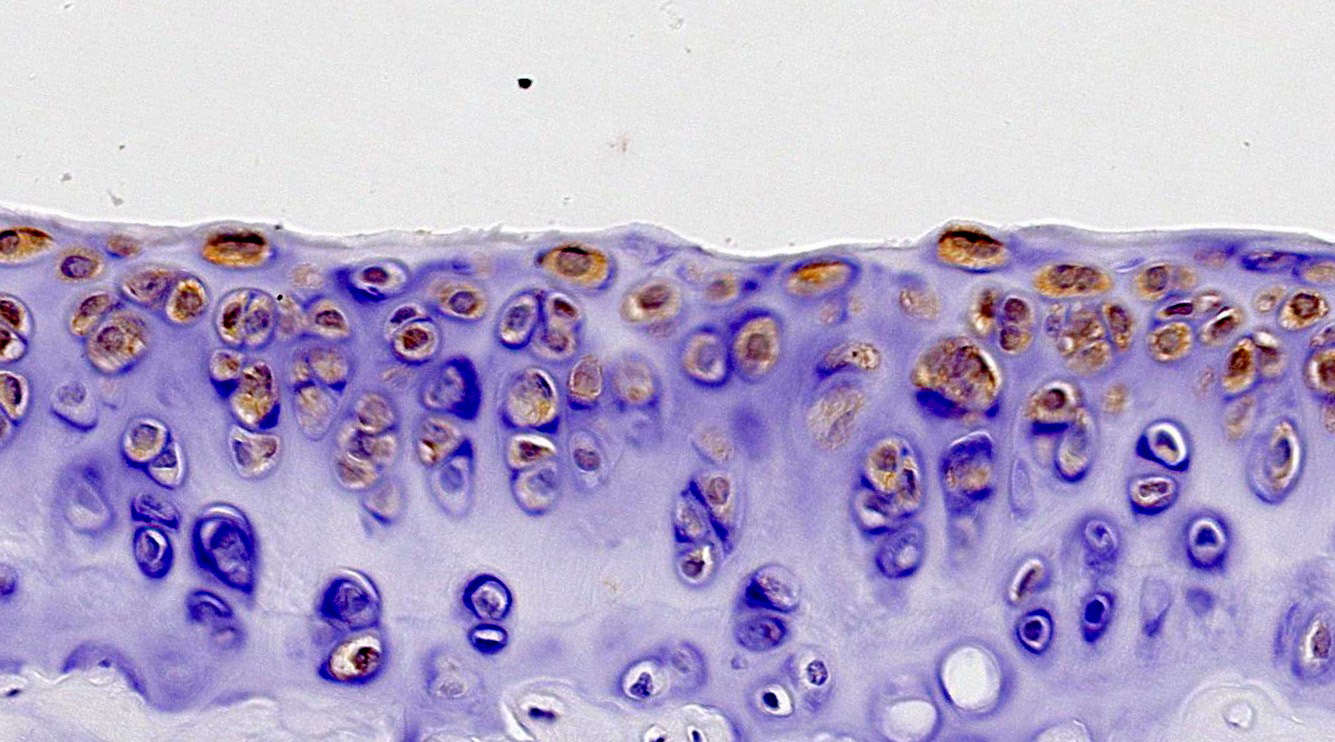

Supplement: Supplementary file 10 — Source data Fig. 6 [file 44321_2025_268_MOESM10_ESM.zip › Figure 6/6H/LTDMM vehicle Apoe +-.jpg]

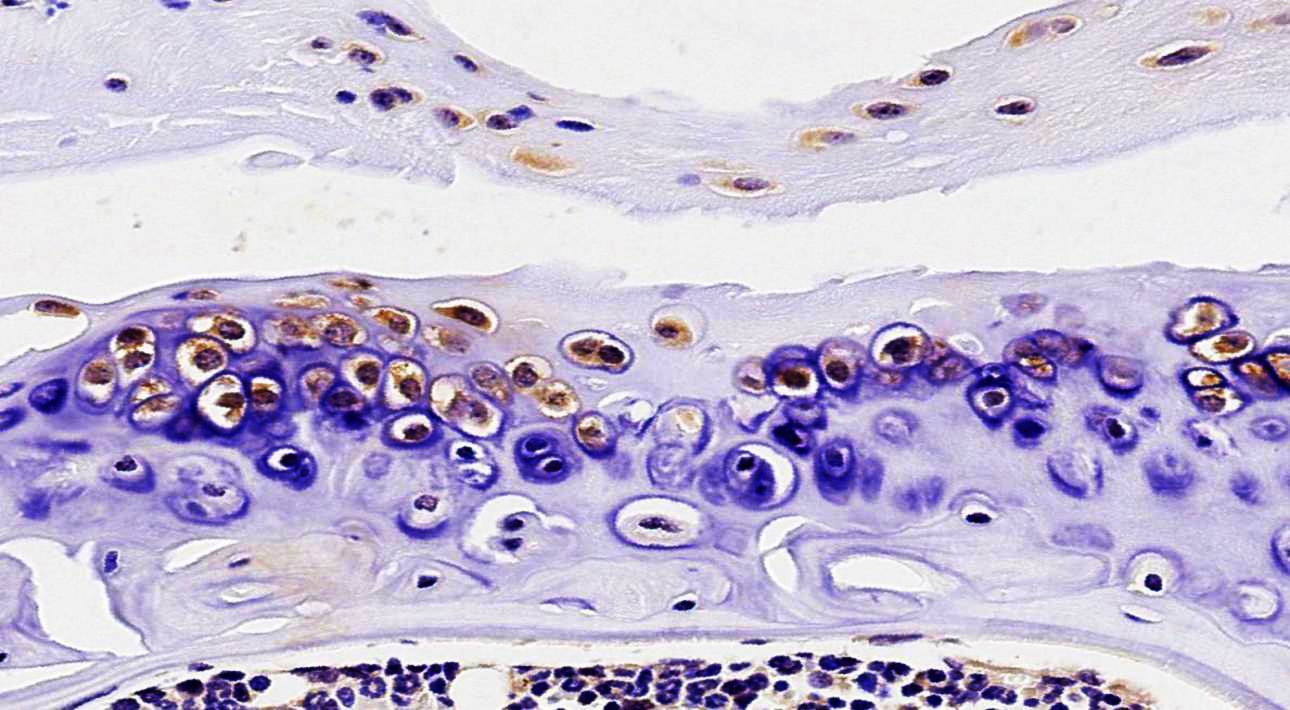

Supplement: Supplementary file 10 — Source data Fig. 6 [file 44321_2025_268_MOESM10_ESM.zip › Figure 6/6H/LTDMM vehicle Apoe fl.jpg]

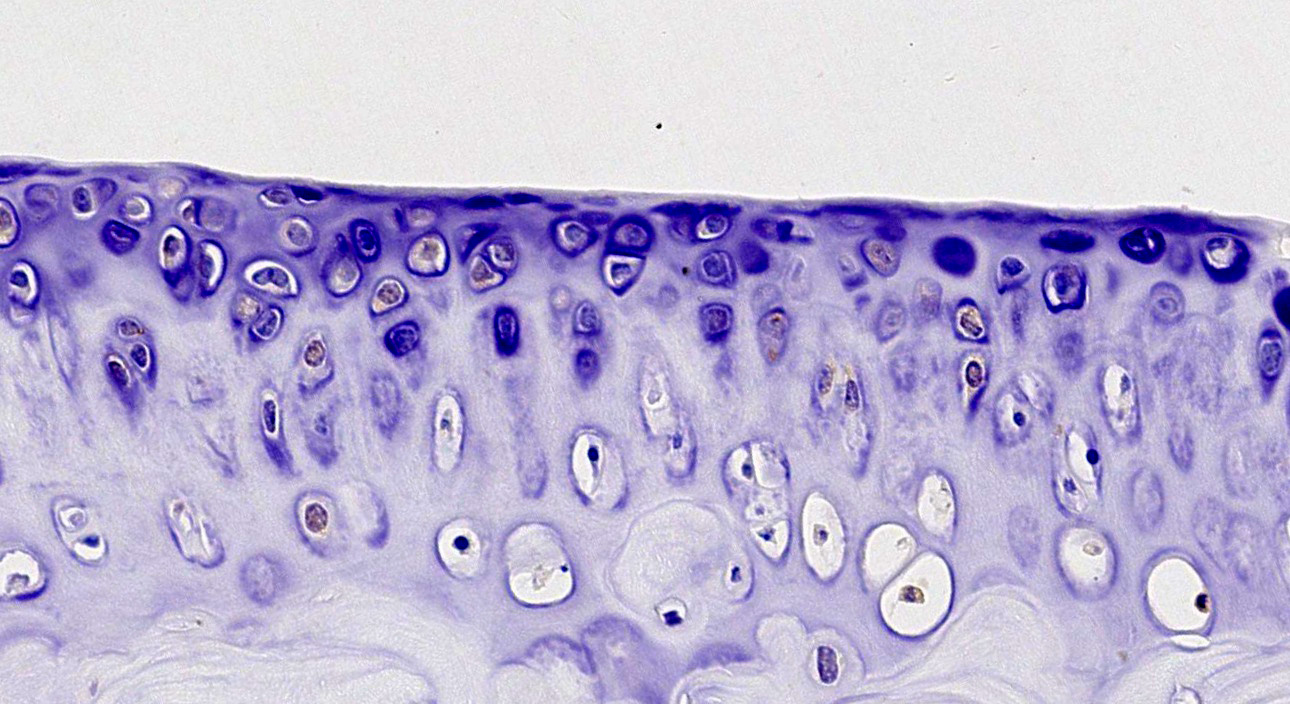

Supplement: Supplementary file 10 — Source data Fig. 6 [file 44321_2025_268_MOESM10_ESM.zip › Figure 6/6H/RTDMM Apoe fl.jpg]

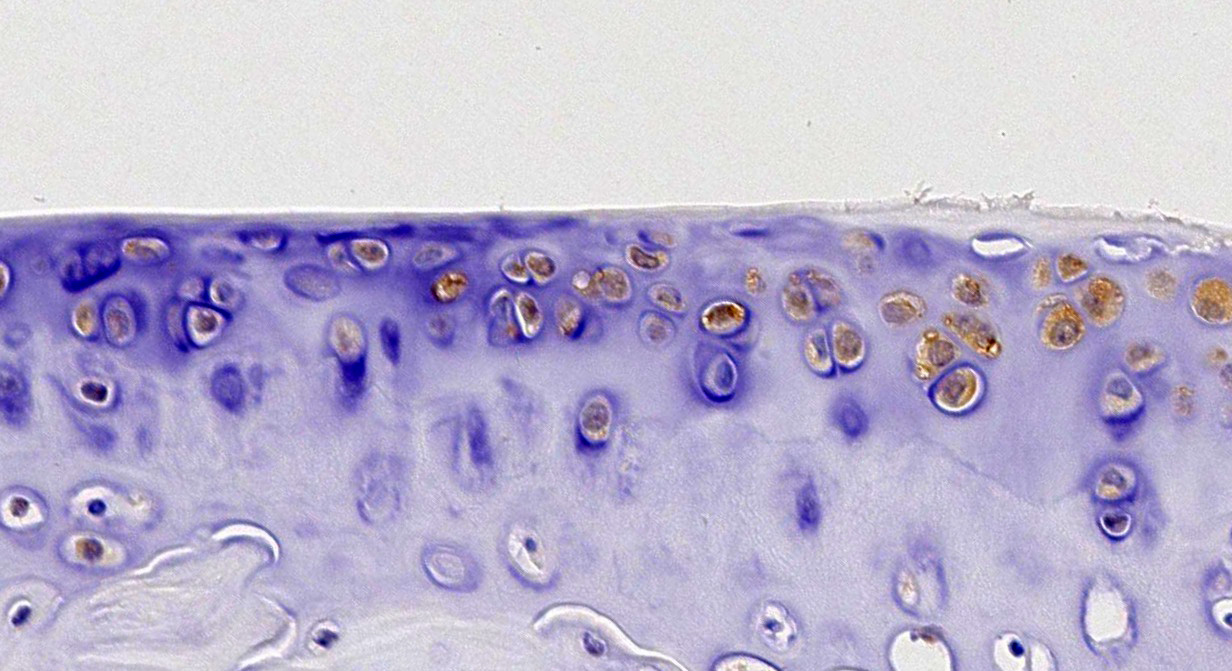

Supplement: Supplementary file 10 — Source data Fig. 6 [file 44321_2025_268_MOESM10_ESM.zip › Figure 6/6H/RTDMM Apole +-.jpg]

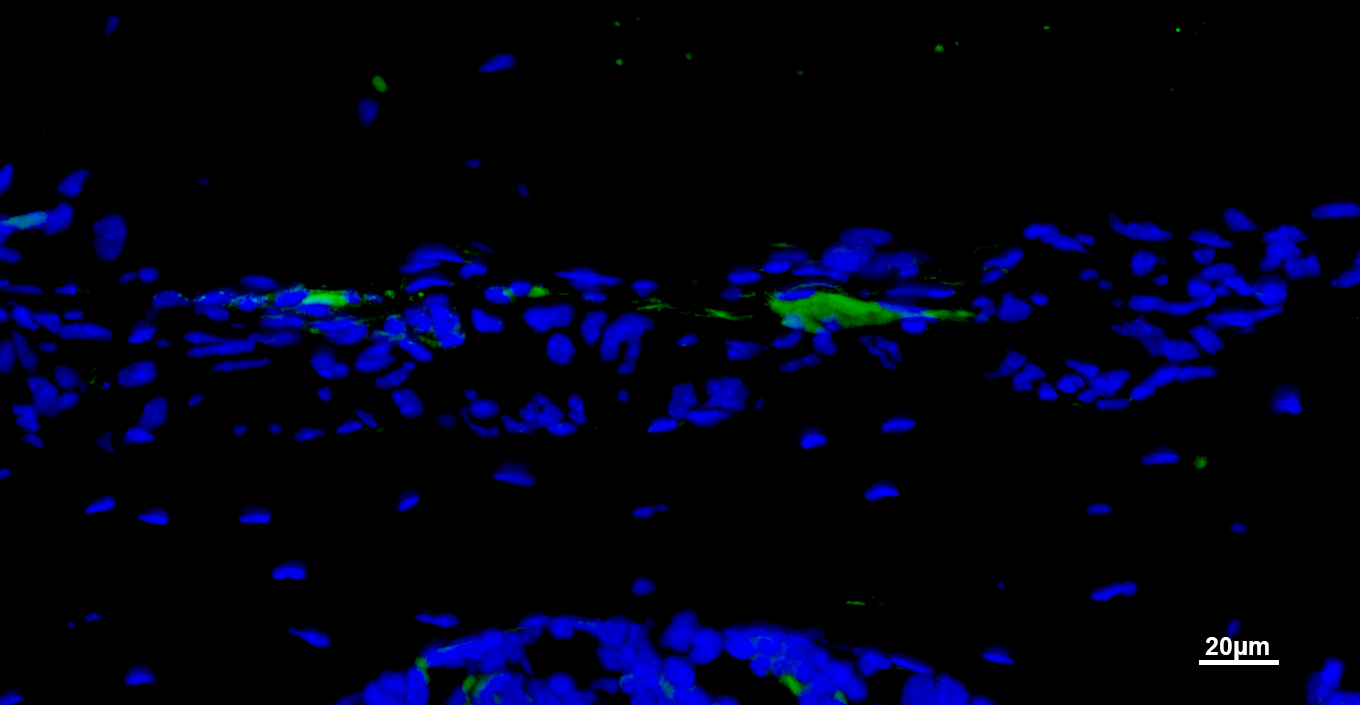

Supplement: Supplementary file 10 — Source data Fig. 6 [file 44321_2025_268_MOESM10_ESM.zip › Figure 6/6I/LTDMM RGX-104 Apoe +-.tif]

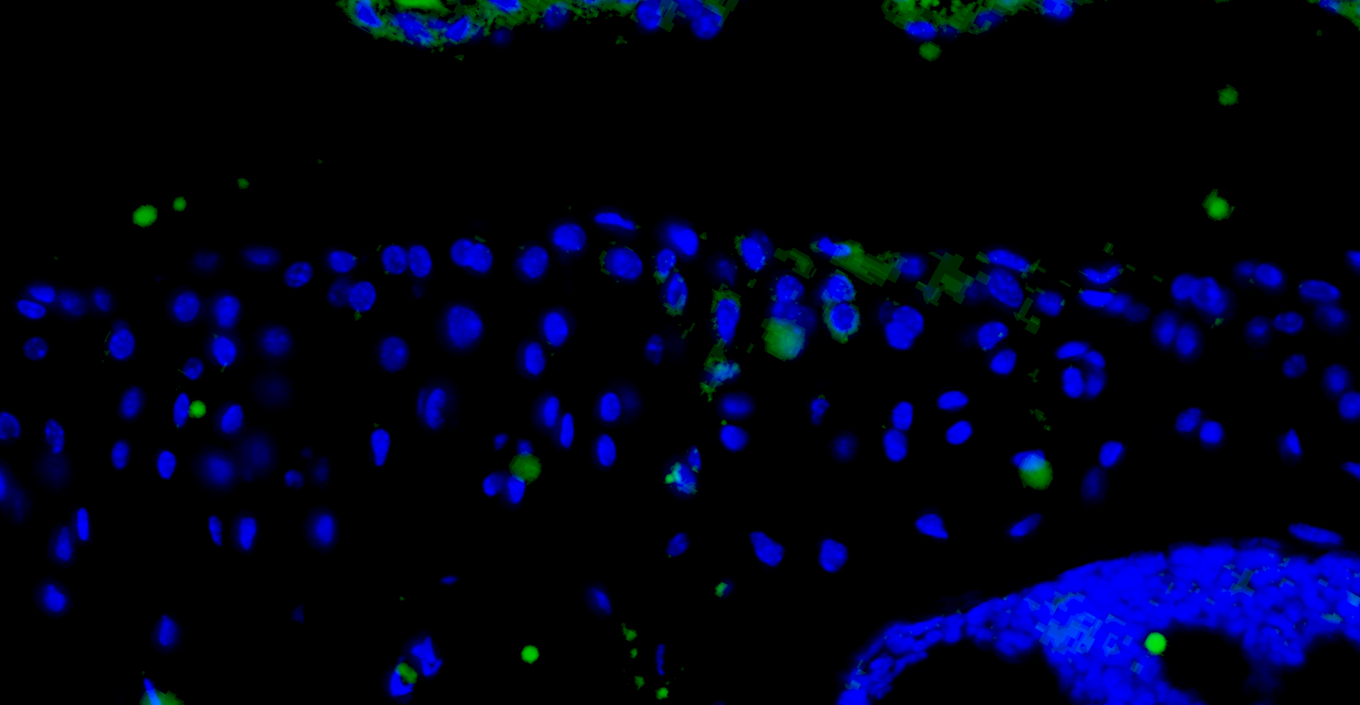

Supplement: Supplementary file 10 — Source data Fig. 6 [file 44321_2025_268_MOESM10_ESM.zip › Figure 6/6I/LTDMM RGX-104 Apoe flox.tif]

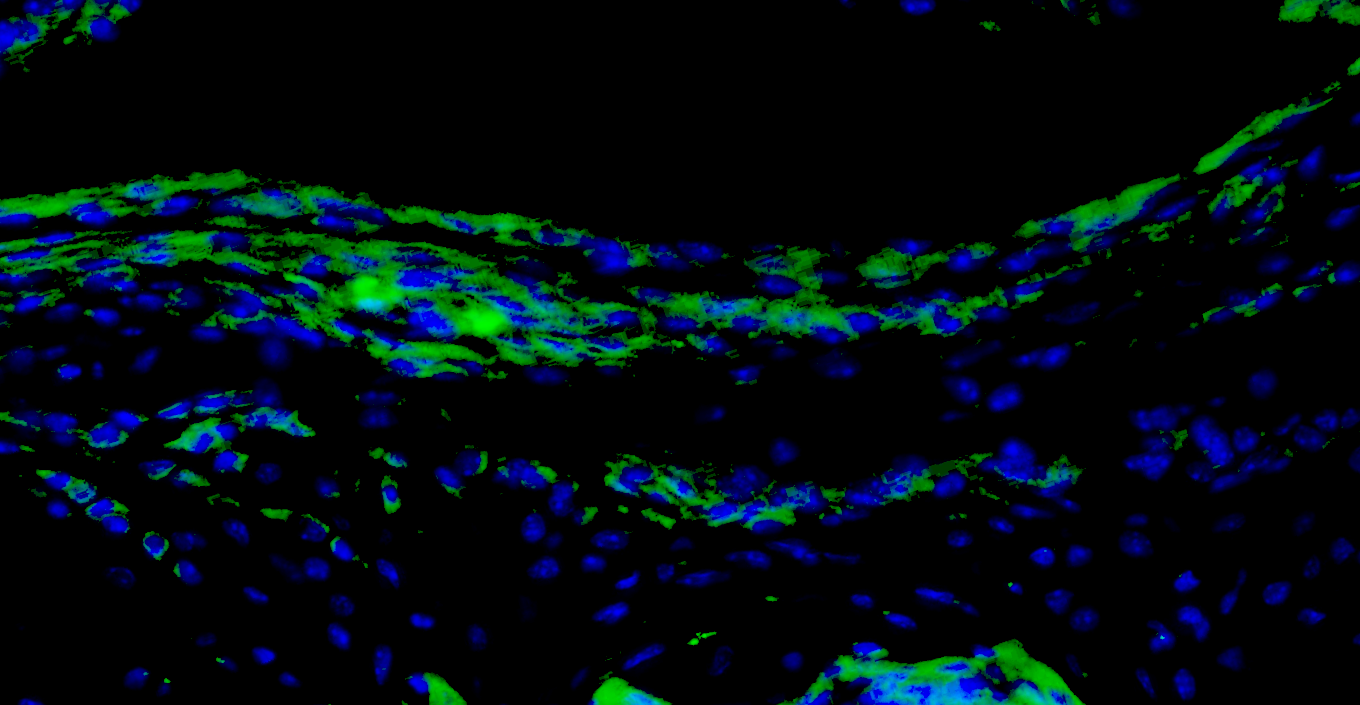

Supplement: Supplementary file 10 — Source data Fig. 6 [file 44321_2025_268_MOESM10_ESM.zip › Figure 6/6I/LTDMM Vehicle Apoe +-.tif]

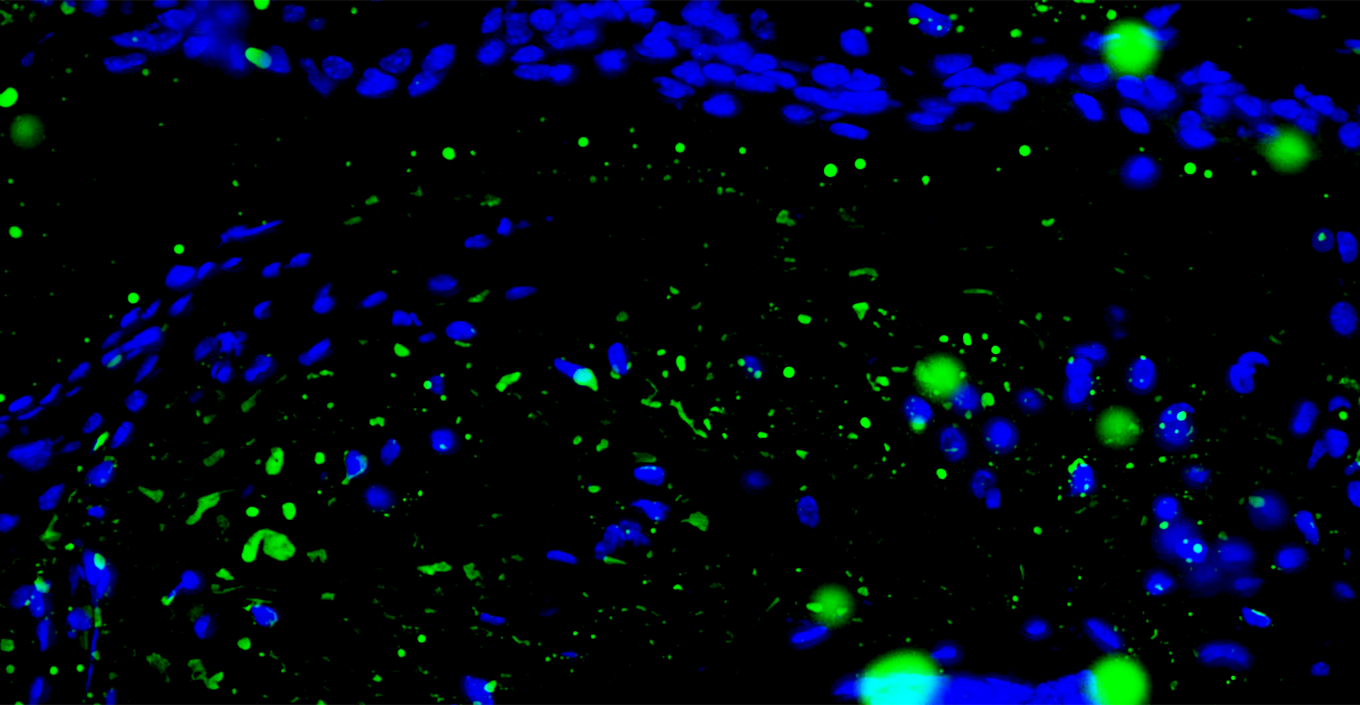

Supplement: Supplementary file 10 — Source data Fig. 6 [file 44321_2025_268_MOESM10_ESM.zip › Figure 6/6I/LTDMM Vehicle Apoe flox.tif]

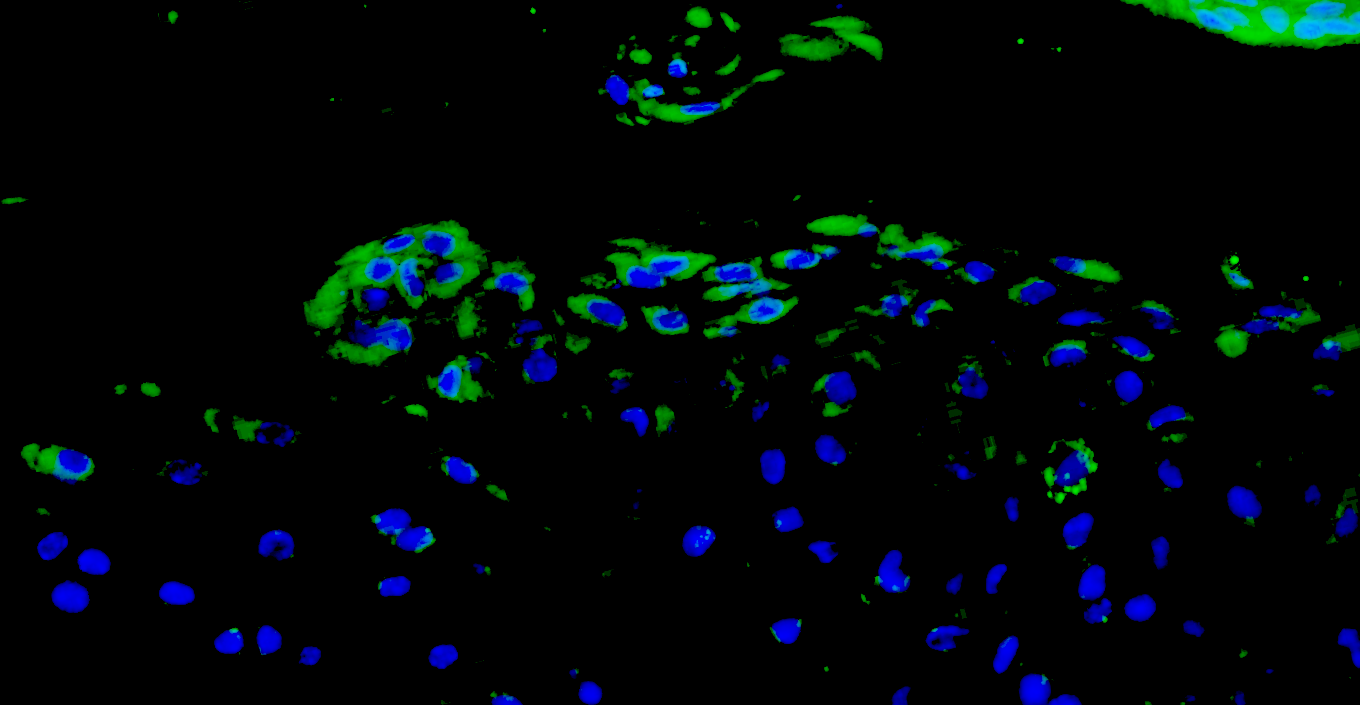

Supplement: Supplementary file 10 — Source data Fig. 6 [file 44321_2025_268_MOESM10_ESM.zip › Figure 6/6I/RTDMM Apoe +-.tif]

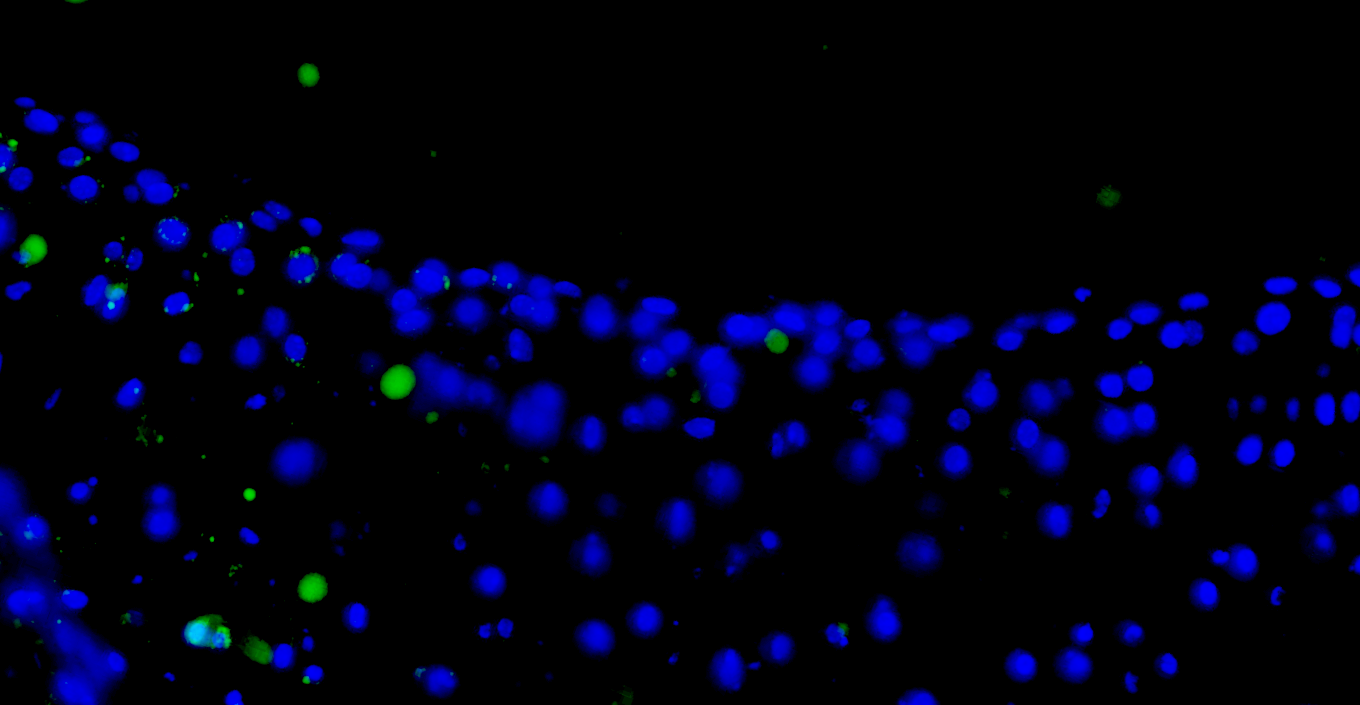

Supplement: Supplementary file 10 — Source data Fig. 6 [file 44321_2025_268_MOESM10_ESM.zip › Figure 6/6I/RTDMM Apoe flox.tif]

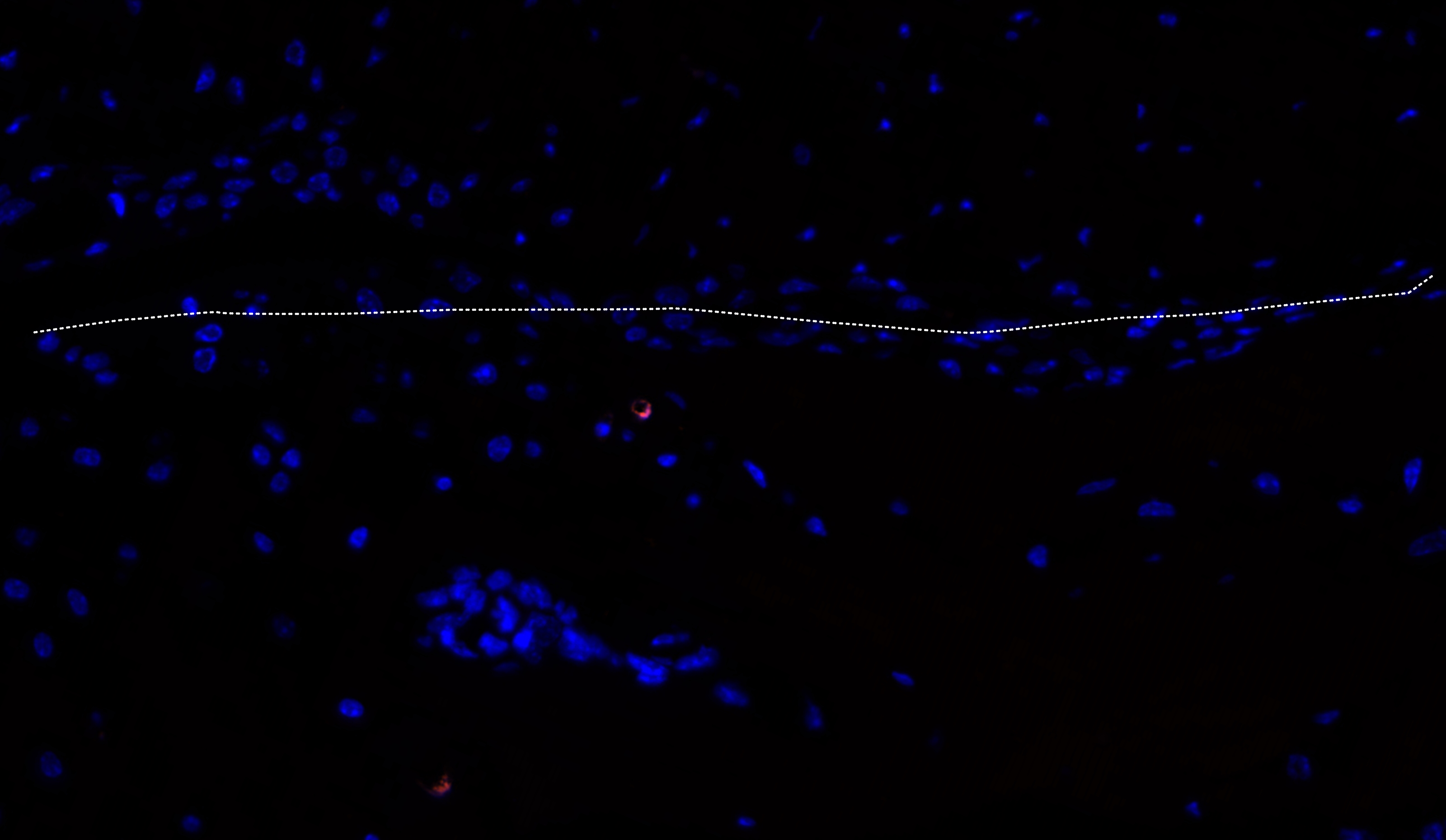

Supplement: Supplementary file 10 — Source data Fig. 6 [file 44321_2025_268_MOESM10_ESM.zip › Figure 6/6J/LTDMM RGX-104 Apoe +-.tif]

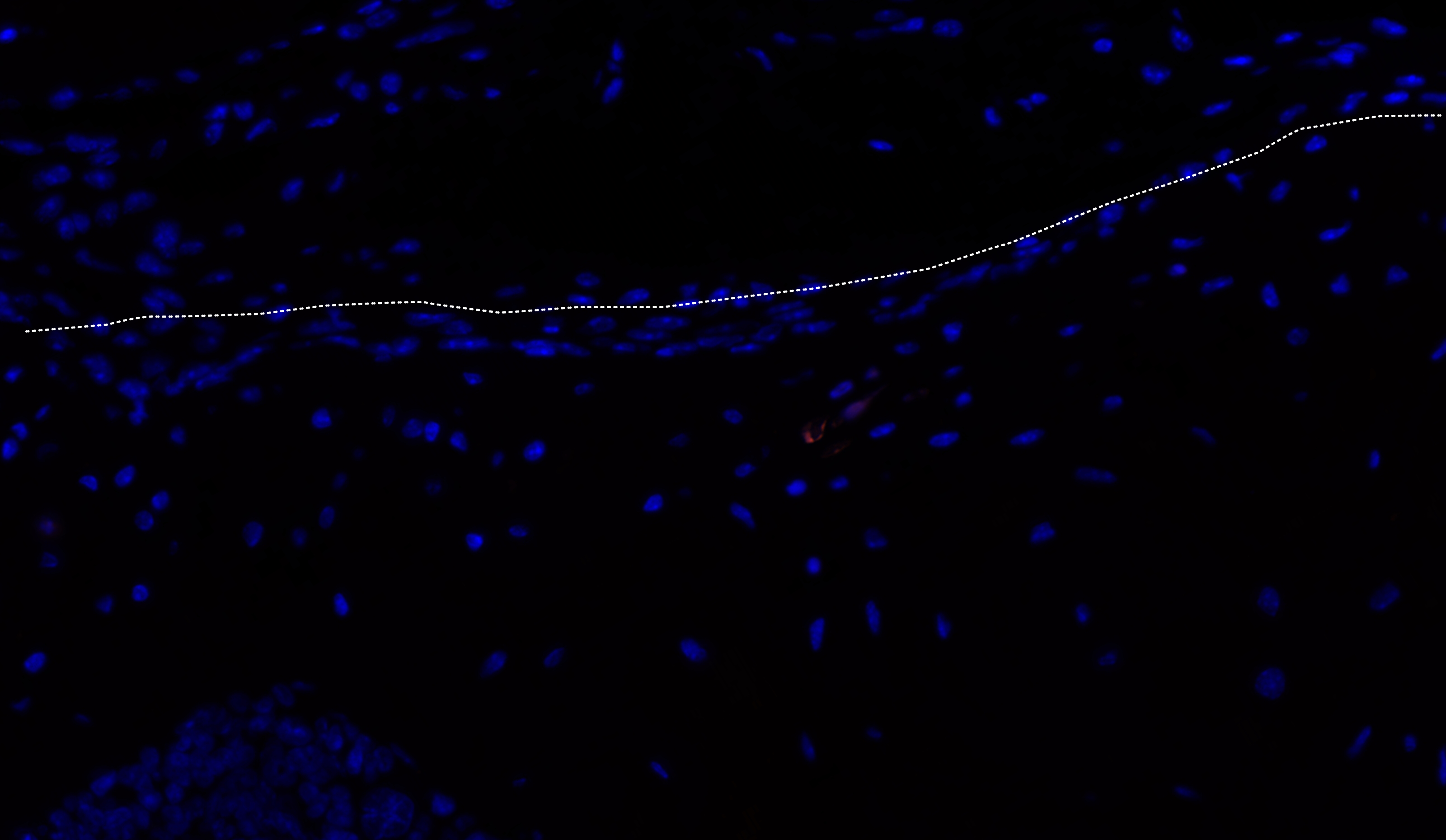

Supplement: Supplementary file 10 — Source data Fig. 6 [file 44321_2025_268_MOESM10_ESM.zip › Figure 6/6J/LTDMM RGX-104 Apoe flox.tif]

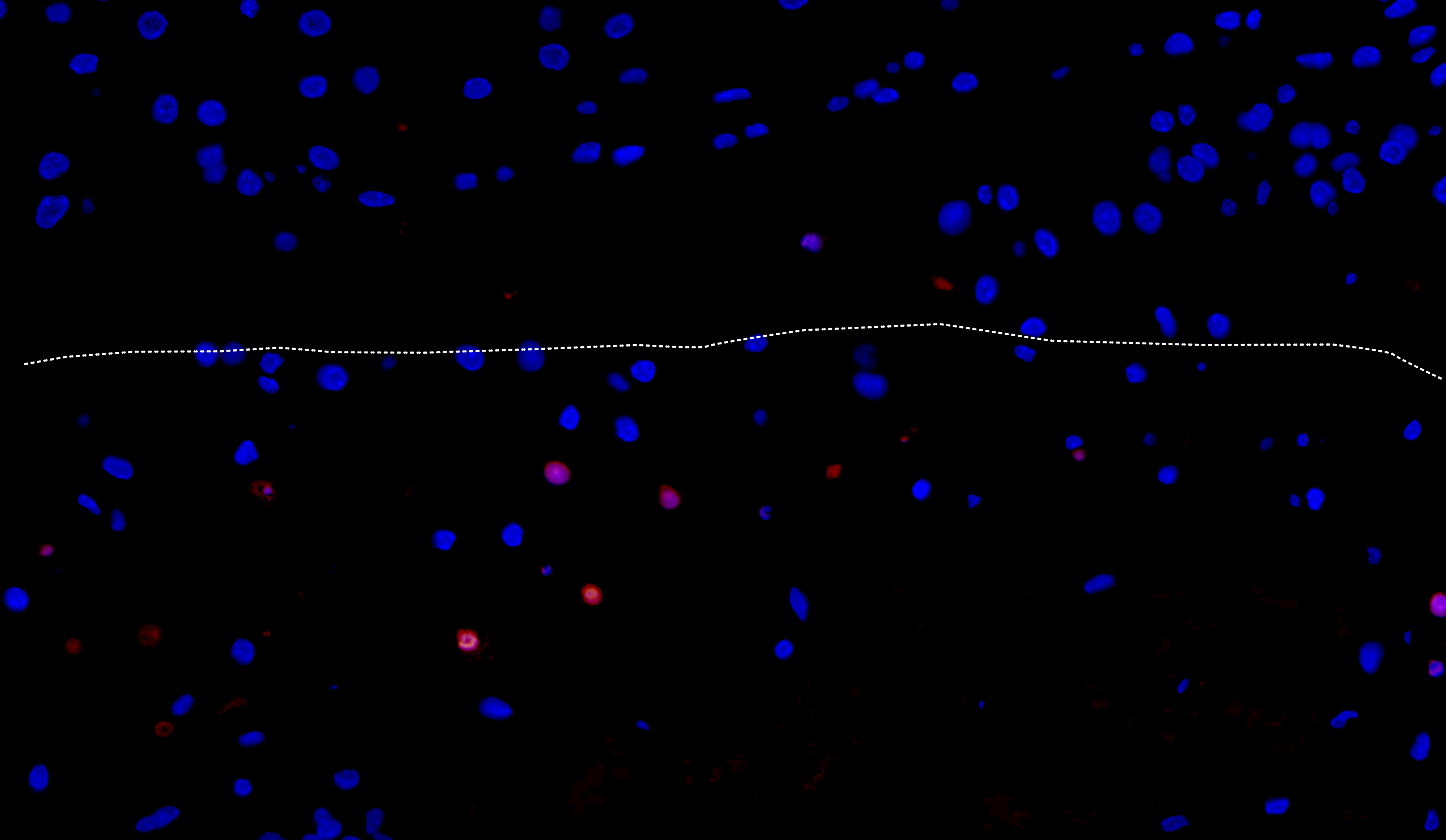

Supplement: Supplementary file 10 — Source data Fig. 6 [file 44321_2025_268_MOESM10_ESM.zip › Figure 6/6J/LTDMM Vehicle Apoe +-.tif]

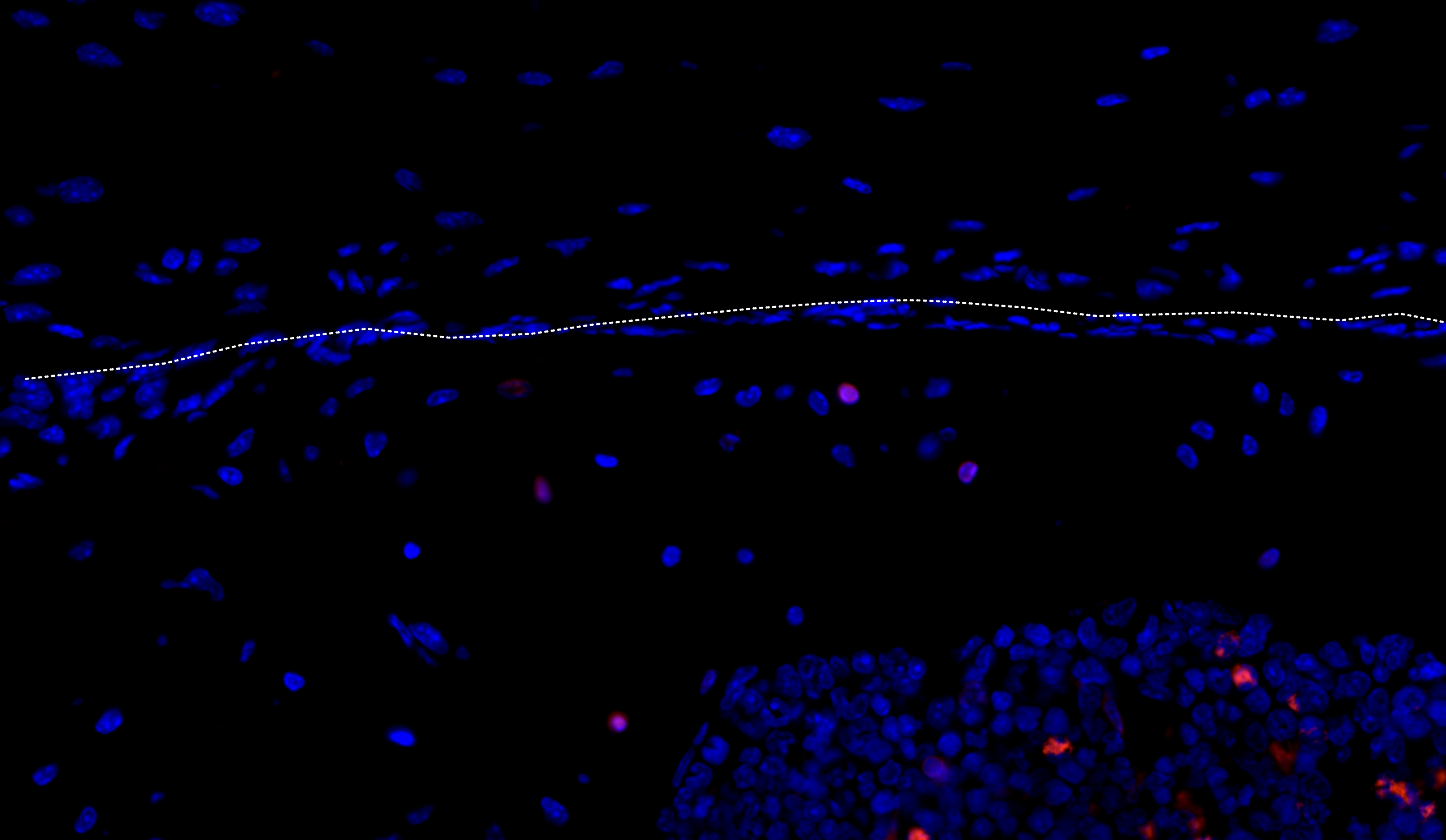

Supplement: Supplementary file 10 — Source data Fig. 6 [file 44321_2025_268_MOESM10_ESM.zip › Figure 6/6J/LTDMM Vehicle Apoe flox.tif]

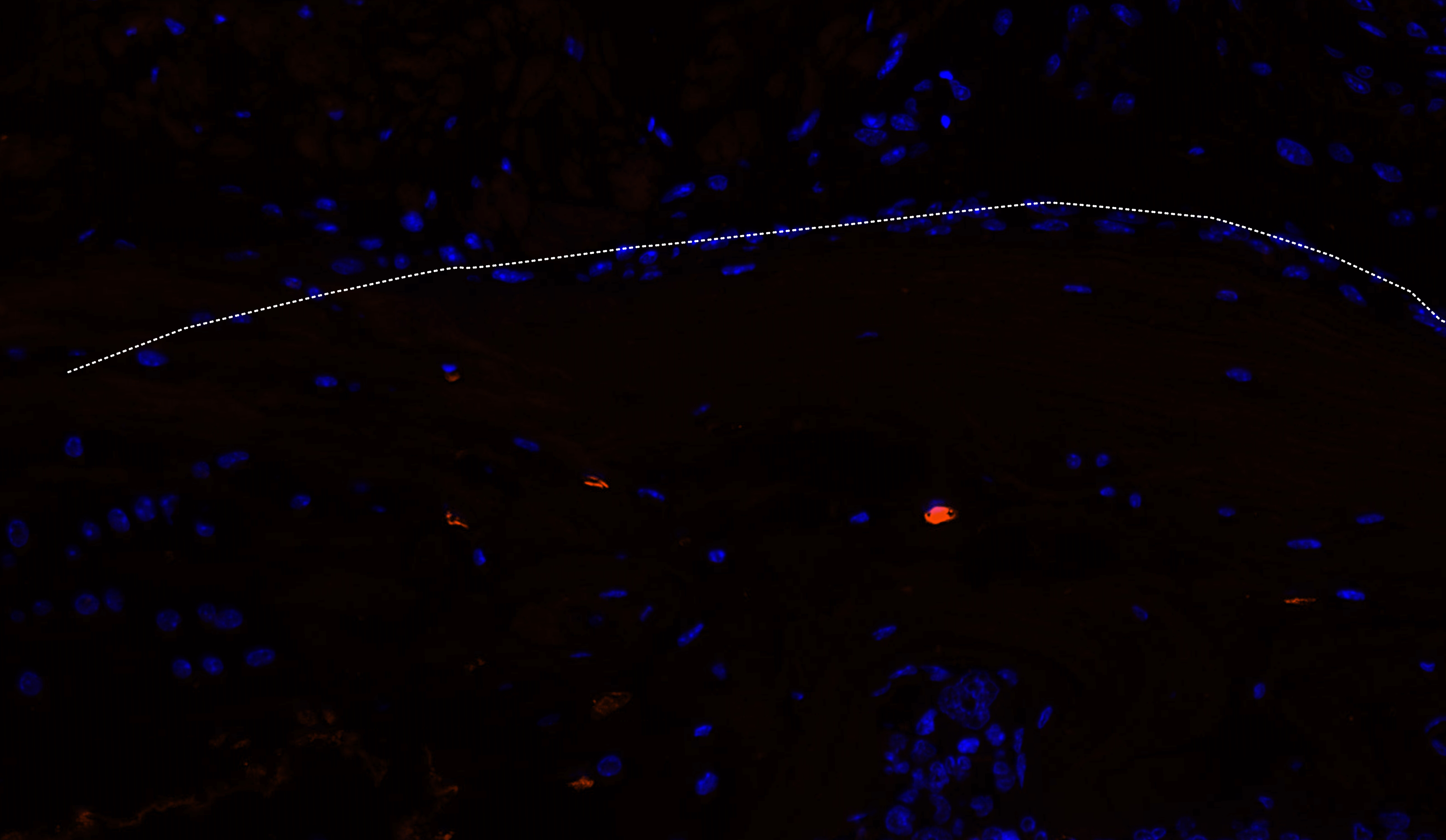

Supplement: Supplementary file 10 — Source data Fig. 6 [file 44321_2025_268_MOESM10_ESM.zip › Figure 6/6J/RTDMM Apoe +-.tif]

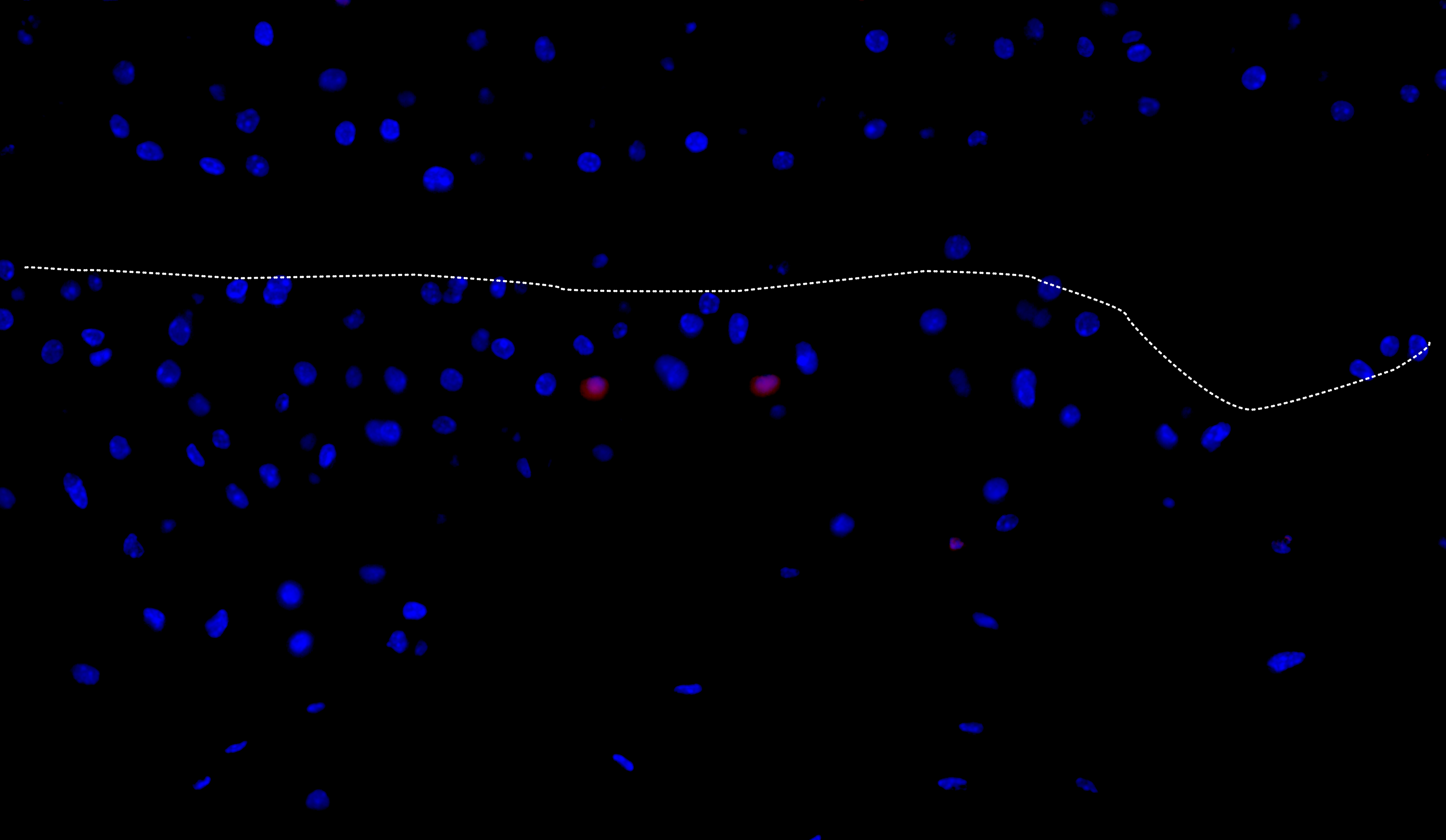

Supplement: Supplementary file 10 — Source data Fig. 6 [file 44321_2025_268_MOESM10_ESM.zip › Figure 6/6J/RTDMM Apoe flox.tif]

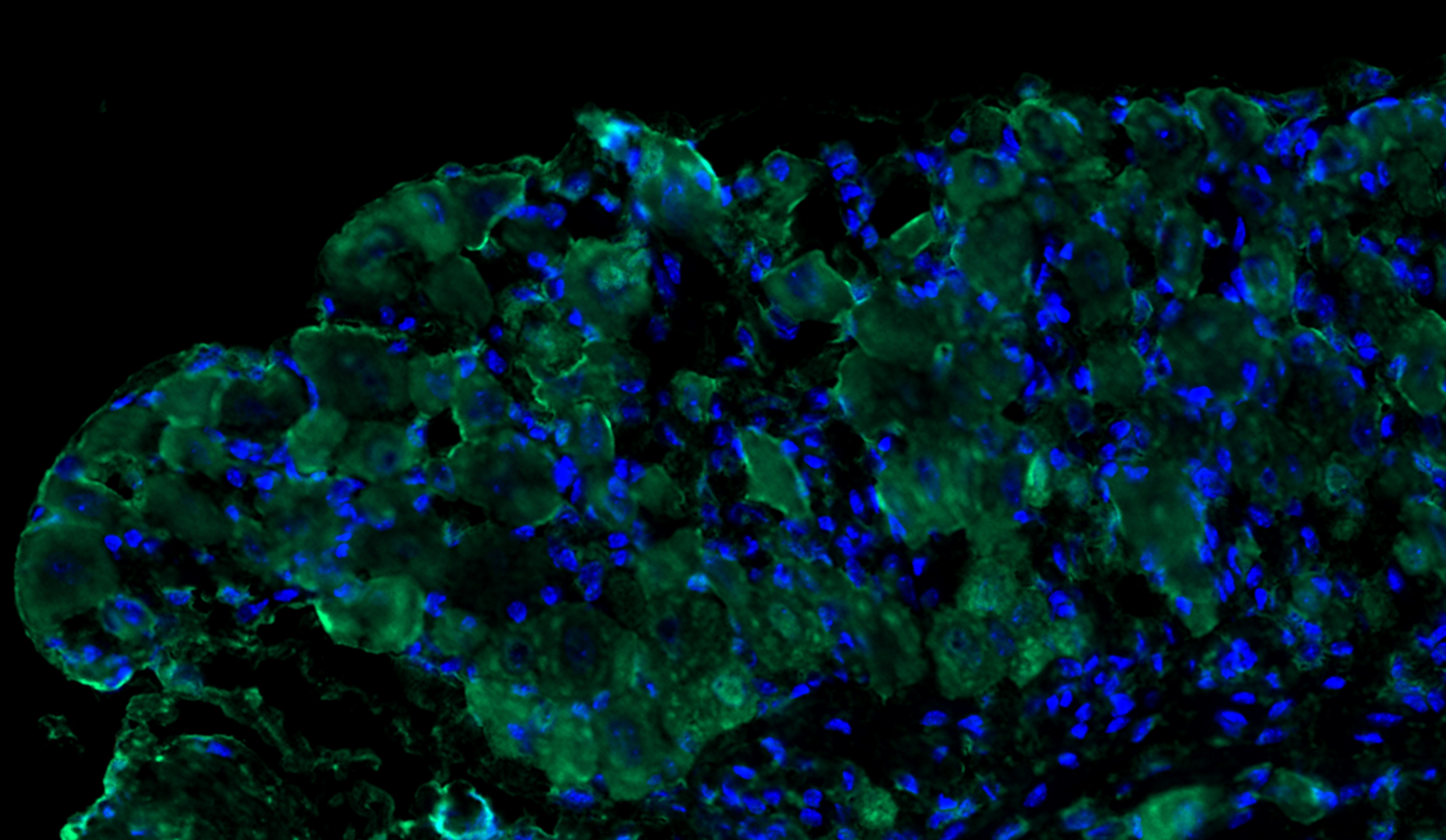

Supplement: Supplementary file 11 — Figure EV3 Source Data [file 44321_2025_268_MOESM11_ESM.zip › Figure EV3/EV3A/Apoe cko.jpg]

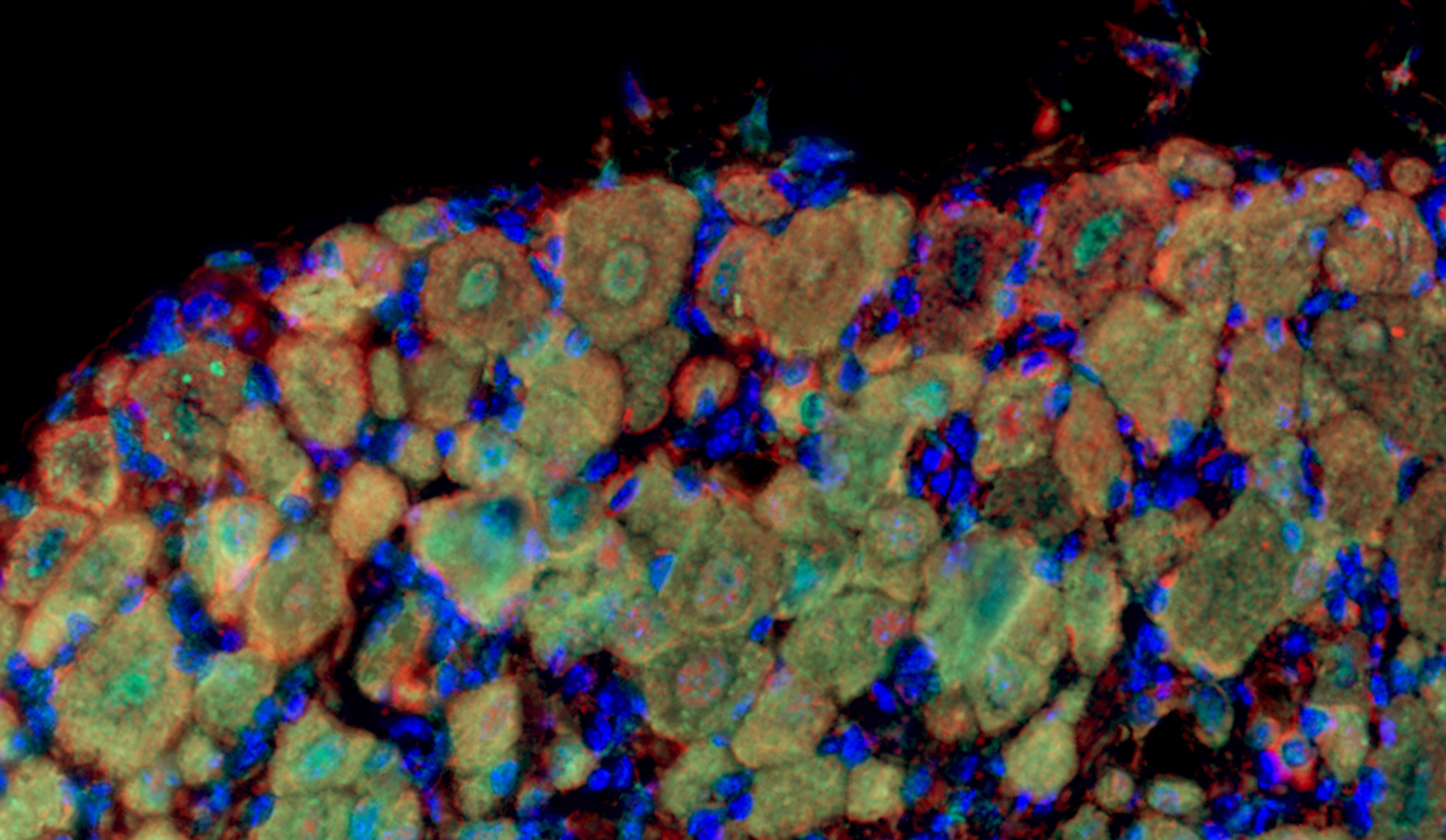

Supplement: Supplementary file 11 — Figure EV3 Source Data [file 44321_2025_268_MOESM11_ESM.zip › Figure EV3/EV3A/Apoe fl.jpg]

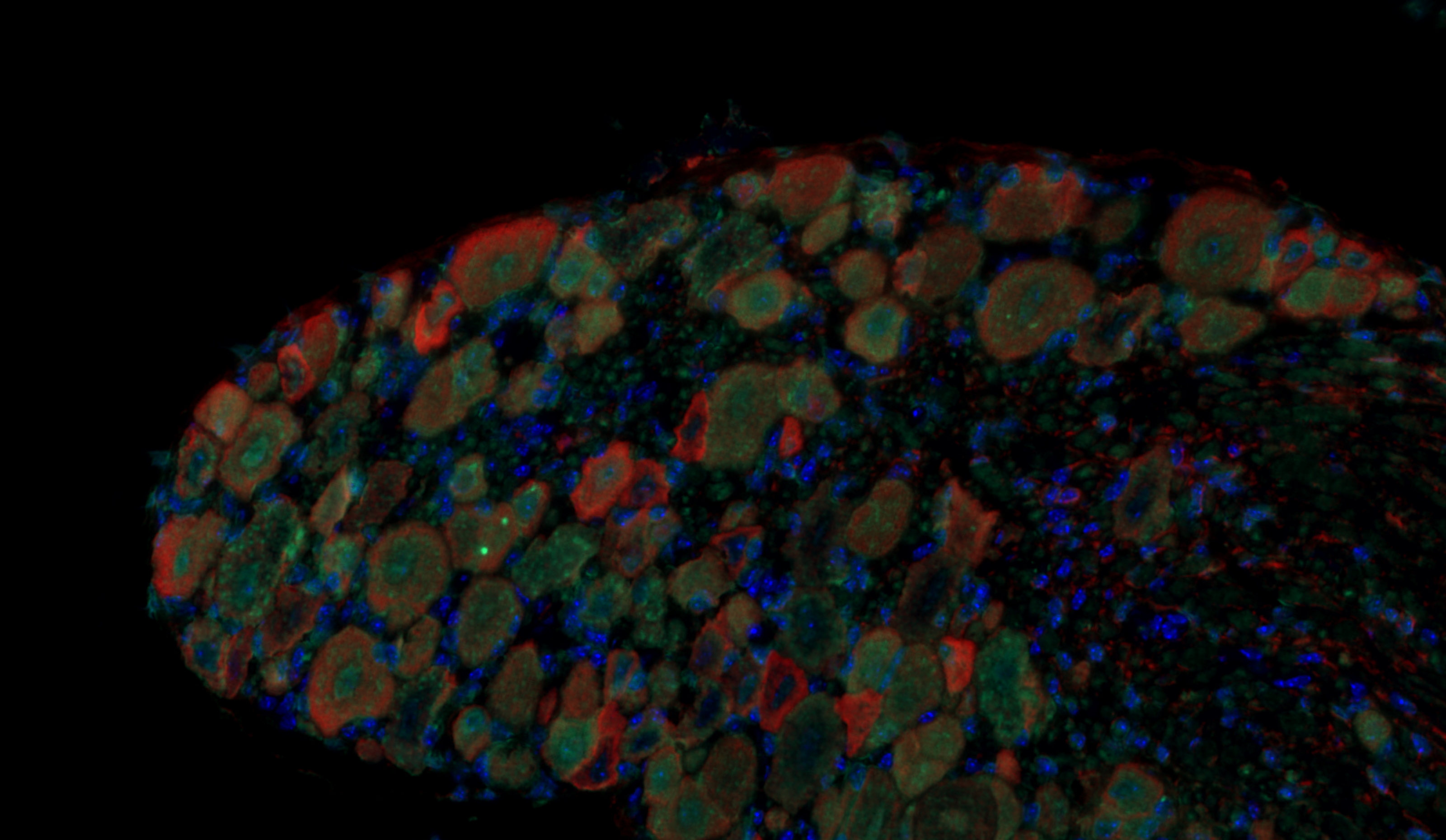

Supplement: Supplementary file 11 — Figure EV3 Source Data [file 44321_2025_268_MOESM11_ESM.zip › Figure EV3/EV3G/NAV1.7 Apoe cko DMM.jpg]

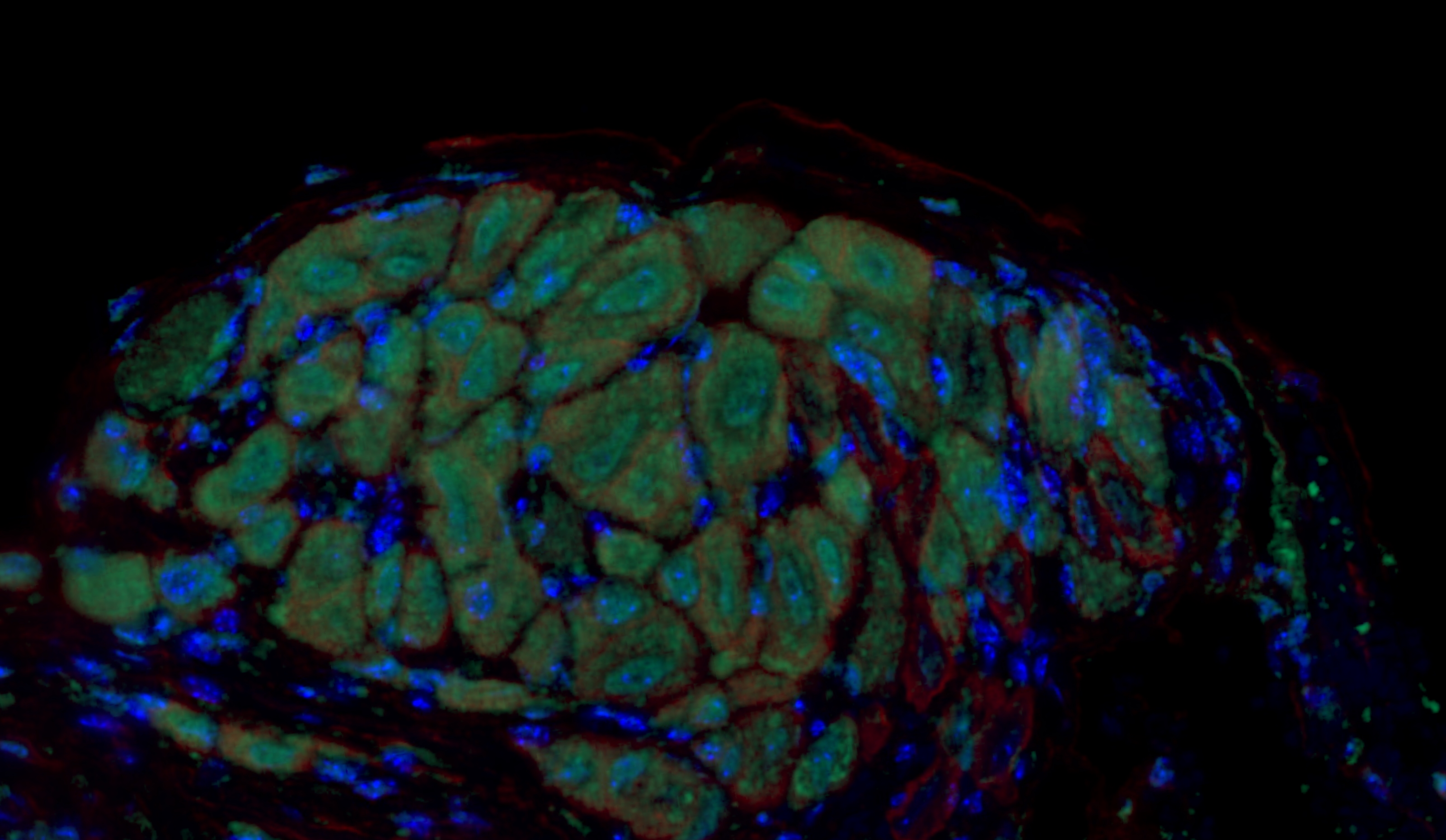

Supplement: Supplementary file 11 — Figure EV3 Source Data [file 44321_2025_268_MOESM11_ESM.zip › Figure EV3/EV3G/NAV1.7 Apoe cko Sham.jpg]

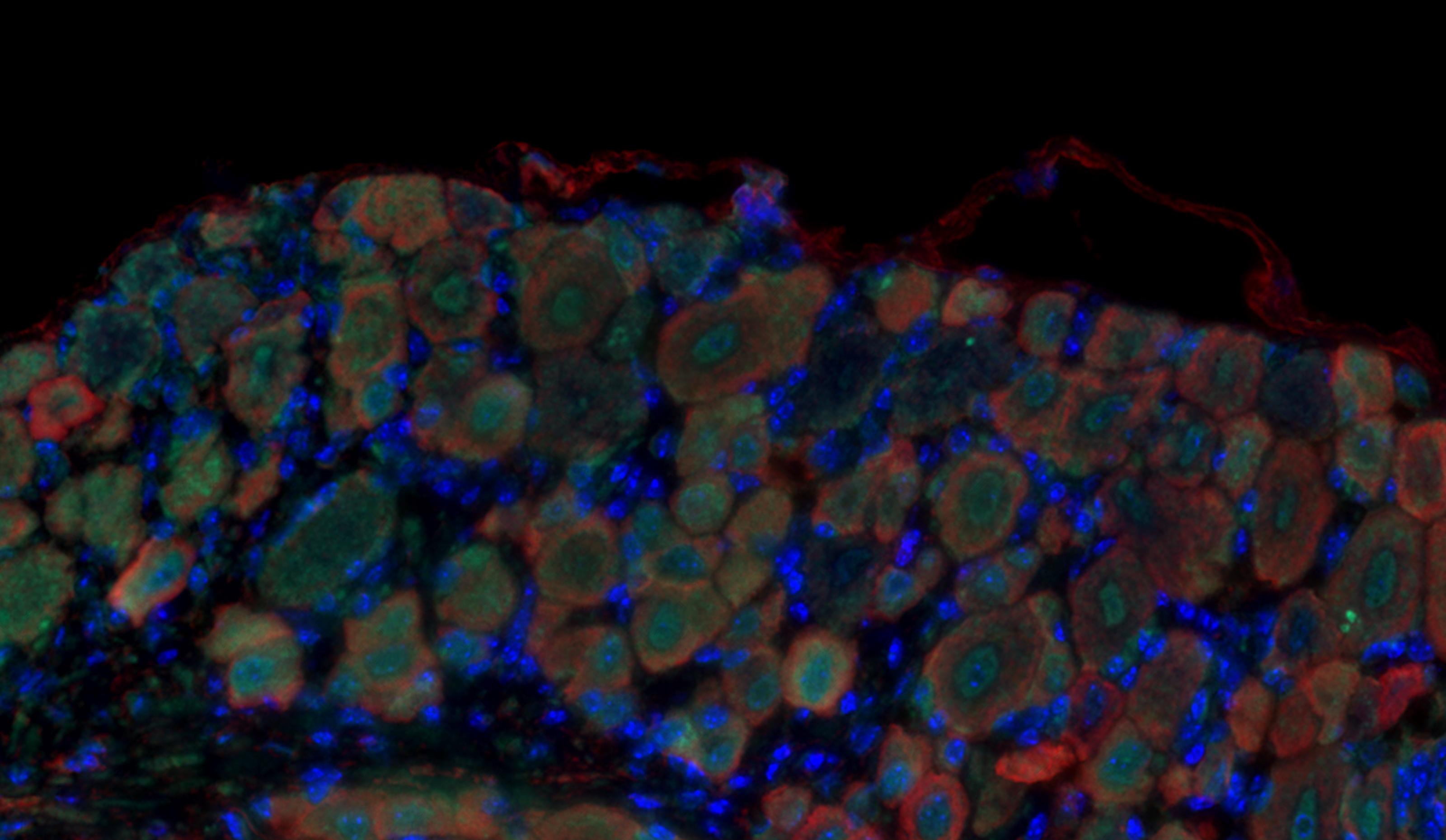

Supplement: Supplementary file 11 — Figure EV3 Source Data [file 44321_2025_268_MOESM11_ESM.zip › Figure EV3/EV3G/NAV1.7 Apoe fl DMM.jpg]

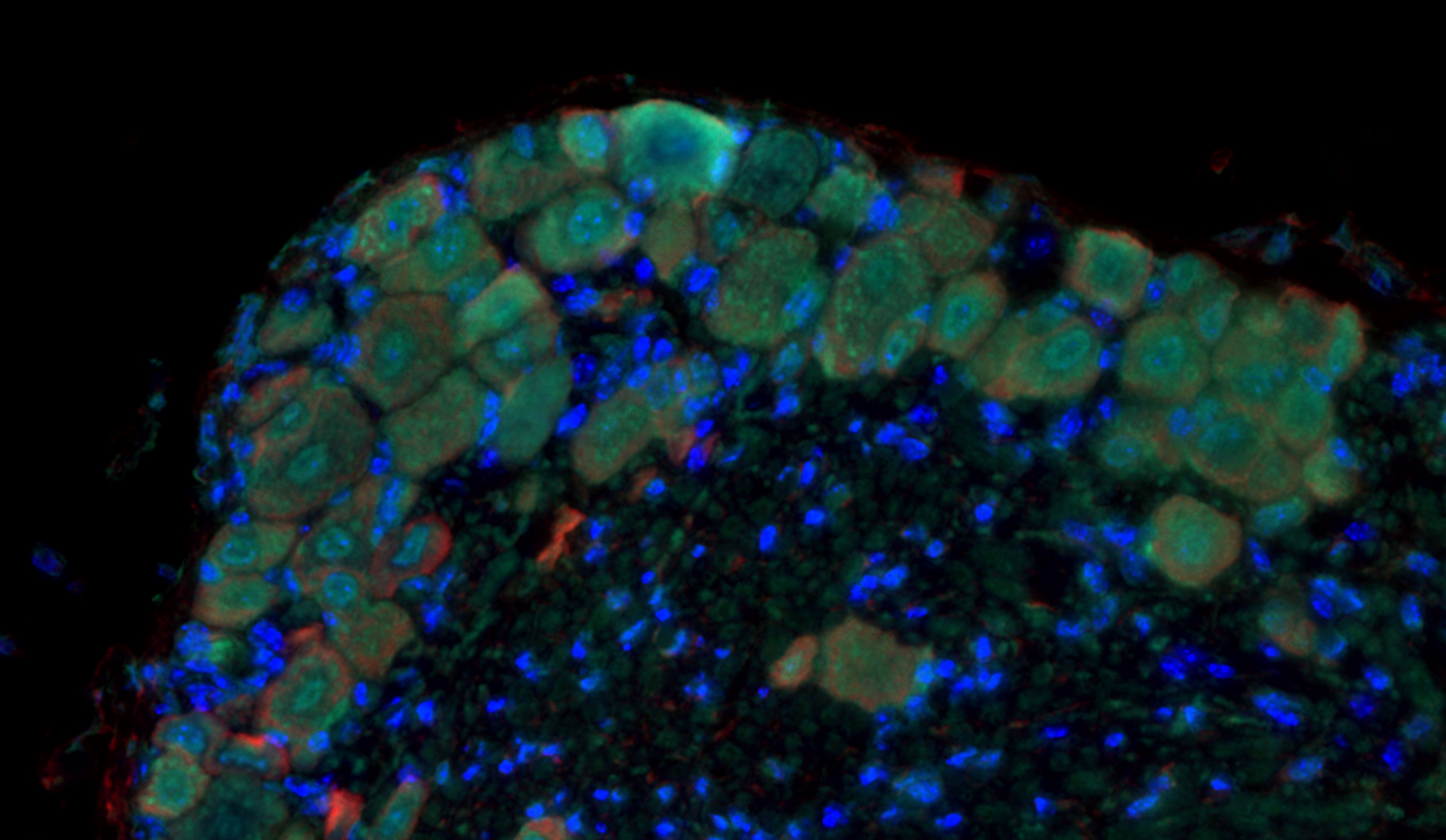

Supplement: Supplementary file 11 — Figure EV3 Source Data [file 44321_2025_268_MOESM11_ESM.zip › Figure EV3/EV3G/NAV1.7 Apoe fl Sham.jpg]

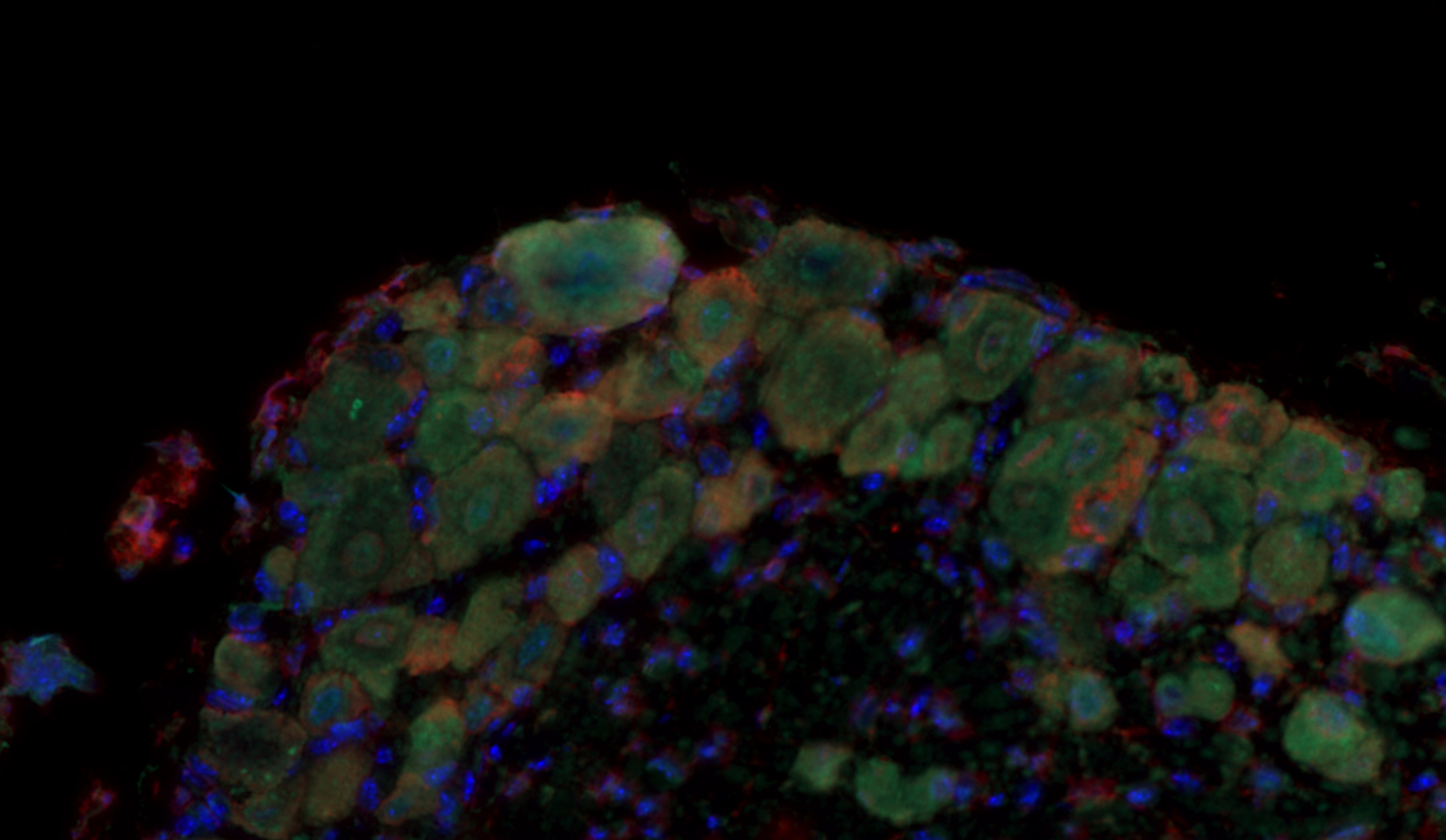

Supplement: Supplementary file 11 — Figure EV3 Source Data [file 44321_2025_268_MOESM11_ESM.zip › Figure EV3/EV3G/TAC1 Apoe cko DMM.jpg]

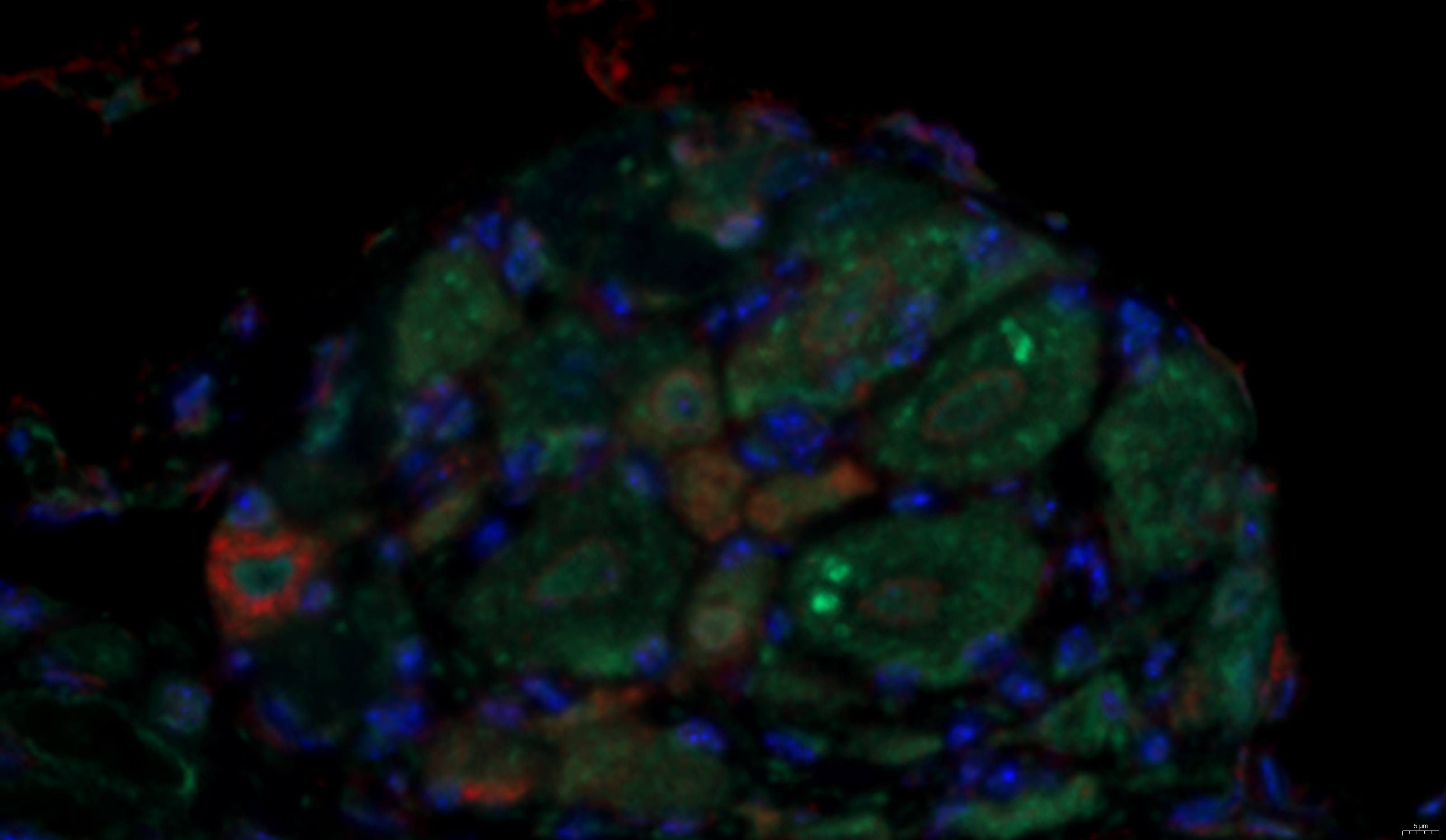

Supplement: Supplementary file 11 — Figure EV3 Source Data [file 44321_2025_268_MOESM11_ESM.zip › Figure EV3/EV3G/TAC1 Apoe cko Sham.jpg]

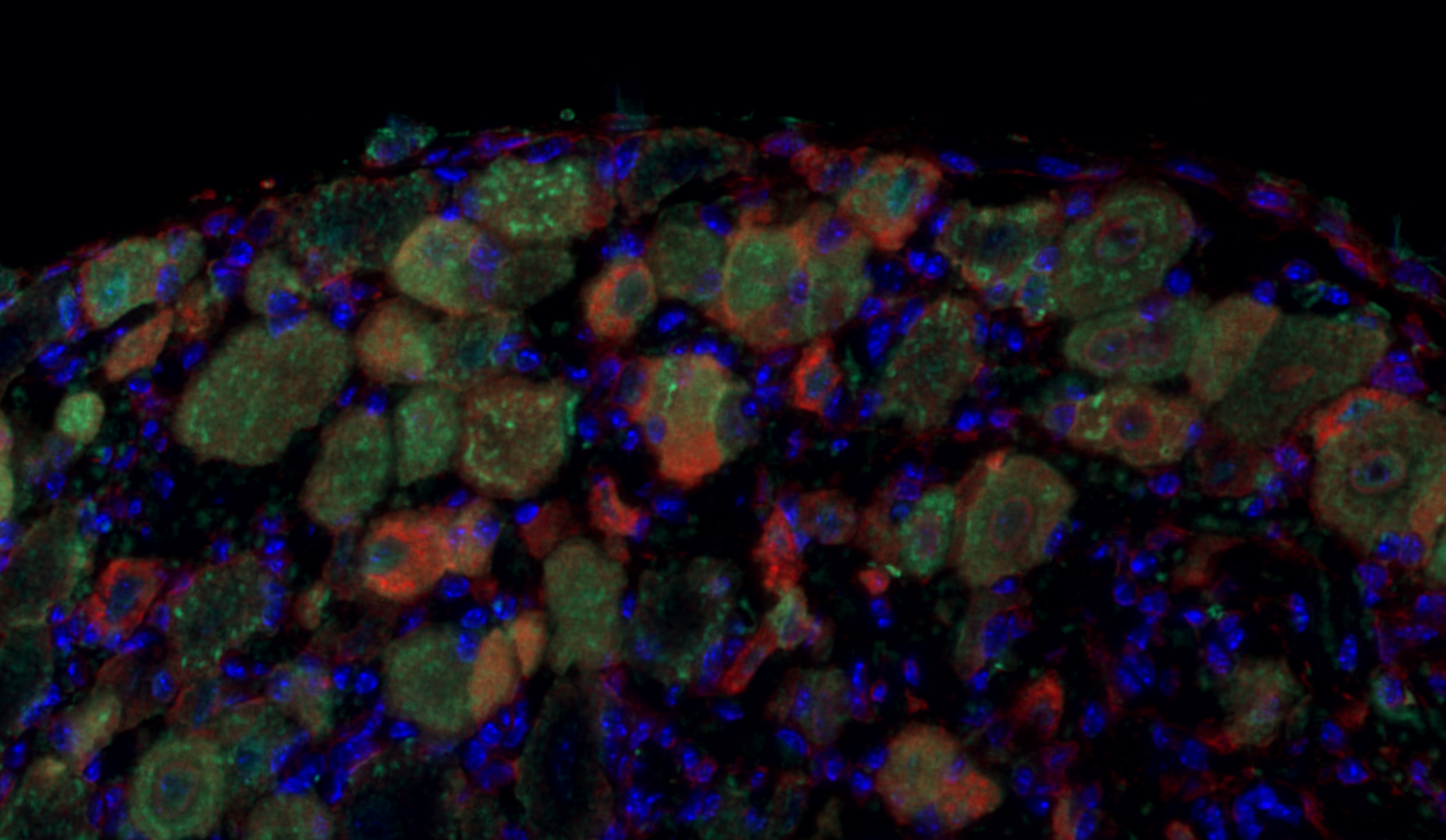

Supplement: Supplementary file 11 — Figure EV3 Source Data [file 44321_2025_268_MOESM11_ESM.zip › Figure EV3/EV3G/TAC1 Apoe fl DMM.jpg]

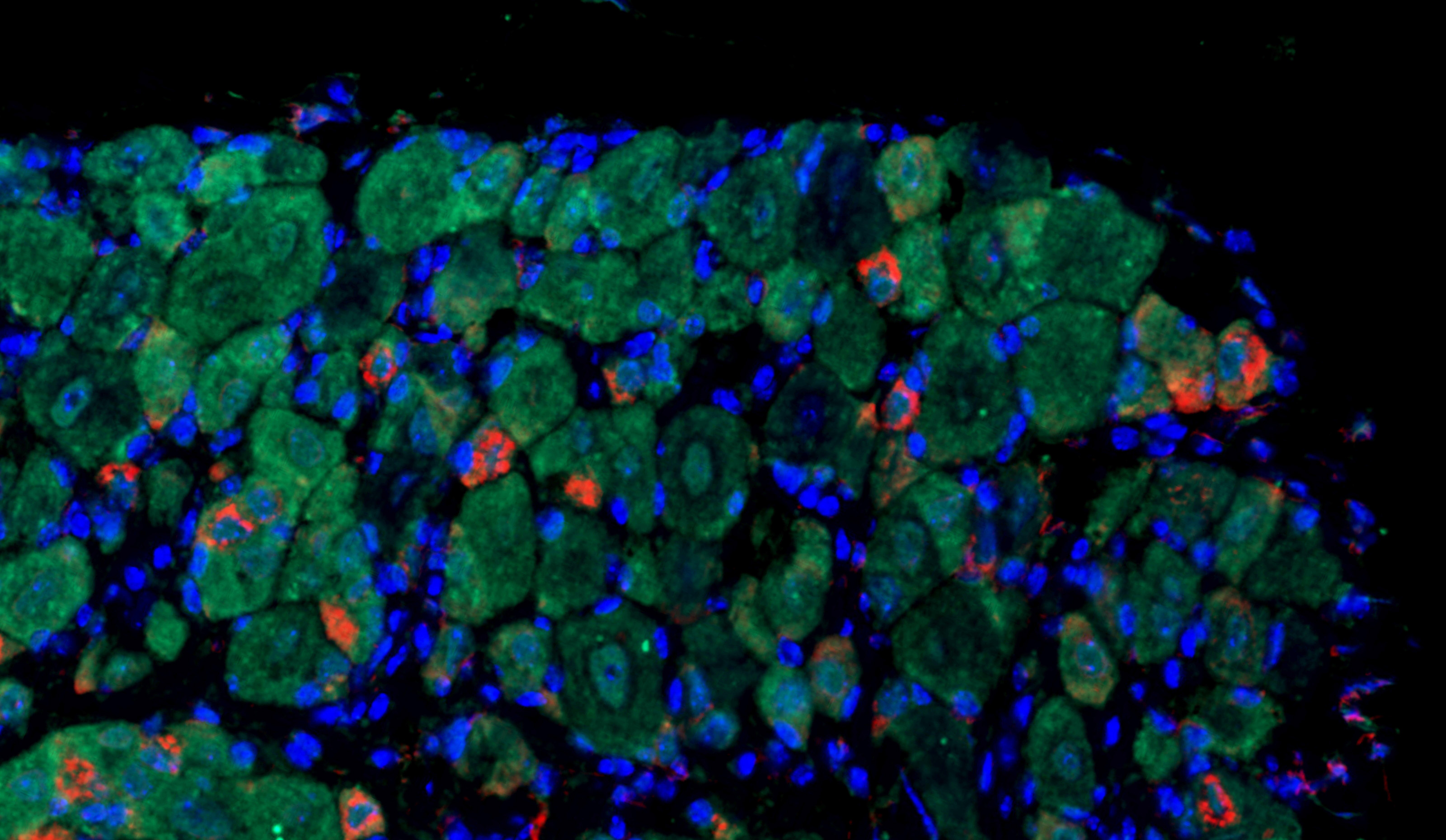

Supplement: Supplementary file 11 — Figure EV3 Source Data [file 44321_2025_268_MOESM11_ESM.zip › Figure EV3/EV3G/TAC1 Apoe fl Sham.jpg]
