# Supplementary material for: Cold exposure promotes the progression of osteoarthritis through downregulating APOE in cartilage
Source: EMBO Mol Med. 2025 Jul 15;17(8):2137–62. doi: 10.1038/s44321-025-00268-6 (PMC12340072; doi:10.1038/s44321-025-00268-6)
Supplement: Supplementary file 13 — Expanded View Figures [file 44321_2025_268_MOESM13_ESM.pdf]

Expanded View Figures

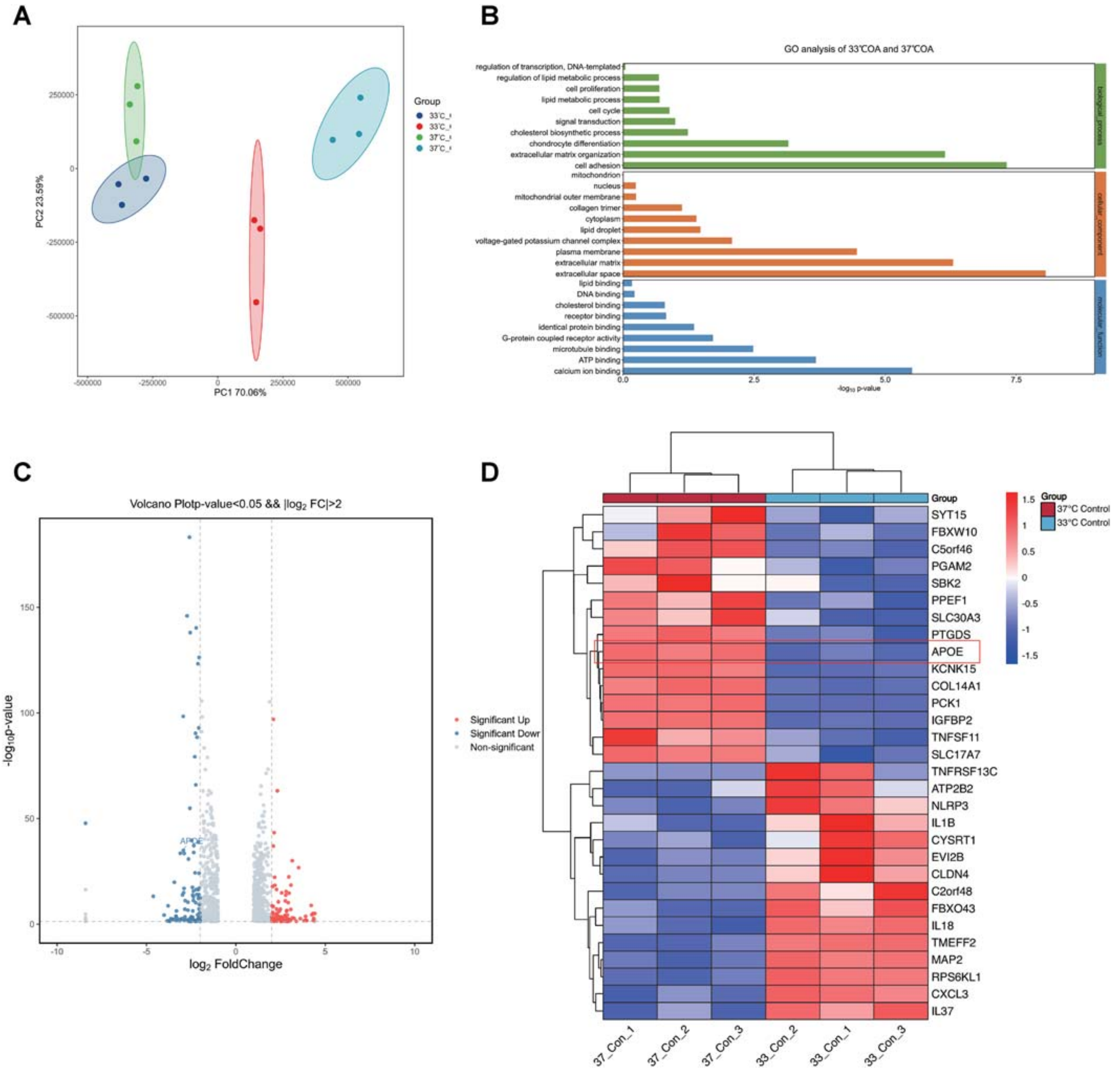

Figure EV1. Bioinformatics analysis of RNA sequencing of chondrocytes cultured at different temperatures.

(**A**) Principal Component Analysis (PCA) of the gene expression. (**B**) GO analysis of the DEGs. (**C**) Volcano plot of gene expressions between 33 °C control group and 37 °C control group ($n = 3$ patients per group). (**D**) Heatmap of DEGs between 33 °C control group and 37 °C control group. Statistical analysis was performed using the hypergeometric test (**B**) and the Wald test within the DESeq2 framework (**C**).

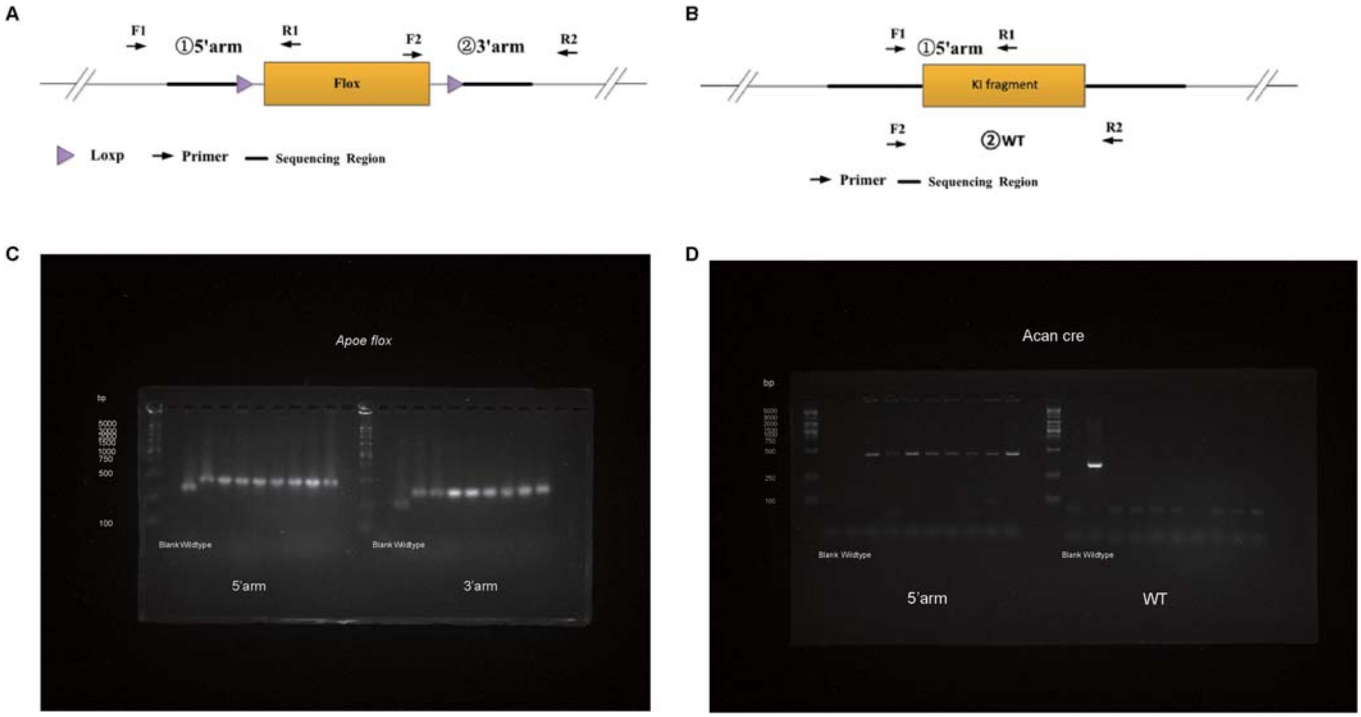

Figure EV2. Result of the genotyping of *Apoe*$^{-/-}$*Acan*$^{Cre\text{-}ERT2}$ mice.

(**A**) Genotyping strategy for *Apoe*$^{f/f}$. (**B**) Genotyping strategy for *Acan*$^{Cre\text{-}ERT2}$. (**C**) Genotyping result for *Apoe*$^{f/f}$. (**D**) Genotyping result for *Acan*$^{Cre\text{-}ERT2}$.

 © The Author(s)

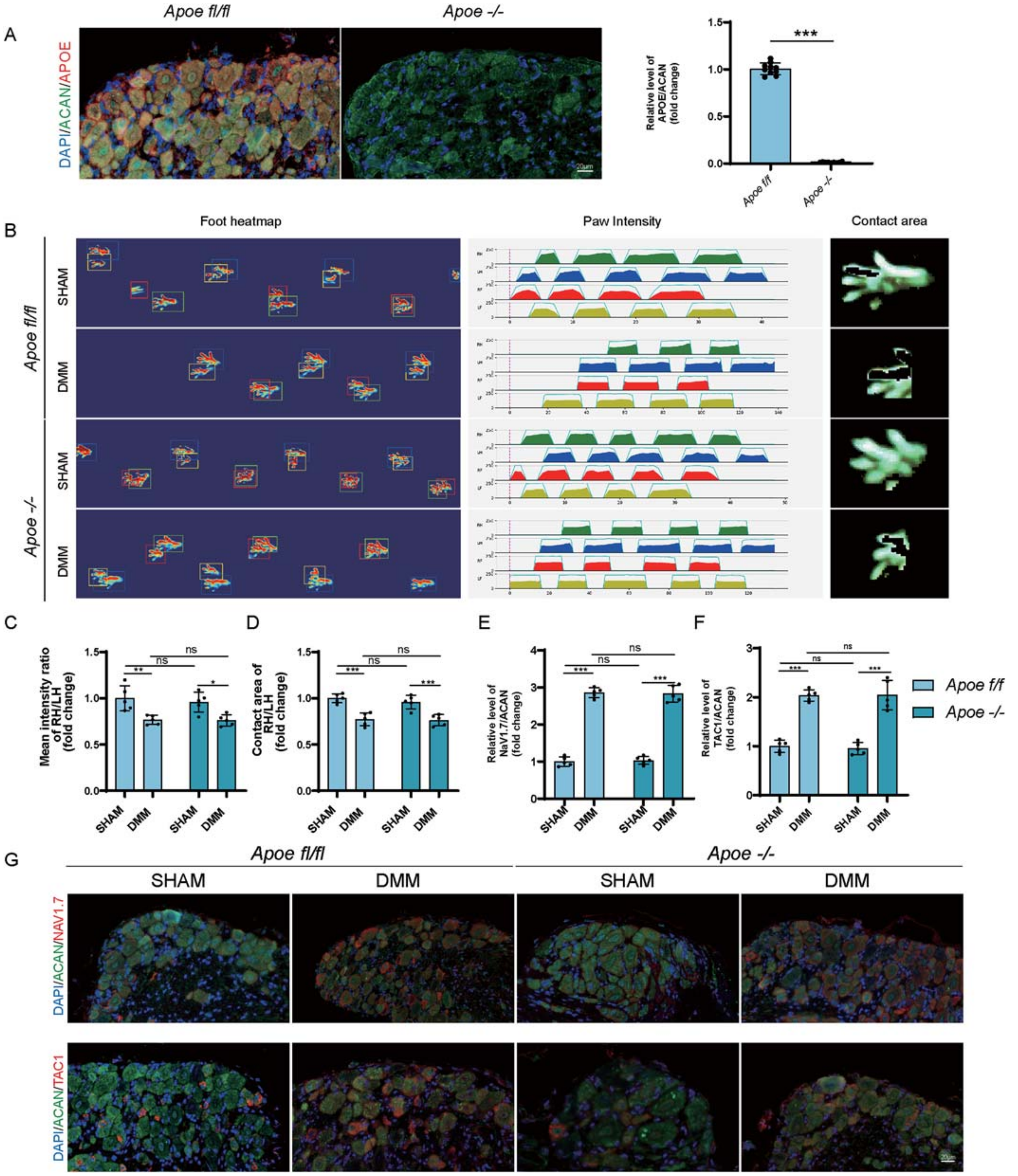
A
Apoe fl/fl
Apoe -/-
DAPI/ACAN/APOE
Relative level of APOE/ACAN (fold change)
***
Apoe f/f
Apoe -/-
B
Foot heatmap
Paw Intensity
Contact area
Apoe fl/fl
Apoe -/-
SHAM
DMM
C
Mean intensity ratio of RH/LH (fold change)
D
Contact area of RH/LH (fold change)
E
Relative level of NaV1.7/ACAN (fold change)
F
Relative level of TAC1/ACAN (fold change)
ns
Apoe f/f
Apoe -/-
G
DAPI/ACAN/NAV1.7
DAPI/ACAN/TAC1

Figure EV3. Analysis of catwalk gait parameters and immunohistochemical staining for pain-related markers in dorsal root ganglia of *Apoe*$^{f/f}$ and *Apoe*$^{-/-}$ mice.

(**A**) Immunofluorescence staining and quantitative analysis of ACAN and APOE co-expression in dorsal root ganglia ($n = 5$). $P < 0.0001$. (**B**) Catwalk gait analysis of *Apoe* $^{f/f}$ and *Apoe*$^{-/-}$ mice and typical result of foot heatmap, paw intensity and contact area. (**C**) Quantitative analysis of mean intensity ratio of right hind (RH) / left hind (LH) limb ($n = 5$). $P = 0.0065$ (*Apoe* $^{f/f}$ SHAM vs. *Apoe* $^{f/f}$ DMM), $P = 0.0224$ (*Apoe* $^{-/-}$ SHAM vs. *Apoe* $^{-/-}$ DMM), $P = 0.9032$ (*Apoe* $^{f/f}$ SHAM vs. *Apoe* $^{-/-}$SHAM), $P = 0.9999$ (*Apoe* $^{f/f}$ DMM vs. *Apoe*$^{-/-}$ DMM). (**D**) Quantitative analysis of ratio of contact area of RH/LH ($n = 5$). $P = 0.0002$ (*Apoe*$^{f/f}$ SHAM vs. *Apoe*$^{f/f}$ DMM), $P = 0.0009$ (*Apoe*$^{-/-}$ SHAM vs. *Apoe*$^{-/-}$ DMM), $P = 0.7479$ (*Apoe* $^{f/f}$ SHAM vs. *Apoe*$^{-/-}$ SHAM), $P = 0.9954$ (*Apoe* $^{f/f}$ DMM vs. *Apoe*$^{-/-}$ DMM). (**E**) Quantitative analysis of Nav1.7/ACAN level in DRG ($n = 5$). $P < 0.0001$ (*Apoe* $^{f/f}$ SHAM vs. *Apoe* $^{f/f}$ DMM), $P < 0.0001$ (*Apoe*$^{-/-}$ SHAM vs. *Apoe*$^{-/-}$ DMM), $P = 0.9863$ (*Apoe*$^{f/f}$ SHAM vs. *Apoe* $^{-/-}$ SHAM), $P = 0.9922$ (*Apoe*$^{f/f}$ DMM vs. *Apoe*$^{-/-}$ DMM). (**F**) Quantitative analysis of TAC1/ACAN level in DRG ($n = 5$). $P < 0.0001$ (*Apoe*$^{f/f}$ SHAM vs. *Apoe*$^{f/f}$ DMM), $P < 0.0001$ (*Apoe*$^{-/-}$ SHAM vs. *Apoe*$^{-/-}$ DMM), $P = 0.9805$ (*Apoe*$^{f/f}$ SHAM vs. *Apoe*$^{-/-}$ SHAM), $P = 0.9999$ (*Apoe*$^{f/f}$ DMM vs. *Apoe*$^{-/-}$ DMM). (**G**) Immunofluorescence co-staining of Nav1.7 (voltage-gated sodium channel) and TAC1 (Substance P precursor) with ACAN (Aggrecan) in dorsal root ganglia (DRG) of *Apoe*$^{f/f}$ and *Apoe*$^{-/-}$ mice. Statistical analysis was performed using Whelch's *t* test (**A**), one-way ANOVA test (**C–F**). Data are shown as mean ± SD (error bar) (*$P < 0.05$, **$P < 0.01$,***$P < 0.001$). Source data are available online for this figure.

© The Author(s)

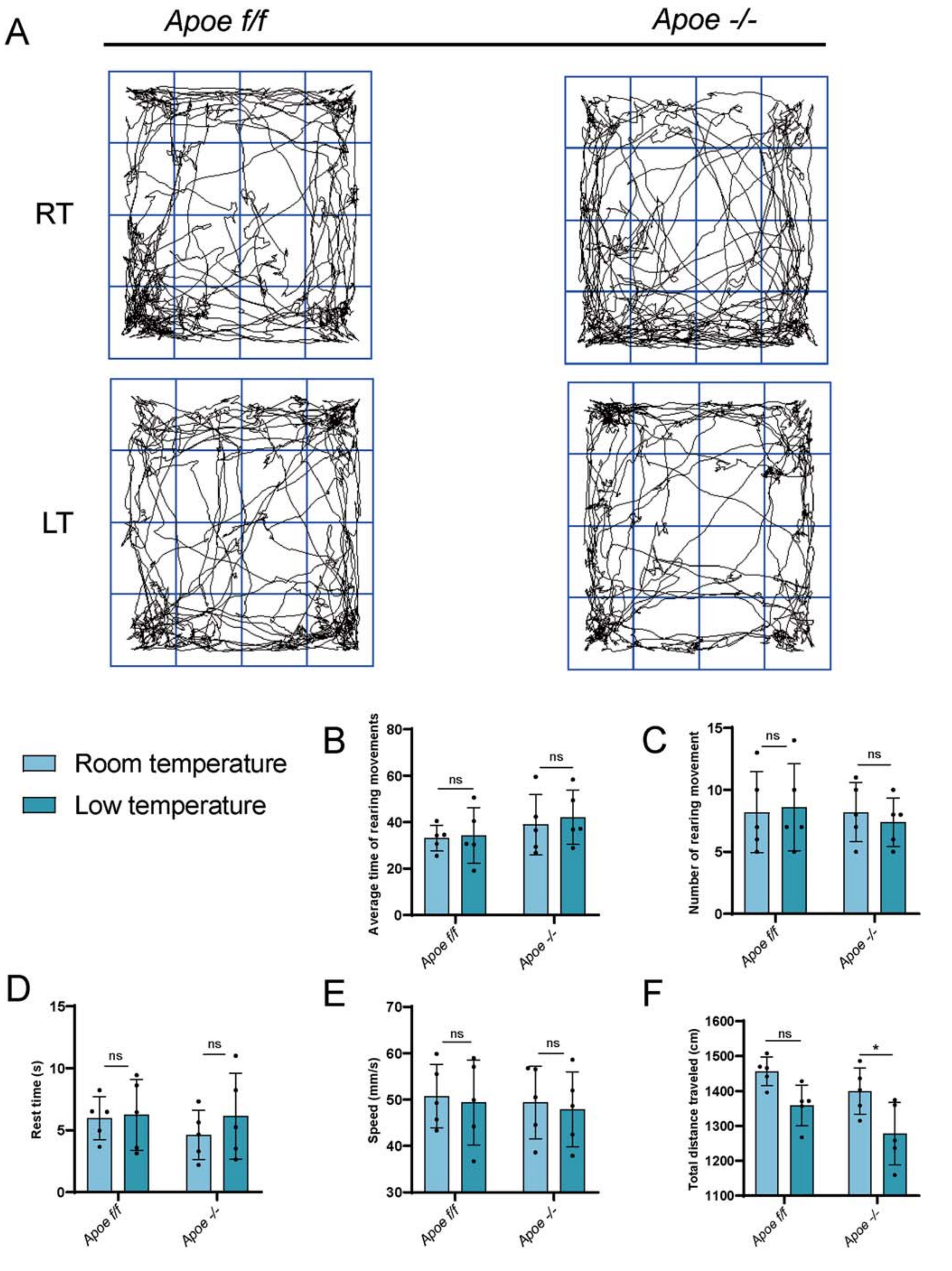
A
Apoe f/f
Apoe -/-
RT
LT
Room temperature
Low temperature
B
Average time of rearing movements
C
Number of rearing movement
D
Rest time (s)
E
Speed (mm/s)
F
Total distance traveled (cm)
ns
*
Apoe f/f
Apoe -/-

Figure EV4. Open filed test of *Apoe* $^{f/f}$ and *Apoe*$^{-/-}$ mice at room and low temperature.

(**A**) Images of the mouse movement pathways. (**B**) Average time of rearing movements ($n = 5$). (**C**) Number of rearing movement ($n = 5$). (**D**) Rest time ($n = 5$). (**E**) Speed ($n = 5$). (**F**) Total distance traveled ($n = 5$). $P = 0.1339$ (*Apoe* $^{f/f}$ RT vs. *Apoe* $^{f/f}$ LT), $P = 0.0449$ (*Apoe*$^{-/-}$ RT vs. *Apoe*$^{-/-}$ LT). Statistical analysis was performed using one-way ANOVA test (**B**, **D–F**), Kruskal–Wallis test (**C**). Data are shown as mean ± SD (error bar) (*$P < 0.05$). Source data are available online for this figure.

© The Author(s)
